# A highly diastereoselective “super silyl” governed aldol reaction: synthesis of α,β-dioxyaldehydes and 1,2,3-triols†
†Electronic supplementary information (ESI) available: For experimental procedures and full compound characterization, including NMR spectra. CCDC 1409678 (**27**) and 1409680 (**3a**). For ESI and crystallographic data in CIF or other electronic format see DOI: 10.1039/c5sc03307a


**DOI:** 10.1039/c5sc03307a

**Published:** 2015-10-06

**Authors:** Wafa Gati, Hisashi Yamamoto

**Affiliations:** a Molecular Catalyst Research Center , Chubu University , 1200 Matsumoto-cho , Kasugai , Aichi 487-8501 , Japan . Email: wafagati@isc.chubu.ac.jp ; Email: hyamamoto@isc.chubu.ac.jp

## Abstract

A highly diastereoselective approach for the synthesis of protected α,β-dioxyaldehydes derived from (*Z*)-tris(trimethylsilyl)silyl “super silyl” enol ethers is described. A general and highly *syn*-stereoselective aldol reaction directed by the “super silyl” group catalyzed by triflimide (HNTf_2_) is developed providing α,β-dioxyaldehydes and 1,2,3-triol fragments which can be a useful platform for the elaboration of natural and unnatural sugar derivatives.

## Abstract 2

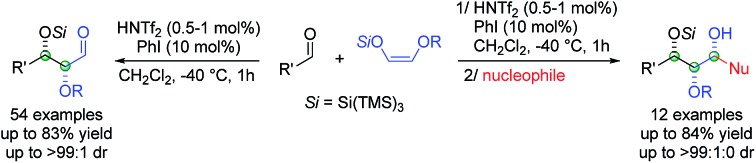
“Super silyl” governed diastereoselective Mukaiyama aldol reaction for the construction of protected α,β-dioxyaldehydes and 1,2,3-triols *via* Lewis acid catalysis.

## Introduction

Polyols are among the most interesting motifs present in various natural and synthetic products. Over the past several decades, organic chemists have made great efforts to invent simpler and more efficient strategies to access stereodefined polyol motifs toward the synthesis of complex sugar frameworks. Because the majority of molecules containing this motif require a multistep protocol for access, chemists have been engaged with creating multiple stereocenters in a one-pot procedure. Although there are numerous routes to C–C bond formation, the aldol reaction remains the most promising and straightforward method for creating two new adjacent stereogenic centers toward the construction of the required polyol subunits.^1^,^2^

Recently our group has actively investigated the Mukaiyama aldol reaction of tris(trimethylsilyl)silyl “super silyl” enol ethers for the highly diastereoselective synthesis of β-super siloxy aldehydes and α-halo-β-super siloxy aldehydes employing Lewis acid catalysis.^3^ This efficient methodology allows for a rapid and stereoselective construction of mono-, bis- and tris-hydroxyaldehydes through mono, double and triple cross aldol processes, respectively, affording polyketide-like scaffolds which are particularly useful for the oriented construction of complex natural polyketides. In our continuous studies on Mukaiyama aldol reactions of super silyl enol ethers, we questioned whether a similar strategy might provide access to α,β-dioxygenated aldehydes which could be a useful building block for construction of complex sugar moieties.^4^

## Results and discussion

Herein we describe the first highly diastereoselective aldol reaction with dioxy enol ethers to give protected α,β-dioxygenated aldehydes in moderate to good yields and with exclusively high *syn* selectivities.

**Scheme 1:**
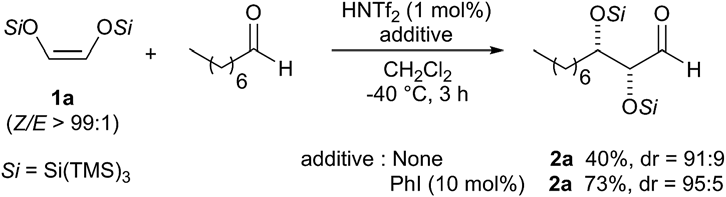
Influence of the additive on the aldol reaction. Yields of isolated aldehydes are shown. The dr values are determined from crude ^1^H NMR.

The super silyl enol ether derived from silyloxy acetaldehyde^5^ was prepared according to the general procedure recently developed in our laboratory.2*a* We began our studies by establishing optimal conditions for the Mukaiyama aldol reaction of bissuper silyloxy enol ether **1a** with 1-octanal using 1 mol% of HNTf_2_ as catalyst in dichloromethane at –40 °C (Scheme 1).

**Scheme 2:**
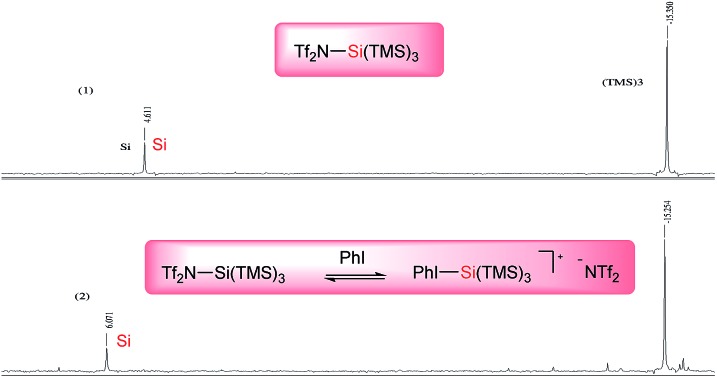
^29^Si NMR study on the influence of iodobenzene on the aldol reaction. 1 equiv. of iodobenzene was used. Experiments conducted in NMR tube in CD_2_Cl_2_ under nitrogen atmosphere and at room temperature.

We were pleased to find that the aldol adduct was obtained in high diastereoselectivity (dr = 91 : 9) but with moderate yield (40%). Thus, in an attempt to optimize the conditions we performed the reaction in the presence of 10 mol% of iodobenzene, which has previously been found to be very useful for increasing the reactivity and the rate of the aldol reaction.3*d* Gratifyingly, we found that the reaction works more efficiently and the adduct **2a** was obtained with much better yield (73%) and a slightly improved diastereoselectivity (dr = 95 : 5). Although we are not sure about the exact role of iodobenzene, we believe that it acts as a co-catalyst that stabilizes the silylenium cation formed *in situ*. Because the additive seemed to be playing a critical role in affecting the rate of the reaction, we conducted a ^29^Si NMR study with the hypothesis that [PhI-Si(TMS)_3_]^+^ is the real active catalytic species. We first recorded a reference ^29^Si NMR spectrum using a simple test substrate (allyltris(trimethylsilyl)silane) in the presence of triflimide (Scheme 2, (1)). We detected a first singlet corresponding to three trimethylsilyl groups that appears at –15.35 ppm and a second singlet corresponding to central silicon that appears at 4.61 ppm. Iodobenzene was then added to the NMR tube and a second ^29^Si NMR spectrum was recorded after 45 min at room temperature.

Surprisingly, we found that the second singlet was shifted up to 6.07 ppm (Scheme 2, (2)). Surprised by the large effect that iodobenzene had on the outcome of the NMR experiment, we decided to perform other experiments varying the stoichiometry and the reaction time. Interestingly, we found that with higher amounts of iodobenzene, the silicon shift is more pronounced, and the singlets also shift more with longer reaction times (see ESI†). To the best of our knowledge, this is the first NMR proof of the role of organoiodide compounds in the Mukaiyama aldol reaction and the ^29^Si NMR study was proof of our principle considering [PhI-Si(TMS)_3_]^+^ as a more active catalytic species than Tf_2_N-Si(TMS)_3_.

**Scheme 3:**
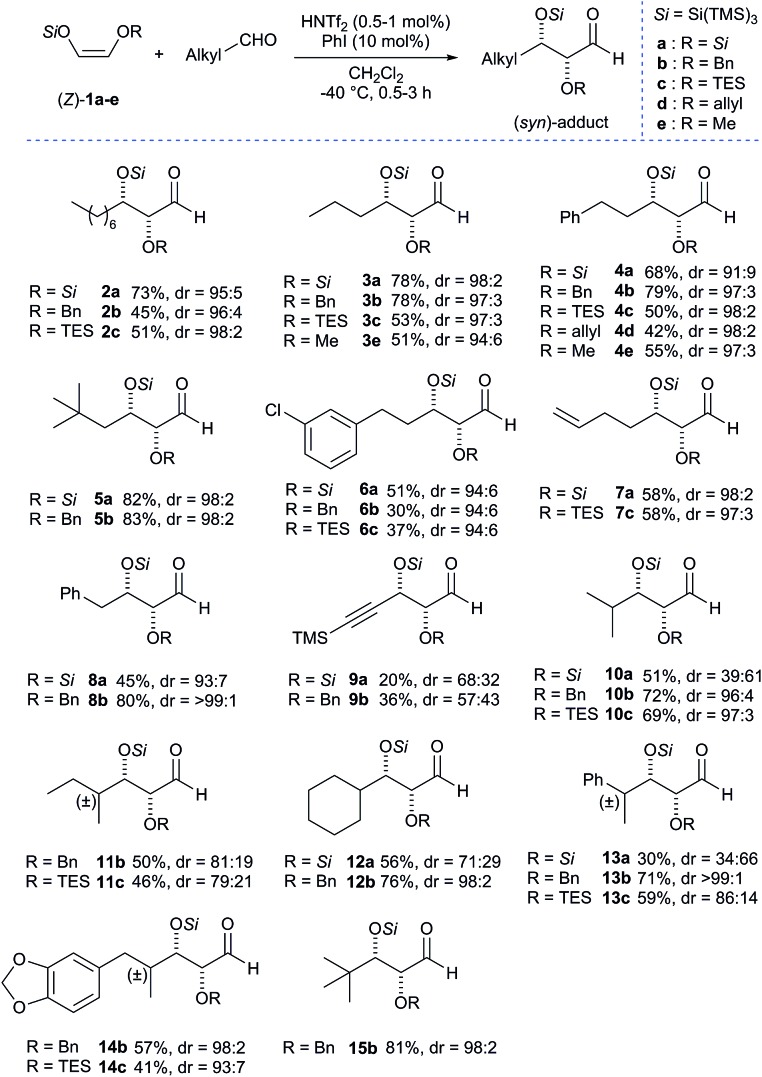
Synthesis of protected α,β-dioxyaldehydes: substrate scope of aliphatic aldehydes. Unless otherwise noted, all reactions were carried out on a 0.2 mmol scale. Yields of isolated aldehydes are shown. The dr values are based on the integration of the ^1^H NMR signals of crude material. The attribution of *syn* and *anti*-ratios was based on the coupling constants of characteristic protons.

Satisfied with these results, we applied our general conditions to the reaction of various super silyl enol ethers with a broad array of aldehydes to afford protected α,β-dioxygenated aldehydes (Scheme 3). Most linear aliphatic aldehydes reacted very smoothly and selectively with super silyl enol ether **1a–e** providing the desired α,β-dioxyaldehydes (**2–8**) in moderate to high yields (up to 83% for compound **5b**) and with excellent and exclusive *syn*-selectivities (up to 98 : 2). Fortunately, the major diastereomer of compound **3a** was crystalline, and the *syn* stereochemistry was directly determined from X-ray analysis.^6^ An aldehyde bearing an unsaturation (alkynyl group) in alpha to the carbonyl group was also tested and found to react rather sluggishly with super silyl enol ethers **1a** and **1b** to afford the corresponding adducts **9a** and **9b** respectively with low yields and poor selectivity. We next investigated aliphatic aldehydes bearing an additional substitution in alpha to the carbonyl which were also tolerated but with moderate yields (up to 51%) and selectivities (up to 71 : 29 dr) (**10a**, **12–13a**) due to the presence of the extraordinarily bulky silyloxy group. Nevertheless when we tested these branched aldehydes with a less bulky silyl enol ether by substitution of one of the super silyloxy groups with a benzyloxy (**1b**) or a triethylsilyloxy group (**1c**), we found that the previously obtained yields and diastereoselectivities were incredibly improved (**10a***vs.***10b** and **10c**, **12a***vs.***12b**, **13a***vs.***13b** and **13c**). (*Z*)-1-Supersilyloxy-2-benzyloxy enol ether **1b** reacted as expected with remarkably high selectivities (up to >99 : 1) and better yields (up to 72%) obtained in almost all products (**2–15**). Notably, pivalaldehyde, which was unreactive with other super silyl enol ethers, was found to react smoothly with (*Z*)-1-supersilyloxy-2-benzyloxy enol ether **1b** to afford the corresponding aldol adduct **15b** with excellent yield and diastereoselectivity (81%, dr = 98 : 2). On the other hand, (*Z*)-1-supersilyloxy-2-triethylsilyloxy enol ether **1c** was found to react less effectively affording the corresponding aldol adducts with diminished yields, probably due to the competitive reaction of the triethylsilyloxy group with our catalyst, although we did not observe the formation of the corresponding regioisomer, and with no remarkable changes in the diastereoselectivity ratios obtained with **1a** or **1b**. Subsequently, additional super silyl enol ethers bearing allyloxy (**1d**) or methoxy (**1e**) groups were also briefly investigated. We subjected (*Z*)-1-allyloxy-2-supersilyloxy enol ether **1d** and (*Z*)-1-methoxy-2-supersilyloxy enol ether **1e** to our optimized reaction conditions, affording the corresponding desired aldol adducts with comparable yields and diastereoselectivities.

**Scheme 4:**
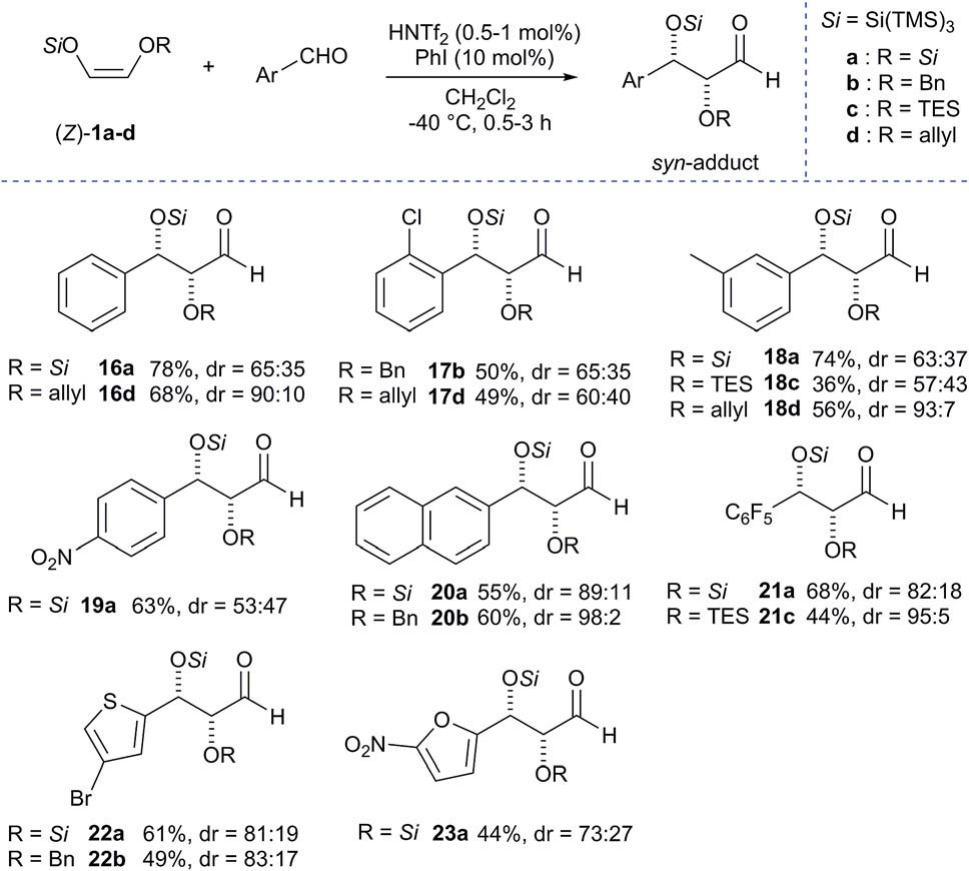
Synthesis of protected α,β-dioxyaldehydes: substrate scope of aromatic aldehydes. Unless otherwise noted, all reactions were carried out on a 0.2 mmol scale. Yields of isolated aldehydes are shown. The dr values are based on the integration of the ^1^H NMR signals of crude material. The attribution of *syn* and *anti*-ratios was based on the coupling constants of characteristic protons.

After the exploration of the scope of aliphatic aldehydes, we next turned our attention to the scope of aromatic aldehydes which were found to be more challenging. When we first investigated the reactivity of benzaldehyde with **1a** using 1 mol% of triflimide catalyst without any additive, we found that the reaction did not proceed and only trace amounts (<5%) of the desired adduct were detected. However, when the reaction was performed with 10 mol% of iodobenzene, the results were remarkably improved and the reaction provided the desired aldol adduct **16a** in high yield (78%) but with moderate diastereoselectivity (Scheme 4). Despite the encouraging results regarding the increased yield using iodobenzene, all other attempts to improve the diastereoselectivity ratios for aromatic aldehydes failed, probably due to the presence of two very bulky super silyl groups. Even so, we were interested in examining the scope of aromatic substrates with our super silyl enol ethers. The reaction with **1a** was found to have poor selectivity (**16a**, **18–23a**) due to steric hindrance with the two silyloxy groups. Nevertheless, we were delighted to find that the diastereoselectivity could be improved up to 98% (for compound **20b**) starting from **1b** and up to 93% (for compound **18d**) starting from **1d**. Then we considered the use of heteroaromatic aldehydes and experiments have shown that an electron-withdrawing group on the heteroaromatic ring is necessary for the reaction to proceed. Our scope was then extended and compounds **22a-b** and **23a** were obtained in acceptable yields and diastereoselectivities. It is worth noting that all the protected *syn*-α,β-dioxyaldehydes obtained are stable in almost all cases and can be kept for weeks in the freezer, since these compounds are known to be rather sensitive to both elimination and epimerisation.

The scope of the reaction was further examined by reacting an optically pure aldehyde with different super silyl enol ethers. In this case, it is known that the stereochemical outcome of the reaction can be controlled by the chirality of the substrate (1,2-asymmetric induction).^7^,^8^

**Scheme 5:**
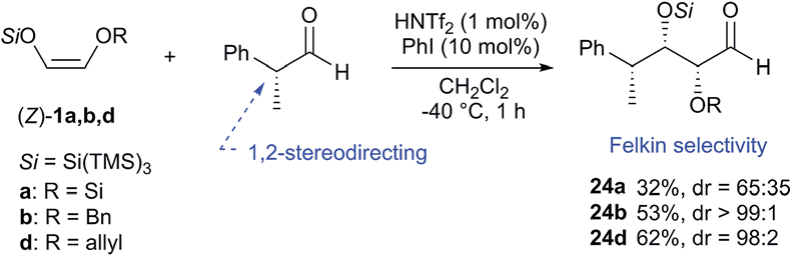
1,2-Stereodirected aldol reaction. Unless otherwise noted, all reactions were carried out on a 0.2 mmol scale. Yields of isolated aldehydes are shown. The dr values are based on the integration of the ^1^H NMR signals of crude material. The attribution of *syn* and *anti*-ratios was based on the coupling constants of characteristic protons.

Indeed, the use of (*R*)-2-phenylpropanal exhibited, as expected, a high Felkin control in conjunction with *syn* selectivity to afford **24a,b,d** with three adjacent stereocenters in excellent diastereoselectivity ratios (up to >99 : 1 *syn*–*syn* for compound **24b**) (Scheme 5).^9^

Next, we investigated the possibility of subsequent one-pot sequential transformation of the obtained protected α,β-dioxygenated aldehydes (Table 1). The addition of alkyl, vinyl, alkynyl, thiophen-2-yl or aryl Grignard reagents to the crude material proceeded smoothly to afford trishydroxy products **25–34** with good to excellent yields (50–84%) and exceptionally high *syn*–*syn* diastereoselectivities (>99 : 1 : 0) which was confirmed by single crystal X-ray analysis of triol **27**.^10^ In the same fashion as in Scheme 5, we considered the use of an aldehyde with a defined α-stereocenter for a 1,2-asymmetric induction investigation. After reaction of (*R*)-2-phenylpropanal with super silyl enol ether **1b** and addition of phenylmagnesium chloride we obtained the desired triol **33** in moderate yield (50%) and diastereoselectivity (dr = 83 : 17 : 0).

**Table 1:** Diastereoselective one-pot sequential reactions^*a*^

Entry	*R*	*R*′	Nucleophile^*b*^	Major product	%Yield^*c*^ (dr)^*d*^
1	Bn	CH_2_CH_2_Ph	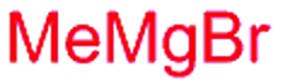	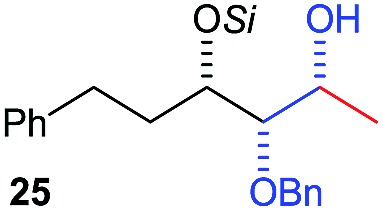	84% (dr > 99 : 1 : 0)
2	Bn	CH_2_CH_2_Ph	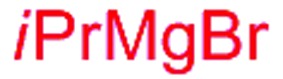	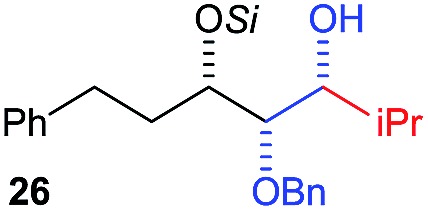	81% (dr > 99 : 1 : 0)
3	Bn	CH_2_Ph	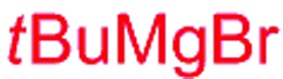	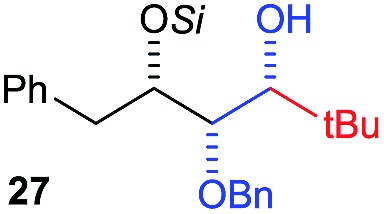	78% (dr > 99 : 1 : 0)
4	Bn	CH_2_Ph	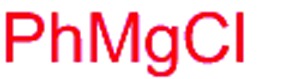	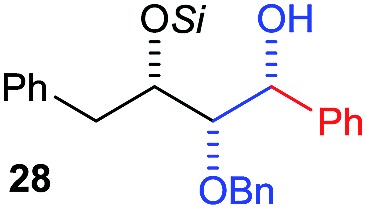	59% (dr > 99 : 1 : 0)
5	Bn	CH_2_Ph	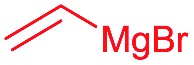	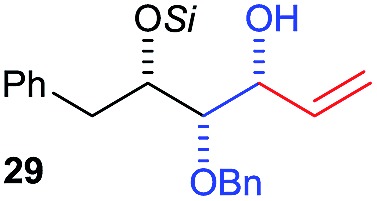	68% (dr > 99 : 1 : 0)
6	Bn	CH_2_CH(CH_3_)_2_	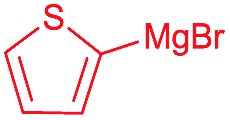	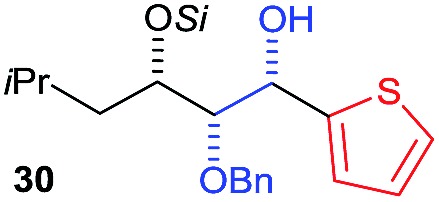	75% (dr > 99 : 1 : 0)
7	Allyl	CH_2_CH_2_Ph	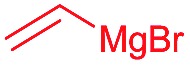	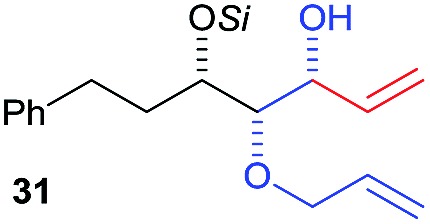	82% (dr > 99 : 1 : 0)
8	Allyl	CH_2_CH(CH_3_)_2_	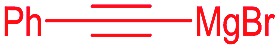	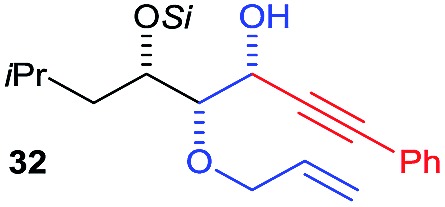	52% (dr = 88 : 12 : 0)
9	Bn	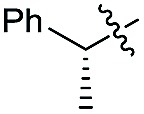	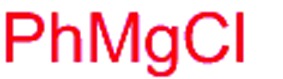	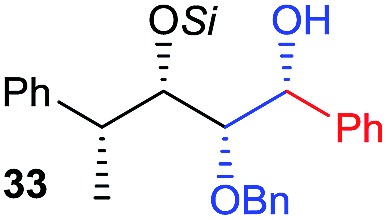	50% (dr = 83 : 17 : 0)
10	Allyl	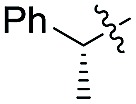	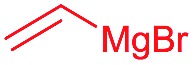	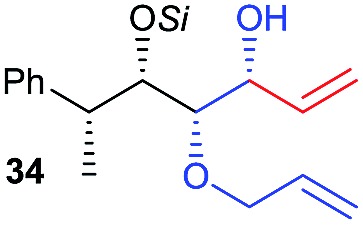	63% (dr > 99 : 1 : 0)
11^*e*^	Bn	CH_2_(CH_3_)_3_	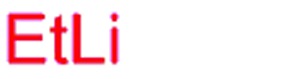	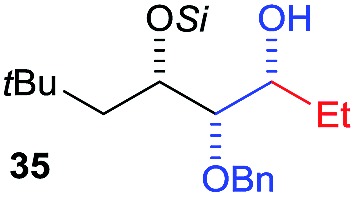	33% (dr > 99 : 1 : 0)
12^*e*^	Allyl	CH_2_(CH_3_)_3_	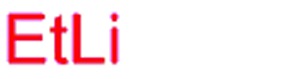	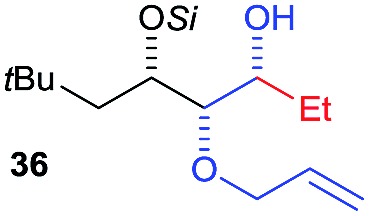	36% (dr > 99 : 1 : 0)

^*a*^Unless otherwise noted, all reactions were carried out on a 0.2 mmol scale.

^*b*^1.5 equiv. of nucleophile was used.

^*c*^Yields of isolated products are shown.

^*d*^The dr are based on the integration of the ^1^H NMR signals of crude material.

^*e*^The reaction was slowly warmed to –20 °C after addition of 2.0 equiv. of nucleophile.

Then we decided to test the more reactive (*Z*)-supersilyloxy-2-allyloxy enol ether **1d** and introduce an additional vinyl group, as it is a rather valuable handle for further transformations. By the addition of a vinyl Grignard reagent we were delighted to easily isolate the synthetically useful vinylic triol **34** generating three new adjacent stereocenters in a one-pot sequential manner in 63% yield and excellent all-*syn* diastereoselectivity (>99 : 1 : 0). Moreover, the reaction was also successful using the lithiated nucleophile EtLi affording the desired alkyl triols **35** and **36** in high diastereoselectivity but with dramatically decreased yields (33% and 36% respectively).

**Scheme 6:**
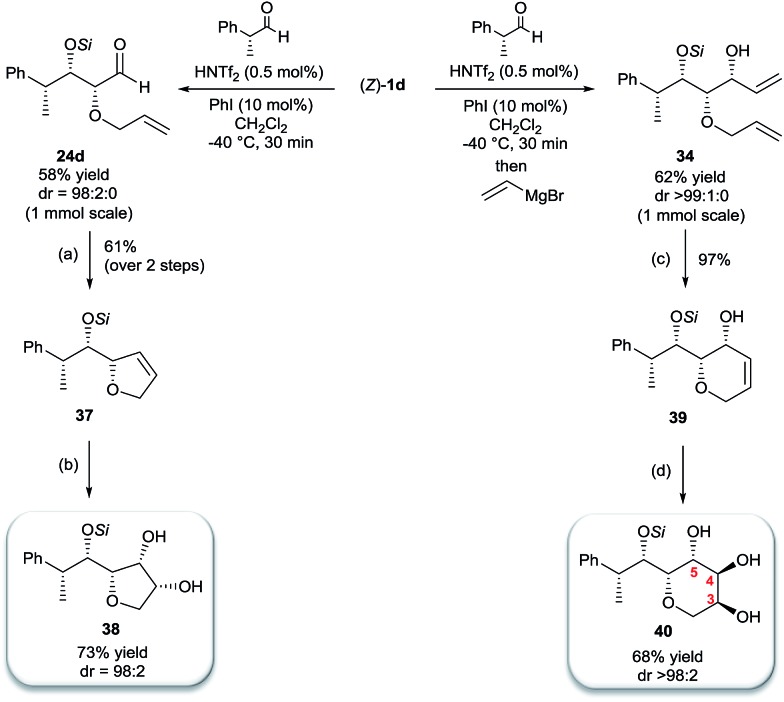
Synthesis of pentose and hexose-like scaffolds. (a) (1) CH_3_PPh_3_, *n*-BuLi, THF (2) Grubbs 2^nd^ generation (2 mol%), CH_2_Cl_2_, 40 °C, 2 h (b) AD-mix-β, MeSO_2_NH_2_, *t*-BuOH/H_2_O (1 : 1), 0 °C, 4 d (c). Grubbs 2^nd^ generation (2 mol%), CH_2_Cl_2_, 40 °C, 2 h (d). OsO_4_, NMO, *t*-BuOH, acetone/H_2_O (1.7 : 1), r.t, 12 h.

Inspired by the important skeleton of the vinylic all-*syn* triol **34** and in an attempt to further probe the utility of our highly diastereoselective one-pot sequential aldol reaction, we targeted pentose and hexose-like scaffolds which are usually difficult to access without employing natural sugar as starting material.^11^ We first applied our strategy to establish the desired α-allyloxy-β-supersilyloxyaldehyde **24d** which was obtained at a slightly decreased yield (58%) on a 1 mmol scale but with no loss of selectivity (98 : 2 *syn*–*syn*). Olefination through Wittig reaction and ring closing metathesis using Grubbs second generation catalyst yielded the five member ring compound **37** in 61% yield (over 2 steps). The last step of the asymmetric dihydroxylation was performed under optimal conditions using catalytic AD-mix-β in biphasic solution at 0 °C for four days^12^ which afforded a single diastereomer of **38** in 73% yield containing five adjacent stereocenters in excellent all-*syn* selectivity (Scheme 6). The stereochemistry of compound **38** was determined based on ^1^H, NOE and NOESY experiments (see the ESI†) in comparison with the literature.

Finally, we considered the possibility of hexose-like scaffold construction, which can be a useful building block to access complex natural and unnatural sugar targets. First we employed our highly diastereoselective Lewis acid catalyzed one-pot sequential aldol scaled-up reaction (1 mmol scale) starting from (*R*)-2-phenylpropanal and silyl enol ether **1d** followed by nucleophilic addition of vinyl magnesium bromide to obtain the desired vinylic triol **34** with no loss of reactivity or diastereoselectivity (62%, 99% dr). Next, a very low loading of the Grubbs second generation catalyst (2 mol%) gave access to six membered ring **39** in excellent yield (97%). A quick optimization of the asymmetric dihydroxylation step (see ESI†) showed that *cis*-osmilation using osmium tetroxide in presence of excess of *N*-methylmorpholine *N*-oxide provided the desired hexose-like structure.^13^ Thus, compound **40**, containing six adjacent stereocenters, was obtained in 68% yield and with an exclusive 4,5-*anti* stereochemistry. The determination of the stereochemistry of the latter compound was based on the optimization reactions where we obtained the same single isomer using both chiral AD-mix-α or β with comparable selectivity but with a slower reaction rate (50% conversion after 4 days), which can be explained by the preferred attack of osmium from the opposite side of the free hydroxy group present in **39**. In addition, a very high coupling constant value (*J*_4-5_ > 10.6 Hz) was detected which emphasizes an *anti*-like relationship between C4–H and C5–H.^14^

## Conclusions

In summary, a very useful strategy to generate synthetically important protected *syn*-α,β-dioxyaldehydes using Lewis acid catalysis has been described. To the best of our knowledge, this is the first synthesis of α-hydroxyaldehydes using Mukaiyama aldol reaction. Furthermore, a ^29^Si NMR study was performed providing the first proof of the role of iodobenzene as additive in increasing the reactivity of the active silylenium cation formed *in situ*. Since the ability of using different protecting groups in the same molecule is an attractive tool to discriminate among chemically similar hydroxyl groups, super silyloxy, benzyloxy, triethylsilyloxy, allyloxy and methoxy have proved to be suitable for the construction of α,β-dioxyaldehydes and 1,2,3-triols. Various nucleophiles were found to react smoothly in a sequential manner allowing for the highly stereoselective construction of all-*syn* 1,2,3-triols. We have finally demonstrated the utility of our methodology as a key step for the elegant construction of pentose and hexose-like scaffolds. Further applications using super silyl governed aldol reactions targeting complex sugar construction are currently underway in our laboratory and will be reported in due course.

## Supplementary Material

Supplementary information

Crystal structure data
