# Supplementary material for: A highly diastereoselective “super silyl” governed aldol reaction: synthesis of α,β-dioxyaldehydes and 1,2,3-triols
Source: Chem Sci. 2015 Oct 6;7(1):394–9. doi: 10.1039/c5sc03307a (PMC5952264; doi:10.1039/c5sc03307a)
Supplement: Supplementary file 1 [file SC-007-C5SC03307A-s001.pdf]

Electronic Supplementary Material (ESI) for Chemical Science.
This journal is © The Royal Society of Chemistry 2015

A Highly Diastereoselective "Super Silyl" Governed Aldol Reaction:

Synthesis of α,β-Dioxyaldehydes and 1,2,3-Triols

Wafa Gati* and Hisashi Yamamoto*

Molecular Catalyst Research Center, Chubu University, 1200 Matsumoto, Kasugai, Aichi 487-8501, Japan

— Supporting Information —

Table of contents

1. General methods....2

2. Preparation of super silyl enol ethers....2

3. Mukaiyama aldol reaction : synthesis of protected α,β-dioxyaldehydes....3

4. One-pot sequential reaction : synthesis of protected 1,2,3-triols....13

5. Synthesis of the pentose-like scaffold....17

6. Synthesis of the Hexose-like scaffold....19

7. ^{1}H, ^{13}C, ^{19}F, NOE and NOESY spectra of compound **1a** to **40**....22

8. ^{29}Si NMR study on the influence of iodobenzene....101

1. General methods.

Chemicals. Anhydrous THF and CH_2Cl_2 were dried with Glass Contour solvent purification system. Dry *t*BuOH, acetone and *n*-hexane were purchased from WAKO chemicals and used as received. $HNTf_2$ was purchased from Aldrich and used in glove box. All aldehydes were freshly distilled before use in aldol reactions. All other chemicals were purchased from their commercial sources and used as received.

Analytics. NMR spectra were recorded on a JEOL JNM LA-400 (400 MHz for ^{1}H NMR, 100 MHz for ^{13}C NMR and 376 MHz for ^{19}F). Chemical shifts were reported in ppm on the δ scale relative to Me_4Si (δ = 0 for ^{1}H NMR), $CDCl_3$ (δ = 77.2 for ^{13}C NMR), α,α,α-trifluorotoluene (δ = -63.72 for ^{19}F NMR) as an internal reference. Multiplicities are indicated as: s (singlet), br. s (broad singlet), d (doublet), t (triplet), dd (doublet of doublet), dt (doublet of triplet), dq (doublet of quadruplet) or m (multiplet). Coupling constants (*J*) are reported in Hertz (Hz). X-ray crystallographic analysis was performed with a Rigaku XtaLAB mini diffractomer (graphite monochromator, MoKα radiation, λ = 0.71075 Å), ESI mass spectra were measured on a Bruker Daltonics micrOTOF. Optical rotations were measured on an ATAGO AP-300 polarimeter with a path length of 100 mm at 589 nm. Column chromatography was conducted with silica gel 60 N (KANTO CHEMICAL, spherical, neutral, 40-50 or 63-210 μm). For thin-layer chromatography (TLC) analysis Merck precoated TLC plates (silica gel 60 F254 0.25 mm) were used. Visualization was accomplished by UV light (254 nm), I_2, anisaldehyde and $KMnO_4$.

***Si*=Si(TMS)$_3$**

2. Preparation of super silyl enol ethers

GP1. General procedure

To a solution of triflic acid (0.93 mL, 10.5 mmol) in CH_2Cl_2 (20 mL) was added tris(trimethylsilyl)silane (3.08 mL, 10 mmol) at 0 °C. The reaction mixture was stirred at the same temperature for 1h. In a second flask, a mixture of aldehyde[1] (11 mmol) and Et_3N (2.1 mL, 15 mmol) in CH_2Cl_2 (20 mL) was then cooled to –60 °C. The triflic solution was canulated to the reaction vessel at this temperature then cold bath was removed. After being stirred at room temperature until TLC analysis indicated total conversion of starting material, the reaction was quenched by sat. aq. $NaHCO_3$, and extracted with Hexane. The combined organic phase was washed with brine, dried over Na_2SO_4, and concentrated under reduced pressure to give the crude product which was purified by flash column chromatography on silica gel (hexane/CH_2Cl_2 = 100/0-85/15) to give the desired pure compound.

(1a): (*Z*)-1,2-bis(tris(trimethylsilyl)siloxy)-ethene :

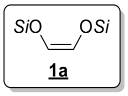

(68%, >99:1 *Z/E*); white solid; TLC (hexane:CH_2Cl_2, 90:10) R_f =0.69; ^{1}H NMR (400 MHz, $CDCl_3$): δ = 5.24 (s, 2H), 0.19 (s, 54H) ppm. ^{13}C NMR (100 MHz, $CDCl_3$): δ = 0.2, 128.6 ppm. HRMS (ESI+) calculated for $C_{20}H_{57}O_2Si_8$ ([M+H]$^+$) : 553.2507, found : 553.2497.

(1b): (*Z*)-1-benzyloxy-2-tris(trimethylsilyl)siloxy-ethene:

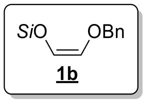

(73%, >99:1 *Z/E*); colorless oil; TLC (hexane:CH_2Cl_2, 80:20) R_f =0.33; ^{1}H NMR (400 MHz, $CDCl_3$): δ = 0.20 (s, 27H), 4.80 (s, 2H), 5.34 (d, *J* = 3.2 Hz, 1H), 5.36 (d, *J* = 3.2 Hz, 1H), 7.27-7.33 (m, 5H) ppm. ^{13}C NMR (100 MHz, $CDCl_3$): δ = 0.2, 73.7, 127.4, 127.7, 128.0, 128.4, 130.5, 138.1 ppm. HRMS (ESI+) calculated for $C_{18}H_{36}O_2Si_4Na$ ([M+Na]$^+$) : 419.1685, found : 419.1688.

(1c): (*Z*)-1-triethylsilyloxy-2-tris(trimethylsilyl)siloxy-ethene:

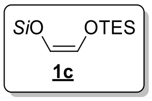

(52%, >99:1 *Z/E*); colorless oil; TLC (hexane:CH_2Cl_2, 90:10) R_f = 0.45; ^{1}H NMR (400 MHz, $CDCl_3$): δ = 0.20 (s, 27H), 0.64 (q, *J* = 8.0 Hz, 6H), 0.96 (t, *J* = 8.2 Hz, 9H), 5.34 (d, *J* = 3.2 Hz, 1H), 5.43 (d, *J* = 3.2 Hz, 1H) ppm. ^{13}C

NMR (100 MHz, $CDCl_3$): δ = 0.1, 4.7, 6.7, 124.3, 129.6 ppm. HRMS (ESI+) calculated for $C_{17}H_{44}O_2Si_5Na$ ($[M+Na]^+$) : 443.2080, found : 443.2084.

(1d): (*Z*)-1-allyloxy-2-tris(trimethylsilyl)siloxy-ethene :

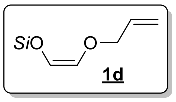

(69%, >99:1 *Z/E*); colorless oil; TLC (hexane:CH_2Cl_2, 80:20) R_f = 0.62 ^{1}H NMR (400 MHz, $CDCl_3$): δ = 0.20 (s, 27H), 4.22 (td, *J* = 1.6, 5.5 Hz 2H), 5.17 (ddd, *J* = 1.4, 3.0, 10.6 Hz, 1H), 5.29 (ddd, *J* = 1.6, 3.4, 17.2 Hz, 1H), 5.30 (d, *J* = 3.4 Hz, 1H), 5.35 (d, *J* = 3.4 Hz, 1H), 5.90 (m, 1H) ppm. ^{13}C NMR (100 MHz, $CDCl_3$): δ = 0.2, 72.7, 117.0, 127.4, 130.5, 134.4 ppm. HRMS (ESI+) calculated for $C_{14}H_{34}O_2Si_4Na$ ($[M+Na]^+$) : 369.1528, found : 369.1531.

(1e): (*Z*)-1-methoxy-2-tris(trimethylsilyl)siloxy-ethene :

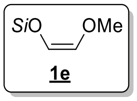

(18%, >99:1 *Z/E*); colorless oil; TLC (hexane:AcOEt, 95:5) R_f = 0.55; ^{1}H NMR (400 MHz, $CDCl_3$): δ = 0.20 (s, 27H), 3.56 (s, 3H), 5.22 (d, *J* = 3.4 Hz, 1H), 5.31 (d, *J* = 3.4 Hz, 1H) ppm. ^{13}C NMR (100 MHz, $CDCl_3$): δ = 0.1, 59.7, 126.9, 132.5 ppm. HRMS (ESI+) calculated for $C_{12}H_{32}O_2Si_4Na$ ($[M+Na]^+$) : 343.1372, found : 343.1370.

3. Mukaiyama aldol reaction : synthesis of protected α,β-dioxyaldehydes

GP2. General procedure

A stirred solution of silyl enol ether (0.2 mmol), aldehyde (0.2 mmol) and iodobenzene (2.3 µL, 0.02 mmol) in anhydrous dichloromethane (2 mL) was cooled to -40 °C. 10 to 20 µL of a fresh solution of triflimide (0.001-0.002 mmol, 0.1 M in CH_2Cl_2) was added dropwise and the solution was stirred at the same temperature for 1 h. After TLC analysis indicated consumption of the starting materials, the reaction was quenched by the addition of saturated aqueous solution of sodium bicarbonate (1 mL). The reaction was allowed to warm to ambient temperature and stirred vigorously for 5 min. The mixture was diluted with 5 mL of hexane and washed with water and brine. The organic layer was dried over sodium sulfate, filtered through cotton and concentrated under reduced pressure. The residue was then purified by flash chromatography on silica gel eluting with Hexane/ CH_2Cl_2 (5% to 40% gradient).

(2a): (2*S*,3*R*)/(2*R*,3*S*)-2,3-bis(tris(trimethylsilyl)siloxy)-decanal :

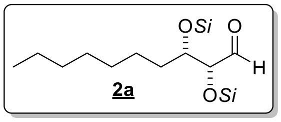

(73%, 95/5 *syn/anti*); white solid; TLC (hexane:CH_2Cl_2, 90:10) R_f = 0.51; ^{1}H NMR (400 MHz, $CDCl_3$): δ = 0.16 (s, 27H), 0.16 (s, 27H), 0.86 (t, *J* = 6.6 Hz, 3H), 1.16-1.32 (m, 10H), 1.32-1.42 (m, 1H), 1.53-1.65 (m, 1H), 3.66 (dt, *J* =4.3, 7.5 Hz, 1H), 3.79 (d, *J* = 4.3 Hz, 1H), 9.72 (s, 1H) ppm. ^{13}C NMR (100 MHz, $CDCl_3$): δ = 0.4, 0.5, 14.1, 22.7, 25.8, 29.3, 30.0, 31.8, 33.2, 79.1, 82.6, 205.0 ppm. HRMS (ESI+) calculated for $C_{28}H_{72}O_3Si_8Na$ ($[M+Na]^+$) : 703.3528, found : 703.3522.

(2b): (2*S*,3*R*)/(2*R*,3*S*)-2-benzyloxy-3-tris(trimethylsilyl)siloxy-decanal :

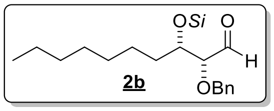

(45%, 96/4 *syn/anti*); colorless oil; TLC (hexane:CH_2Cl_2, 90:10) R_f = 0.45; ^{1}H NMR (400 MHz, $CDCl_3$): δ = 0.16 (s, 27H), 0.86 (t, *J* = 6.8 Hz, 3H), 1.07-1.40 (m, 10H), 1.70-1.73 (m, 2H), 3.70 (dt, *J* =4.4, 6.1 Hz, 1H), 3.79 (dd, *J* = 1.2, 4.4 Hz, 1H), 4.46 (d, *J* = 12.2 Hz, 1H), 4.76 (d, *J* = 12.1 Hz, 1H), 7.25-7.32 (m, 5H), 9.75 (d, *J* = 0.9 Hz, 1H) ppm. ^{13}C NMR (100 MHz, $CDCl_3$): δ = 0.4, 14.2, 22.7, 25.6, 29.3, 29.9, 31.8, 77.8, 78.1, 84.4, 127.9, 128.0, 128.4, 128.8, 137.5, 204.3 ppm. HRMS (ESI+) calculated for $C_{26}H_{52}O_3Si_4Na$ ($[M+Na]^+$) : 547.2886, found : 547.2889.

(2c): (2*S*,3*R*)/(2*R*,3*S*)-2-triethylsilyloxy-3-tris(trimethylsilyl)siloxy-decanal :

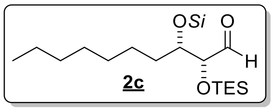

(51%, 98/2 *syn/anti*); colorless oil; TLC (hexane:CH_2Cl_2, 90:10) R_f = 0.27; ^{1}H NMR (400 MHz, $CDCl_3$): δ = 0.18 (s, 27H), 0.56 (qd, *J* = 3.2, 8.5 Hz, 6H), 0.93 (t, *J* = 5.5 Hz, 9H), 1.20-1.31 (m, 15H), 3.65 (dt, *J* = 4.4, 6.6 Hz, 1H), 4.05 (dd, *J* = 0.7, 4.1 Hz, 1H), 9.70 (d, *J* = 0.7 Hz, 1H) ppm. ^{13}C NMR (100 MHz, $CDCl_3$): δ = 0.5, 5.1, 6.8, 14.2, 22.7, 25.9, 29.3, 29.9, 31.8, 33.1, 78.9, 79.4, 204.2 ppm. HRMS (ESI+) calculated for $C_{25}H_{60}O_3Si_5Na$ ($[M+Na]^+$) : 571.3281, found : 571.3256.

(3a): (2*S*,3*R*)/(2*R*,3*S*)-2,3-bis(tris(trimethylsilyl)siloxy)-hexanal :

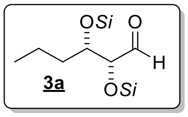

(78%, 98/2 *syn/anti*); colorless crystals (crystallized from methanol); TLC (hexane:CH_2Cl_2, 90:10) R_f = 0.5; ^{1}H NMR (400 MHz, $CDCl_3$): δ = 0.16 (s, 27H), 0.16 (s, 27H), 0.87 (t, *J* = 7.1 Hz, 3H), 1.16-1.40 (m, 3H), 1.54-1.64 (m, 1H), 3.68 (dt, *J* = 4.3, 7.7 Hz, 1H), 3.78 (d, *J* = 4.1 Hz, 1H), 9.71 (s, 1H) ppm. ^{13}C NMR (100 MHz, $CDCl_3$): δ = 0.4, 0.5, 14.3, 19.0, 35.6, 78.5, 82.5, 204.8 ppm. HRMS (ESI+) calculated for $C_{24}H_{64}O_3Si_8Na$ ($[M+Na]^+$) : 647.2902, found : 647.2903

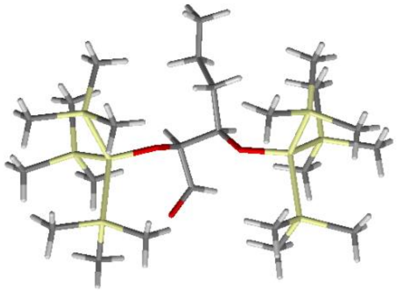

Single crystal X-ray crystallographic analysis for **3a** (CCDC No. 1409680)

Bond precision: C-C = 0.0320 A Wavelength=0.71075

Cell:	a=16.614(19)	b=18.221(19)	c=14.051(16)
	alpha=90	beta=105.69(2)	gamma=90

Temperature: 173 K

	Calculated	Reported
Volume	4095(8)	4095(8)
Space group	P 21/c	P 1 21/c 1
Hall group	-P 2ybc	-P 2ybc
Moiety formula	C24 H64 O3 Si8	C24 H64 O3 Si8
Sum formula	C24 H64 O3 Si8	C24 H64 O3 Si8
Mr	625.47	625.45
Dx,g cm-3	1.015	1.014
Z	4	4
Mu (mm-1)	0.283	0.282
F000	1376.0	1376.0
F000'	1378.89	
h,k,lmax	21,23,18	21,23,18
Nref	9668	9468
Tmin,Tmax	0.945,0.945	0.714,0.945
Tmin'	0.945	

Correction method= # Reported T Limits: Tmin=0.714 Tmax=0.945
AbsCorr = MULTI-SCAN

Data completeness= 0.979 Theta(max)= 27.770

R(reflections)= 0.2228(3829) wR2(reflections)= 0.5310(9468)

S = 1.285 Npar= 306

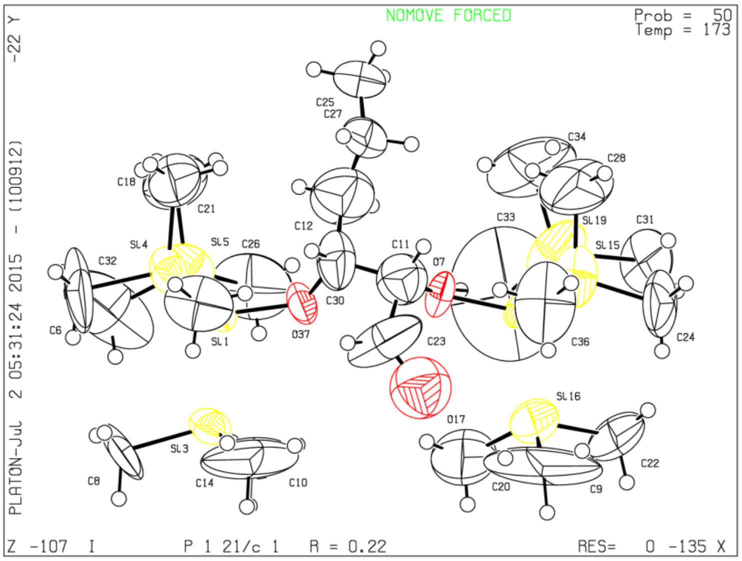

(3b): (2*S*,3*R*)/(2*R*,3*S*)-2-benzyloxy-3-tris(trimethylsilyl)siloxy-hexanal :

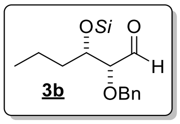

(78%, 97/3 *syn/anti*); colorless oil; TLC (hexane:CH_2Cl_2, 80:20) R_f = 0.26; ^1H NMR (400 MHz, $CDCl_3$): δ = 0.16 (s, 27H), 0.86 (t, *J* = 7.3 Hz, 3H), 1.18-1.40 (m, 3H), 1.68-1.71 (m, 1H), 3.73 (dt, *J* = 4.6, 6.2 Hz, 1H), 3.79 (dd, *J* = 0.9, 4.4 Hz, 1H), 4.47 (d, *J* = 12.2 Hz, 1H), 4.76 (d, *J* = 12.2 Hz, 1H), 9.75 (d, *J* = 1.1 Hz, 1H) ppm. ^{13}C NMR (100 MHz, $CDCl_3$): δ = 0.5, 14.3, 18.9, 35.6, 72.6, 77.7, 84.4, 128.1, 128.2, 128.6, 137.5, 204.1 ppm. HRMS (ESI+) calculated for $C_{22}H_{44}O_3Si_4Na$ ($[M+Na]^+$) : 491.2260, found : 491.2257.

(3c): (2*S*,3*R*)/(2*R*,3*S*)-2-triethylsilyloxy-3-tris(trimethylsilyl)siloxy-hexanal :

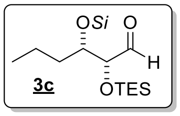

(53%, 97/3 *syn/anti*); colorless oil; TLC (hexane:CH_2Cl_2, 90:10) R_f = 0.19; ^1H NMR (400 MHz, $CDCl_3$): δ = 0.17 (s, 27H), 0.59 (qd, *J* = 2.0, 7.6 Hz, 6H), 0.90 (t, *J* = 7.2 Hz, 3H), 0.93 (t, *J* = 8.0 Hz, 9H), 1.22-1.38 (m, 3H), 1.61-1.69 (m, 1H), 3.66 (m, 1H), 4.05 (dd, *J* = 0.5, 4.2 Hz, 1H), 9.71 (d, *J* = 0.7 Hz, 1H) ppm. ^{13}C NMR (100 MHz, $CDCl_3$): δ = 0.5, 5.0, 6.8, 14.4, 19.1, 35.3, 78.7, 79.4, 204.2 ppm. HRMS (ESI+) calculated for $C_{21}H_{52}O_3Si_5Na$ ($[M+Na]^+$) : 515.2655, found : 515.2652.

(3e): (2*S*,3*R*)/(2*R*,3*S*)-2-methoxy-3-tris(trimethylsilyl)siloxy-hexanal :

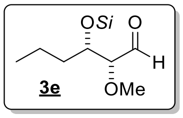

(51%, 94/6 *syn/anti*); colorless oil; TLC (hexane:CH_2Cl_2, 60:40) R_f = 0.46; ^1H NMR (400 MHz, $CDCl_3$): δ = 0.18 (s, 27H), 0.89 (t, *J* = 7.1 Hz, 3H), 1.24-1.40 (m, 3H), 1.57-1.67 (m, 1H), 3.42 (s, 3H), 3.59 (dd, *J* = 1.2, 4.6 Hz, 1H), 3.70-3.75 (m, 1H), 9.72 (d, *J* = 1.2 Hz, 1H) ppm. ^{13}C NMR (100 MHz, $CDCl_3$): δ = 0.6, 14.3, 18.8, 35.5, 58.9, 77.2, 87.6, 203.7 ppm. HRMS (ESI+) calculated for $C_{16}H_{40}O_3Si_4Na$ ($[M+Na]^+$) : 415.1947, found : 415.1948.

(4a): (2*S*,3*R*)/(2*R*,3*S*)-2,3-bis(tris(trimethylsilyl)siloxy)-5-phenylpentanal :

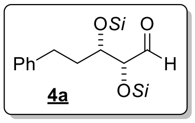

(68%, 91/9 *syn/anti*); colorless oil; TLC (hexane:CH_2Cl_2, 90:10) R_f = 0.56; ^1H NMR (400 MHz, $CDCl_3$): δ = 0.20 (s, 27H), 0.21 (s, 27H), 1.71-1.78 (m, 1H), 1.87-1.97 (m, 1H), 2.45-2.55 (m, 1H), 2.59-2.69 (m, 1H), 3.82 (dt, *J* = 4.8, 7.1 Hz, 1H), 3.97 (d, *J* = 4.8 Hz, 1H), 7.10-7.20 (m, 3H), 7.25-7.30 (m, 2H), 9.83 (s, 1H) ppm. ^{13}C NMR (100 MHz, $CDCl_3$): δ = 0.4, 0.5, 31.9, 35.5, 78.4, 82.8, 125.9, 128.9, 128.5, 141.9, 204.0 ppm. HRMS (ESI+) calculated for $C_{29}H_{66}O_3Si_8Na$ ($[M+Na]^+$) : 709.3058, found : 709.3052.

(4b): (2*S*,3*R*)/(2*R*,3*S*)-2-benzyloxy-3-tris(trimethylsilyl)siloxy-5-phenylpentanal

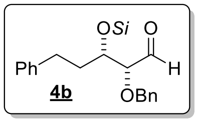

(79%, 97/3 *syn/anti*); colorless oil; TLC (hexane:CH_2Cl_2, 80:20) R_f = 0.38; ^1H NMR (400 MHz, $CDCl_3$): δ = 0.20 (s, 27H), 1.70-1.75 (m, 1H), 2.05-2.15 (m, 1H), 2.50-2.55 (m, 1H), 2.60-2.65 (m, 1H), 3.82-3.87 (m, 1H), 3.91 (dd, *J* = 0.8, 4.4 Hz, 1H), 4.50 (d, *J* = 12.0 Hz, 1H), 4.81 (d, *J* = 12.0 Hz, 1H), 7.14-7.40 (m, 10H), 9.81 (d, *J* = 0.9 Hz, 1H) ppm. ^{13}C NMR (100 MHz, $CDCl_3$): δ = 0.6, 32.0, 35.4, 72.6, 84.3, 126.0, 128.2, 128.3, 128.4, 128.5 (2C), 128.7, 137.4, 141.7, 203.7 ppm. HRMS (ESI+) calculated for $C_{27}H_{46}O_3Si_4Na$ ($[M+Na]^+$) : 553.2416, found : 553.2418.

(4c): (2*S*,3*R*)/(2*R*,3*S*)-2-triethylsilyloxy-3-tris(trimethylsilyl)siloxy-5-phenylpentanal

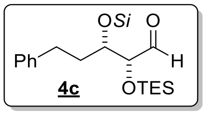

(50%, 98/2 *syn/anti*); colorless oil; TLC (hexane:CH_2Cl_2, 90:10) R_f = 0.24; ^1H NMR (400 MHz, $CDCl_3$): δ = 0.20 (s, 27H), 0.64 (qd, *J* = 2.1, 7.6 Hz, 6H), 0.97 (t, *J* = 8.0 Hz, 9H), 1.60-1.70 (m, 1H), 1.98-2.10 (m, 1H), 2.55-2.71 (m, 2H), 3.77 (dt, *J* = 4.6, 10.6 Hz, 1H), 4.18 (dd, *J* = 0.7, 4.6 Hz, 1H), 7.14-7.20 (m, 3H), 7.25-7.29 (m, 2H), 9.77 (d, *J* = 0.7 Hz, 1H) ppm. ^{13}C NMR (100 MHz, $CDCl_3$): δ = 0.5, 5.1, 6.9, 32.3, 35.2, 78.6, 79.5, 126.0, 128.3, 128.5, 141.9, 203.5 ppm. HRMS (ESI+) calculated for $C_{26}H_{54}O_3Si_5Na$ ($[M+Na]^+$) : 577.2811, found : 577.2812.

(4d): (2*S*,3*R*)/(2*R*,3*S*)-2-allyloxy-3-tris(trimethylsilyl)siloxy-5-phenylpentanal

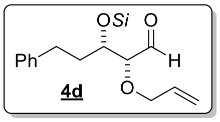

(42%, 98/2 *syn/anti*); colorless oil; TLC (hexane:CH_2Cl_2, 60:40) R_f = 0.37; ^1H NMR (400 MHz, $CDCl_3$): δ = 0.20 (s, 27H), 1.61-1.71 (m, 1H), 2.00-2.08 (m, 1H), 2.57 (td, *J* = 5.3, 11.5 Hz, 1H), 2.66 (td, *J* = 5.5, 11.7 Hz, 1H), 3.81 (dt, *J* = 4.6, 10.8, 1H), 3.87 (dd, *J* = 1.2, 4.8 Hz, 1H), 3.98 (tdd, *J* = 1.2, 6.2, 12.8 Hz, 1H), 4.20 (tdd, *J* = 1.4, 5.3, 12.8 Hz, 1H), 5.22 (ddd, *J* = 1.1, 2.6, 10.4 Hz, 1H), 5.26 (ddd, *J* = 1.6, 3.2, 17.2 Hz, 1H), 5.85-5.95 (m, 1H), 7.14-7.18 (m, 3H), 7.24-7.27 (m, 2H), 9.77 (d, *J* = 1.1 Hz, 1H) ppm. ^{13}C NMR (100 MHz, $CDCl_3$): δ = 0.6, 32.0, 35.4, 72.0, 77.4, 84.6, 118.4, 126.0, 128.4, 128.5, 134.2, 141.7, 203.6 ppm. HRMS (ESI+) calculated for $C_{23}H_{44}O_3Si_4Na$ ($[M+Na]^+$) : 503.2260, found : 503.2251.

(4e): (2*S*,3*R*)/(2*R*,3*S*)-2-methoxy-3-tris(trimethylsilyl)siloxy-5-phenylpentanal

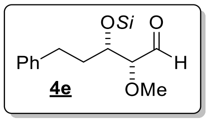

(55%, 97/3 *syn/anti*); colorless oil; TLC (hexane:CH_2Cl_2, 80:20) R_f = 0.45; ^1H NMR (400 MHz, $CDCl_3$): δ = 0.20 (s, 27H), 1.60-1.69 (m, 1H), 1.94-2.03 (m, 1H), 2.53-2.70 (m, 2H), 3.43 (s, 3H), 3.68 (dd, *J* = 1.6, 4.8 Hz, 1H), 3.80 (dt, *J* = 4.8, 10.8 Hz, 1H), 7.14-7.20 (m, 3H), 7.24-7.27 (m, 2H), 9.75 (d, *J* = 1.4 Hz, 1H) ppm. ^{13}C NMR (100 MHz, $CDCl_3$): δ = 0.6, 31.9, 35.3, 58.9, 75.8, 87.5, 126.0, 128.4, 128.5, 141.7, 203.4 ppm. HRMS (ESI+) calculated for $C_{21}H_{42}O_3Si_4Na$ ($[M+Na]^+$) : 477.2103, found : 477.2105.

(5a): (2*S*,3*R*)/(2*R*,3*S*)-2,3-bis(tris(trimethylsilyl)siloxy)-5,5-dimethylhexanal :

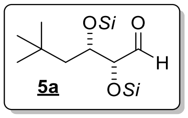

(82%, 98/2 *syn/anti*); colorless oil; TLC (hexane:CH_2Cl_2, 90:10) R_f = 0.54; ^1H NMR (400 MHz, $CDCl_3$): δ = 0.16 (s, 27H), 0.21 (s, 27H), 0.88 (s, 9H), 1.12 (dd, *J* = 6.9, 14.0 Hz, 1H), 1.50 (dd, *J* = 2.8, 14.4 Hz, 1H), 3.70-3.74 (m, 1H), 4.00 (d, *J* = 5.0 Hz, 1H), 9.80 (s, 1H) ppm. ^{13}C NMR (100 MHz, $CDCl_3$): δ = 0.5, 1.1, 30.0, 30.4, 46.9, 76.5, 82.0, 203.9 ppm. HRMS (ESI+) calculated for $C_{26}H_{68}O_3Si_8Na$ ($[M+Na]^+$) : 675.3215, found : 675.3217.

(5b): (2*S*,3*R*)/(2*R*,3*S*)-2-benzyloxy-3-tris(trimethylsilyl)siloxy-5,5-dimethylhexanal :

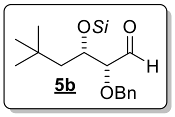

(83%, 98/2 *syn/anti*); colorless oil; TLC (hexane:CH_2Cl_2, 90:10) R_f = 0.49; ^{1}H NMR (400 MHz, $CDCl_3$): δ = 0.19 (s, 27H), 0.91 (s, 9H), 1.25 (dd, *J* = 6.4, 13.8 Hz, 1H), 1.72 (dd, *J* = 4.4, 14.0 Hz, 1H), 3.90-3.94 (m, 2H), 4.50 (d, *J* = 11.7 Hz, 1H), 4.75 (d, *J* = 11.9 Hz, 1H), 7.26-7.35 (m, 5H), 9.74 (s, 1H) ppm. ^{13}C NMR (100 MHz, $CDCl_3$): δ = 0.9, 30.0, 30.3, 46.5, 72.2, 75.3, 84.8, 127.8, 127.9, 128.5, 137.8, 203.9 ppm. HRMS (ESI+) calculated for $C_{24}H_{48}O_3Si_4Na$ ($[M+Na]^+$) : 519.2573, found : 519.2577.

(6a): (2*S*,3*R*)/(2*R*,3*S*)-2,3-bis(tris(trimethylsilyl)siloxy)-5-(3-chlorophenyl) pentanal :

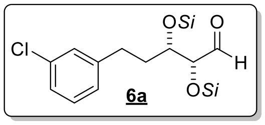

(51%, 94/6 *syn/anti*); colorless oil; TLC (hexane:CH_2Cl_2, 90:10) R_f = 0.42; ^{1}H NMR (400 MHz, $CDCl_3$): δ = 0.18 (s, 27H), 0.19 (s, 27H), 1.64-1.74 (m, 1H), 1.80-1.89 (m, 1H), 2.44-2.52 (m, 1H), 2.55-2.63 (m, 1H), 3.77 (dt, *J* = 5.0, 6.9 Hz, 1H), 3.95 (d, *J* = 5.0 Hz, 1H), 6.98 (d, *J* = 7.1 Hz, 1H), 7.10-7.20 (m, 3H), 9.80 (s, 1H) ppm. ^{13}C NMR (100 MHz, $CDCl_3$): δ = 0.4, 0.5, 35.3, 78.0, 78.4, 82.8, 126.0, 126.8, 128.1, 129.8, 134.3, 144.0, 203.9 ppm. HRMS (ESI+) calculated for $C_{29}H_{65}O_3Si_8ClNa$ ($[M+Na]^+$) : 743.2669, found : 743.2660.

(6b): (2*S*,3*R*)/(2*R*,3*S*)-2-benzyloxy-3-tris(trimethylsilyl)siloxy-5-(3-chlorophenyl) pentanal :

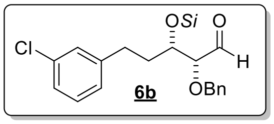

(30%, 94/6 *syn/anti*); colorless oil; TLC (hexane:CH_2Cl_2, 80:20) R_f = 0.37; ^{1}H NMR (400 MHz, $CDCl_3$): δ = 0.18 (s, 27H), 1.59-1.68 (m, 1H), 1.97-2.06 (m, 1H), 2.40-2.49 (m, 1H), 2.53-2.62 (m, 1H), 3.77 (dt, *J* = 4.8, 6.2 Hz, 1H), 3.88 (dd, *J* = 1.2, 4.8 Hz, 1H), 4.45 (d, *J* = 11.9 Hz, 1H), 4.78 (d, *J* = 11.9 Hz, 1H), 7.00-7.18 (m, 4H), 7.30-7.35 (m, 5H), 9.78 (d, *J* = 1.1 Hz, 1H) ppm. ^{13}C NMR (100 MHz, $CDCl_3$): δ = 0.5, 31.5, 35.1, 72.6, 84.2, 126.2, 126.5, 126.6, 128.3, 128.3, 128.5, 128.7, 129.7, 134.2, 137.2, 143.7, 203.5 ppm. HRMS (ESI+) calculated for $C_{27}H_{45}ClO_3Si_4Na$ ($[M+Na]^+$) : 587.2026, found : 587.2005.

(6c): (2*S*,3*R*)/(2*R*,3*S*)-2-triethylsilyloxy-3-tris(trimethylsilyl)siloxy-5-(3-chlorophenyl) pentanal :

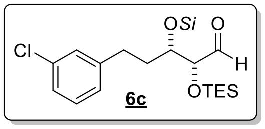

(37%, 94/6 *syn/anti*); colorless oil; TLC (hexane:CH_2Cl_2, 80:20) R_f = 0.55; ^{1}H NMR (400 MHz, $CDCl_3$): δ = 0.19 (s, 27H), 0.62 (qd, *J* = 2.4, 7.7 Hz, 6H), 0.95 (t, *J* = 7.9 Hz, 9H), 1.58-1.67 (m, 1H), 1.93-2.02 (m, 1H), 2.50-2.66 (m, 2H), 3.73 (ddd, *J* = 4.4, 6.0, 10.8 Hz, 1H), 4.17 (d, *J* = 4.4 Hz, 1H), 7.00-7.02 (m, 1H), 7.12-7.20 (m, 3H), 9.76 (d, *J* = 0.5 Hz, 1H) ppm. ^{13}C NMR (100 MHz, $CDCl_3$): δ = 0.5, 5.1, 6.9, 31.9, 35.0, 78.4, 79.5, 126.2, 126.5, 128.5, 129.8, 134.2, 144.0, 203.3 ppm. HRMS (ESI+) calculated for $C_{26}H_{53}O_3Si_5ClNa$ ($[M+Na]^+$) : 611.2422, found : 611.2417.

(7a): (2*S*,3*R*)/(2*R*,3*S*)-2,3-bis(tris(trimethylsilyl)siloxy)-hept-6-enal :

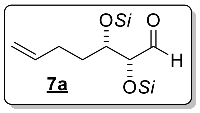

(58%, 98/2 *syn/anti*); colorless oil; TLC (hexane:CH_2Cl_2, 90:10) R_f = 0.24; ^{1}H NMR (400 MHz, $CDCl_3$): δ = 0.16 (s, 27H), 0.17 (s, 27H), 1.43-1.52 (m, 1H), 1.65-1.74 (m, 1H), 1.89-2.08 (m, 2H), 3.72 (dt, *J* = 4.8, 7.1 Hz, 1H), 3.81 (d, *J* = 4.8 Hz, 1H), 4.92-5.00 (m, 2H), 5.70-5.81 (m, 1H), 9.73 (s, 1H) ppm. ^{13}C NMR (100 MHz, $CDCl_3$): δ = 0.3, 0.4, 29.8, 32.5, 78.2, 82.5, 114.9, 137.9, 204.5 ppm. HRMS (ESI+) calculated for $C_{25}H_{64}O_3Si_8Na$ ($[M+Na]^+$) : 659.2902, found : 659.2896.

(7c): (2*S*,3*R*)/(2*R*,3*S*)-2-triethylsilyloxy-3-tris(trimethylsilyl)siloxy- hept-6-enal :

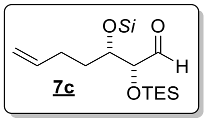

(58%, 97/3 *syn/anti*); colorless oil; TLC (hexane:CH_2Cl_2, 90:10) R_f = 0.19; ^{1}H NMR (400 MHz, $CDCl_3$): δ = 0.19 (s, 27H), 0.59 (qd, *J* = 2.1, 7.6 Hz, 6H), 0.93 (t, *J* = 8.0 Hz, 9H), 1.40-1.49 (m, 1H), 1.73-1.83 (m, 1H), 2.00-2.05 (m, 2H), 3.68 (dt, *J* = 4.4, 6.4 Hz, 1H), 4.07 (dd, *J* = 0.9, 4.6 Hz, 1H), 4.92-5.01 (m, 2H), 5.71-5.82 (m, 1H), 9.71 (d, *J* = 0.7 Hz, 1H) ppm. ^{13}C NMR (100 MHz, $CDCl_3$): δ = 0.5, 5.1, 6.8, 29.9, 32.3, 78.3, 79.3, 114.8, 137.9, 203.9 ppm. HRMS (ESI+) calculated for $C_{22}H_{52}O_3Si_5Na$ ($[M+Na]^+$) : 527.2654, found : 527.2647.

(8a): (2*S*,3*R*)/(2*R*,3*S*)-2,3-bis(tris(trimethylsilyl)siloxy)-4-phenylbutanal :

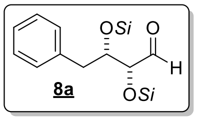

(45%, 93/7 *syn/anti*); colorless oil; TLC (hexane:CH_2Cl_2, 90:10) R_f = 0.25; ^{1}H NMR (400 MHz, $CDCl_3$): δ = 0.16 (s, 27H), 0.18 (s, 27H), 2.60 (dd, *J* = 5.7, 13.7 Hz, 1H), 2.96 (dd, *J* = 5.5, 13.7 Hz, 1H), 3.81 (d, *J* = 4.8 Hz, 1H), 3.98 (dd, *J* = 5.5, 10.6 Hz, 1H), 7.14-7.25 (m, 5H), 9.53 (s, 1H) ppm. ^{13}C NMR (100 MHz, $CDCl_3$): δ = 0.5, 0.6, 38.8, 79.8, 81.6, 126.6, 128.3, 130.2, 138.0, 203.3 ppm. HRMS (ESI+) calculated for $C_{28}H_{64}O_3Si_8Na$ ([M+Na]$^+$) : 695.2902, found : 695.2896.

(8b): (2*S*,3*R*)/(2*R*,3*S*)-2-benzyloxy-3-tris(trimethylsilyl)siloxy-4-phenylbutanal :

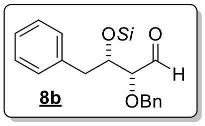

(80%, >99/1 *syn/anti*); colorless oil; TLC (hexane:CH_2Cl_2, 80:20) R_f = 0.35; ^{1}H NMR (400 MHz, $CDCl_3$): δ = 0.17 (s, 27H), 2.78 (dd, *J* = 6.0, 13.3 Hz, 1H), 3.05 (dd, *J* = 7.8, 13.3 Hz, 1H), 3.59 (dd, *J* = 1.0, 3.8 Hz, 1H), 4.07 (ddd, *J* = 1.6, 3.9, 6.0 Hz, 1H), 4.47 (d, *J* = 11.7 Hz, 1H), 4.66 (d, *J* = 11.7 Hz, 1H), 7.14-7.16 (m, 2H), 7.20-7.27 (m, 3H), 7.28-7.37 (m, 5H), 9.62 (d, *J* = 1.1 Hz, 1H) ppm. ^{13}C NMR (100 MHz, $CDCl_3$): δ = 0.6, 39.4, 72.7, 78.9, 83.8, 126.7, 128.0, 128.1, 128.5, 128.6, 129.8, 137.6, 137.7, 204.3 ppm. HRMS (ESI+) calculated for $C_{26}H_{44}O_3Si_4Na$ ([M+Na]$^+$) : 539.2260, found : 539.2264.

(9a): (2*S*,3*R*)/(2*R*,3*S*)-2,3-bis(tris(trimethylsilyl)siloxy)-5-(trimethylsilyl)pent-4-ynal:

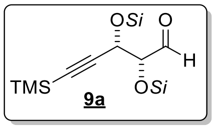

(20%, 68/32 *syn/anti*); colorless oil; TLC (hexane:CH_2Cl_2, 80:20) R_f = 0.55; Major : ^{1}H NMR (400 MHz, $CDCl_3$): δ = 0.12 (s, 9H), 0.17 (s, 27H), 0.18 (s, 27H), 3.78 (dd, *J* = 1.4, 3.4 Hz, 1H), 4.19 (d, *J* = 3.4 Hz, 1H), 9.54 (d, *J* = 1.4 Hz, 1H) ppm. ^{13}C NMR (100 MHz, $CDCl_3$): δ = 0.3, 0.4, 0.5, 70.8, 85.9, 92.7, 104.3, 200.3 ppm. Minor : ^{1}H NMR (400 MHz, $CDCl_3$): δ = 0.15 (s, 9H), 0.17 (s, 27H), 0.19 (s, 27H), 3.60 (dd, *J* = 1.8, 4.8 Hz, 1H), 4.25 (d, *J* = 4.8 Hz, 1H), 9.61 (d, *J* = 1.8 Hz, 1H) ppm. ^{13}C NMR (100 MHz, $CDCl_3$): δ = -0.1, 0.3, 0.4, 68.9, 82.0, 92.1, 103.9, 201.6 ppm. HRMS (ESI+) calculated for $C_{26}H_{66}O_3Si_9Na$ ([M+Na]$^+$) : 701.2833, found : 701.2827.

(9b): (2*S*,3*R*)/(2*R*,3*S*)-2-benzyloxy-3-tris(trimethylsilyl)siloxy-5-(trimethylsilyl)pent-4-ynal:

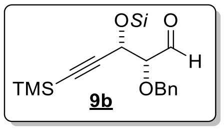

(36%, 57/43 *syn/anti*); colorless oil; TLC (hexane:AcOEt, 95:5) R_f = 0.55; Major : ^{1}H NMR (400 MHz, $CDCl_3$): δ = 0.16 (s, 9H), 0.18 (s, 27H), 3.84 (dd, *J* = 1.6, 4.6 Hz, 1H), 4.39 (d, *J* = 4.6 Hz, 1H), 4.68 (d, *J* = 12.4 Hz, 1H), 4.77 (d, *J* = 12.4 Hz, 1H), 7.24-7.34 (m, 5H), 9.66 (d, *J* = 1.6 Hz, 1H) ppm. ^{13}C NMR (100 MHz, $CDCl_3$): δ = -0.2, 0.4, 67.5, 72.9, 84.8, 93.4, 103.4, 128.0, 128.1, 128.5, 137.4, 200.7 ppm. Minor : ^{1}H NMR (400 MHz, $CDCl_3$): δ = 0.15 (s, 9H), 0.19 (s, 27H), 3.76 (dd, *J* = 1.8, 5.3 Hz, 1H), 4.34 (d, *J* = 5.3 Hz, 1H), 4.66 (d, *J* = 12.0 Hz, 1H), 4.70 (d, *J* = 12.0 Hz, 1H), 7.24-7.34 (m, 5H), 9.63 (d, *J* = 1.8 Hz, 1H) ppm. ^{13}C NMR (100 MHz, $CDCl_3$): δ = -0.2, 0.4, 68.3, 73.0, 85.9, 92.6, 103.7, 127.5, 128.1, 128.2, 128.5, 200.0 ppm. HRMS (ESI+) calculated for $C_{24}H_{46}O_3Si_5Na$ ([M+Na]$^+$) : 545.2185, found : 545.2186.

(10a): (2*S*,3*R*)/(2*R*,3*S*)-2,3-bis(tris(trimethylsilyl)siloxy)-4-methylpentanal :

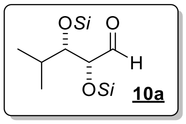

(51%, 39/61 *syn/anti*); Major : colorless oil; TLC (hexane:CH_2Cl_2, 90:10) R_f = 0.46; ^{1}H NMR (400 MHz, $CDCl_3$): δ = 0.16 (s, 27H), 0.17 (s, 27H), 0.90 (d, *J* = 7.1 Hz, 3H), 0.92 (d, *J* = 6.9 Hz, 3H), 1.84 (m, 1H), 3.52 (dd, *J* = 2.5, 3.9 Hz, 1H), 3.68 (dd, *J* = 2.0, 2.3 Hz, 1H), 9.45 (d, *J* = 1.8 Hz, 1H) ppm. ^{13}C NMR (100 MHz, $CDCl_3$): δ = 0.6, 0.7, 16.3, 19.1, 33.0, 82.4, 86.0, 201.7 ppm. Minor : colorless oil; TLC (hexane:CH_2Cl_2, 90:10) R_f = 0.56; ^{1}H NMR (400 MHz, $CDCl_3$): δ = 0.16 (s, 27H), 0.19 (s, 27H), 0.82 (d, *J* = 7.1 Hz, 3H), 0.86 (d, *J* = 7.1 Hz, 3H), 1.86 (m, 1H), 3.64 (dd, *J* = 3.2, 6.0 Hz, 1H), 3.90 (d, *J* = 6.2 Hz, 1H), 9.88 (s, 1H) ppm. ^{13}C NMR (100 MHz, $CDCl_3$): δ = 0.4, 0.7, 18.3, 19.3, 32.1, 82.8, 83.2, 203.9 ppm. HRMS (ESI+) calculated for $C_{24}H_{64}O_3Si_8Na$ ([M+Na]$^+$) : 647.2902, found : 647.2922.

(10b): (2*S*,3*R*)/(2*R*,3*S*)-2-benzyloxy-3-tris(trimethylsilyl)siloxy-4-methylpentanal :

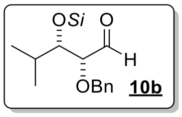

(72%, 96/4 *syn/anti*); colorless oil; TLC (hexane:CH_2Cl_2, 80:20) R_f = 0.45; ^{1}H NMR (400 MHz, $CDCl_3$): δ = 0.18 (s, 27H), 0.85 (d, *J* = 6.9 Hz, 3H), 0.96 (d, *J* = 7.1 Hz, 3H), 1.90-1.97 (m, 1H), 3.62 (dd, *J* = 4.0, 5.2 Hz, 1H), 3.88 (dd, *J* = 0.5, 5.2 Hz, 1H), 4.48 (d, *J* = 11.9 Hz, 1H), 4.77 (d, *J* = 11.9 Hz, 1H), 7.23-7.35 (m, 5H), 9.83 (d, *J* = 0.7 Hz, 1H) ppm. ^{13}C NMR (100 MHz, $CDCl_3$): δ = 0.7, 18.2, 19.6, 31.8, 72.7, 81.8, 85.1, 128.0, 128.0, 128.6, 137.6, 203.3 ppm. HRMS (ESI+) calculated for $C_{22}H_{44}O_3Si_4Na$ ([M+Na]$^+$) : 491.2260, found : 491.2262.

(10c): (2*S*,3*R*)/(2*R*,3*S*)-2-triethylsilyloxy-3-tris(trimethylsilyl)siloxy-4-methylpentanal :

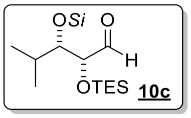

(69%, 97/3 *syn/anti*); colorless oil; TLC (hexane:CH_2Cl_2, 90:10) R_f = 0.21; ^{1}H NMR (400 MHz, $CDCl_3$): δ = 0.20 (s, 27H), 0.58 (qd, *J* = 3.6, 7.6 Hz, 6H), 0.81 (d, *J* = 6.9 Hz, 3H), 0.91-0.96 (m, 12H), 1.89-1.97 (m, 1H), 3.57 (dd, *J* = 3.9, 5.8 Hz, 1H), 4.15 (d, *J* = 5.5 Hz, 1H), 9.82 (s, 1H) ppm. ^{13}C NMR (100 MHz, $CDCl_3$): δ = 0.7, 5.1, 6.8, 18.2, 19.9, 31.4, 80.0, 83.1, 203.1 ppm. HRMS (ESI+) calculated for $C_{21}H_{52}O_3Si_5Na$ ([M+Na]$^+$) : 515.2655, found : 515.2643.

(11B): (2*S*,3*R*)/(2*R*,3*S*)-2-benzyloxy-3-tris(trimethylsilyl)siloxy-4-methylhexanal :

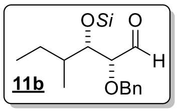

(50%, 81/19 *syn/anti*); colorless oil; TLC (hexane:CH_2Cl_2, 60:40) R_f = 0.52; <u>Major :</u> ^{1}H NMR (400 MHz, $CDCl_3$): δ = 0.16 (s, 27H), 0.80 (d, *J* = 6.9 Hz, 3H), 0.86 (t, *J* = 7.3 Hz, 3H), 1.16-1.21 (m, 1H), 1.47-1.52 (m, 1H), 1.62-1.67 (m, 1H), 3.69 (dd, *J* = 3.2, 5.5 Hz, 1H), 3.85 (dd, *J* = 0.9, 5.7 Hz, 1H), 4.49 (d, *J* = 11.9 Hz, 1H), 4.75 (d, *J* = 11.9 Hz, 1H), 7.26-7.32 (m, 5H), 9.82 (d, *J* = 0.7 Hz, 1H) ppm. ^{13}C NMR (100 MHz, $CDCl_3$): δ = 0.7, 12.3, 14.5, 26.5, 38.4, 72.7, 81.2, 85.1, 127.9, 128.0, 128.5, 137.6, 203.1 ppm. HRMS (ESI+) calculated for $C_{23}H_{46}O_3Si_4Na$ ([M+Na]$^+$) : 505.2422, found : 505.2418.

(11c): (2*S*,3*R*)/(2*R*,3*S*)-2-triethylsilyloxy-3-tris(trimethylsilyl)siloxy-4-methylhexanal :

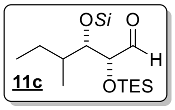

(46%, 79/21 *syn/anti*); colorless oil; TLC (hexane:CH_2Cl_2, 80:20) R_f = 0.50; <u>Major :</u> ^{1}H NMR (400 MHz, $CDCl_3$): δ = 0.20 (s, 27H), 0.55-0.63 (m, 6H), 0.77 (d, *J* = 4.8 Hz, 3H), 0.93 (t, *J* = 7.8 Hz, 9H), 1.09-1.12 (m, 3H), 1.40-1.48 (m, 2H), 1.61-1.69 (m, 1H), 3.65 (dd, *J* = 3.0, 5.8 Hz, 1H), 4.14 (d, *J* = 5.7 Hz, 1H), 9.83 (s, 1H) ppm. ^{13}C NMR (100 MHz, $CDCl_3$): δ = 0.7, 5.1, 6.8, 12.4, 14.7, 26.9, 37.8, 80.0, 82.4, 202.9 ppm. HRMS (ESI+) calculated for $C_{22}H_{54}O_3Si_5Na$ ([M+Na]$^+$) : 529.2811, found : 529.2788.

(12a): (2*S*,3*R*)/(2*R*,3*S*)-3-cyclohexyl-2,3-bis(tris(trimethylsilyl)siloxy)propanal

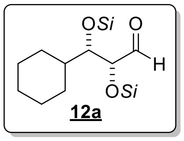

(56%, 71/29 *syn/anti*); colorless oil; TLC (hexane:CH_2Cl_2, 90:10) R_f = 0.40; <u>Major :</u> ^{1}H NMR (400 MHz, $CDCl_3$): δ = 0.17 (s, 27H), 0.17 (s, 27H), 0.98-1.28 (m, 5H), 1.50-1.57 (m, 2H), 1.68-1.77 (m, 4H), 3.49 (dd, *J* = 2.8,3.4 Hz, 1H), 3.72 (dd, *J* = 2.0, 2.4 Hz, 1H), 9.45 (d, *J* = 1.8 Hz, 1H) ppm. ^{13}C NMR (100 MHz, $CDCl_3$): δ = 0.6, 0.6, 26.3, 26.5, 26.8, 27.1, 29.9, 43.5, 83.0, 86.0, 201.7 ppm. HRMS (ESI+) calculated for $C_{27}H_{68}O_3Si_8Na$ ([M+Na]$^+$) : 687.3215, found : 687.3217.

(12b): (2*S*,3*R*)/(2*R*,3*S*)-3-cyclohexyl-2-benzyloxy-3-tris(trimethylsilyl)siloxy-propanal

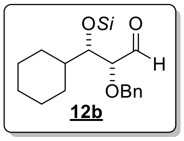

(76%, 98/2 *syn/anti*); colorless oil; TLC (hexane:AcOEt, 95:5) R_f = 0.46; ^{1}H NMR (400 MHz, $CDCl_3$): δ = 0.16 (s, 27H), 1.04-1.19 (m, 6H), 1.49-1.77 (m, 5H), 3.58 (dd, *J* = 4.2, 5.5 Hz, 1H), 3.84 (dd, *J* = 0.7, 5.5 Hz, 1H), 4.48 (d, *J* = 12.2 Hz, 1H), 4.76 (d, *J* = 12.2 Hz, 1H), 7.27-7.34 (m, 5H), 9.83 (d, *J* = 0.7 Hz, 1H) ppm. ^{13}C NMR (100 MHz, $CDCl_3$): δ = 0.7, 26.4, 26.6, 28.5, 29.7, 41.9, 72.7, 81.6, 84.9, 128.0, 128.5, 137.6, 203.2 ppm. HRMS (ESI+) calculated for $C_{25}H_{48}O_3Si_4Na$ ([M+Na]$^+$) : 531.2572, found : 531.2568.

(13a): (2*S*,3*R*)/(2*R*,3*S*)-2,3-bis(tris(trimethylsilyl)siloxy)-4-phenylpentanal :

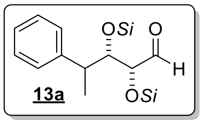

(30%, 34/66 *syn/anti*); colorless oil; TLC (hexane:CH_2Cl_2, 90:10) R_f = 0.43; Major : ^{1}H NMR (400 MHz, $CDCl_3$): δ = 0.15 (s, 27H), 0.18 (s, 27H), 1.22 (d, *J* = 7.3 Hz, 3H), 3.11-3.17 (m, 1H), 3.87 (d, *J* = 6.6 Hz, 1H), 3.96 (dd, *J* = 2.0, 6.4 Hz, 1H), 7.16-7.31 (m, 5H), 9.42 (s, 1H) ppm. ^{13}C NMR (100 MHz, $CDCl_3$): δ = 0.5, 0.8, 15.0, 41.1, 82.7, 83.5, 126.7, 128.0, 128.9, 143.4, 201.3 ppm. Minor : ^{1}H NMR (400 MHz, $CDCl_3$): δ = 0.02 (s, 27H), 0.23 (s, 27H), 1.32 (d, *J* = 7.3 Hz, 3H), 3.11-3.17 (m, 1H), 3.58 (d, *J* = 1.8 Hz, 1H), 3.98 (dd, *J* = 1.8, 4.8 Hz, 1H), 7.16-7.31 (m, 5H), 9.28 (d, *J* = 1.8 Hz, 1H) ppm. ^{13}C NMR (100 MHz, $CDCl_3$): δ = 0.9, 1.1, 12.4, 43.5, 85.1, 86.7, 128.3, 128.8, 130.2, 141.5, 202.5 ppm. HRMS (ESI+) calculated for $C_{29}H_{66}O_3Si_8Na$ ($[M+Na]^+$) : 709.3058, found : 709.3061.

(13b): (2*S*,3*R*)/(2*R*,3*S*)-2-benzyloxy-3-tris(trimethylsilyl)siloxy-4-phenylpentanal :

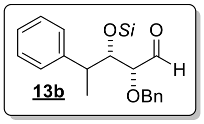

(71%, >99/1 *syn/anti*); colorless oil; TLC (hexane: AcOEt, 95:5) R_f = 0.52; ^{1}H NMR (400 MHz, $CDCl_3$): δ = 0.15 (s, 27H), 1.30 (d, *J* = 7.2 Hz, 3H), 3.12-3.19 (m, 1H), 3.80 (d, *J* = 5.6 Hz, 1H), 3.98 (dd, *J* = 4.4, 5.6 Hz, 1H), 4.43 (d, *J* = 12.0 Hz, 1H), 4.70 (d, *J* = 12.0 Hz, 1H), 7.18-7.34 (m, 10H), 9.61 (s, 1H) ppm. ^{13}C NMR (100 MHz, $CDCl_3$): δ = 0.8, 16.7, 42.4, 72.8, 82.1, 84.6, 126.7, 127.7, 127.9, 128.3, 128.5, 128.6, 137.7, 143.9, 202.2 ppm. HRMS (ESI+) calculated for $C_{27}H_{46}O_3Si_4Na$ ($[M+Na]^+$) : 553.2416, found : 553.2434.

(13c): (2*S*,3*R*)/(2*R*,3*S*)-2-triethylsilyloxy-3-tris(trimethylsilyl)siloxy-4-phenylpentanal :

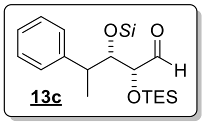

(59%, 86/14 *syn/anti*); colorless oil; TLC (hexane: CH_2Cl_2, 80:20) R_f = 0.40; ^{1}H NMR (400 MHz, $CDCl_3$): δ = 0.16 (s, 27H), 0.57 (qd, *J* = 2.0, 7.8 Hz, 6H), 0.92 (t, *J* = 7.8 Hz, 9H), 1.23 (d, *J* = 7.4 Hz, 3H), 3.12 (qd, *J* = 3.2, 7.1 Hz, 1H), 3.90 (dd, *J* = 3.5, 6.0 Hz, 1H), 4.12 (d, *J* = 6.0 Hz, 1H), 7.14-7.18 (m, 3H), 7.21-7.25 (m, 2H), 9.54 (s, 1H) ppm. ^{13}C NMR (100 MHz, $CDCl_3$): δ = 0.7, 5.1, 6.9, 16.2, 41.7, 79.6, 83.4, 126.5, 128.1, 128.6, 144.2, 201.5 ppm. HRMS (ESI+) calculated for $C_{26}H_{54}O_3Si_5Na$ ($[M+Na]^+$) : 577.2811, found : 577.2794.

(14b): (2*S*,3*R*)/(2*R*,3*S*)-2-benzyloxy-3-tris(trimethylsilyl)siloxy-4-methyl-5-(3,4-methylenedioxy phenyl)pentanal

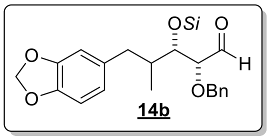

(57%, 98/2 *syn/anti*); colorless oil; TLC (hexane:AcOEt, 95:5) R_f = 0.34; ^{1}H NMR (400 MHz, $CDCl_3$): δ = 0.19 (s, 27H), 0.70 (d, *J* = 6.8 Hz, 3H), 2.00-2.07 (m, 1H), 2.27 (dd, *J* = 11.2, 13.3 Hz, 1H), 2.80 (dd, *J* = 3.6, 13.2 Hz, 1H), 3.77 (dd, *J* = 3.6, 5.6 Hz, 1H), 3.95 (d, *J* = 5.9 Hz, 1H), 4.50 (d, *J* = 12.0 Hz, 1H), 4.81 (d, *J* = 12.0 Hz, 1H), 5.90 (s, 2H), 6.55 (dd, *J* = 1.6, 8.0 Hz, 1H), 6.61 (d, *J* = 1.6 Hz, 1H), 6.70 (d, *J* = 7.6 Hz, 1H), 7.25-7.35 (m, 5H), 9.89 (s, 1H) ppm. ^{13}C NMR (100 MHz, $CDCl_3$): δ = 0.8, 14.2, 38.5, 40.0, 72.8, 81.3, 85.0, 100.8, 108.0, 109.6, 122.0, 128.0, 128.1, 128.6, 134.6, 137.5, 145.7, 147.5, 202.6 ppm. HRMS (ESI+) calculated for $C_{29}H_{48}O_5Si_4Na$ ($[M+Na]^+$) : 611.2471, found : 611.2471.

(14c): (2*S*,3*R*)/(2*R*,3*S*)-2-triethylsilyloxy-3-tris(trimethylsilyl)siloxy-4-methyl-5-(3,4-methylene dioxyphenyl)pentanal

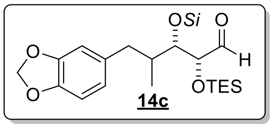

(41%, 93/7 *syn/anti*); colorless oil; TLC (hexane:CH_2Cl_2, 80:20) R_f = 0.29; ^{1}H NMR (400 MHz, $CDCl_3$): δ = 0.23 (s, 27H), 0.58-0.68 (m, 9H), 0.95 (t, *J* = 8.0 Hz, 9H), 2.05-2.10 (m, 1H), 2.27 (dd, *J* = 11.2, 13.2 Hz, 1H), 2.73 (dd, *J* = 3.9, 13.1 Hz, 1H), 3.74 (dd, *J* = 3.2, 6.2 Hz, 1H), 4.22 (d, *J* = 6.0 Hz, 1H), 5.90 (s, 2H), 6.54 (dd, *J* = 1.6, 7.8 Hz, 1H), 6.59 (d, *J* = 1.6 Hz, 1H), 6.69 (d, *J* = 8.0 Hz, 1H), 9.90 (s, 1H) ppm. ^{13}C NMR (100 MHz, $CDCl_3$): δ = 0.8, 5.1, 6.9, 14.0, 37.9, 40.6, 79.9, 82.6, 100.8, 108.1, 109.4, 121.9, 134.8, 145.7, 147.5, 202.5 ppm. HRMS (ESI+) calculated for $C_{28}H_{56}O_5Si_5Na$ ($[M+Na]^+$) : 635.2823, found : 635.2830.

(15b): (2*S*,3*R*)/(2*R*,3*S*)-2-benzyloxy-3-tris(trimethylsilyl)siloxy-4,4-dimethylpentanal :

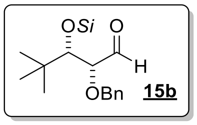

(81%, 98/2 *syn/anti*); colorless oil; TLC (hexane:CH_2Cl_2, 80:20) R_f = 0.42; ^{1}H NMR (400 MHz, $CDCl_3$): δ = 0.19 (s, 27H), 0.92 (s, 9H), 3.56 (d, *J* = 5.1 Hz, 1H), 4.00 (d, *J* = 5.1 Hz, 1H), 4.44 (d, *J* = 11.7 Hz, 1H), 4.80

(d, J = 11.7 Hz, 1H), 7.25-7.35 (m, 5H), 9.92 (s, 1H) ppm. ^{13}C NMR (100 MHz, $CDCl_3$): δ = 0.9, 27.4, 36.9, 72.9, 85.0, 86.7, 127.5, 127.8, 128.5, 137.9, 202.7 ppm. HRMS (ESI+) calculated for $C_{23}H_{46}O_3Si_4Na$ ($[M+Na]^+$) : 505.2416, found : 505.2417.

(16a): (2*S*,3*R*)/(2*R*,3*S*)-2,3-bis(tris(trimethylsilyl)siloxy)-3-phenylpropanal :

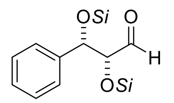

16a

(78%, 65/35 *syn/anti*); colorless oil; TLC (hexane:CH_2Cl_2, 90:10) R_f = 0.32; Major : ^{1}H NMR (400 MHz, $CDCl_3$): δ = 0.07 (s, 27H), 0.08 (s, 27H), 3.83 (dd, J = 1.4, 4.6 Hz, 1H), 4.61 (d, J = 4.4 Hz, 1H), 7.18-7.30 (m, 5H), 9.65 (d, J = 1.4 Hz, 1H) ppm. ^{13}C NMR (100 MHz, $CDCl_3$): δ = 0.3, 0.4, 80.7, 83.7, 127.4, 127.8, 128.4, 140.0, 204.1 ppm. Minor : ^{1}H NMR (400 MHz, $CDCl_3$): δ = 0.03 (s, 27H), 0.04 (s, 27H), 3.59 (dd, J = 3.0, 6.4 Hz, 1H), 4.37 (d, J = 6.2 Hz, 1H), 7.18-7.30 (m, 5H), 9.58 (d, J = 3.0 Hz, 1H) ppm. ^{13}C NMR (100 MHz, $CDCl_3$): δ = 0.3, 0.5, 81.0, 86.1, 127.7, 127.9, 128.3, 141.5, 201.7 ppm. HRMS (ESI+) calculated for $C_{27}H_{62}O_3Si_8Na$ ($[M+Na]^+$) : 681.2745, found : 681.2734.

(16d): (2*S*,3*R*)/(2*R*,3*S*)-2-allyloxy-3-tris(trimethylsilyl)siloxy-3-phenylpropanal

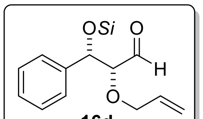

16d

(68%, 90/10 *syn/anti*); colorless oil; TLC (hexane:CH_2Cl_2, 90:10) R_f = 0.28; ^{1}H NMR (400 MHz, $CDCl_3$): δ = 0.09 (s, 27H), 3.82 (dd, J = 2.1, 5.5 Hz, 1H), 3.88-3.95 (m, 1H), 4.02-4.07 (m, 1H), 4.65 (d, J = 5.3 Hz, 1H), 5.10-5.21 (m, 2H), 5.73-5.82 (m, 1H), 7.23-7.34 (m, 5H), 9.53 (d, J = 1.8 Hz, 1H) ppm. ^{13}C NMR (100 MHz, $CDCl_3$): δ = 0.4, 72.1, 79.2, 86.7, 117.9, 127.2, 128.2, 128.7, 134.1, 139.7, 202.1 ppm. HRMS (ESI+) calculated for $C_{21}H_{40}O_3Si_4Na$ ($[M+Na]^+$) : 475.1946, found : 475.1928.

(17b): (2*S*,3*R*)/(2*R*,3*S*)-2-benzyloxy-3-tris(trimethylsilyl)siloxy-3-(2-chlorophenyl)propanal

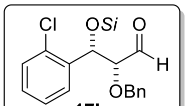

17b

(50%, 65/35 *syn/anti*); colorless oil; TLC (hexane:AcOEt, 95:5) R_f = 0.48; Major : ^{1}H NMR (400 MHz, $CDCl_3$): δ = 0.08 (s, 27H), 3.83 (dd, J = 1.8, 4.3 Hz, 1H), 4.33 (d, J = 12.4 Hz, 1H), 4.47 (d, J = 12.4 Hz, 1H), 5.26 (d, J = 4.4 Hz, 1H), 7.06-7.08 (m, 2H), 7.20-7.32 (m, 6H), 7.52 (dd, J = 2.0, 7.6 Hz, 1H), 9.68 (d, J = 1.8 Hz, 1H) ppm. ^{13}C NMR (100 MHz, $CDCl_3$): δ = 0.4, 73.1, 75.7, 85.5, 126.9, 127.9, 128.4, 128.4, 129.2, 129.2, 130.1, 132.0, 137.2, 137.7, 202.5 ppm. Minor : ^{1}H NMR (400 MHz, $CDCl_3$): δ = 0.10 (s, 27H), 3.76 (dd, J = 2.0, 2.5 Hz, 1H), 4.52 (d, J = 12.2 Hz, 1H), 4.70 (d, J = 12.2 Hz, 1H), 5.17 (d, J = 2.6 Hz, 1H), 7.18-7.31 (m, 8H), 7.38 (dd, J = 2.0, 7.6 Hz, 1H), 9.61 (d, J = 2.0 Hz, 1H) ppm. ^{13}C NMR (100 MHz, $CDCl_3$): δ = 0.3, 72.6, 77.8, 85.8, 126.9, 128.1, 128.4, 128.7, 129.2, 129.2, 129.4, 132.0, 137.1, 138.1, 201.5 ppm. HRMS (ESI+) calculated for $C_{25}H_{41}O_3Si_4ClNa$ ($[M+Na]^+$) : 559.1719, found : 559.1699.

(17d): (2*S*,3*R*)/(2*R*,3*S*)-2-allyloxy-3-tris(trimethylsilyl)siloxy-3-(2-chlorophenyl)propanal

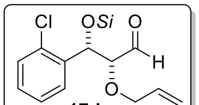

17d

(49%, 60/40 *syn/anti*); white solid; TLC (hexane:AcOEt, 95:5) R_f = 040; Major : ^{1}H NMR (400 MHz, $CDCl_3$): δ = 0.09 (s, 27H), 3.78 (dd, J = 1.8, 4.6 Hz, 1H), 3.80-3.92 (m, 2H), 5.06-5.10 (m, 2H), 5.23 (d, J = 4.8 Hz, 1H), 5.58-5.65 (m, 1H), 7.20-7.32 (m, 3H), 7.51 (dd, J = 2.0, 7.8 Hz, 1H), 9.66 (d, J = 2.0 Hz, 1H) ppm. ^{13}C NMR (100 MHz, $CDCl_3$): δ = 0.4, 72.5, 75.5, 86.3, 118.1, 127.0, 129.2, 129.3, 129.9, 132.0, 133.8, 137.8, 202.4 ppm. Minor : ^{1}H NMR (400 MHz, $CDCl_3$): δ = 0.11 (s, 27H), 3.75 (dd, J = 2.3, 5.1 Hz, 1H), 4.01-4.17 (m, 2H), 5.09-5.21 (m, 2H), 5.06 (d, J = 5.3 Hz, 1H), 5.80-5.87 (m, 1H), 7.18-7.31 (m, 3H), 7.38 (dd, J = 2.0, 7.6 Hz, 1H), 9.61 (d, J = 2.3 Hz, 1H) ppm. ^{13}C NMR (100 MHz, $CDCl_3$): δ = 0.3, 71.8, 77.9, 86.0, 118.3, 128.2, 129.3, 129.3, 129.4, 132.0, 134.0, 138.0, 201.8 ppm. HRMS (ESI+) calculated for $C_{21}H_{39}O_3Si_4ClNa$ ($[M+Na]^+$) : 509.1557, found : 509.1556.

(18a): (2*S*,3*R*)/(2*R*,3*S*)-2,3-bis(tris(trimethylsilyl)siloxy)-3-(3-methylphenyl)propanal

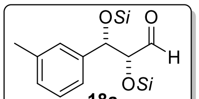

18a

(74%, 63/37 *syn/anti*); yellow solid; TLC (hexane:CH_2Cl_2, 90:10) R_f = 0.49; Major : ^{1}H NMR (400 MHz, $CDCl_3$): δ = 0.07 (s, 27H), 0.08 (s, 27H), 2.30 (s, 3H), 3.81 (dd, J = 1.4, 4.4 Hz, 1H), 4.58 (d, J = 4.2 Hz, 1H), 6.96-7.20 (m, 4H), 9.63 (d, J = 1.4 Hz, 1H) ppm. ^{13}C NMR (100 MHz, $CDCl_3$): δ = 0.2, 0.4, 21.5, 80.7, 83.8, 124.4, 127.7, 128.1, 128.6, 137.1, 139.8, 204.3 ppm. Minor : ^{1}H NMR (400 MHz, $CDCl_3$): δ = 0.03 (s, 27H), 0.04 (s, 27H), 2.31 (s, 3H), 3.57 (dd, J = 3.2, 6.6 Hz, 1H), 4.32 (d, J = 6.6 Hz, 1H), 6.96-7.20 (m, 4H), 9.56 (d, J = 3.0 Hz, 1H) ppm.

^{13}C NMR (100 MHz, $CDCl_3$): δ = 0.3, 0.4, 21.5, 80.9, 86.1, 124.9, 128.3, 128.4, 129.0, 137.8, 141.4, 201.7 ppm. HRMS (ESI+) calculated for $C_{28}H_{64}O_3Si_8Na$ ($[M+Na]^+$) : 695.2902, found : 695.2892.

(18c): (2*S*,3*R*)/(2*R*,3*S*)-2-triethylsilyloxy-3-tris(trimethylsilyl)siloxy-3-(3-methylphenyl) propanal

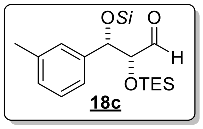

(36%, 57/43 *syn/anti*); colorless oil; TLC (hexane:CH_2Cl_2, 90:10) R_f = 0.35; Major : ^{1}H NMR (400 MHz, $CDCl_3$): δ = 0.08 (s, 27H), 0.45 (qd, *J* = 3.0, 7.6 Hz, 6H), 0.83 (t, *J* = 8.0 Hz, 9H), 2.30 (s, 3H), 4.01 (dd, *J* = 1.6, 4.1 Hz, 1H), 4.60 (d, *J* = 4.1 Hz, 1H), 7.00-7.18 (m, 4H), 9.58 (d, *J* = 1.6 Hz, 1H) ppm. ^{13}C NMR (100 MHz, $CDCl_3$): δ = 0.4, 4.8, 6.7, 21.5, 80.5, 81.0, 124.5, 127.7, 128.2, 128.7, 137.3, 139.9, 203.3 ppm. Minor : ^{1}H NMR (400 MHz, $CDCl_3$): δ = 0.06 (s, 27H), 0.39 (dq, *J* = 3.0, 7.5 Hz, 6H), 0.77 (t, *J* = 8.0 Hz, 9H), 2.32 (s, 3H), 3.84 (dd, *J* = 2.7, 6.0 Hz, 1H), 4.46 (d, *J* = 6.0 Hz, 1H), 7.00-7.18 (m, 4H), 9.60 (d, *J* = 2.5 Hz, 1H) ppm. ^{13}C NMR (100 MHz, $CDCl_3$): δ = 0.4, 4.8, 6.7, 21.5, 80.5, 81.0, 124.5, 127.7, 128.2, 128.7, 137.3, 139.9, 203.3 ppm. HRMS (ESI+) calculated for $C_{25}H_{52}O_3Si_5Na$ ($[M+Na]^+$) : 563.2660, found : 563.2657.

(18d): (2*S*,3*R*)/(2*R*,3*S*)-2-allyloxy-3-tris(trimethylsilyl)siloxy-3-(3-methylphenyl) propanal

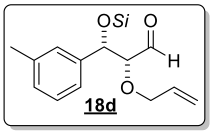

(56%, 93/7 *syn/anti*); colorless oil; TLC (hexane:CH_2Cl_2, 80:20) R_f = 0.48; ^{1}H NMR (400 MHz, $CDCl_3$): δ = 0.09 (s, 27H), 2.31 (s, 3H), 3.80 (dd, *J* = 1.8, 5.2 Hz, 1H), 3.92 (tdd, *J* = 1.6, 6.0, 13.1 Hz, 1H), 4.05 (tdd, *J* = 1.6, 5.6, 13.1 Hz, 1H), 4.60 (d, *J* = 5.6 Hz, 1H), 5.14 (ddd, *J* = 1.6, 3.2, 10.8 Hz, 1H), 5.20 (ddd, *J* = 1.6, 3.2, 17.2 Hz, 1H), 5.73-5.84 (m, 1H), 7.02-7.08 (m, 3H), 7.15-7.21 (m, 1H), 9.52 (d, *J* = 1.8 Hz, 1H) ppm. ^{13}C NMR (100 MHz, $CDCl_3$): δ = 0.4, 21.5, 72.1, 79.2, 86.9, 117.9, 124.4, 127.9, 128.1, 129.0, 134.2, 137.8, 139.7, 202.1 ppm. HRMS (ESI+) calculated for $C_{22}H_{42}O_3Si_4Na$ ($[M+Na]^+$) : 489.2103, found : 489.2107.

(19a): (2*S*,3*R*)/(2*R*,3*S*)-2,3-bis(tris(trimethylsilyl)siloxy)-3-(4-nitrophenyl)propanal

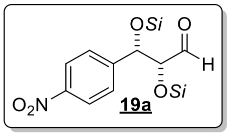

(63%, 53/47 *syn/anti*); yellow cristals; TLC (hexane:CH_2Cl_2, 90:10) R_f = 0.19; Major : ^{1}H NMR (400 MHz, $CDCl_3$): δ = 0.08 (s, 27H), 0.10 (s, 27H), 3.94 (dd, *J* = 1.1, 5.0 Hz, 1H), 4.71 (d, *J* = 5.0 Hz, 1H), 8.14 (d, *J* = 8.7 Hz, 2H), 8.19 (d, *J* = 8.7 Hz, 2H), 9.63 (d, *J* = 1.2 Hz, 1H) ppm. ^{13}C NMR (100 MHz, $CDCl_3$): δ = 0.3, 0.5, 80.1, 83.0, 123.0, 128.2, 147.5, 148.0, 202.6 ppm. Minor : ^{1}H NMR (400 MHz, $CDCl_3$): δ = 0.03 (s, 27H), 0.06 (s, 27H), 3.65 (dd, *J* = 2.5, 5.8 Hz, 1H), 4.52 (d, *J* = 5.8 Hz, 1H), 7.38 (d, *J* = 8.5 Hz, 2H), 7.41 (d, *J* = 8.7 Hz, 2H), 9.57 (d, *J* = 2.5 Hz, 1H) ppm. ^{13}C NMR (100 MHz, $CDCl_3$): δ = 0.3, 0.4, 80.4, 85.7, 123.4, 128.4, 147.7, 149.0, 201.7 ppm. HRMS (ESI+) calculated for $C_{27}H_{61}NO_5Si_8Na$ ($[M+Na]^+$) : 726.2596, found : 726.259.

(20a): (2*S*,3*R*)/(2*R*,3*S*)-2,3-bis(tris(trimethylsilyl)siloxy)3-(naphthalen-2-yl)propanal

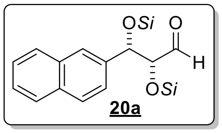

(55%, 89/11 *syn/anti*); colorless oil; TLC (hexane:CH_2Cl_2, 90:10) R_f = 0.47; Major : ^{1}H NMR (400 MHz, $CDCl_3$): δ = 0.05 (s, 27H), 0.09 (s, 27H), 3.92 (dd, *J* = 1.4, 4.3 Hz, 1H), 4.80 (d, *J* = 4.4 Hz, 1H), 7.32-7.42 (m, 3H), 7.70-7.80 (m, 4H), 9.67 (d, *J* = 1.4 Hz, 1H) ppm. ^{13}C NMR (100 MHz, $CDCl_3$): δ = 0.2, 0.4, 80.6, 83.8, 125.9, 126.1, 127.1, 127.7, 128.1, 132.9, 133.3, 137.6, 203.9 ppm. Minor : ^{1}H NMR (400 MHz, $CDCl_3$): δ = -0.01 (s, 27H), 0.04 (s, 27H), 3.70 (dd, *J* = 2.8, 6.2 Hz, 1H), 4.56 (d, *J* = 6.2 Hz, 1H), 7.30-7.47 (m, 3H), 7.70-7.80 (m, 4H), 9.63 (d, *J* = 2.9 Hz, 1H). ^{13}C NMR (100 MHz, $CDCl_3$): δ = 0.5 (2C), 81.3, 86.1, 125.5, 125.7, 126.1, 126.8, 128.3, 133.1, 133.5, 138.5, 201.7 ppm. HRMS (ESI+) calculated for $C_{31}H_{64}O_3Si_8Na$ ($[M+Na]^+$) : 731.2902, found : 731.2905.

(20b): (2*S*,3*R*)/(2*R*,3*S*)-2-benzyloxy-3-tris(trimethylsilyl)siloxy-3-(naphthalen-2-yl) propanal

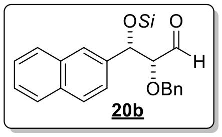

(60%, 98/2 *syn/anti*); colorless oil; TLC (hexane:AcOEt, 95:5) R_f = 0.51; ^{1}H NMR (400 MHz, $CDCl_3$): δ = 0.07 (s, 27H), 3.97 (dd, *J* = 1.8, 5.0 Hz, 1H), 4.46 (d, *J* = 12.3 Hz, 1H), 4.66 (d, *J* = 12.2 Hz, 1H), 4.90 (d, *J* = 5.0 Hz, 1H), 7.18-7.20 (m, 2H), 7.23-7.26 (m, 3H), 7.41-7.49 (m, 3H), 7.77-7.86 (m, 4H), 9.65 (d, *J* = 1.8 Hz, 1H) ppm. ^{13}C NMR (100 MHz, $CDCl_3$): δ = 0.5, 72.8, 79.4, 86.3, 125.3, 126.1, 126.2, 126.2, 127.9,

128.0, 128.2, 128.5, 133.1, 133.4, 137.4, 137.5, 202.3 ppm. HRMS (ESI+) calculated for $C_{29}H_{44}O_3Si_4Na$ ($[M+Na]^+$) : 575.2260, found : 575.2252.

(21a): (2*S*,3*R*)/(2*R*,3*S*)-2,3-bis(tris(trimethylsilyl)siloxy)-3-pentafluorophenylpropanal :

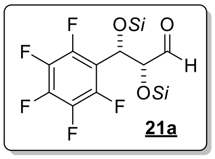

(68%, 82/18 *syn/anti*); white solid; TLC (hexane:CH_2Cl_2, 90:10) R_f = 0.30; Major : ^{1}H NMR (400 MHz, $CDCl_3$): δ = 0.05 (s, 27H), 0.08 (s, 27H), 3.95 (dd, *J* = 2.7, 8.5 Hz, 1H), 4.80 (d, *J* = 8.5 Hz, 1H), 9.65 (d, *J* = 2.6 Hz, 1H) ppm. ^{13}C NMR (100 MHz, $CDCl_3$): δ = 0.04, 0.18, 71.8, 82.6, 200.4 ppm. ^{19}F NMR (376 MHz, $CDCl_3$): δ = -140.1 (br. s, 2F), -154.2 (t, *J* = 22.9 Hz, 1F), -162.1 (br. s, 2F); Minor : ^{1}H NMR (400 MHz, $CDCl_3$): δ = 0.09 (s, 27H), 0.13 (s, 27H), 4.10 (d, *J* = 6.4 Hz, 1H), 5.14 (d, *J* = 6.4 Hz, 1H), 9.79 (d, *J* = 3.0 Hz, 1H) ppm. ^{13}C NMR (100 MHz, $CDCl_3$): δ = 0.04, 0.18, 73.8, 82.3, 201.0 ppm. ^{19}F NMR (376 MHz, $CDCl_3$): δ = -140.1 (br. s, 2F), -154.2 (t, *J* = 22.9 Hz, 1F), -162.1 (br. s, 2F); HRMS (ESI+) calculated for $C_{27}H_{57}O_3Si_8F_5Na$ ($[M+Na]^+$) : 771.2274, found : 771.2259.

(21c): (2*S*,3*R*)/(2*R*,3*S*)-2-triethylsilyloxy-3-tris(trimethylsilyl)siloxy-3-pentafluorophenylpropanal :

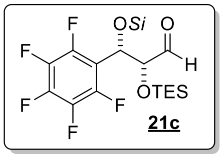

(44%, 95/5 *syn/anti*); colorless oil; TLC (hexane:AcOEt, 95:5) R_f = 0.35; ^{1}H NMR (400 MHz, $CDCl_3$): δ = 0.09 (s, 27H), 0.33-0.47 (m, 6H), 0.78 (t, *J* = 7.8 Hz, 9H), 4.14 (dd, *J* = 2.5, 7.8 Hz, 1H), 4.93 (d, *J* = 7.6 Hz, 1H), 9.65 (d, *J* = 2.7 Hz, 1H) ppm. ^{13}C NMR (100 MHz, $CDCl_3$): δ = 0.2, 4.5, 6.3, 72.1, 79.5, 199.9 ppm. ^{19}F NMR (376 MHz, $CDCl_3$): δ = -140.2 (d, *J* = 51.9 Hz, 2F), -153.9 (t, *J* = 23.1 Hz, 1F), -162.2 to -162.0 (m, 2F); HRMS (ESI+) calculated for $C_{24}H_{45}O_3Si_5F_5Na$ ($[M+Na]^+$) : 639.2033, found : 639.2028.

(22a): (2*S*,3*R*)/(2*R*,3*S*)- 2,3-bis(tris(trimethylsilyl)siloxy)-3-(4-bromothiophen-2-yl) propanal

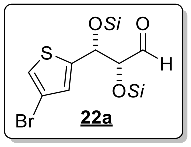

(61%, 81/19 *syn/anti*); yellow solid; TLC (hexane:CH_2Cl_2, 90:10) R_f = 0.29; Major : ^{1}H NMR (400 MHz, $CDCl_3$): δ = 0.13 (s, 27H), 0.14 (s, 27H), 3.86 (dd, *J* = 1.2, 5.0 Hz, 1H), 4.75 (dd, *J* = 0.9, 5.0 Hz, 1H),6.79 (dd, *J* = 0.9, 1.4 Hz, 1H), 7.08 (d, *J* = 1.4 Hz, 1H), 9.58 (d, *J* = 1.2 Hz, 1H) ppm. ^{13}C NMR (100 MHz, $CDCl_3$): δ = 0.3, 0.4, 82.3, 85.8, 109.1, 122.3, 127.6, 144.7, 202.3 ppm. Minor : ^{1}H NMR (400 MHz, $CDCl_3$): δ = 0.09 (s, 27H), 0.09 (s, 27H), 3.64 (dd, *J* = 2.7, 5.9 Hz, 1H), 4.56 (d, *J* = 6.0 Hz, 1H), 6.79 (dd, *J* = 1.6 Hz, 1H), 7.15 (d, *J* = 1.6 Hz, 1H), 9.55 (d, *J* = 2.5 Hz, 1H) ppm. ^{13}C NMR (100 MHz, $CDCl_3$): δ = 0.4, 0.5, 77.6, 97.9, 109.2, 122.6, 128.1, 146.9, 201.4 ppm. HRMS (ESI+) calculated for $C_{25}H_{59}BrO_3SSi_8Na$ ($[M+Na]^+$) : 765.1420, found : 765.1409.

(22b): (2*S*,3*R*)/(2*R*,3*S*)-2-benzyloxy-3-tris(trimethylsilyl)siloxy-3-(4-bromothiophen-2-yl) propanal

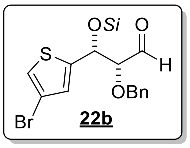

(49%, 83/17 *syn/anti*); yellow solid; TLC (hexane:AcOEt, 95:5) R_f = 0.23; Major : ^{1}H NMR (400 MHz, $CDCl_3$): δ = 0.10 (s, 27H), 3.82 (dd, *J* = 1.8, 5.0 Hz, 1H), 4.50 (d, *J* = 12.1 Hz, 1H), 4.72 (d, *J* = 12.1 Hz, 1H), 4.82 (dd, *J* = 0.9, 5.0 Hz, 1H), 6.89 (dd, *J* = 0.9, 1.4 Hz, 1H), 7.13 (d, *J* = 1.6 Hz, 1H), 7.28-7.33 (m, 5H), 9.58 (d, *J* = 1.8 Hz, 1H) ppm. ^{13}C NMR (100 MHz, $CDCl_3$): δ = 0.3, 72.9, 75.3, 84.6, 109.3, 122.7, 127.7, 128.1, 128.2, 128.6, 137.1, 144.9, 201.1 ppm. HRMS (ESI+) calculated for $C_{23}H_{39}O_3BrSSi_4Na$ ($[M+Na]^+$) : 609.0778, found : 609.0777.

(23a): (2*S*,3*R*)/(2*R*,3*S*)-2,3-bis(tris(trimethylsilyl)siloxy)-3-(5-nitrofuran-2-yl) propanal

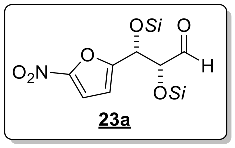

(44%, 58/42 *syn/anti*); yellow oil; TLC (hexane:AcOEt, 80:20) R_f = 0.33; Major : ^{1}H NMR (400 MHz, $CDCl_3$): δ = 0.11 (s, 27H), 0.13 (s, 27 H), 3.96 (dd, *J* = 1.4, 5.5 Hz, 1H), 4.70 (d, *J* = 5.5 Hz, 1H), 6.36 (dd, *J* = 0.5, 3.7 Hz, 1H), 7.23-7.35 (m, 1H), 9.72 (d, *J* = 1.4 Hz, 1H) ppm. ^{13}C NMR (100 MHz, $CDCl_3$): δ = 0.2, 0.3, 74.8, 81.8, 111.4, 112.0, 128.3, 157.5, 201.3 ppm. Minor : ^{1}H NMR (400 MHz, $CDCl_3$): δ = 0.10 (s, 27H), 0.12 (s, 27H), 3.86 (dd, *J* = 2.1, 4.8 Hz, 1H), 4.56 (d, *J* = 5.3 Hz, 1H), 6.46 (dd, *J* = 0.7, 3.7 Hz, 1H), 7.23-7.35 (m, 1H), 9.58 (d, *J* = 2.0 Hz, 1H) ppm. ^{13}C NMR (100 MHz, $CDCl_3$): δ = 0.4, 0.5, 75.9, 84.1, 111.6, 112.1, 128.5, 157.7, 201.1 ppm. HRMS (ESI+) calculated for $C_{25}H_{59}O_6NSi_8Na$ ($[M+Na]^+$) : 716.2394, found : 716.2380.

(24a): (2*S*,3*R*,4*R*)/(2*R*,3*S*,4*R*)-2,3-bis(tris(trimethylsilyl)siloxy)-4-phenylpentanal :

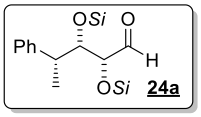

(32%, 65/35 *syn/anti*); white solid; TLC (hexane:CH_2Cl_2, 90:10) R_f = 0.51; Major : ^{1}H NMR (400 MHz, $CDCl_3$): δ = 0.16 (s, 27H), 0.19 (s, 27H), 1.23 (d, *J* = 7.6 Hz, 3H), 3.10-3.17 (m, 1H), 3.87 (d, *J* = 6.5 Hz, 1H), 3.96 (dd, *J* = 1.8, 6.4 Hz, 1H), 7.17-7.30 (m, 5H), 9.43 (s, 1H) ppm. ^{13}C NMR (100 MHz, $CDCl_3$): δ = 0.5, 0.8, 15.0, 41.2, 77.4, 83.5, 126.7, 128.1, 128.9, 143.4, 201.3 ppm. Minor : ^{1}H NMR (400 MHz, $CDCl_3$): δ = 0.02 (s, 27H), 0.24 (s, 27H), 1.32 (d, *J* = 7.4 Hz, 3H), 3.11-3.17 (m, 1H), 3.59 (dd, *J* = 0.7, 1.8 Hz, 1H), 3.98 (d, *J* = 4.8 Hz, 1H), 7.18-7.31 (m, 5H), 9.29 (d, *J* = 1.8 Hz, 1H) ppm. ^{13}C NMR (100 MHz, $CDCl_3$): δ = 0.5, 0.9, 12.4, 43.5, 77.4, 86.7, 127.0, 128.3, 128.9, 141.5, 202.5 ppm. HRMS (ESI+) calculated for $C_{29}H_{66}O_3Si_8Na$ ([M+Na]$^+$) : 709.3058, found : 709.3050. $[\alpha]_D^{26}$ -6.80 (c 1.47, $CHCl_3$).

(24b): (2*S*,3*R*,4*R*)/(2*R*,3*S*,4*R*)-2-benzyloxy-3-tris(trimethylsilyl)siloxy-4-phenylpentanal :

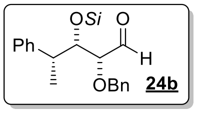

(53%, 98/2 *syn/anti*); colorless oil; TLC (hexane:AcOEt, 95:5) R_f = 0.42; ^{1}H NMR (400 MHz, $CDCl_3$): δ = 0.14 (s, 27H), 1.28 (d, *J* = 7.2 Hz, 3H), 3.15 (dq, *J* = 4.4, 7.4 Hz, 1H), 3.79 (d, *J* = 5.3 Hz, 1H), 3.97 (dd, *J* = 4.6, 5.3 Hz, 1H), 4.42 (d, *J* = 12.1 Hz, 1H), 4.69 (d, *J* = 12.0 Hz, 1H), 7.18-7.31 (m, 10H), 9.60 (s, 1H) ppm. ^{13}C NMR (100 MHz, $CDCl_3$): δ = 0.7, 16.7, 42.4, 72.8, 82.1, 84.6, 126.6, 127.7, 127.9, 128.3, 128.5, 128.6, 137.7, 143.8, 202.2 ppm. HRMS (ESI+) calculated for $C_{27}H_{46}O_3Si_4Na$ ([M+Na]$^+$) : 553.2416, found : 553.2415. $[\alpha]_D^{26}$ -15.70 (c 1.02, $CHCl_3$).

(24d): (2*S*,3*R*,4*R*)/(2*R*,3*S*,4*R*)-2-allyloxyoxy-3-tris(trimethylsilyl)siloxy-4-phenylpentanal :

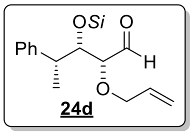

(62%, 98/2 *syn/anti*); colorless oil; TLC (hexane:AcOEt, 95:5) R_f = 0.42 ^{1}H NMR (400 MHz, $CDCl_3$): δ = 0.17 (s, 27H), 1.30 (d, *J* = 7.4 Hz, 3H), 3.10 (dq, *J* = 4.4, 7.4 Hz, 1H), 3.73 (d, *J* = 5.3 Hz, 1H), 3.91 (ddt, *J* = 1.6, 5.6, 12.8 Hz, 1H), 3.96 (dd, *J* = 4.2, 5.3 Hz, 1H), 4.10 (ddt, *J* = 1.4, 5.2, 12.8 Hz, 1H), 5.13-5.22 (m, 2H), 5.77-5.85 (m, 1H), 7.16-7.20 (m, 3H), 7.23-7.25 (m, 2H), 9.59 (s, 1H) ppm. ^{13}C NMR (100 MHz, $CDCl_3$): δ = 0.8, 16.5, 42.2, 71.9, 82.0, 84.7, 117.7, 126.6, 128.3, 128.5, 134.2, 143.9, 202.2 ppm. HRMS (ESI+) calculated for $C_{23}H_{44}O_3Si_4Na$ ([M+Na]$^+$) : 503.2260, found : 503.2252. $[\alpha]_D^{24}$ -46.00 (c 1, $CHCl_3$).

4. One-pot sequential reaction : synthesis of protected 1,2,3-triols

GP3. General procedure

The aldol reaction was done according to GP2, but instead of quenching the reaction, the following procedure was performed: Reaction was maintained at -40 °C and a solution of nucleophile (1.5 eq.) was added dropwise. After stirring for 1-2h at -40 °C reaction was quenched by the addition of saturated aqueous solution of sodium bicarbonate (2 mL). The reaction was allowed to warm to ambient temperature and stirred vigorously for 20 min. The mixture was diluted with 5 mL of dichloromethane and washed with water and brine. The organic layer was dried over sodium sulfate, filtered through cotton and concentrated under reduced pressure. The residue was then purified by flash chromatography on silica gel eluting with Hexane/ CH_2Cl_2 (20% to 50% gradient).

(25): (2*S*,3*S*,4*R*)/(2*R*,3*R*,4*S*)-3-benzyloxy-4-tris(trimethylsilyl)siloxy-6-phenylhexan-2-ol

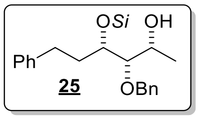

(84%, >99/1/0 *syn/anti*); colorless oil; TLC (hexane:AcOEt, 95:5) R_f = 0.27; ^{1}H NMR (400 MHz, $CDCl_3$): δ = 0.20 (s, 27H), 1.21 (d, *J* = 6.4 Hz, 3H), 1.73-1.83 (m, 1H), 1.98-2.05 (m, 1H), 2.49 (d, *J* = 4.6 Hz, 1H), 2.67 (dd, *J* = 8.5, 8.7 Hz, 1H), 3.28 (dd, *J* = 3.9, 5.7 Hz, 1H), 3.75 (dt, *J* = 3.9, 6.4 Hz, 1H), 3.96-4.00 (m, 1H), 4.62 (d, *J* = 11.3 Hz, 1H), 4.76 (d, *J* = 11.3 Hz, 1H), 7.14-7.18 (m, 3H), 7.23-7.27 (m, 3H), 7.29-7.36 (m, 4H) ppm. ^{13}C NMR (100 MHz, $CDCl_3$): δ = 0.7, 20.5, 32.7, 35.2, 66.5, 73.4, 76.0, 85.4, 125.9, 127.6, 127.9, 128.3, 128.5, 128.6, 138.4, 142.3 ppm. HRMS (ESI+) calculated for $C_{28}H_{50}O_3Si_4Na$ ([M+Na]$^+$) : 569.2729, found : 569.2737.

(26): (3*S*,4*S*,5*R*)/(3*R*,4*R*,5*S*)-4-benzyloxy-5-tris(trimethylsilyl)siloxy-2-methyl-7-phenylheptan-3-ol

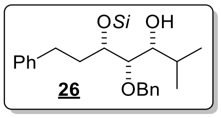

(81%, >99/1/0 *syn/anti*); colorless oil; TLC (hexane: AcOEt, 95:5) R_f = 0.29; ^{1}H NMR (400 MHz, $CDCl_3$): δ = 0.21 (s, 27H), 0.89 (d, *J* = 6.8 Hz, 3H), 0.96 (d, *J* = 6.8 Hz, 3H), 1.69-1.88 (m, 2H), 2.05-2.16 (m, 1H), 2.41 (d, *J* = 7.6 Hz, 1H), 2.62 (td, *J* = 5.2, 12.0 Hz, 1H), 2.81 (td, *J* = 4.8, 12.8 Hz, 1H), 3.51-3.55 (m, 1H), 3.77-3.81 (m, 1H), 4.64 (d, *J* = 11.5 Hz, 1H), 4.79 (d, *J* = 11.4 Hz, 1H), 7.15-7.22 (m, 2H), 7.24-7.36 (m, 8H) ppm. ^{13}C NMR (100 MHz, $CDCl_3$): δ = 0.8, 17.6, 19.9, 31.9, 32.1, 35.7, 73.1, 74.1, 75.8, 80.8, 125.8, 127.8, 127.9, 128.4, 128.5, 128.6, 138.4, 142.4 ppm. HRMS (ESI+) calculated for $C_{30}H_{54}O_3Si_4Na$ ($[M+Na]^+$) : 597.3042, found : 597.3044.

(27): (3*R*,4*S*,5*R*)/(3*S*,4*R*,5*S*)-4-benzyloxy-2,2-dimethyl-5-tris(trimethylsilyl)siloxy-6-phenylhex-1-en-3-ol

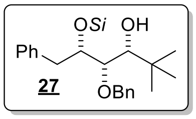

(78%, >99/1/0 *syn/anti*); colorless oil; TLC (hexane:AcOEt, 90:10) R_f = 0.64; ^{1}H NMR (400 MHz, $CDCl_3$): δ = 0.25 (s, 27H), 0.82 (s, 9H), 2.75 (d, *J* = 9.0 Hz, 1H), 2.90 (dd, *J* = 3.5, 14.0 Hz, 1H), 3.15 (dd, *J* = 10.3, 14.0 Hz, 1H), 3.47-3.49 (m, 2H), 4.09 (dt, *J* = 3.7, 10.3 Hz, 1H), 4.23 (d, *J* = 10.7 Hz, 1H), 4.35 (d, *J* = 10.6 Hz, 1H), 6.72-6.75 (m, 2H), 7.12-7.23 (m, 6H), 7.28-7.30 (m, 2H) ppm. ^{13}C NMR (100 MHz, $CDCl_3$): δ = 0.8, 26.4, 35.1, 39.6, 72.1, 75.0, 75.8, 78.6, 125.9, 127.5, 128.0, 128.4, 129.3, 137.6, 138.9 ppm. HRMS (ESI+) calculated for $C_{30}H_{54}O_3Si_4Na$ ($[M+Na]^+$) : 597.3042, found : 597.3041.

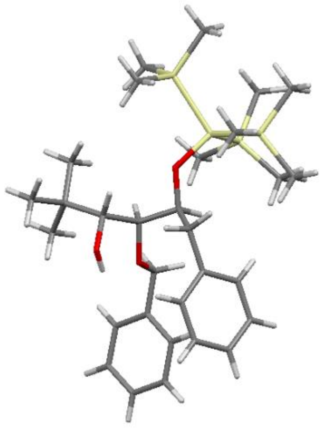

Single crystal X-ray crystallographic analysis for **27** (CCDC No. 1409678)

Bond precision:	C-C = 0.0087 A	Wavelength=0.71075	
Cell:	a=11.113(3)	b=35.197(9)	c=9.482(3)
	alpha=90	beta=102.084(7)	gamma=90
Temperature:	293 K		

	Calculated	Reported
Volume	3626.7(18)	3626.5(16)
Space group	P 21/c	P 1 21/c 1
Hall group	-P 2ybc	-P 2ybc
Moiety formula	C30 H54 O3 Si4	C30 H54 O3 Si4
Sum formula	C30 H54 O3 Si4	C30 H54 O3 Si4
Mr	575.09	575.10
Dx,g cm-3	1.053	1.580
Z	4	6
Mu (mm-1)	0.189	0.284
F000	1256.0	1884.0

F000'	1257.68	
h,k,lmax	14,45,12	14,45,12
Nref	8322	7577
Tmin,Tmax	0.945,0.945	0.587,0.945
Tmin'	0.945	

Correction method= # Reported T Limits: Tmin=0.587 Tmax=0.945
AbsCorr = MULTI-SCAN

Data completeness= 0.910 Theta(max)= 27.480

R(reflections)= 0.0847(2990) wR2(reflections)= 0.2172(7577)

S = 0.961 Npar= 334

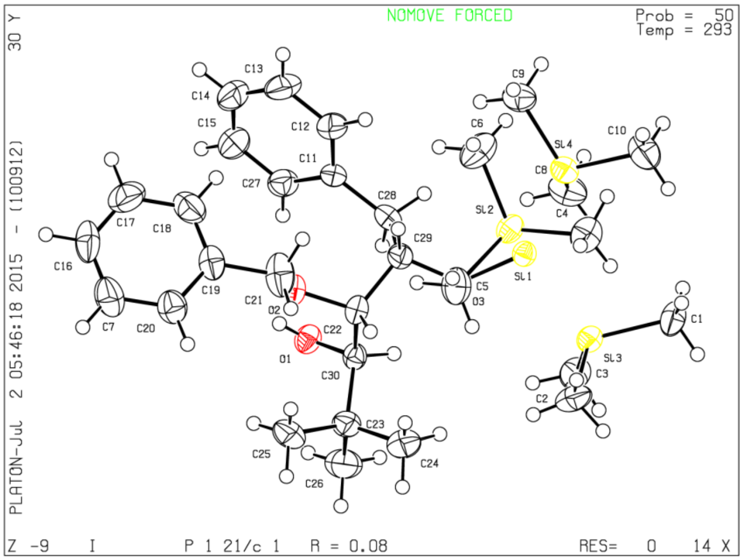

(28): (3*R*,4*S*,5*R*)/(3*S*,4*R*,5*S*)-2-benzyloxy-1,4-diphenyl-3-tris(trimethylsilyl)siloxybutanol

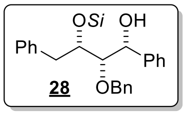

(59%, >99/1/0 *syn/anti*); colorless crystals (crystallized from hexane); TLC (hexane: AcOEt, 95:5) R_f = 0.49; ^{1}H NMR (400 MHz, $CDCl_3$): δ = 0.18 (s, 27H), 2.94 (dd, *J* = 4.4, 13.7 Hz, 1H), 3.10 (dd, *J* = 8.9, 14.0 Hz, 1H), 3.44 (dd, *J* = 0.9, 3.4 Hz, 1H), 3.98-4.01 (m, 1H), 4.22 (d, *J* = 11.5 Hz, 1H), 4.33 (d, *J* = 11.7 Hz, 1H), 4.9 (d, *J* = 0.9 Hz, 1H), 6.98-6.99 (m, 2H), 7.15-7.32 (m, 13H), ppm. ^{13}C NMR (100 MHz, $CDCl_3$): δ = 0.4, 39.9, 72.4, 73.3, 83.2, 126.2, 126.3, 127.2, 127.4, 127.4, 128.2, 128.3, 128.5, 129.5, 138.2, 138.3, 143.0, 147.2 ppm. HRMS (ESI+) calculated for $C_{32}H_{50}O_3Si_4Na$ ($[M+Na]^+$) : 617.2729, found : 617.2701.

(29): (3*R*,4*S*,5*R*)/(3*S*,4*R*,5*S*)-4-benzyloxy-5-tris(trimethylsilyl)siloxy-6-phenylhex-1-en-3-ol

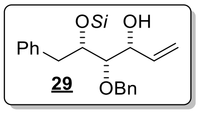

(68%, >99/1/0 *syn/anti*); colorless oil; TLC (hexane: AcOEt, 95:5) R_f = 0.34 ^{1}H NMR (400 MHz, $CDCl_3$): δ = 0.18 (s, 27H), 2.62 (d, *J* = 6.2 Hz, 1H), 2.87 (dd, *J* = 6.4, 13.8 Hz, 1H), 3.08 (dd, *J* = 3.9, 13.8 Hz, 1H), 3.30 (t, *J* = 3.9 Hz, 1H), 3.98 (td, *J* = 3.9, 6.5 Hz, 1H), 4.38-4.42 (m, 1H), 4.56 (d, *J* = 11.7 Hz, 1H), 4.61 (d, *J* = 11.7 Hz, 1H), 5.13 (dt, *J* = 1.6, 10.6 Hz, 1H), 5.34 (dt, *J* = 1.6, 17.2 Hz, 1H), 5.83-5.91 (m, 1H), 7.18-7.38 (m, 10H) ppm. ^{13}C NMR (100 MHz, $CDCl_3$): δ = 0.8, 39.8, 71.0, 73.1, 77.9, 82.1, 115.4, 126.3, 127.5, 127.7, 128.4, 128.5, 129.7, 138.4, 138.9, 140.0 ppm. HRMS (ESI+) calculated for $C_{28}H_{48}O_3Si_4Na$ ($[M+Na]^+$) : 567.2573, found : 567.2578.

(30): (3*R*,4*S*,5*R*)/(3*S*,4*R*,5*S*)-2-benzyloxy-4-isopropyl-1-(2-thienyl)-3-tris(trimethylsilyl)siloxybutanol

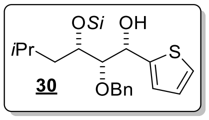

(75%, >99/1/0 *syn/anti*); colorless oil; TLC (hexane: AcOEt, 95:5) R_f = 0.35; ^{1}H NMR (400 MHz, $CDCl_3$): δ = 0.19 (s, 27H), 0.86-0.90 (m, 6H), 1.40-1.44 (m, 1H), 1.72-1.79 (m, 2H), 3.12 (d, *J* = 6.7 Hz, 1H), 3.68 (dd, *J* = 3.2, 3.4 Hz, 1H), 3.72-3.76 (m, 1H), 4.60 (s, 2H), 5.23 (dd, *J* = 3.2, 6.7 Hz, 1H), 6.96 (dd, *J* = 3.5, 5.0 Hz, 1H), 7.00-7.01 (m, 1H), 7.23 (dd, *J* = 1.1, 5.0 Hz, 1H), 7.26-7.34 (m, 5H) ppm. ^{13}C NMR (100 MHz, $CDCl_3$): δ = 0.7, 22.8, 23.9, 24.8, 42.7, 68.4, 73.5, 74.1, 84.0, 124.3, 124.6, 126.6, 127.7, 127.8, 128.5, 138.2, 147.5 ppm. HRMS (ESI+) calculated for $C_{27}H_{50}O_3SSi_4Na$ ($[M+Na]^+$) : 589.2450, found : 589.2461.

(31): (3*R*,4*S*,5*R*)/(3*S*,4*R*,5*S*)-4-allyloxy-5-tris(trimethylsilyl)siloxy-7-phenylhept-1-en-3-ol

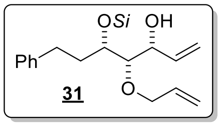

(82%, >99/1/0 *syn/anti*); colorless oil; TLC (hexane:AcOEt, 90:10) R_f = 0.47; ^{1}H NMR (400 MHz, $CDCl_3$): δ = 0.19 (s, 27H), 1.70-1.85 (m, 1H), 2.00-2.10 (m, 1H), 2.60 (d, *J* = 6.6 Hz, 1H), 2.64-2.69 (m, 1H), 3.32 (t, *J* = 4.1 Hz, 1H), 3.67 (dt, *J* = 4.4, 6.2 Hz, 1H), 4.09-4.20 (m, 2H), 4.30-4.33 (m, 1H), 5.13-5.18 (m, 2H), 5.25-5.37 (m, 2H), 5.84-5.97 (m, 2H), 7.15-7.18 (m, 3H), 7.25-7.28 (m, 2H) ppm. ^{13}C NMR (100 MHz, $CDCl_3$): δ = 0.7, 32.4, 35.3, 70.6, 72.6, 76.6, 82.8, 114.9, 117.1, 125.8, 128.4, 128.5, 134.8, 139.2, 142.4 ppm. HRMS (ESI+) calculated for $C_{25}H_{48}O_3Si_4Na$ ($[M+Na]^+$) : 531.2578, found : 531.2564.

(32): (3*R*,4*S*,5*R*)/(3*S*,4*R*,5*S*)-4-allyloxy-7-methyl-5-tris(trimethylsilyl)siloxy-oct-1-yn-3-ol

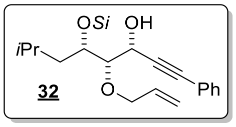

(52%, 88/12/0 *syn/anti*); colorless oil; TLC (hexane: AcOEt, 90:10) R_f = 0.23; ^{1}H NMR (400 MHz, $CDCl_3$): δ = 0.20 (s, 27H), 0.89 (d, *J* = 6.6 Hz, 3H), 0.91 (d, *J* = 6.6 Hz, 3H), 1.35-1.39 (m, 1H), 1.69-1.75 (m, 1H), 2.84 (d, *J* = 7.1 Hz, 1H), 3.59 (dd, *J* = 3.5, 3.6 Hz, 1H), 3.72 (dt, *J* = 3.7, 6.6 Hz, 1H), 4.21-4.26 (m, 2H), 4.36-4.41 (m, 1H), 4.78 (dd, *J* = 3.4, 7.1 Hz, 1H), 5.16-5.19 (m, 1H), 5.28-5.34 (m, 1H), 5.88-6.00 (m, 1H), 7.27-7.30 (m, 3H), 7.39-7.41 (m, 2H) ppm. ^{13}C NMR (100 MHz, $CDCl_3$): δ = 0.7, 23.1, 23.4, 24.7, 42.2, 61.1, 72.6, 75.0, 82.2, 85.0, 89.9, 117.0, 122.9, 128.3, 128.4, 131.7, 134.8 ppm. HRMS (ESI+) calculated for $C_{27}H_{50}O_3Si_4Na$ ($[M+Na]^+$) : 557.2729, found : 557.2714.

(33): (3*R*,4*S*,5*R*)/(3*S*,4*R*,5*S*)-2-benzyloxy-1,4-diphenyl-3-tris(trimethylsilyl)siloxypropanol

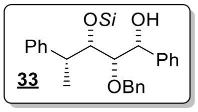

(50%, 83/17/0 *syn/anti*); colorless oil; TLC (hexane: AcOEt, 90:10) R_f = 0.48; ^{1}H NMR (400 MHz, $CDCl_3$): δ = 0.24 (s, 27H), 1.33 (d, *J* = 7.3 Hz, 3H), 3.05 (d, *J* = 8.7Hz, 1H), 3.33 (t, *J* = 7.1 Hz, 1H), 3.50 (dd, *J* = 2.1, 4.8 Hz, 1H), 3.94 (dd, *J* = 4.8, 7.3 Hz, 1H), 4.00 (d, *J* = 11.2 Hz, 1H), 4.35 (d, *J* = 11.2 Hz, 1H), 4.76 (dd, *J* = 1.8, 8.7 Hz, 1H), 6.74-6.76 (m, 2H), 7.07-7.09 (m, 2H), 7.12-7.30 (m, 11H) ppm. ^{13}C NMR (100 MHz, $CDCl_3$): δ = 1.18, 20.2, 43.0, 71.4, 73.5, 80.1, 84.2, 126.1, 126.3, 127.2, 127.4, 127.5, 127.9, 128.1, 128.2, 128.4, 128.5, 137.7, 144.7 ppm. HRMS (ESI+) calculated for $C_{33}H_{52}O_3Si_4Na$ ($[M+Na]^+$) : 631.2885, found : 631.2877. $[\alpha]_D^{26}$ -16.13 (c 0.62, $CHCl_3$).

(34): (3*R*,4*S*,5*R*,6*R*)/(3*S*,4*R*,5*S*,6*S*)-4-allyloxy-5-tris(trimethylsilyl)siloxy-6-phenylhept-1-en-3-ol (24) :

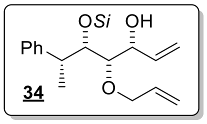

(63%, >99/1/0 *syn/anti*); colorless oil; TLC (hexane:AcOEt, 95:5) R_f = 0.26; ^{1}H NMR (400 MHz, $CDCl_3$): δ = 0.23 (s, 27H), 1.29 (d, *J* = 7.1 Hz, 3H), 2.48 (d, *J* = 7.8 Hz, 1H), 3.20-3.23 (m, 2H), 3.73 (ddt, *J* = 1.2, 6.0, 12.4 Hz, 1H), 3.83 (dd, *J* = 4.0, 7.6 Hz, 2H), 4.29-4.32 (m, 1H), 4.89-4.98 (m, 2H), 5.10 (dt, *J* = 1.6, 17.2 Hz, 1H), 5.45-5.55 (m, 1H), 5.78-5.85 (m, 1H), 7.12-7.28 (m, 5H) ppm. ^{13}C NMR (100 MHz, $CDCl_3$): δ = 1.1, 20.2, 42.5, 70.0, 71.8, 79.5, 82.6, 115.1, 116.5, 125.9, 127.8, 128.2, 134.3, 139.2, 145.5 ppm. HRMS (ESI+) calculated for $C_{25}H_{48}O_3Si_4Na$ ($[M+Na]^+$) : 531.2573, found : 531.2569. $[\alpha]_D^{26}$ -16.95 (c 0.59, $CHCl_3$).

(35): (3*R*,4*S*,5*R*)/(3*S*,4*R*,5*S*)-4-benzyloxy-7,7-dimethyl-5-tris(trimethylsilyl)siloxyoct-3-ol

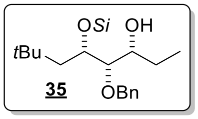

(33%, >99/1/0 *syn/anti*); colorless oil; TLC (hexane:AcOEt, 95:5) R_f = 0.37; ^{1}H NMR (400 MHz, $CDCl_3$): δ = 0.18 (s, 27H), 0.95 (s, 9H), 1.00 (t, *J* = 7.3 Hz, 3H), 1.42 (dd, *J* = 6.4, 14.2 Hz, 1H), 1.42-1.50 (m, 1H), 1.60-1.65 (m, 1H), 1.77 (dd, *J* = 5.8, 14.4 Hz, 1H), 2.29 (br. s, 1H), 3.34 (dd, *J* = 2.8, 4.6 Hz, 1H), 3.76-3.85 (m, 2H), 4.62 (d, *J* = 11.2 Hz, 1H), 4.71 (d, *J* = 11.2 Hz, 1H), 7.25-7.36 (m, 5H), ppm. ^{13}C NMR (100 MHz, $CDCl_3$): δ = -0.5, 1.0, 10.5, 27.4, 30.8, 46.1, 71.5, 72.2, 73.9, 83.6, 127.5, 127.7, 128.5, 138.6 ppm. HRMS (ESI+) calculated for $C_{26}H_{54}O_3Si_4Na$ ($[M+Na]^+$) : 549.3042, found : 549.3046.

(36): (3*R*,4*S*,5*R*)/(3*S*,4*R*,5*S*)-4-allyloxy-7,7-dimethyl-5-tris(trimethylsilyl)siloxyoct-3-ol

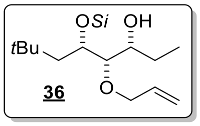

(36%, >99/1/0 *syn/anti*); colorless oil; (hexane:AcOEt, 90:10) R_f = 0.44; ^{1}H NMR (400 MHz, $CDCl_3$): δ = 0.18 (s, 27H), 0.94 (s, 9H), 1.00 (t, *J* = 7.3 Hz, 3H), 1.38 (dd, *J* = 6.0, 14.2 Hz, 1H), 1.38-1.49 (m, 1H), 1.57-1.65 (m, 1H), 1.72 (dd, *J* = 6.0, 14.2 Hz, 1H), 2.28 (br. s, 1H), 3.19 (dd, *J* = 3.9, 5.3 Hz, 1H), 3.72-3.73 (m, 2H), 4.12 (qd, *J* = 5.5, 12.6 Hz, 2H), 5.14 (d, *J* = 10.6 Hz, 1H), 5.27 (d, *J* = 17.2 Hz, 1H), 5.86-5.93 (m, 1H), ppm. ^{13}C NMR (100 MHz, $CDCl_3$): δ = -0.5, 1.1, 10.5, 27.4, 30.7, 46.1, 71.1, 71.5, 73.9, 83.3, 116.5, 135.0 ppm. HRMS (ESI+) calculated for $C_{22}H_{52}O_3Si_4Na$ ($[M+Na]^+$) : 499.2885, found : 499.2865.

5. Synthesis of the pentose-like scaffold

<u>GP4. Procedure for the Wittig reaction</u>

To a stirring suspension of methyltriphenylphosphium bromide (104 mg, 0.29 mmol) in anhydrous THF (1.8 mL) at 0°C was added *n*-Butyllithium (0.19 mL, 0.30 mmol, 1.6 M in hexane), dropwise. The yellow solution was stirred at this temperature for 1h then cooled to −78°C. In a separate flask, aldehyde **24d** was dissolved in anhydrous THF (0.2 mL) and added to the ylide solution by syringe, drop wise. The reaction was slowly warmed to room temperature and stirred until TLC analysis indicated total conversion of the starting aldehyde. The reaction was then quenched by the addition of 3 mL MeOH/H_2O (3:2 v/v) and 2 mL of saturated aqueous NH_4Cl. 5 mL of hexane was then added. The layers were separated and the organic layer was washed with 5 mL of H_2O and 5 mL of brine. The organic layer was dried over Na_2SO_4, filtered through cotton and evaporated. The crude mixture was purified by flash chromatography with hexane as eluent.

(3*R*,4*S*,5*R*)/(3*S*,4*R*,5*S*)-3-allyloxy-4-tris(trimethylsilyl)siloxy-5-phenylhex-1-ene

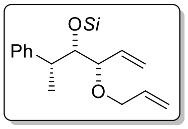

(64%, 98/2 *syn/anti*); colorless oil; TLC (hexane: AcOEt, 95:5) R_f = 0.71; ^{1}H NMR (400 MHz, $CDCl_3$): δ = 0.18 (s, 27H), 1.22 (d, *J* = 7.1 Hz, 3H), 3.00-3.07 (m, 1H), 3.72-3.80 (m, 3H), 3.91-3.96 (m, 1H), 5.01-5.10 (m, 2H), 5.21-5.26 (m, 2H), 5.72-5.80 (m, 1H), 5.84-5.93 (m, 1H), 7.13-7.19 (m, 3H), 7.22-7.28 (m, 2H) ppm. ^{13}C NMR (100 MHz, $CDCl_3$): δ = 0.9, 17.4, 41.1, 69.8, 81.8, 82.4, 115.8, 116.5, 125.8, 128.1, 128.1, 135.1, 135.3, 146.3 ppm. HRMS (ESI+) calculated for $C_{24}H_{46}O_2Si_4Na$ ($[M+Na]^+$) : 501.2467, found : 501.2469. $[\alpha]_D^{26}$ -21.43 (c 1.12, $CHCl_3$).

<u>GP5. Procedure for the RCM reaction</u>

In an oven dried round bottom flask equipped with condenser, 2 mol% of Grubbs catalyst 2nd generation (8 mg, 0.009 mmol) was added to a solution of the olefin (0.47 mmol) in anhydrous dichloromethane (6 mL) and the mixture was then heated at 40 °C for 2h until TLC analysis indicated consumption of starting material. The mixture was then cooled to room temperature and concentrated in vacuum. The crude product was then purified by silica gel flash chromatography using hexane/EtOAc as eluent.

(37): (1*S*,2*S*)/(1*R*,2*R*)-1-((*S*)-2,5-dihydrofuran-2-yl)-1-tris(trimethylsilyl)siloxy-2-phenylpropane

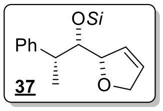

(95%, 98/2 *syn/anti*); colorless oil; TLC (hexane:AcOEt, 95:5) R_f = 0.51; ^{1}H NMR (400 MHz, $CDCl_3$): δ = 0.17 (s, 27H), 1.22 (d, *J* = 7.1 Hz, 3H), 2.72-2.75 (m, 1H), 3.71 (dd, *J* = 4.8, 5.7 Hz, 1H), 3.92-3.98 (m, 1H), 4.35-4.40 (m, 1H), 4.80-4.83 (m, 1H), 5.78-5.80 (m, 1H), 5.82-5.84 (m, 1H), 7.10-7.15 (m, 3H), 7.21-7.25 (m, 2H) ppm. ^{13}C NMR (100 MHz, $CDCl_3$): δ = 0.8, 18.1, 42.2, 75.0, 82.6, 89.6, 125.7, 126.5, 128.0, 128.1, 128.2, 145.7 ppm. HRMS (ESI+) calculated for $C_{22}H_{42}O_2Si_4Na$ ($[M+Na]^+$) : 473.2154, found : 473.2146.

<u>GP6. Optimization of the asymmetric dihydroxylation</u>

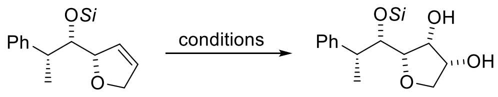

37 **38**

entry	conditions	results
1	OsO_4<br>NMO (2 equiv)<br>*t*-BuOH/H_2O/acetone (17/1/1.3)<br>r.t, 12h	65% yield<br>62:38 dr<br>100% conversion
2	AD-mix-α<br>$MeSO_2NH_2$ (3 equiv)<br>*t*-BuOH/H_2O (1:1)<br>0 °C, 4 days	74% yield<br>82:18 dr<br>100% conversion
3	AD-mix-β<br>$MeSO_2NH_2$ (3 equiv)<br>*t*-BuOH/H_2O (1:1)<br>0 °C, 4 days	73% yield<br>98:2 dr<br>100% conversion

<u>Typical procedure (entry 3):</u> AD-mix-β (124 mg) was dissolved in *t*-BuOH (0.4 mL) and H_2O (0.4 mL). Methanesulfonamide (25 mg, 0.266 mmol) and alkene **37** (40 mg, 0.088 mmol) dissolved in 0.5 mL of *t*-BuOH/H_2O (1:1) were then added at 0 °C and the reaction vigorously stirred for 4 days at same temperature. After complete consumption of the starting material, Na_2SO_3 (122 mg) was then added and the solution stirred for 1h after which the reaction was poured into water (3 mL) and extracted with EtOAc (3 x 5 mL). The combined organics were washed with brine and dried over anhydrous Na_2SO_4. The solvent was removed under reduced pressure to yield the crude diol which was purified by silica gel column chromatography using Hexane/EtOAc (3/2) to yield pure diol **38** (31 mg, 73%) as white solid.

(38): (2*R*,3*S*,4*S*)-2-((1*S*,2*R*)-1-tris(trimethylsilyl)siloxy-2-phenylpropyl)-tetrahydrofuran-3,4-diol

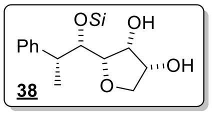

(73%, dr = 98/2, **38**-(*all-syn*)); white solid; TLC (hexane:EtOAc, 60:40) R_f = 0.51, ^{1}H NMR (400 MHz, $CDCl_3$): δ = 0.09 (s, 27H), 1.26 (d, *J* = 7.1 Hz, 3H), 1.90 (d, *J* = 4.4 Hz, 1H; C3-OH), 2.41 (d, *J* = 7.8 Hz, 1H; C4-OH), 2.98 (dq, *J* = 4.4, 7.1 Hz, 1H; C7-H), 3.50 (d, *J* = 2.8, 8.5 Hz, 1H; C5-H), 3.60 (d, *J* = 6.4, 9.2Hz, 1H; C2a-H), 3.75 (dd, *J* = 2.4, 4.8 Hz, 1H; C4-H), 3.80 (dd, *J* = 7.6, 9.2 Hz, 1H; C3-H), 3.90 (dd, *J* = 4.6, 8.5 Hz, 1H; C6-H), 4.29-4.35 (m, 1H; C5b-H), 7.18-7.21 (m, 1H), 7.23-7.29 (m, 4H) ppm. ^{13}C NMR (100 MHz, $CDCl_3$): δ = 0.8, 15.5, 42.7, 71.4, 71.7, 72.4, 80.1, 85.5, 126.5, 128.0, 128.5, 145.2 ppm. HRMS (ESI+) calculated for $C_{22}H_{44}O_4Si_4Na$ ($[M+Na]^+$) : 507.2209, found : 507.2199. $[\alpha]_D^{24}$ -7.69 (c 1.3, $CHCl_3$).

Stereochemistry of compound **38** was assigned *all-syn* by carrying out NOE and NOESY experiments in addition to analysis of coupling constant values of the ^{1}H NMR spectra.

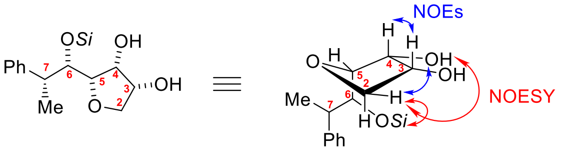

6. Synthesis of the Hexose-like scaffold

(39): (2*S*,3*S*)-2-((1*R*,2*S*)-1-hydroxy-2-phenylpropyl)-3,6-dihydro-2H-pyran-3-ol

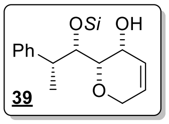

Prepared following **GP5**, (97%, >99/1/0 *syn/anti*); colorless oil; TLC (hexane:AcOEt, 90:10) R_f = 0.33; ^{1}H NMR (400 MHz, $CDCl_3$): δ = 0.06 (s, 27H), 1.26 (d, *J* = 7.3 Hz, 3H), 3.18 (qd, *J* = 1.6, 7.3 Hz, 1H), 3.24 (dd, *J* = 1.6, 8.5 Hz, 1H), 3.85-3.90 (m, 1H), 4.00 (dd, *J* = 2.1, 8.5 Hz, 1H), 4.00-4.07 (m, 1H), 4.20 (ddd, *J* = 1.6, 3.5, 16.9 Hz, 1H), 5.92 (ddd, *J* = 1.4, 3.4, 10.1, 1H), 5.96-6.16 (m, 1H), 7.14-7.18 (m, 1H), 7.24-7.28 (m, 2H), 7.30-7.33 (m, 2H) ppm. ^{13}C NMR (100 MHz, $CDCl_3$): δ = 0.8, 13.0, 40.2, 62.8, 66.4, 81.0, 81.4, 126.1, 126.8, 128.1, 128.3, 130.6, 144.7 ppm. HRMS (ESI+) calculated for $C_{23}H_{44}O_3Si_4Na$ ($[M+Na]^+$) : 503.2260, found : 503.2254. $[\alpha]_D^{26}$ -50.51 (c 0.99, $CHCl_3$).

GP7. Optimization of the asymmetric dihydroxylation

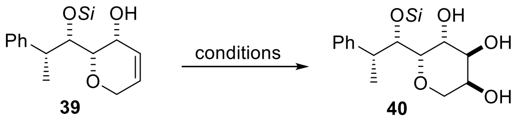

39 **40**

entry	conditions	results
1	OsO_4<br>NMO (2 equiv)<br>*t*-BuOH/H_2O/acetone (17/1/1.3)<br>r.t, 12h	68% yield<br>>98:2 dr<br>100% conversion
2	AD-mix-α<br>$MeSO_2NH_2$ (3 equiv)<br>*t*-BuOH/H_2O (1:1)<br>0 °C, 4 days	62% yield<br>98:2 dr<br>54% conversion
3	AD-mix-β<br>$MeSO_2NH_2$ (3 equiv)<br>*t*-BuOH/H_2O (1:1)<br>0 °C, 4 days	74% yield<br>96:4 dr<br>50% conversion

Typical procedure (entry 1): A solution (0.7 mL) of osmium tetroxide in *t*-BuOH (osmium tetroxide/*t*-BuOH = 1/30 w/v) was added to a mixture of **39** (70 mg, 0.145 mmol) and *N*-methylmorpholine *N*-oxide (36 mg, 0.435 mmol) in acetone/water (0.5 mL, 8/1, v/v) and stirred vigorously for overnight at room temperature. After TLC analysis indicated full consumption of starting material, Na_2SO_3 (420 mg) was added to the reaction mixture with ice-cooling and was then vigorously stirred for 30 min at room temperature. The reaction mixture was evaporated under reduced pressure and the crude triol was purified by silica gel chromatography using hexane/methanol (95/5) as eluent.

(40): (2*S*,3*R*,4*S*,5*S*)-2-((1*S*,2*S*)-1-(tris(trimethylsilyl)siloxy)-2-phenylpropyl)tetrahydro-2H-pyran-3,4,5-triol

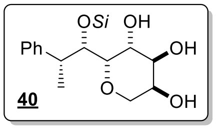

(68%, dr >98/2, **40**-*syn-syn-syn-anti-syn*); white solid; TLC (DCM:MeOH, 90:10) R_f = 0.40; ^{1}H NMR (400 MHz, $CDCl_3$): δ = 0.08 (s, 27H), 1.26 (d, *J* = 7.4 Hz, 3H), 2.01 (d, *J* = 8.4 Hz, 1H; OH), 2.29 (br. s, 1H, OH), 2.51 (br. s, 1H; OH), 3.02 (qd, *J* = 3.0, 7.1 Hz, 1H; C8-H), 3.31 (t, *J* = 10.8 Hz, 1H; C4-H), 3.50 (d, *J* = 7.8 Hz, 1H; C6-H), 3.72 (dd, *J* = 5.5, 10.6 Hz, 1H; C5-H), 3.78-3.81 (m, 1H; C3-H), 3.85 (dd, *J* = 3.2, 7.8 Hz, 1H; C7-H), 3.94-3.97 (m, 2H; C2-H), 7.14-7.18 (m, 1H), 7.23-7.28 (m, 4H) ppm. ^{13}C NMR (100 MHz, $CDCl_3$): δ = 0.9, 14.2, 41.2, 64.5, 66.4, 70.1, 70.3, 77.9, 80.8, 126.3, 128.2, 128.3, 144.4 ppm. HRMS (ESI+) calculated for $C_{23}H_{46}O_5Si_4Na$ ($[M+Na]^+$) : 537.2315, found : 537.2305. $[\alpha]_D^{24}$ -10.75 (c 0.93, $CHCl_3$).

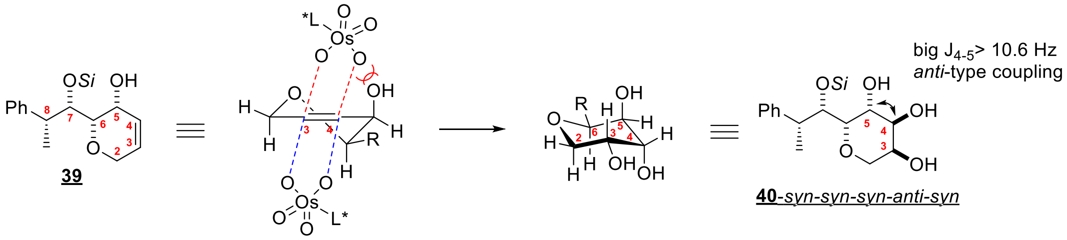

prefered attack from α-face

[1] Benzyloxyacetaldehyde was purchased from aldrich and used as received. All other protected β-hydroxyacetaldehydes were prepared according to reported procedure starting from ethyl glycolate : Angle, S. R.; Choi, I.; Tham, F. S. *J. Org. Chem.* 2008, **73**, 6268-6278.

7. ^{1}H, ^{13}C, ^{19}F, NOE and NOESY spectra of compound 1a to 40

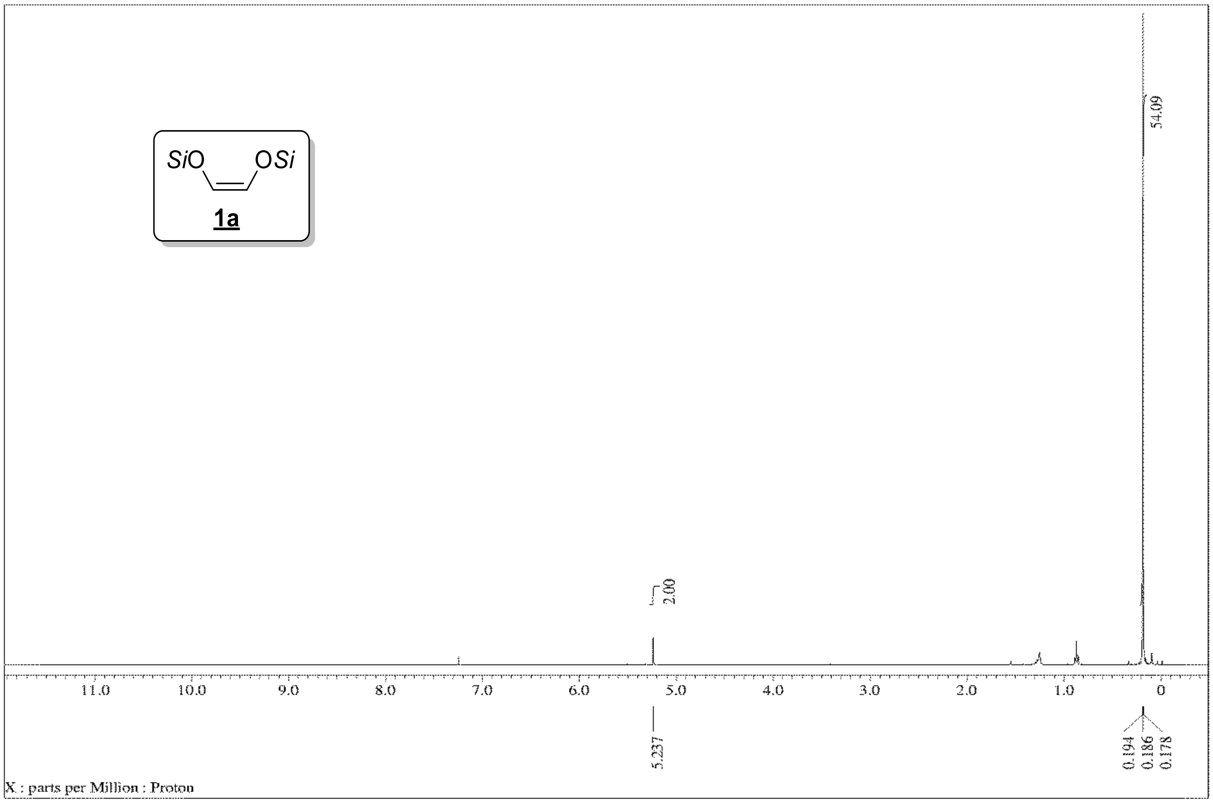

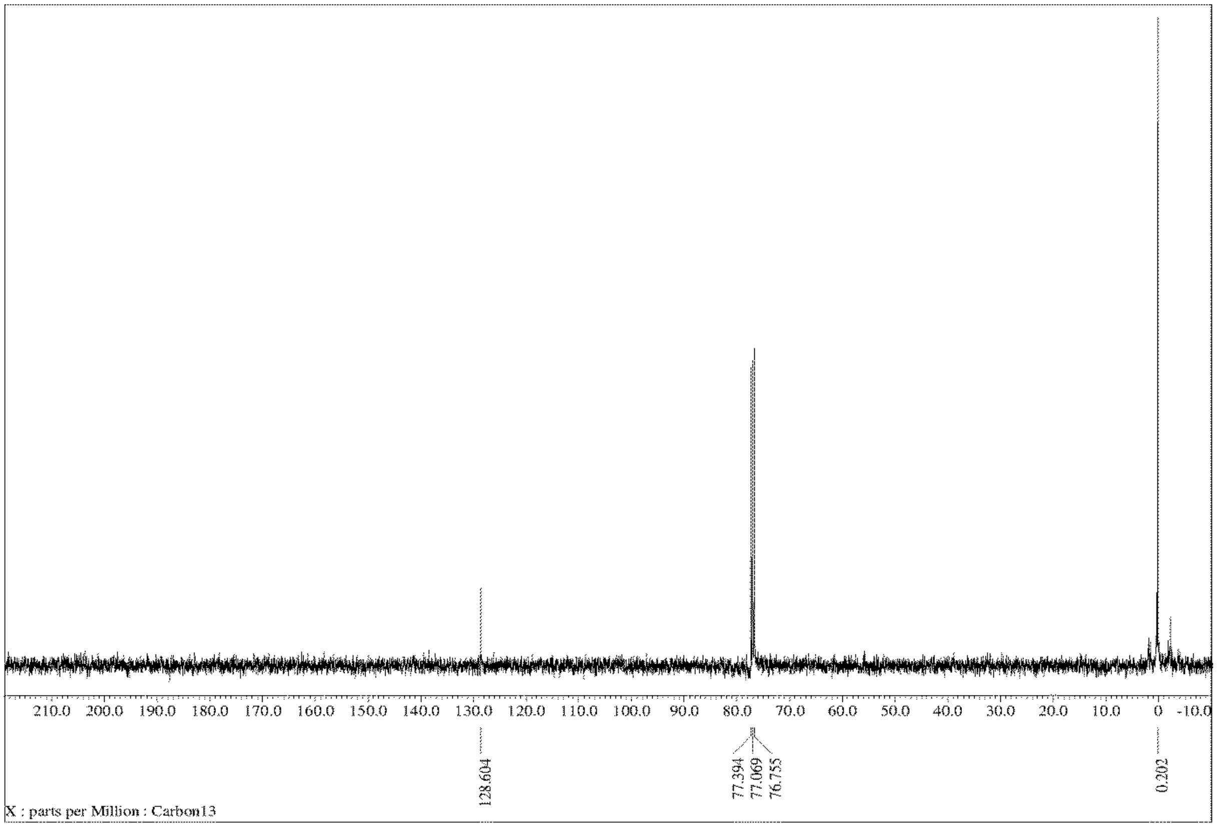

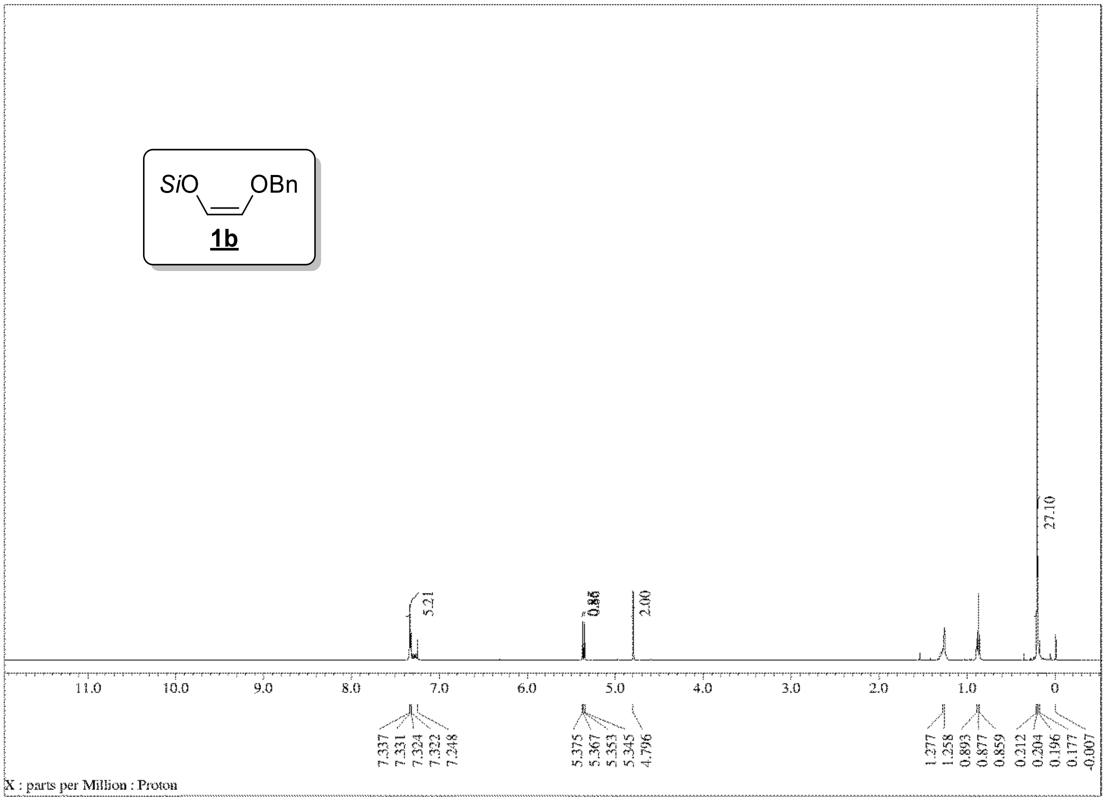
SiO OBn
1b
5.21
0.86
2.00
27.10
11.0 10.0 9.0 8.0 7.0 6.0 5.0 4.0 3.0 2.0 1.0 0
7.337 7.331 7.324 7.322 7.248
5.375 5.367 5.353 5.345 4.796
1.277 1.258 0.893 0.877 0.859 0.212 0.204 0.196 0.177 -0.007
X : parts per Million : Proton

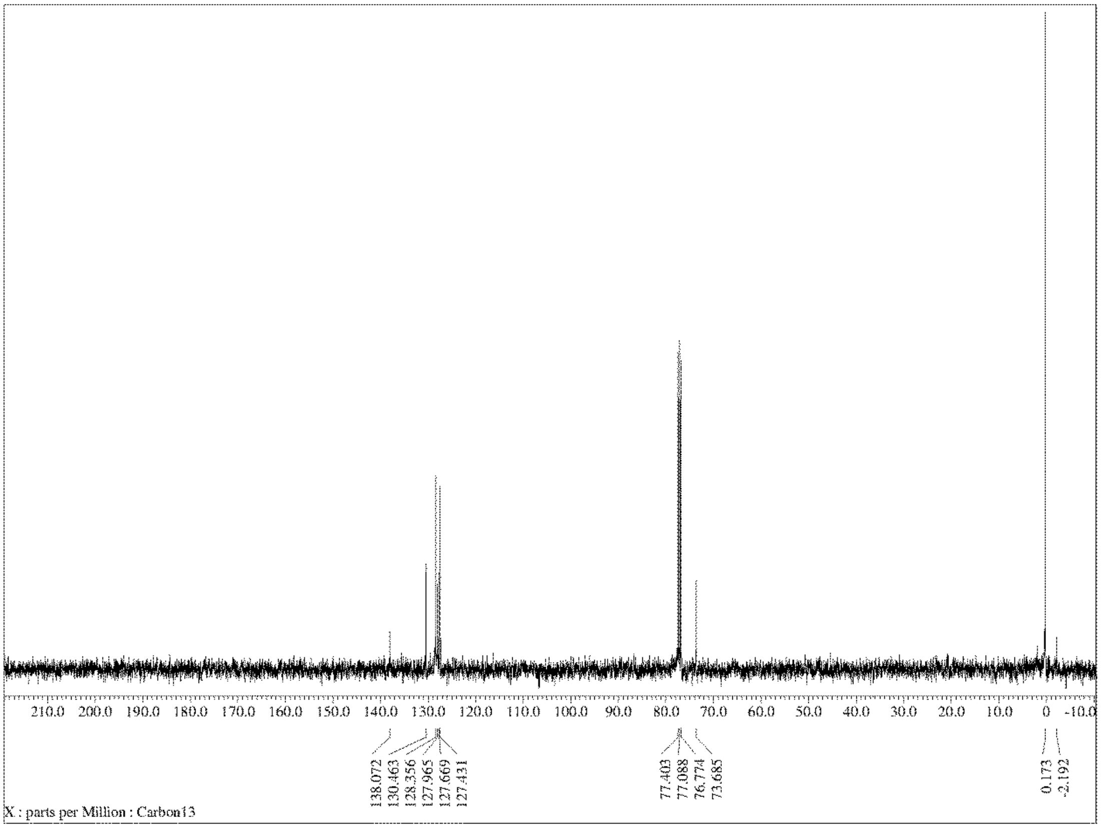
210.0 200.0 190.0 180.0 170.0 160.0 150.0 140.0 130.0 120.0 110.0 100.0 90.0 80.0 70.0 60.0 50.0 40.0 30.0 20.0 10.0 0 -10.0
138.072 130.463 128.356 127.965 127.669 127.431
77.403 77.088 76.774 73.685
0.173 -2.192
X : parts per Million : Carbon13

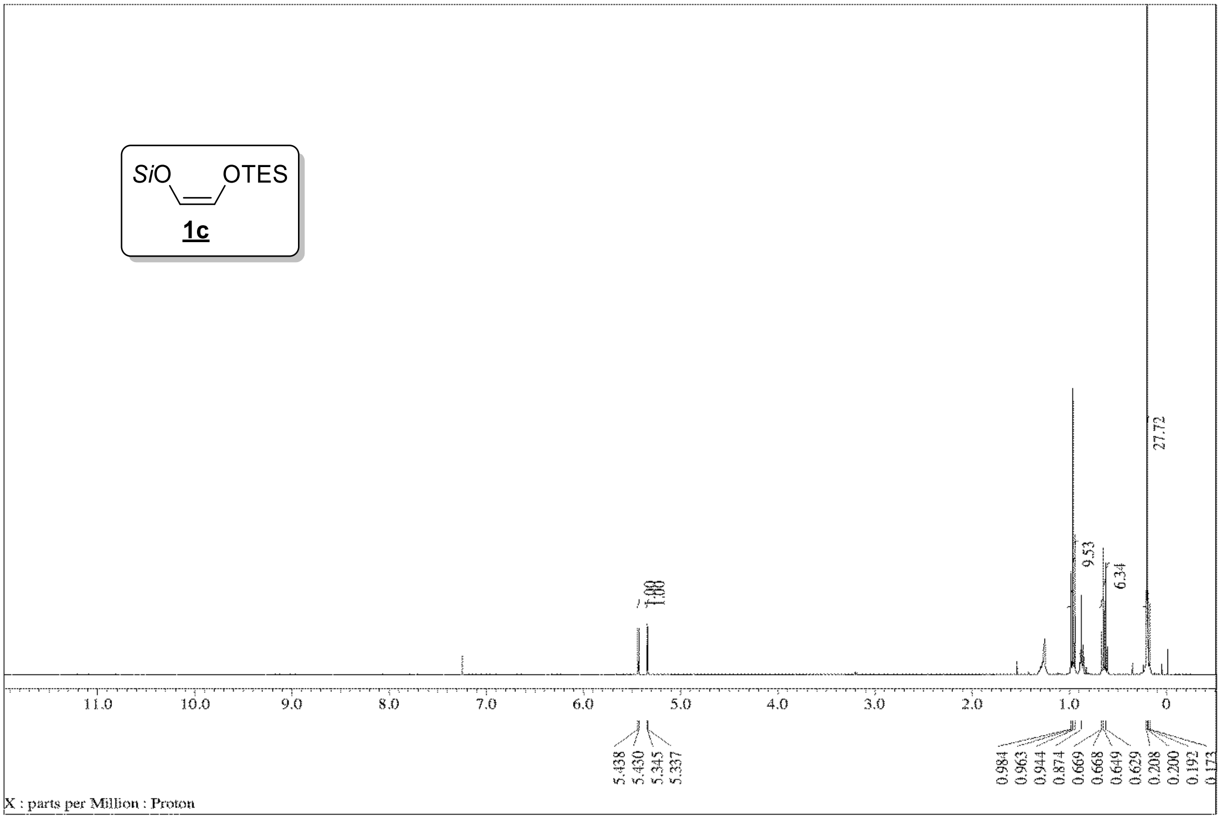

SiO OTES
1c
27.72
9.53
6.34
1.00
1.00
11.0 10.0 9.0 8.0 7.0 6.0 5.0 4.0 3.0 2.0 1.0 0
5.438
5.430
5.345
5.337
0.984
0.963
0.944
0.874
0.669
0.668
0.649
0.629
0.208
0.200
0.192
0.173
X : parts per Million : Proton

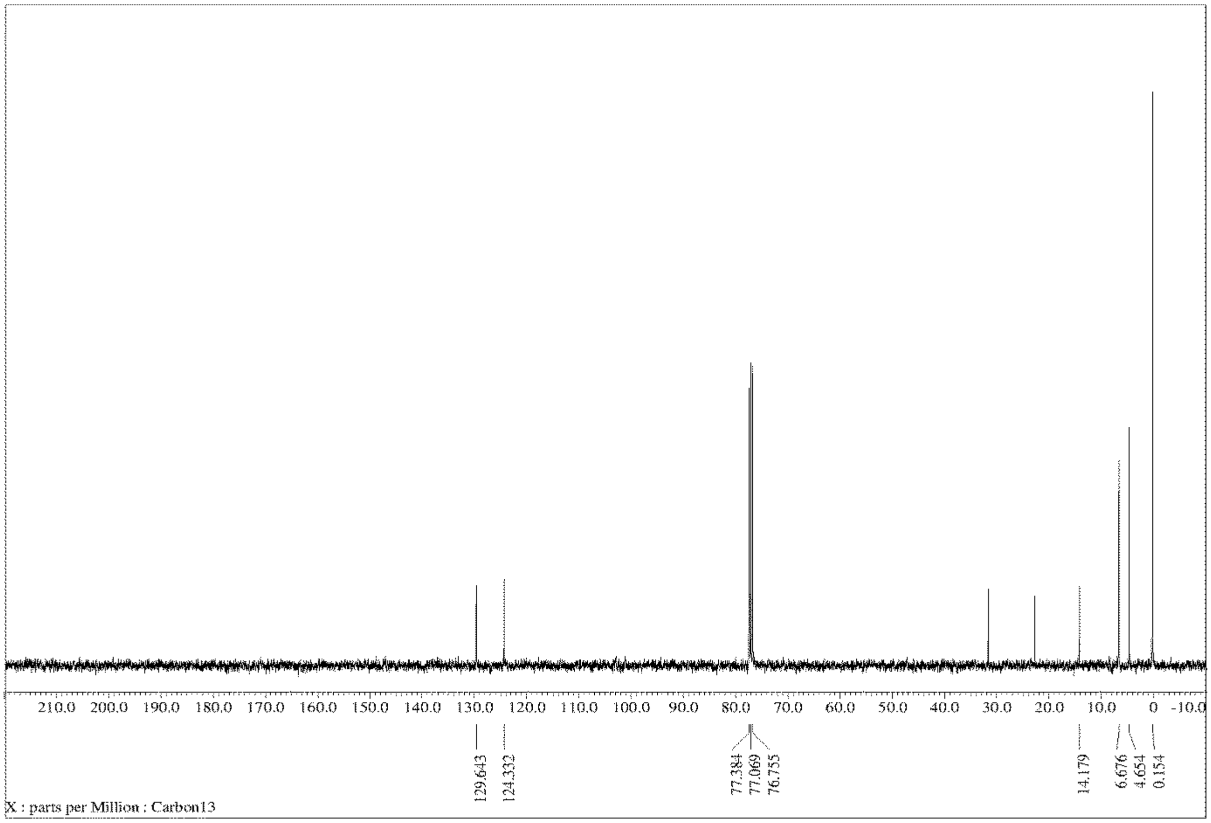

210.0 200.0 190.0 180.0 170.0 160.0 150.0 140.0 130.0 120.0 110.0 100.0 90.0 80.0 70.0 60.0 50.0 40.0 30.0 20.0 10.0 0 -10.0
129.643
124.332
77.384
77.069
76.755
14.179
6.676
4.654
0.154
X : parts per Million : Carbon13

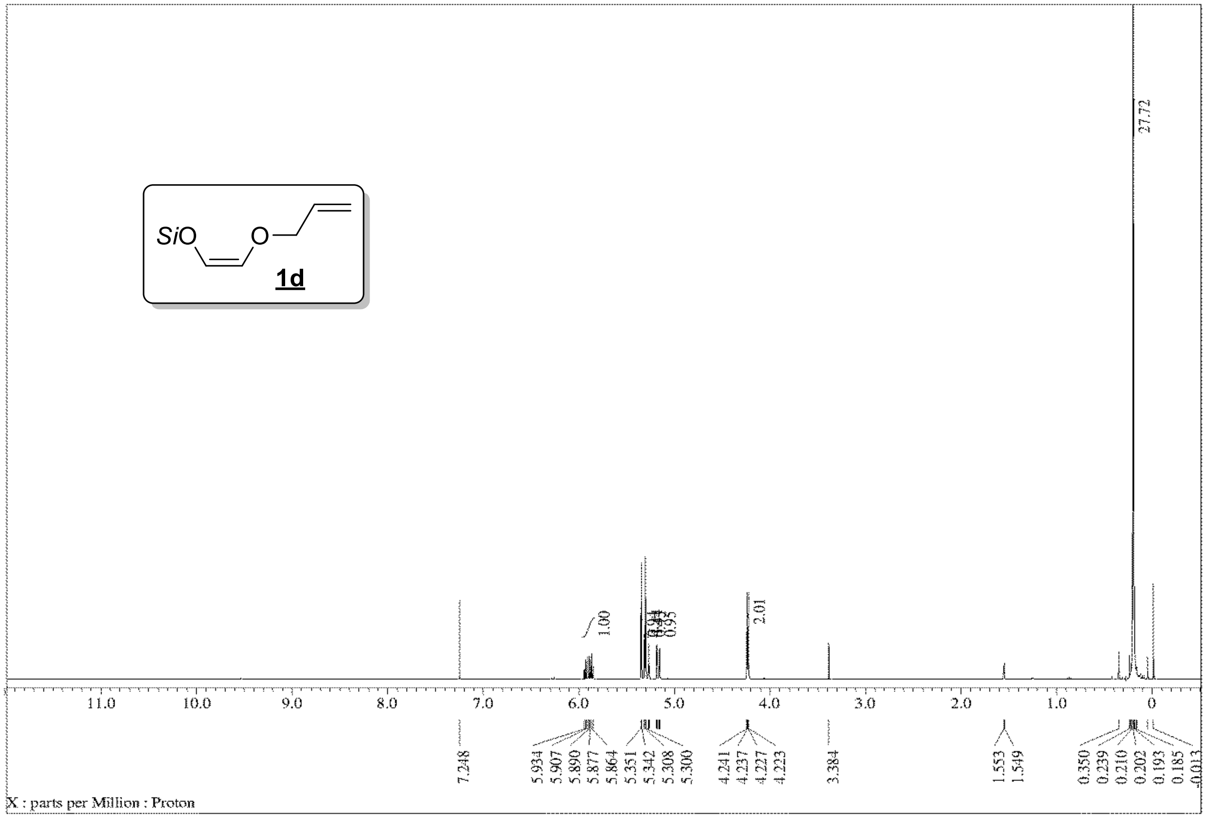
SiO
O
1d
27.72
1.00
2.01
0.94
0.95
11.0
10.0
9.0
8.0
7.0
6.0
5.0
4.0
3.0
2.0
1.0
0
7.248
5.934
5.907
5.890
5.877
5.864
5.351
5.342
5.308
5.300
4.241
4.237
4.227
4.223
3.384
1.553
1.549
0.350
0.239
0.210
0.202
0.193
0.185
-0.013
X : parts per Million : Proton

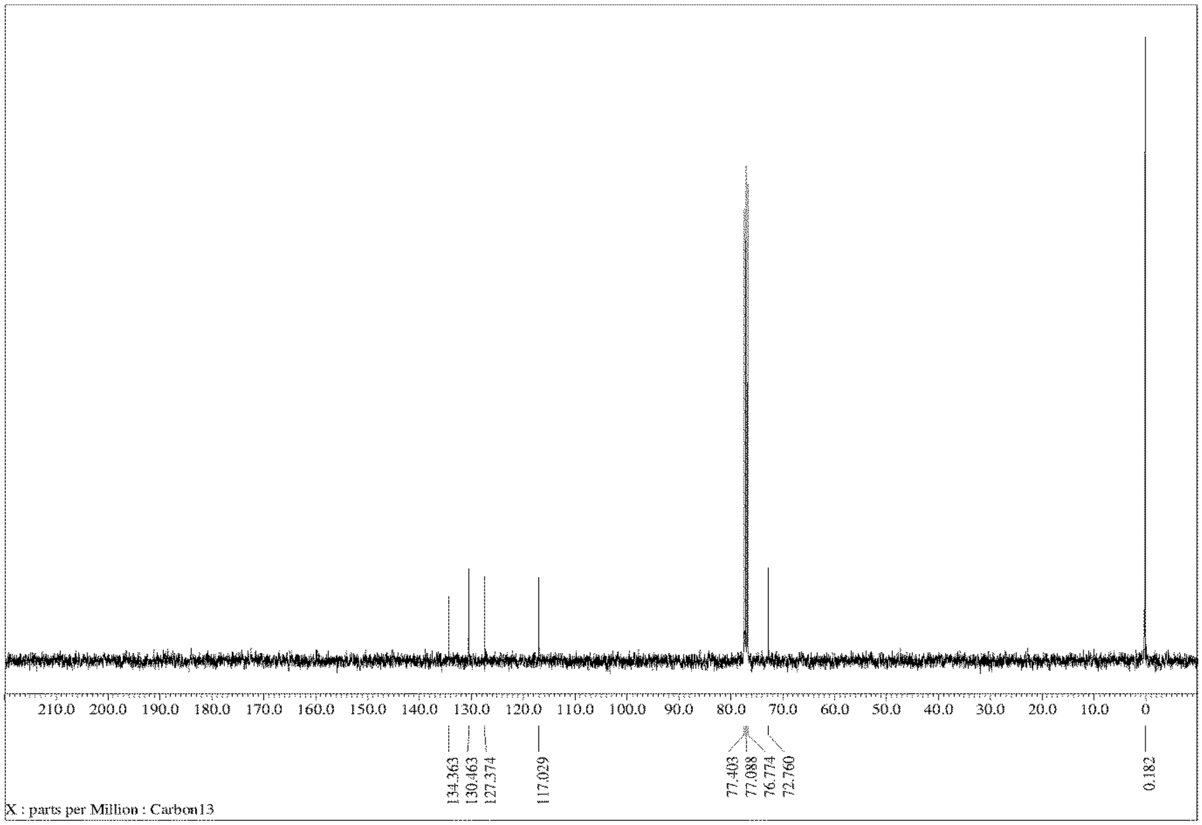
210.0
200.0
190.0
180.0
170.0
160.0
150.0
140.0
130.0
120.0
110.0
100.0
90.0
80.0
70.0
60.0
50.0
40.0
30.0
20.0
10.0
0
134.363
130.463
127.374
117.029
77.403
77.088
76.774
72.760
0.182
X : parts per Million : Carbon13

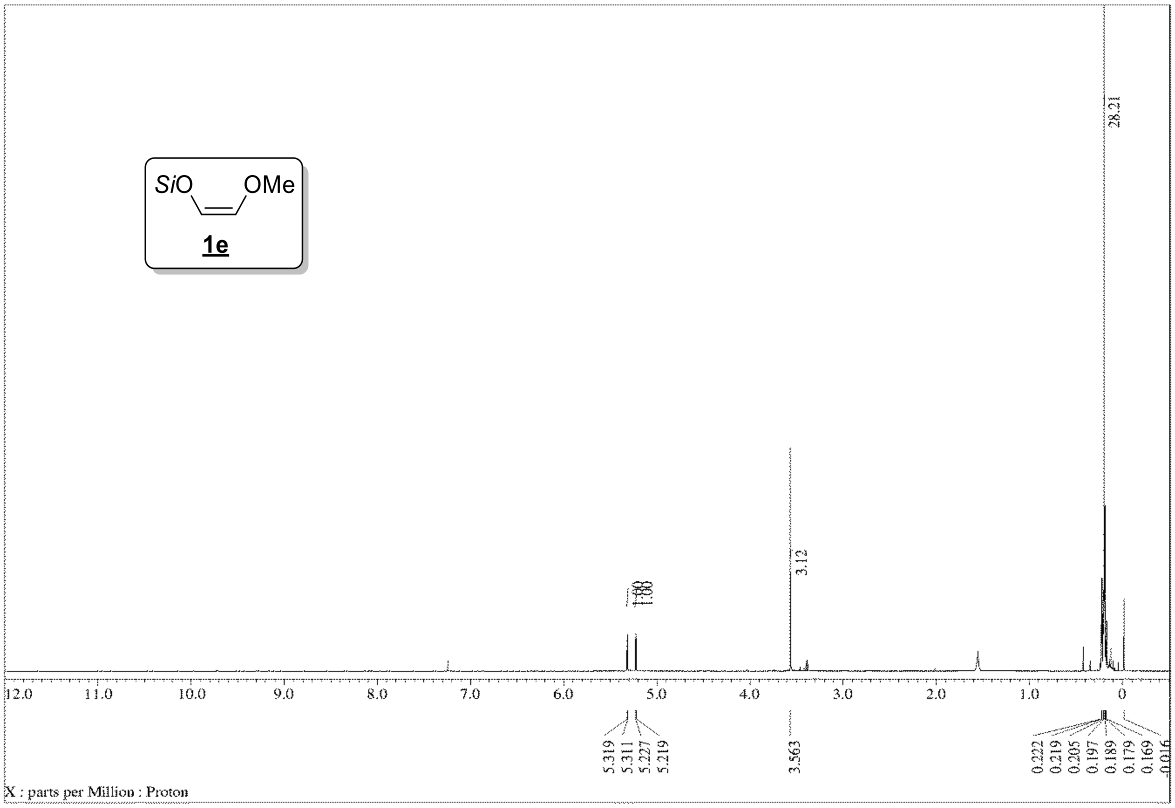
SiO OMe
1e
28.21
3.12
12.0 11.0 10.0 9.0 8.0 7.0 6.0 5.0 4.0 3.0 2.0 1.0 0
5.319
5.311
5.227
5.219
3.563
0.222
0.219
0.205
0.197
0.189
0.179
0.169
X : parts per Million : Proton

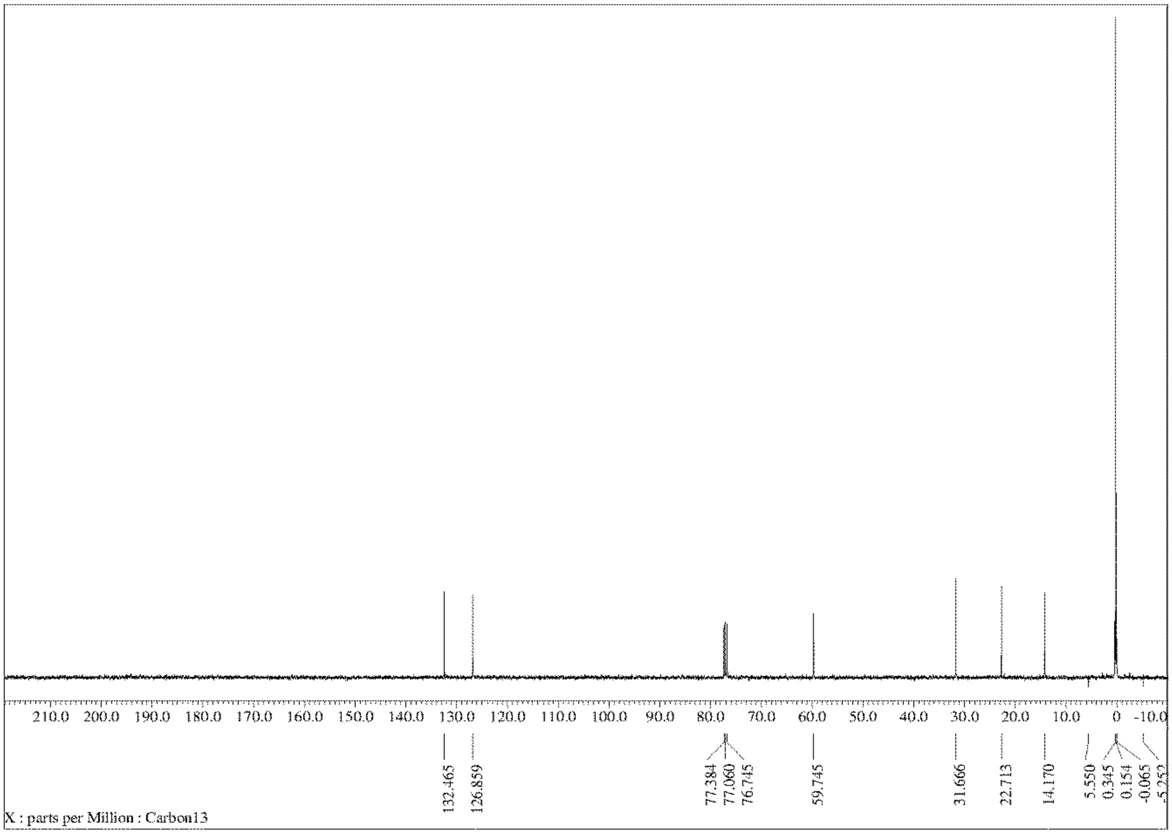
210.0 200.0 190.0 180.0 170.0 160.0 150.0 140.0 130.0 120.0 110.0 100.0 90.0 80.0 70.0 60.0 50.0 40.0 30.0 20.0 10.0 0 -10.0
132.465
126.859
77.384
77.060
76.745
59.745
31.666
22.713
14.170
5.550
0.345
0.154
-0.065
X : parts per Million : Carbon13

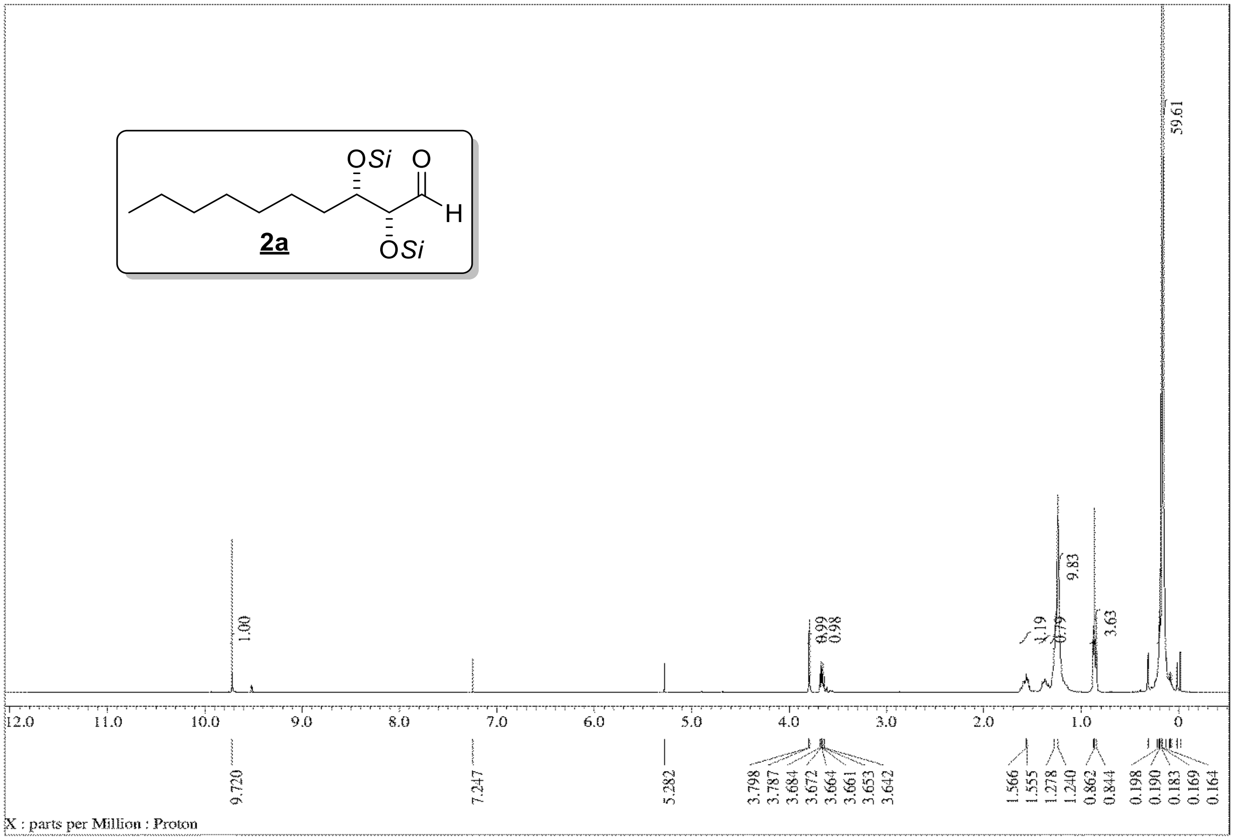
OSi O
H
2a
OSi
59.61
1.00
0.99
0.98
1.19
0.79
9.83
3.63
12.0 11.0 10.0 9.0 8.0 7.0 6.0 5.0 4.0 3.0 2.0 1.0 0
9.720
7.247
5.282
3.798 3.787 3.684 3.672 3.664 3.661 3.653 3.642
1.566 1.555 1.278 1.240 0.862 0.844
0.198 0.190 0.183 0.169 0.164
X : parts per Million : Proton

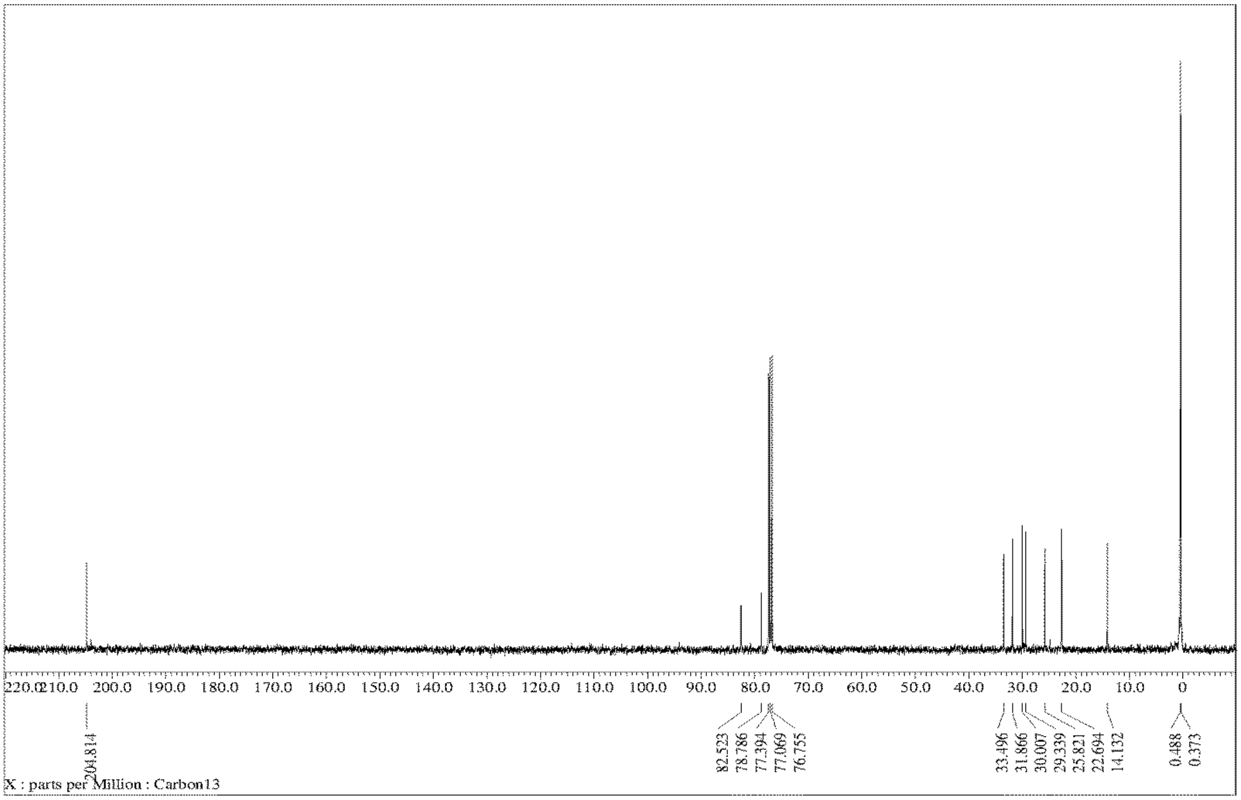
220.0 210.0 200.0 190.0 180.0 170.0 160.0 150.0 140.0 130.0 120.0 110.0 100.0 90.0 80.0 70.0 60.0 50.0 40.0 30.0 20.0 10.0 0
204.814
82.523 78.786 77.394 77.069 76.755
33.496 31.866 30.007 29.339 25.821 22.694 14.132
0.488 0.373
X : parts per Million : Carbon13

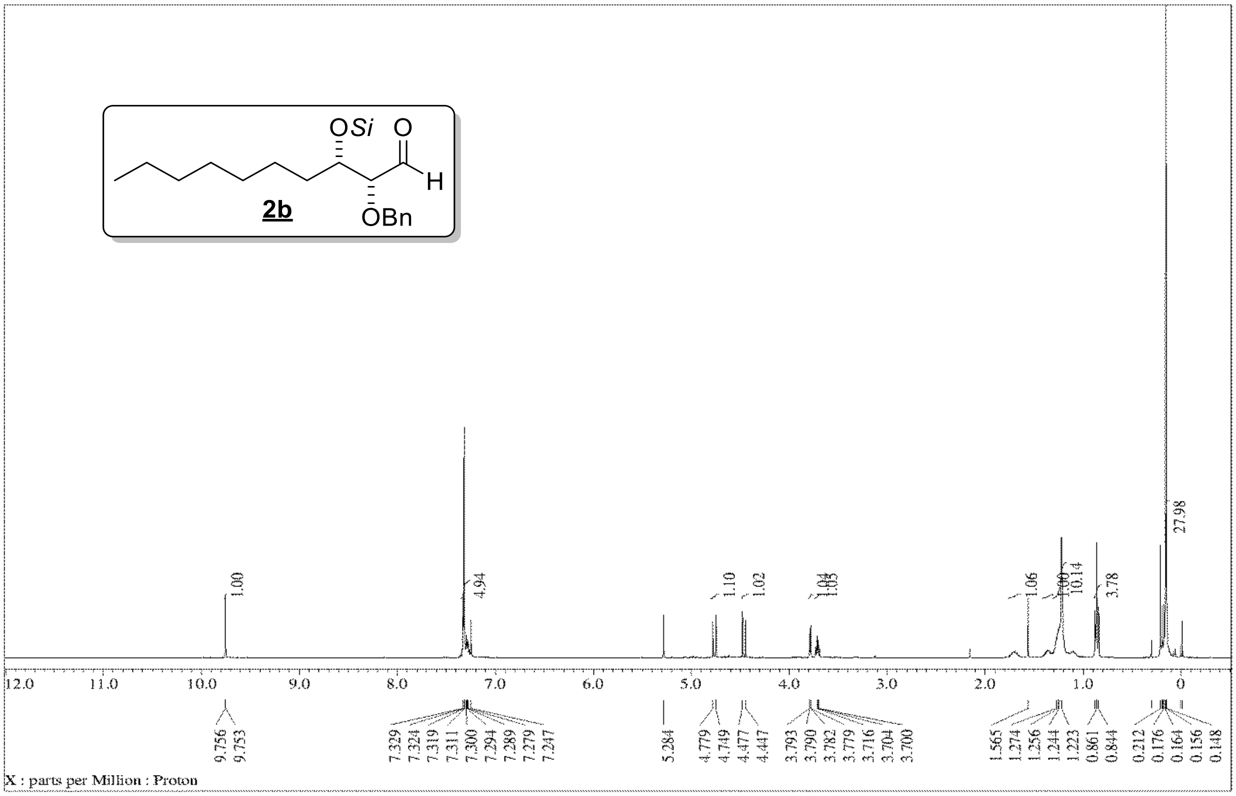
OSi
O
H
2b
OBn
1.00
4.94
1.10
1.02
1.04
1.03
1.06
1.00
10.14
3.78
27.98
12.0 11.0 10.0 9.0 8.0 7.0 6.0 5.0 4.0 3.0 2.0 1.0 0
9.756
9.753
7.329
7.324
7.319
7.311
7.300
7.294
7.289
7.279
7.247
5.284
4.779
4.749
4.477
4.447
3.793
3.790
3.782
3.779
3.716
3.704
3.700
1.565
1.274
1.256
1.244
1.223
0.861
0.844
0.212
0.176
0.164
0.156
0.148
X : parts per Million : Proton

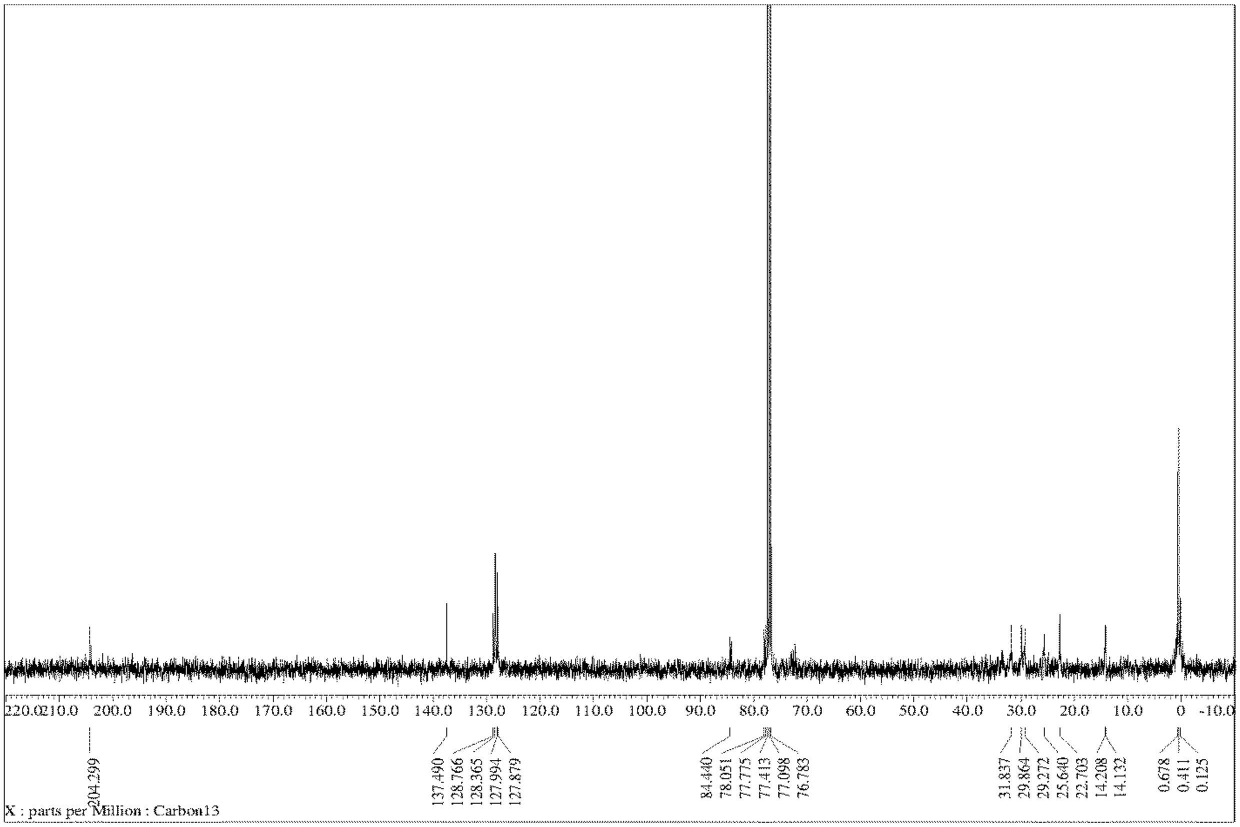
220.0 210.0 200.0 190.0 180.0 170.0 160.0 150.0 140.0 130.0 120.0 110.0 100.0 90.0 80.0 70.0 60.0 50.0 40.0 30.0 20.0 10.0 0 -10.0
204.299
137.490
128.766
128.365
127.994
127.879
84.440
78.051
77.775
77.413
77.098
76.783
31.837
29.864
29.272
25.640
22.703
14.208
14.132
0.678
0.411
0.125
X : parts per Million : Carbon13

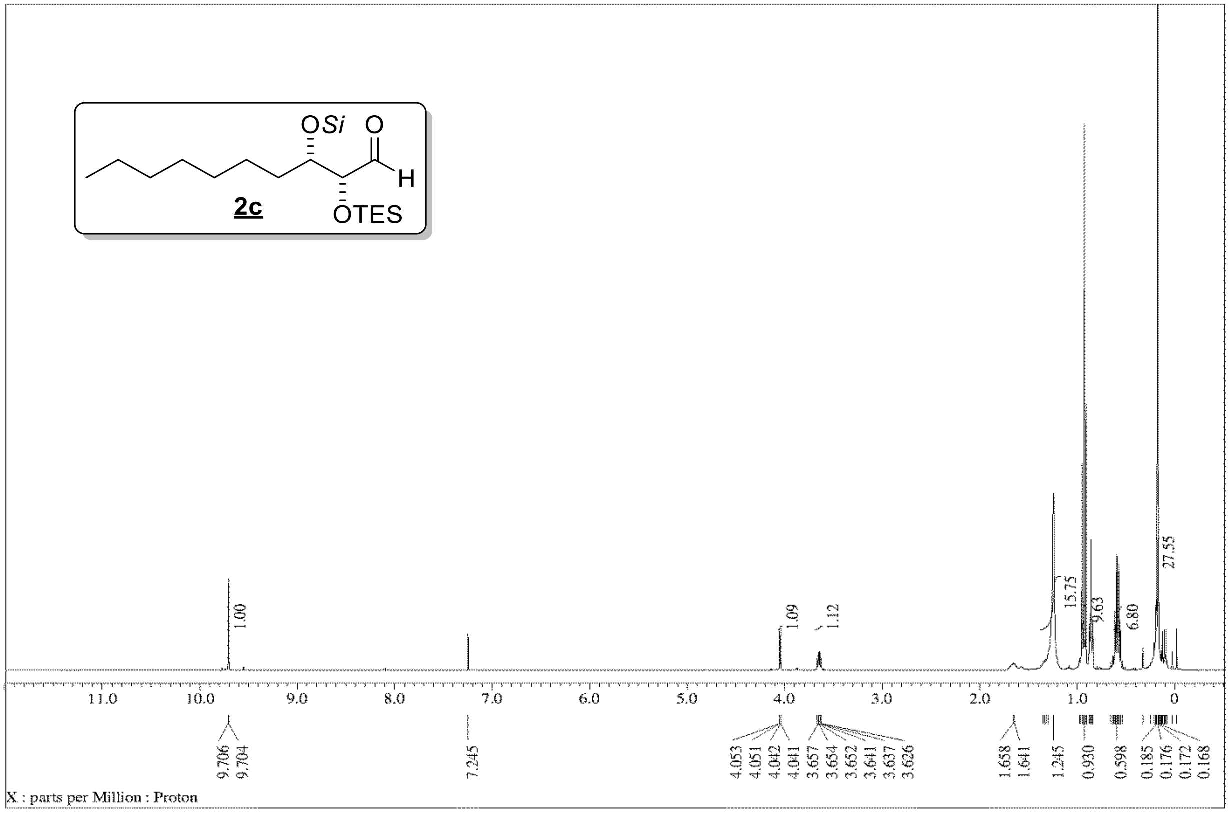
OSi
O
H
2c
OTES
1.00
1.09
1.12
15.75
9.63
6.80
27.55
11.0 10.0 9.0 8.0 7.0 6.0 5.0 4.0 3.0 2.0 1.0 0
9.706
9.704
7.245
4.053
4.051
4.042
4.041
3.657
3.654
3.652
3.641
3.637
3.626
1.658
1.641
1.245
0.930
0.598
0.185
0.176
0.172
0.168
X : parts per Million : Proton

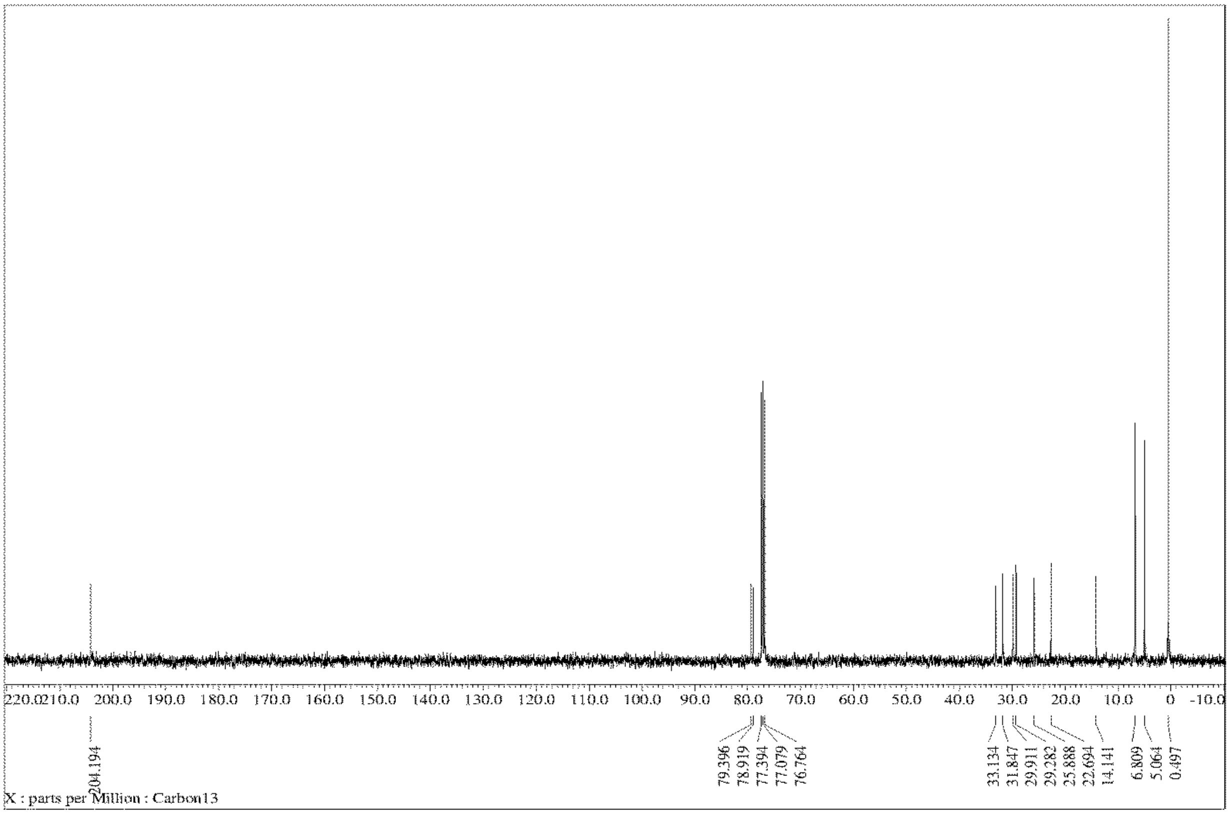
220.0 210.0 200.0 190.0 180.0 170.0 160.0 150.0 140.0 130.0 120.0 110.0 100.0 90.0 80.0 70.0 60.0 50.0 40.0 30.0 20.0 10.0 0 -10.0
204.194
79.396
78.919
77.394
77.079
76.764
33.134
31.847
29.911
29.282
25.888
22.694
14.141
6.809
5.064
0.497
X : parts per Million : Carbon13

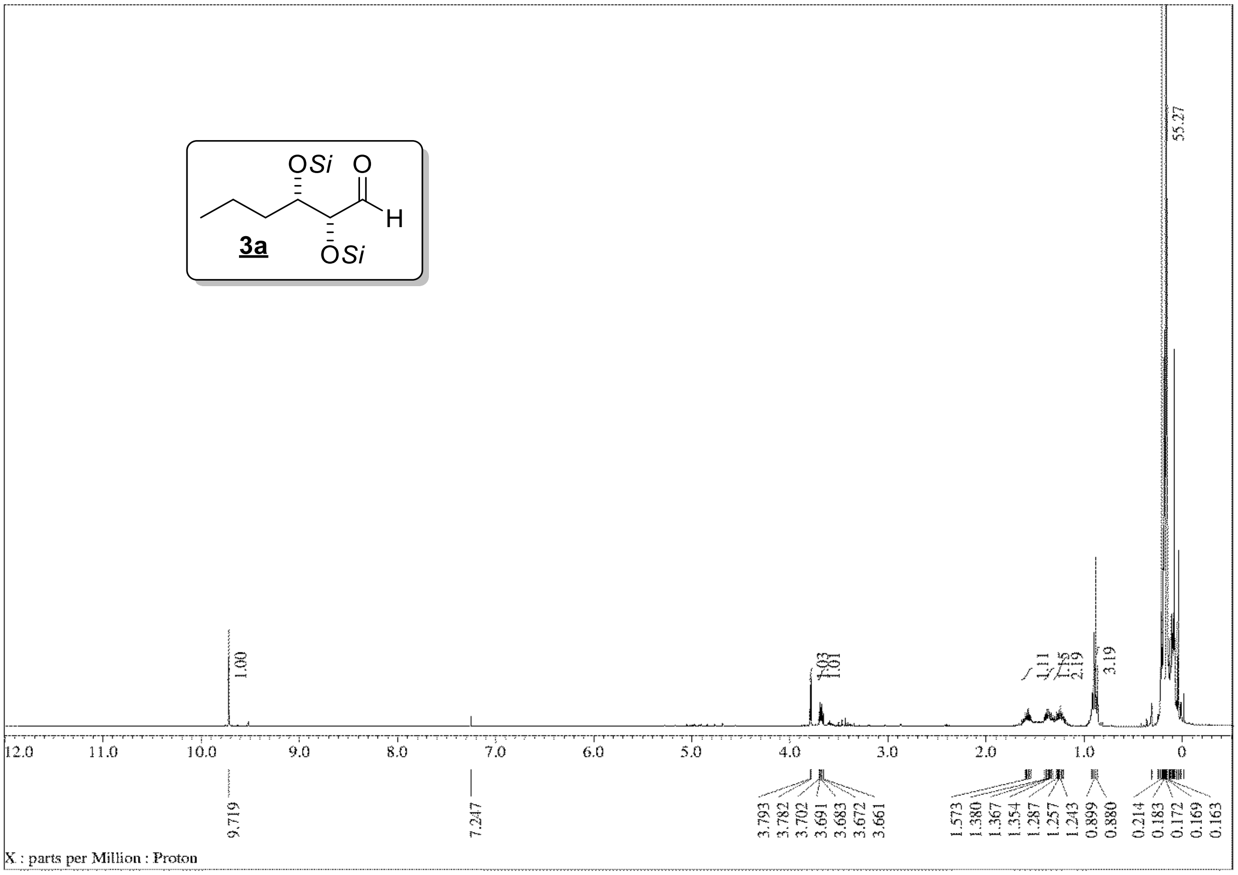
OSi
O
H
3a
OSi
X : parts per Million : Proton

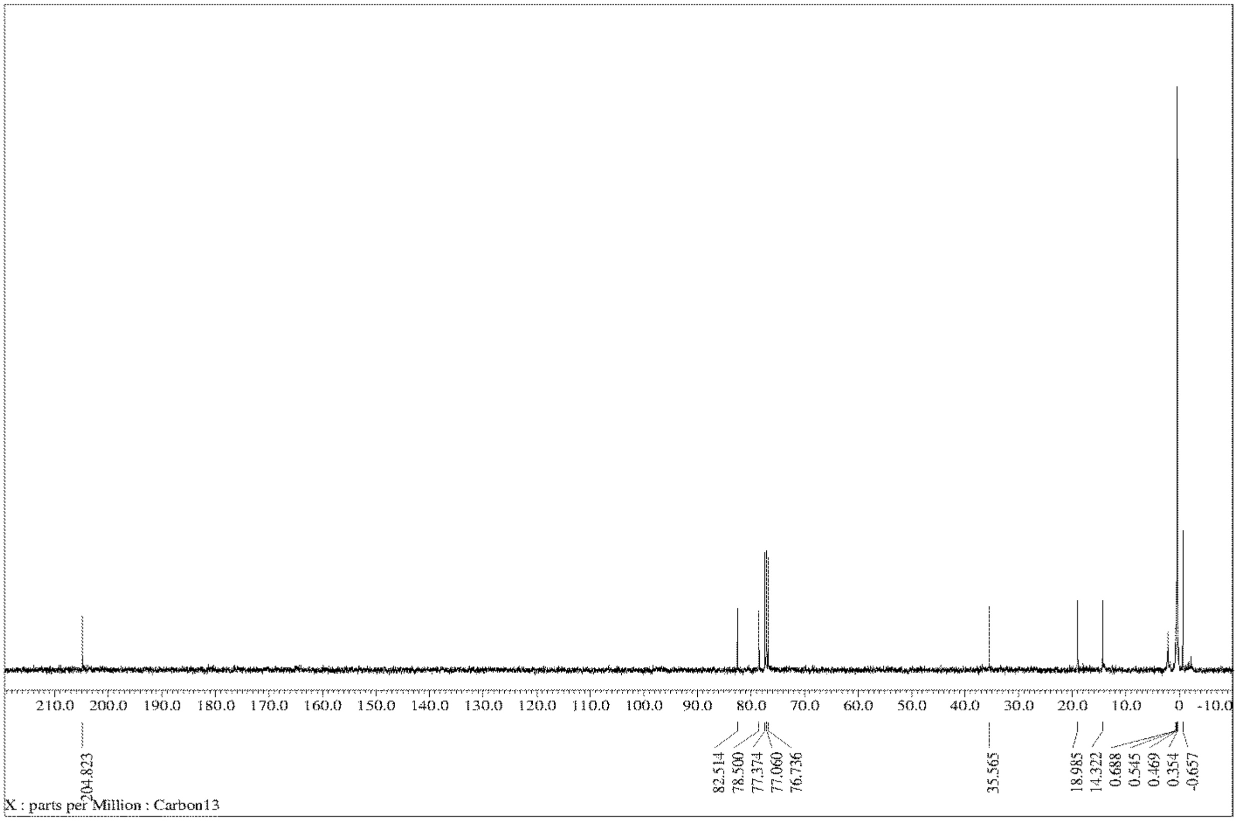
X : parts per Million : Carbon13

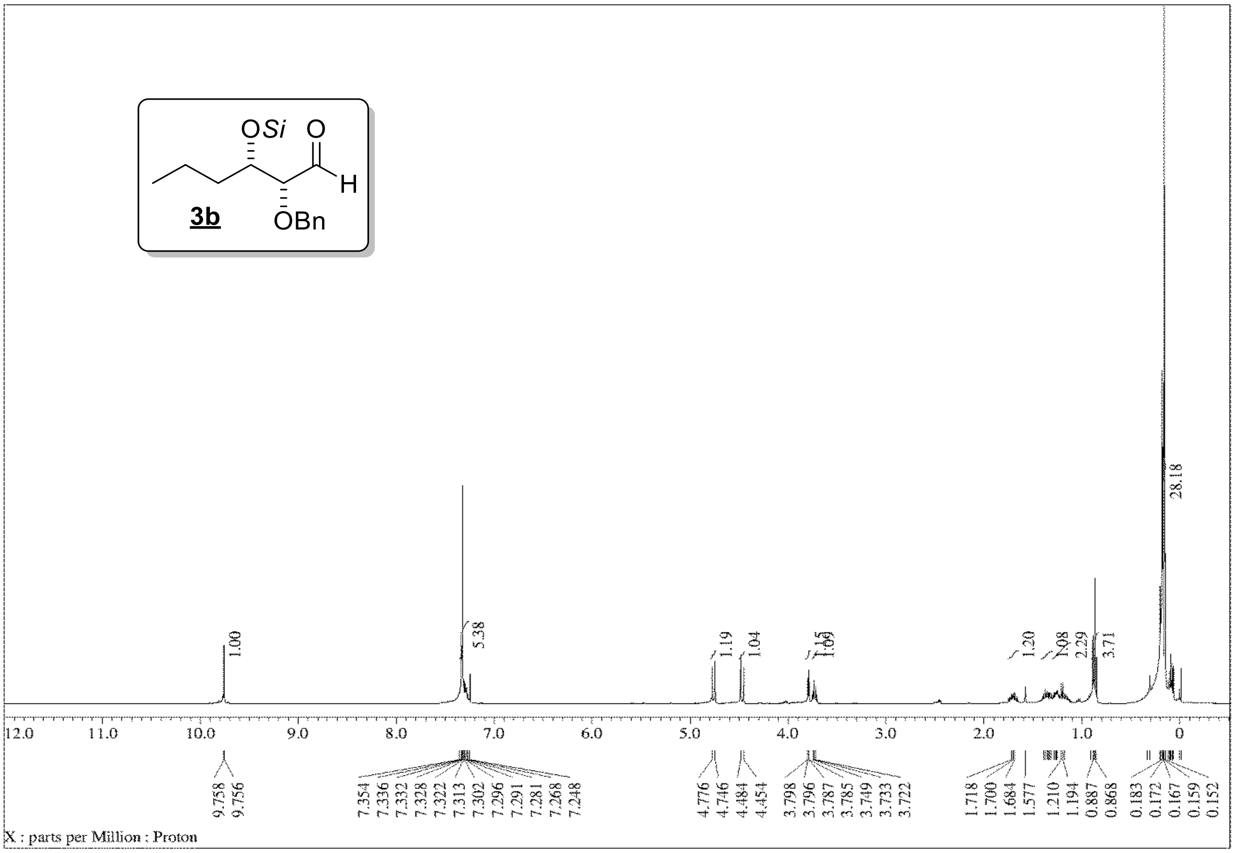
OSi
O
H
3b
OBn
1.00
5.38
1.19
1.04
1.15
1.09
1.20
1.08
2.29
3.71
28.18
12.0 11.0 10.0 9.0 8.0 7.0 6.0 5.0 4.0 3.0 2.0 1.0 0
9.758
9.756
7.354
7.336
7.332
7.328
7.322
7.313
7.302
7.296
7.291
7.281
7.268
7.248
4.776
4.746
4.484
4.454
3.798
3.796
3.787
3.785
3.749
3.733
3.722
1.718
1.700
1.684
1.577
1.210
1.194
0.887
0.868
0.183
0.172
0.167
0.159
0.152
X : parts per Million : Proton

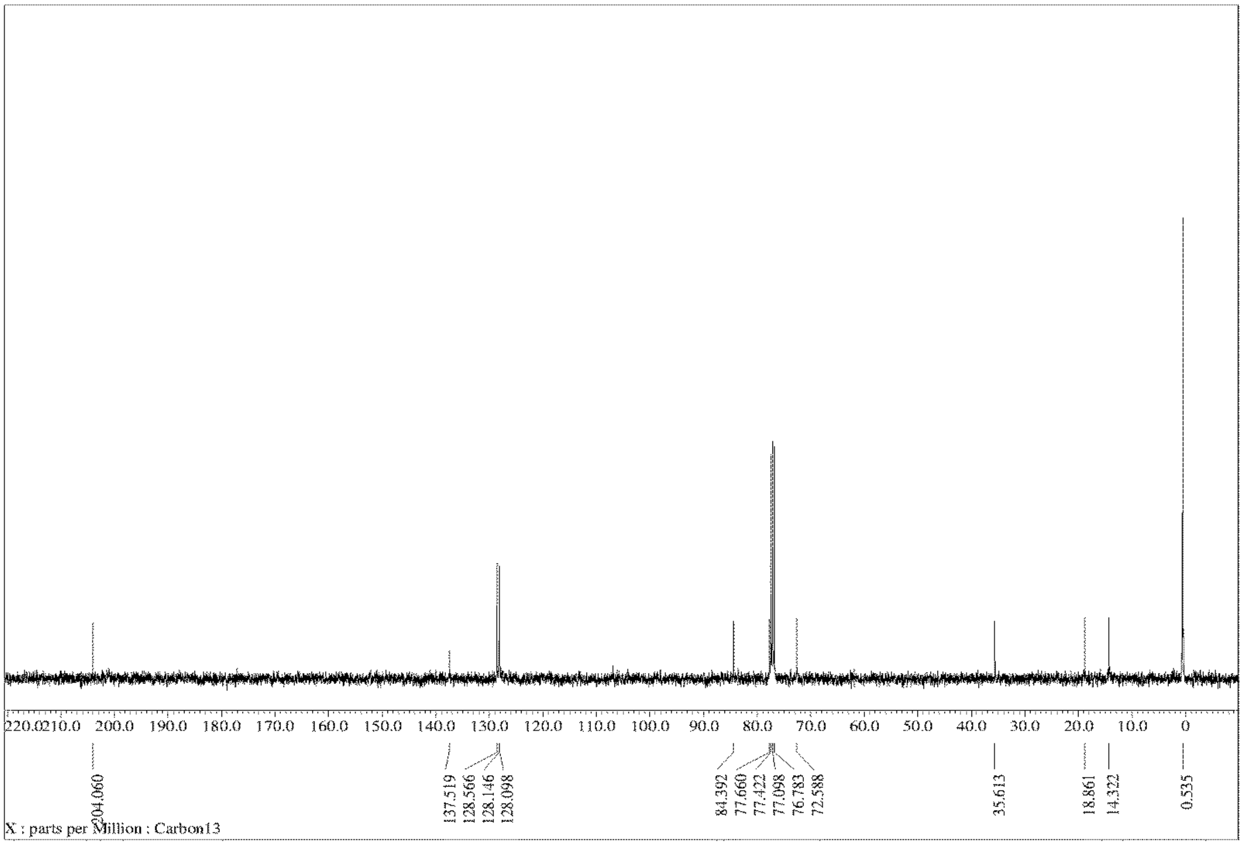
220.0 210.0 200.0 190.0 180.0 170.0 160.0 150.0 140.0 130.0 120.0 110.0 100.0 90.0 80.0 70.0 60.0 50.0 40.0 30.0 20.0 10.0 0
204.060
137.519
128.566
128.146
128.098
84.392
77.660
77.422
77.098
76.783
72.588
35.613
18.861
14.322
0.535
X : parts per Million : Carbon13

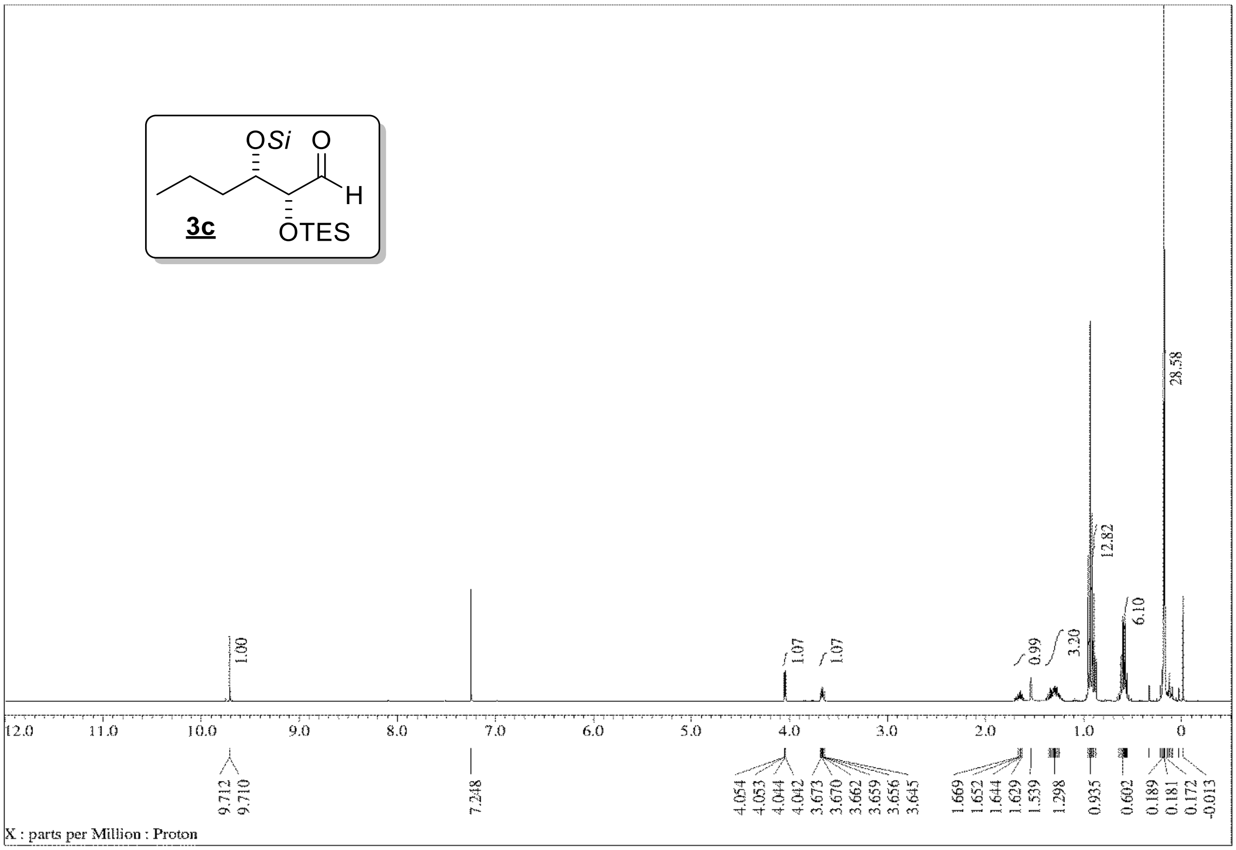
OSi
O
H
3c
OTES
1.00
1.07
1.07
0.99
3.20
12.82
6.10
28.58
12.0 11.0 10.0 9.0 8.0 7.0 6.0 5.0 4.0 3.0 2.0 1.0 0
9.712
9.710
7.248
4.054
4.053
4.044
4.042
3.673
3.670
3.662
3.659
3.656
3.645
1.669
1.652
1.644
1.629
1.539
1.298
0.935
0.602
0.189
0.181
0.172
-0.013
X : parts per Million : Proton

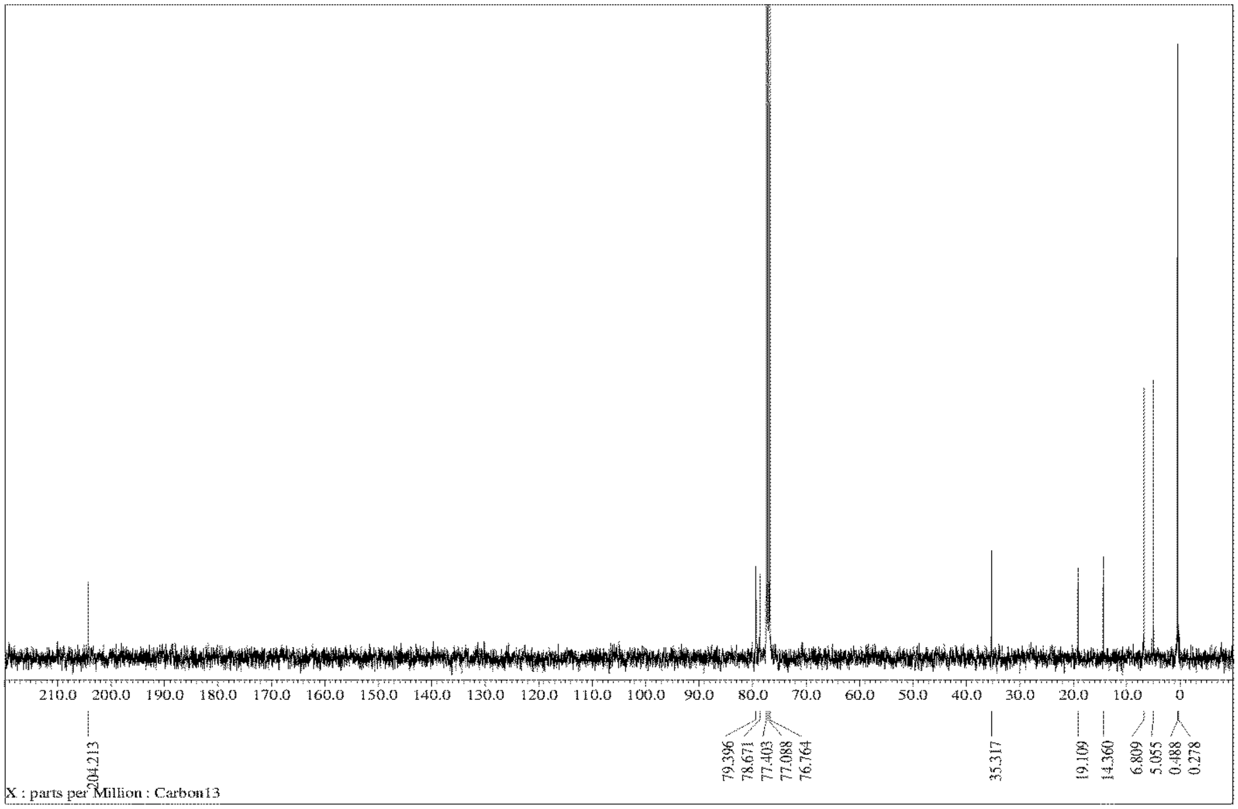
210.0 200.0 190.0 180.0 170.0 160.0 150.0 140.0 130.0 120.0 110.0 100.0 90.0 80.0 70.0 60.0 50.0 40.0 30.0 20.0 10.0 0
204.213
79.396
78.671
77.403
77.088
76.764
35.317
19.109
14.360
6.809
5.055
0.488
0.278
X : parts per Million : Carbon13

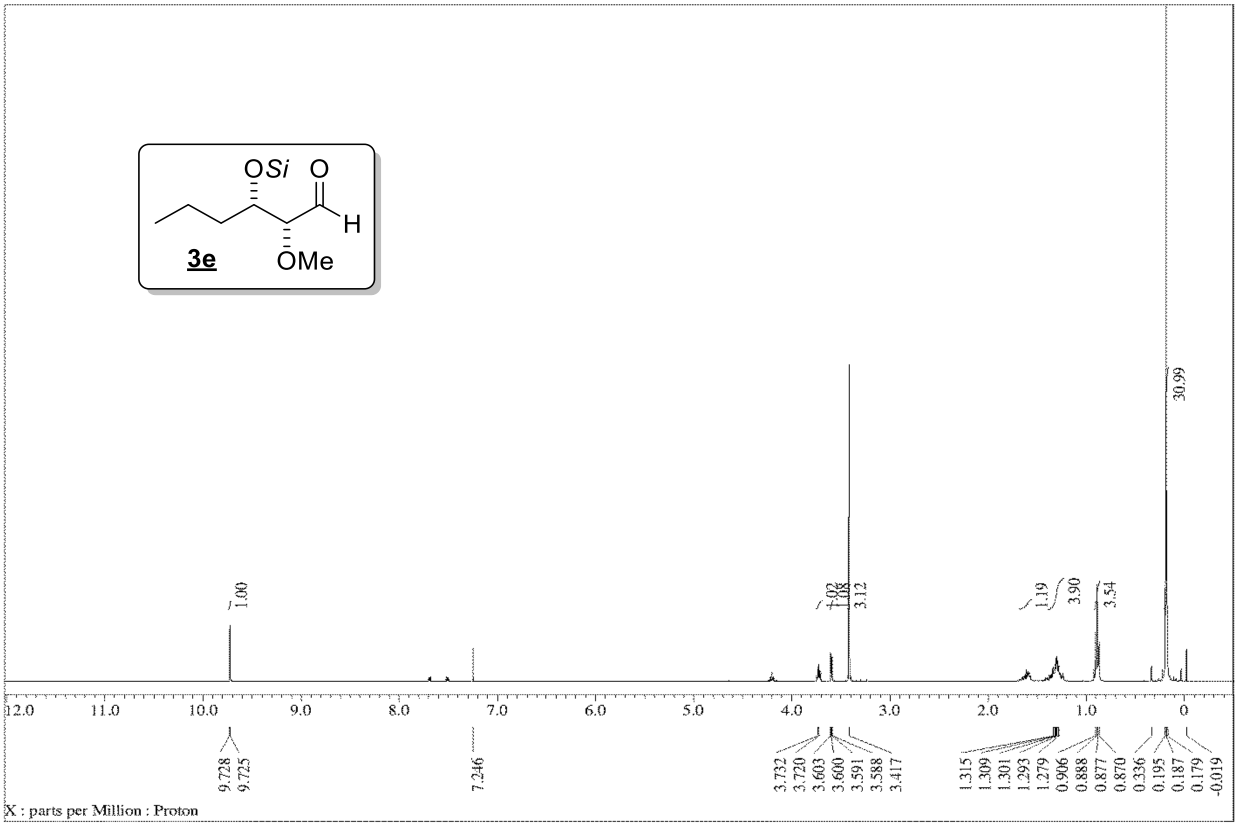
OSi O
H
3e
OMe
1.00
1.02
1.08
3.12
1.19
3.90
3.54
30.99
9.728
9.725
7.246
3.732
3.720
3.603
3.600
3.591
3.588
3.417
1.315
1.309
1.301
1.293
1.279
0.906
0.888
0.877
0.870
0.336
0.195
0.187
0.179
-0.019
X : parts per Million : Proton

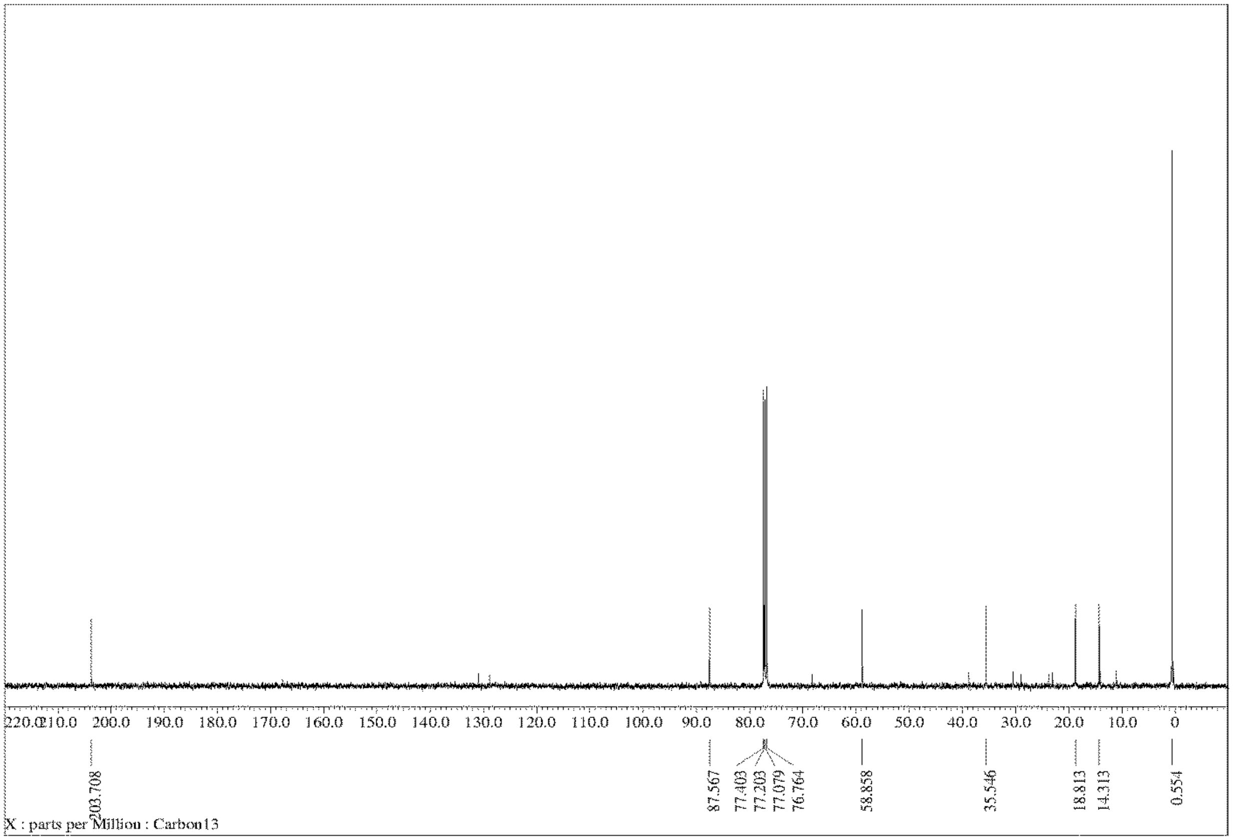
203.708
87.567
77.403
77.203
77.079
76.764
58.858
35.546
18.813
14.313
0.554
X : parts per Million : Carbon13

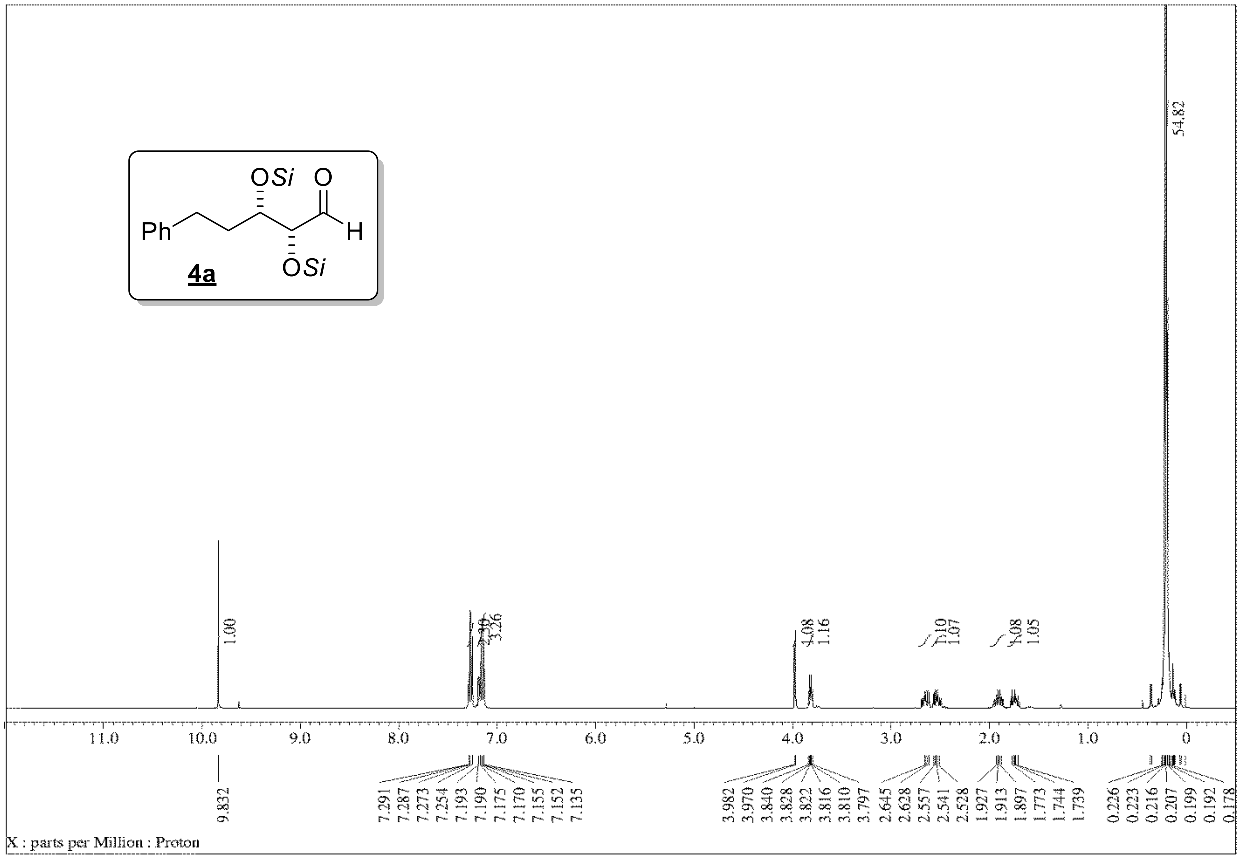

OSi
O
Ph
H
OSi
4a
54.82
1.00
3.26
1.08
1.16
1.10
1.07
1.08
1.05
9.832
7.291
7.287
7.273
7.254
7.193
7.190
7.175
7.170
7.155
7.152
7.135
3.982
3.970
3.840
3.828
3.822
3.816
3.810
3.797
2.645
2.628
2.557
2.541
2.528
1.927
1.913
1.897
1.773
1.744
1.739
0.226
0.223
0.216
0.207
0.199
0.192
0.178
X : parts per Million : Proton

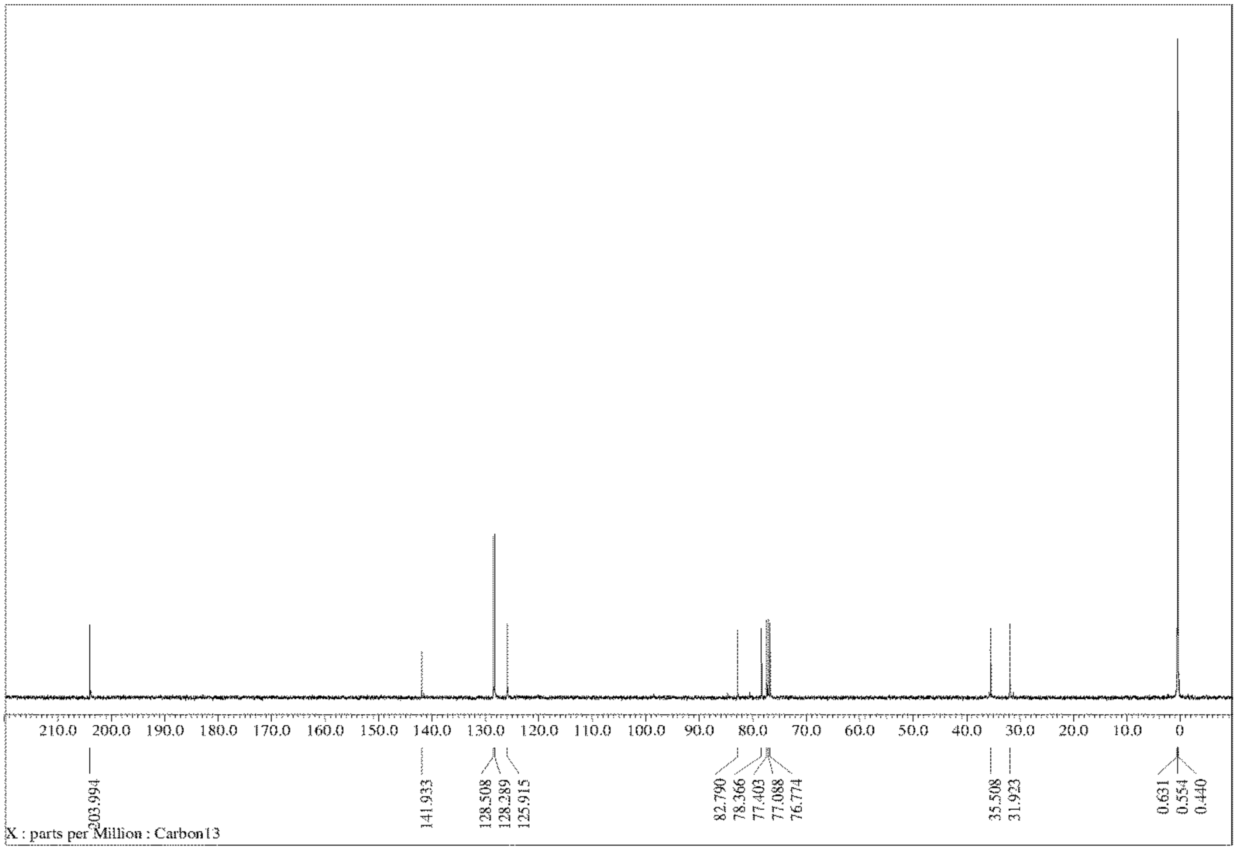

203.994
141.933
128.508
128.289
125.915
82.790
78.366
77.403
77.088
76.774
35.508
31.923
0.631
0.554
0.440
X : parts per Million : Carbon13

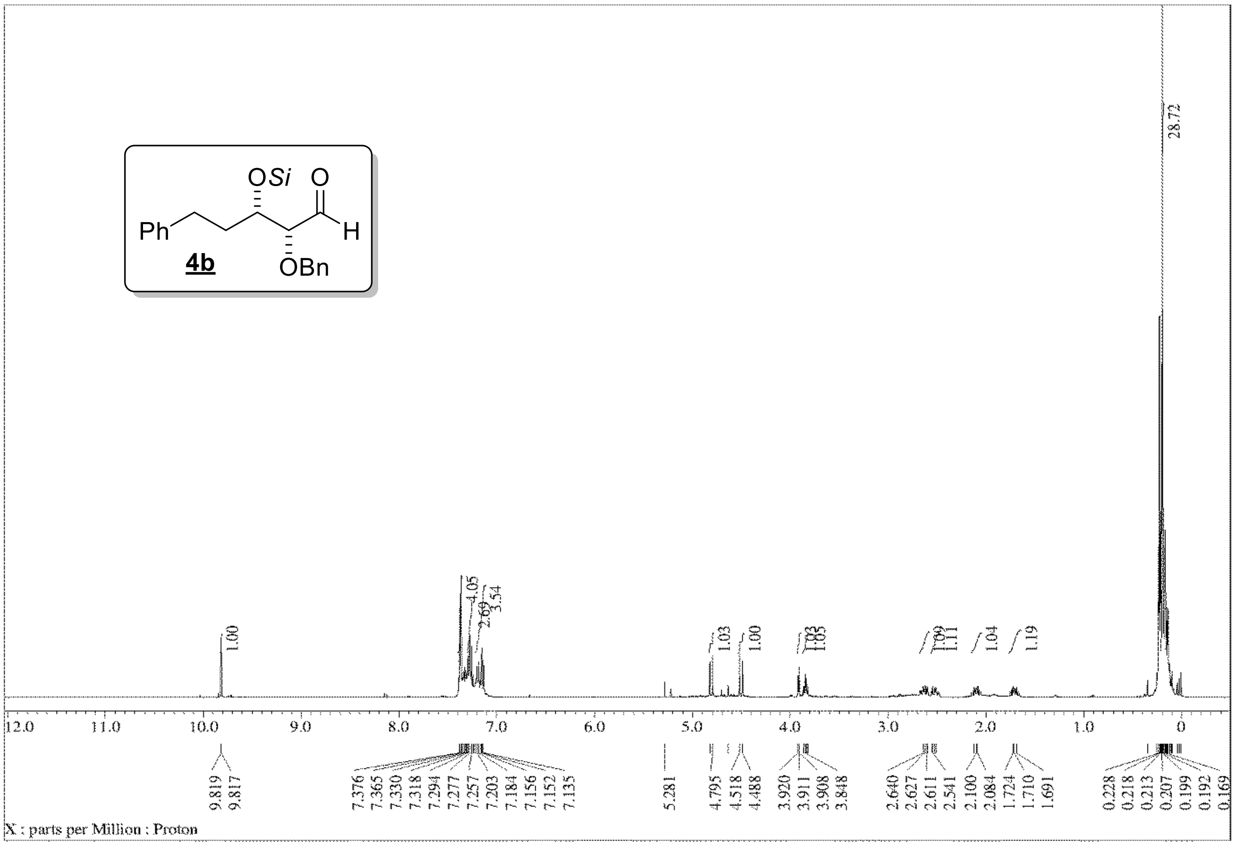
OSi O
Ph
H
4b
OBn
28.72
1.00
4.05
2.69
3.54
1.03
1.00
1.03
1.05
1.09
1.11
1.04
1.19
9.819
9.817
7.376
7.365
7.330
7.318
7.294
7.277
7.257
7.203
7.184
7.156
7.152
7.135
5.281
4.795
4.518
4.488
3.920
3.911
3.908
3.848
2.640
2.627
2.611
2.541
2.100
2.084
1.724
1.710
1.691
0.228
0.218
0.213
0.207
0.199
0.192
0.169
X : parts per Million : Proton

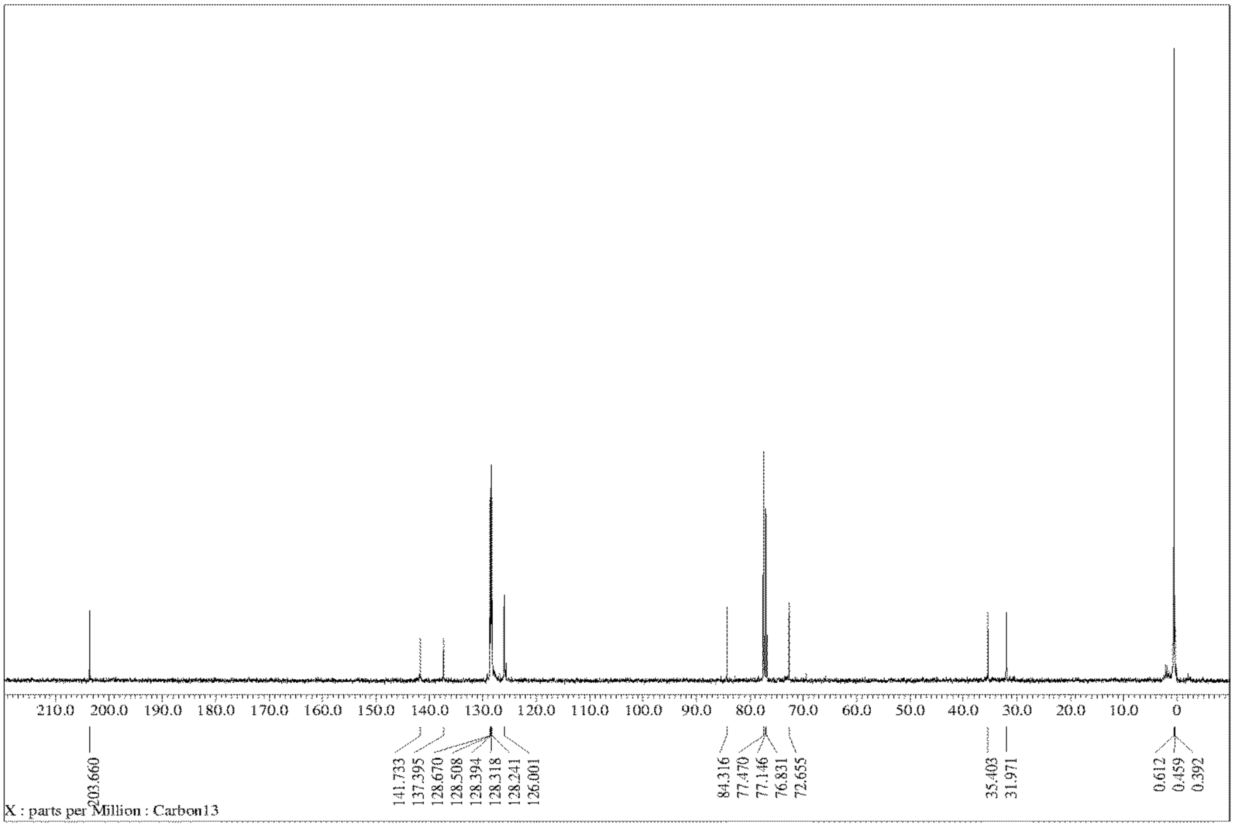
203.660
141.733
137.395
128.670
128.508
128.394
128.318
128.241
126.001
84.316
77.470
77.146
76.831
72.655
35.403
31.971
0.612
0.459
0.392
X : parts per Million : Carbon13

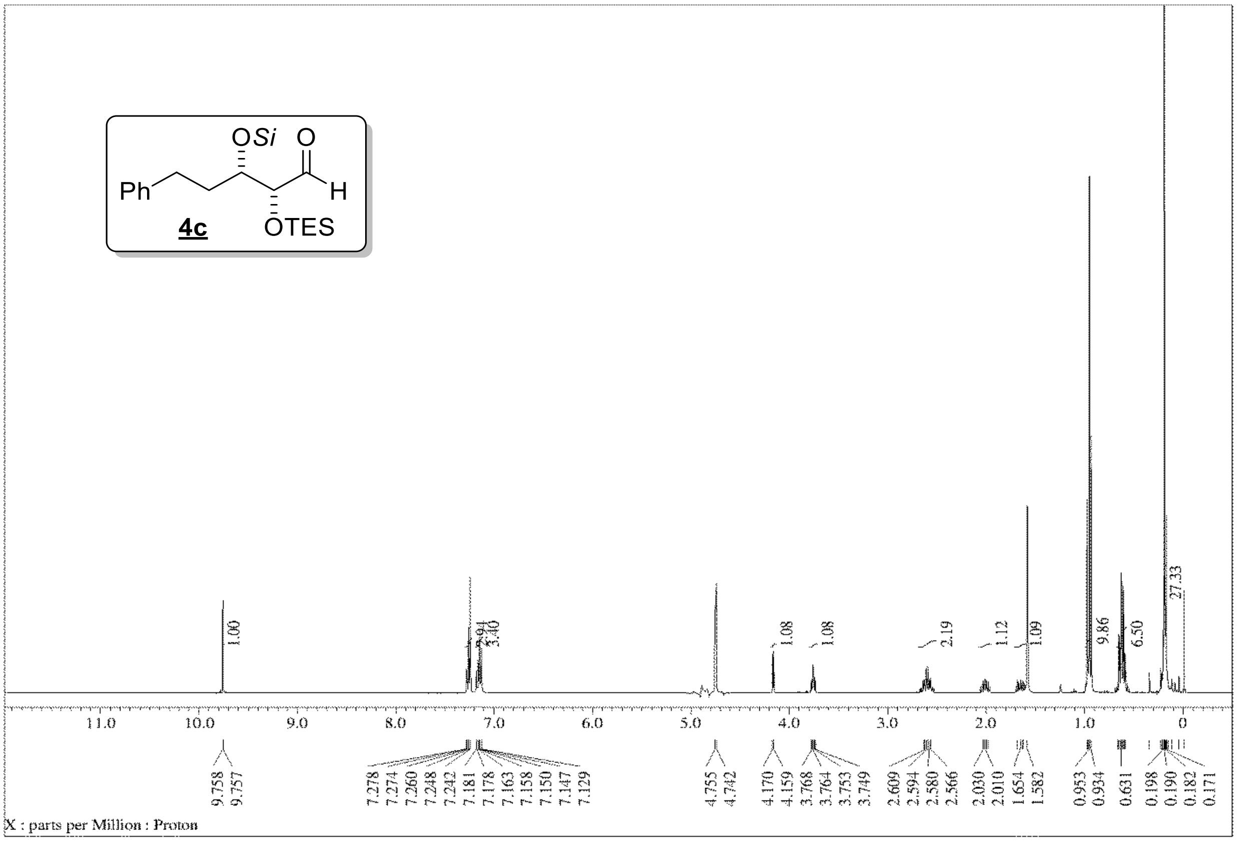

OSi O
Ph H
4c
OTES
1.00
2.94
3.40
1.08
1.08
2.19
1.12
1.09
9.86
6.50
27.33
11.0 10.0 9.0 8.0 7.0 6.0 5.0 4.0 3.0 2.0 1.0 0
9.758
9.757
7.278
7.274
7.260
7.248
7.242
7.181
7.178
7.163
7.158
7.150
7.147
7.129
4.755
4.742
4.170
4.159
3.768
3.764
3.753
3.749
2.609
2.594
2.580
2.566
2.030
2.010
1.654
1.582
0.953
0.934
0.631
0.198
0.190
0.182
0.171
X : parts per Million : Proton

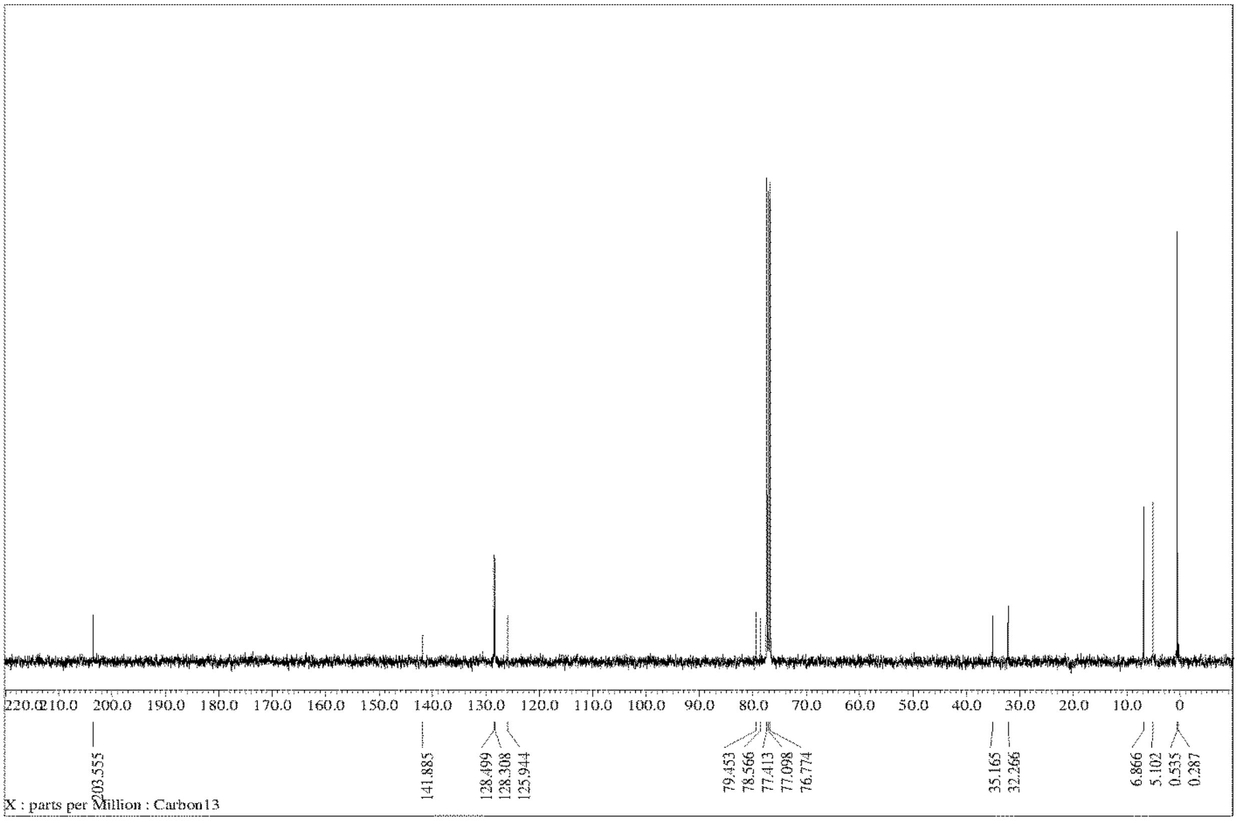

220.0 210.0 200.0 190.0 180.0 170.0 160.0 150.0 140.0 130.0 120.0 110.0 100.0 90.0 80.0 70.0 60.0 50.0 40.0 30.0 20.0 10.0 0
203.555
141.885
128.499
128.308
125.944
79.453
78.566
77.413
77.098
76.774
35.165
32.266
6.866
5.102
0.535
0.287
X : parts per Million : Carbon13

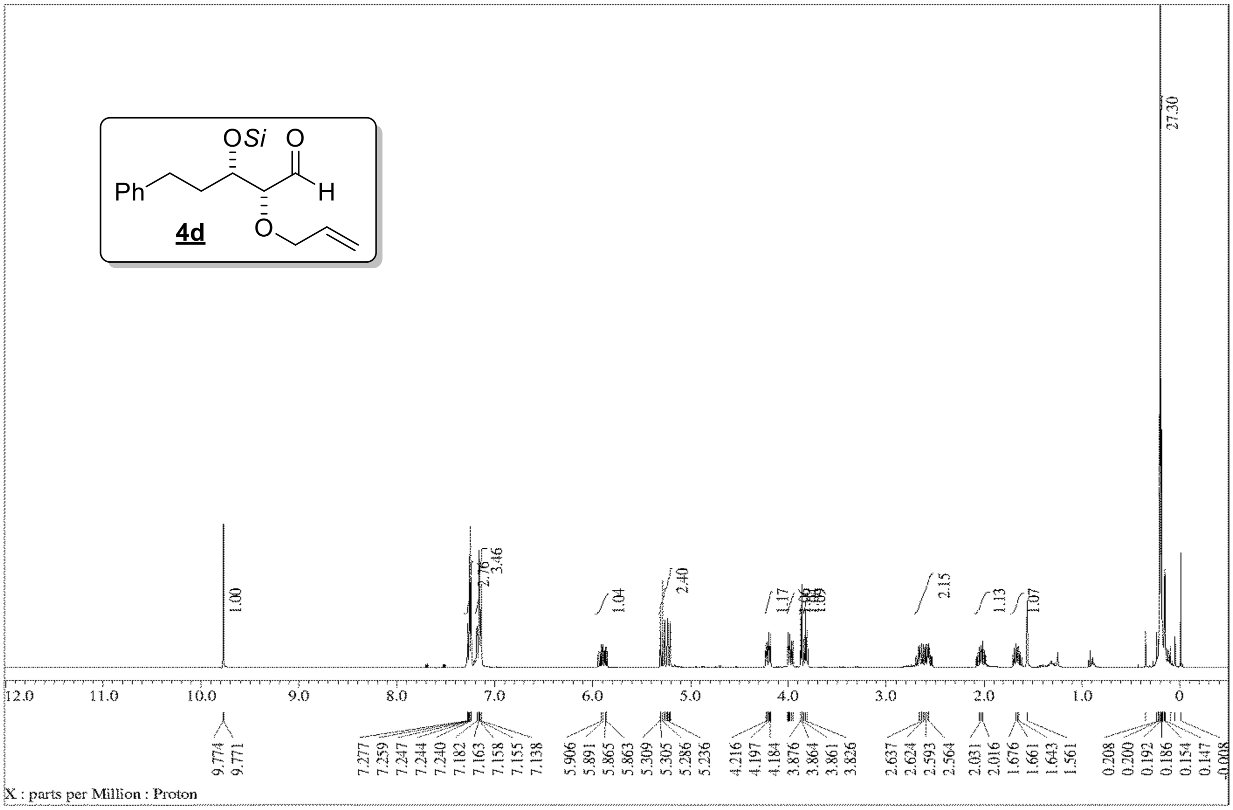
OSi
O
Ph
H
O
4d
1.00
2.76
3.46
1.04
2.40
1.17
2.15
1.13
1.07
27.30
12.0 11.0 10.0 9.0 8.0 7.0 6.0 5.0 4.0 3.0 2.0 1.0 0
9.774
9.771
7.277 7.259 7.247 7.244 7.240 7.182 7.163 7.158 7.155 7.138
5.906 5.891 5.865 5.863 5.309 5.305 5.286 5.236
4.216 4.197 4.184 3.876 3.864 3.861 3.826
2.637 2.624 2.593 2.564 2.031 2.016 1.676 1.661 1.643 1.561
0.208 0.200 0.192 0.186 0.154 0.147 -0.008
X : parts per Million : Proton

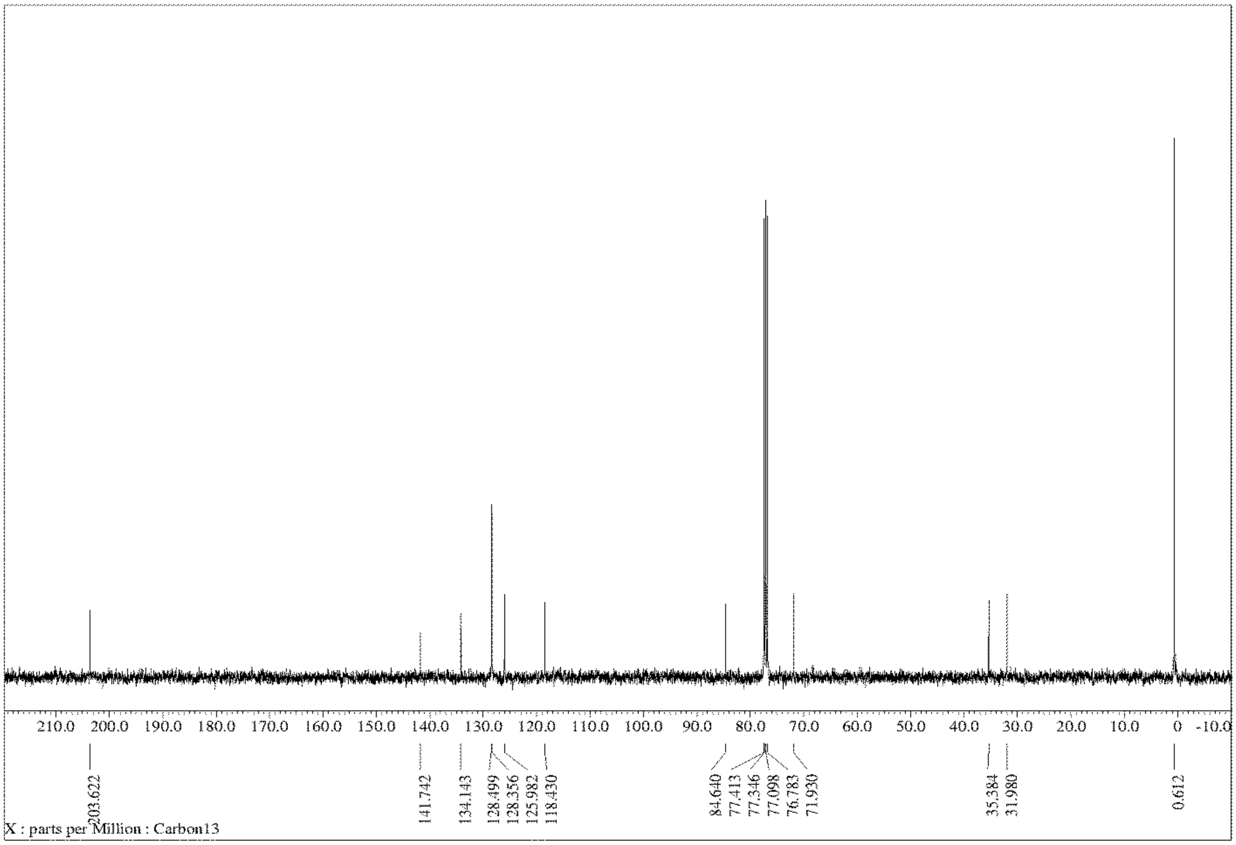
210.0 200.0 190.0 180.0 170.0 160.0 150.0 140.0 130.0 120.0 110.0 100.0 90.0 80.0 70.0 60.0 50.0 40.0 30.0 20.0 10.0 0 -10.0
203.622
141.742
134.143
128.499
128.356
125.982
118.430
84.640
77.413
77.346
77.098
76.783
71.930
35.384
31.980
0.612
X : parts per Million : Carbon13

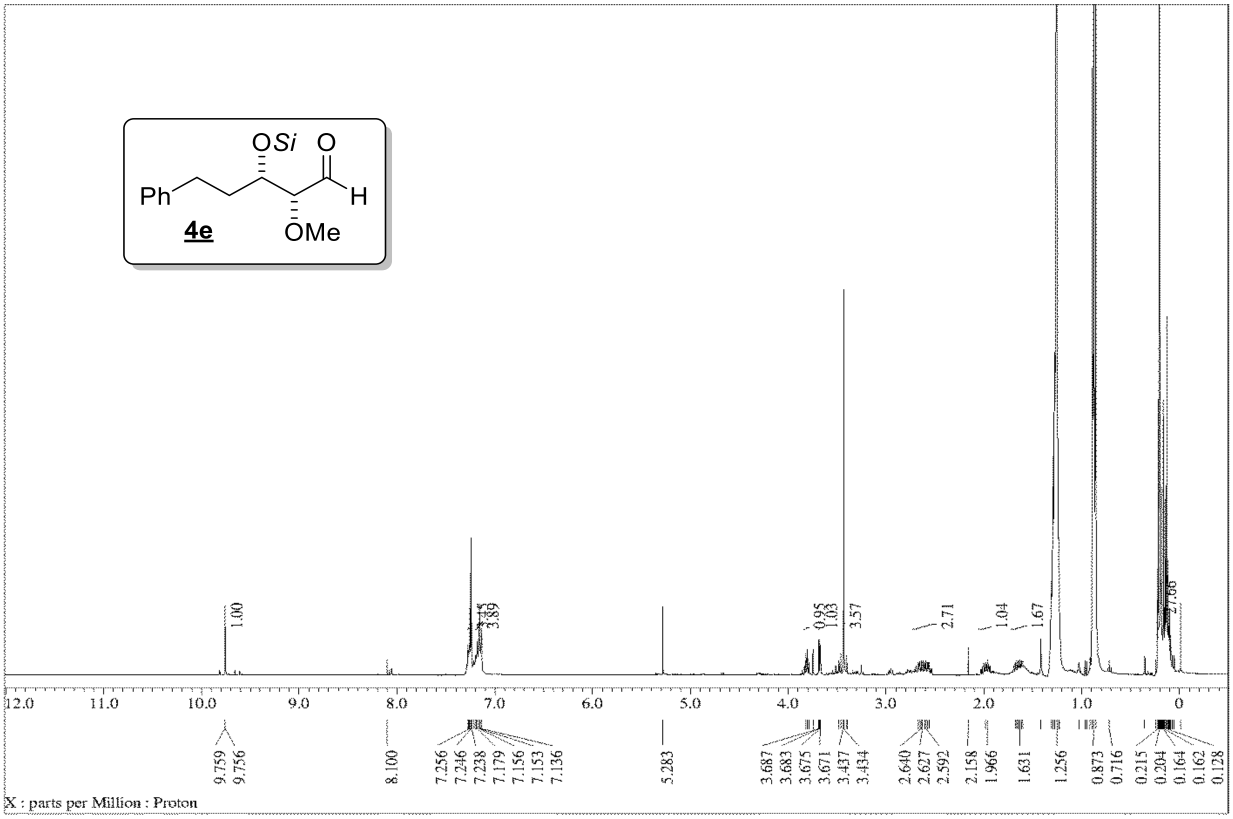
OSi O
Ph
H
4e
OMe
1.00
0.95
1.03
3.57
2.71
1.04
1.67
27.66
12.0 11.0 10.0 9.0 8.0 7.0 6.0 5.0 4.0 3.0 2.0 1.0 0
9.759
9.756
8.100
7.256
7.246
7.238
7.179
7.156
7.153
7.136
5.283
3.687
3.683
3.675
3.671
3.437
3.434
2.640
2.627
2.592
2.158
1.966
1.631
1.256
0.873
0.716
0.215
0.204
0.164
0.162
0.128
X : parts per Million : Proton

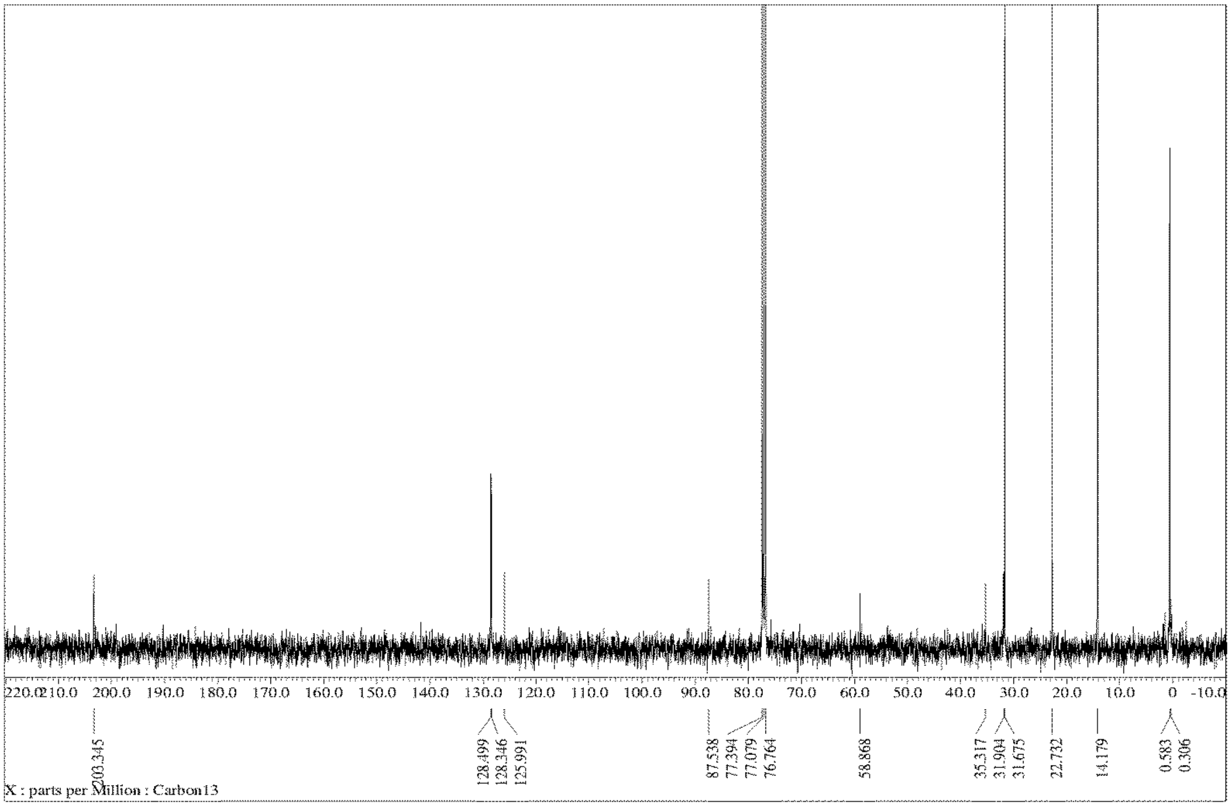
220.0 210.0 200.0 190.0 180.0 170.0 160.0 150.0 140.0 130.0 120.0 110.0 100.0 90.0 80.0 70.0 60.0 50.0 40.0 30.0 20.0 10.0 0 -10.0
203.345
128.499
128.346
125.991
87.538
77.394
77.079
76.764
58.868
35.317
31.904
31.675
22.732
14.179
0.583
0.306
X : parts per Million : Carbon13

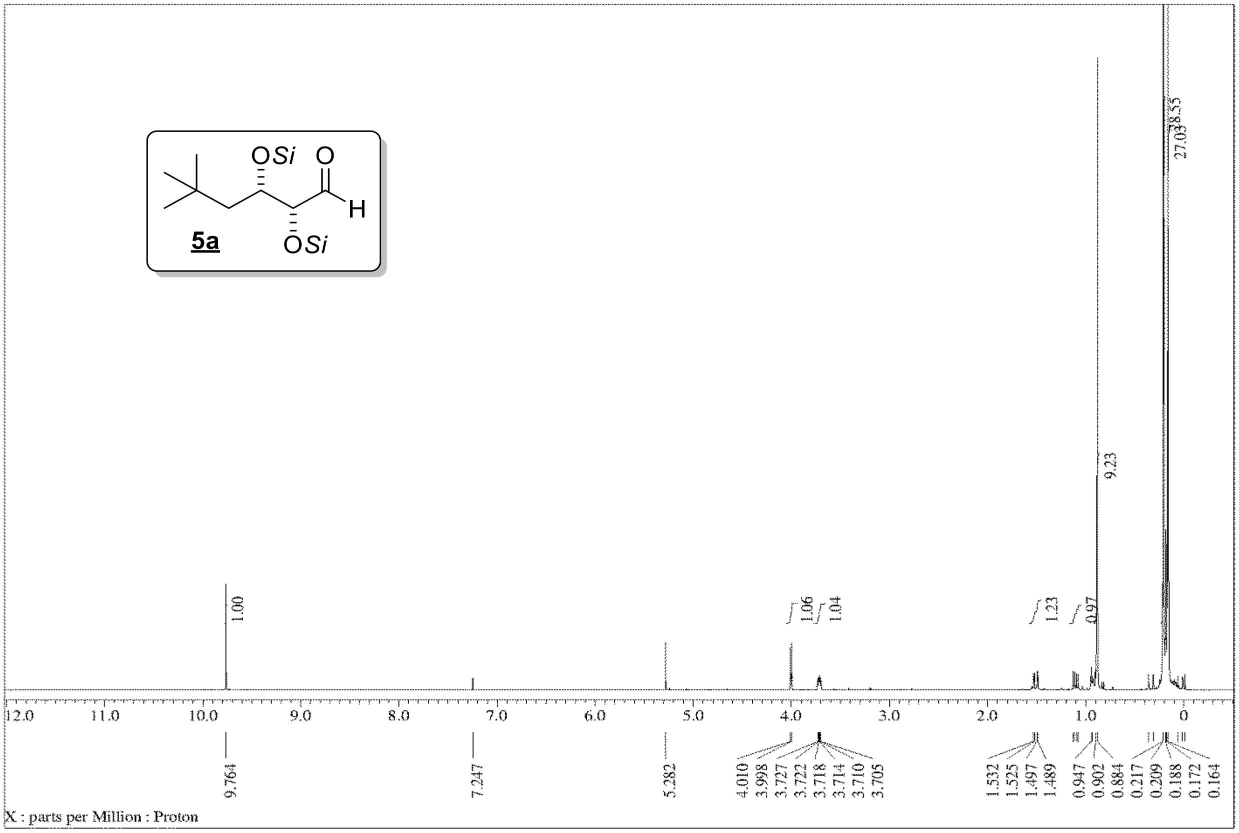

OSi O
H
OSi
5a
1.00
1.06
1.04
1.23
0.97
9.23
27.03
28.55
12.0 11.0 10.0 9.0 8.0 7.0 6.0 5.0 4.0 3.0 2.0 1.0 0
9.764
7.247
5.282
4.010 3.998 3.727 3.722 3.718 3.714 3.710 3.705
1.532 1.525 1.497 1.489
0.947 0.902 0.884 0.217 0.209 0.188 0.172 0.164
X : parts per Million : Proton

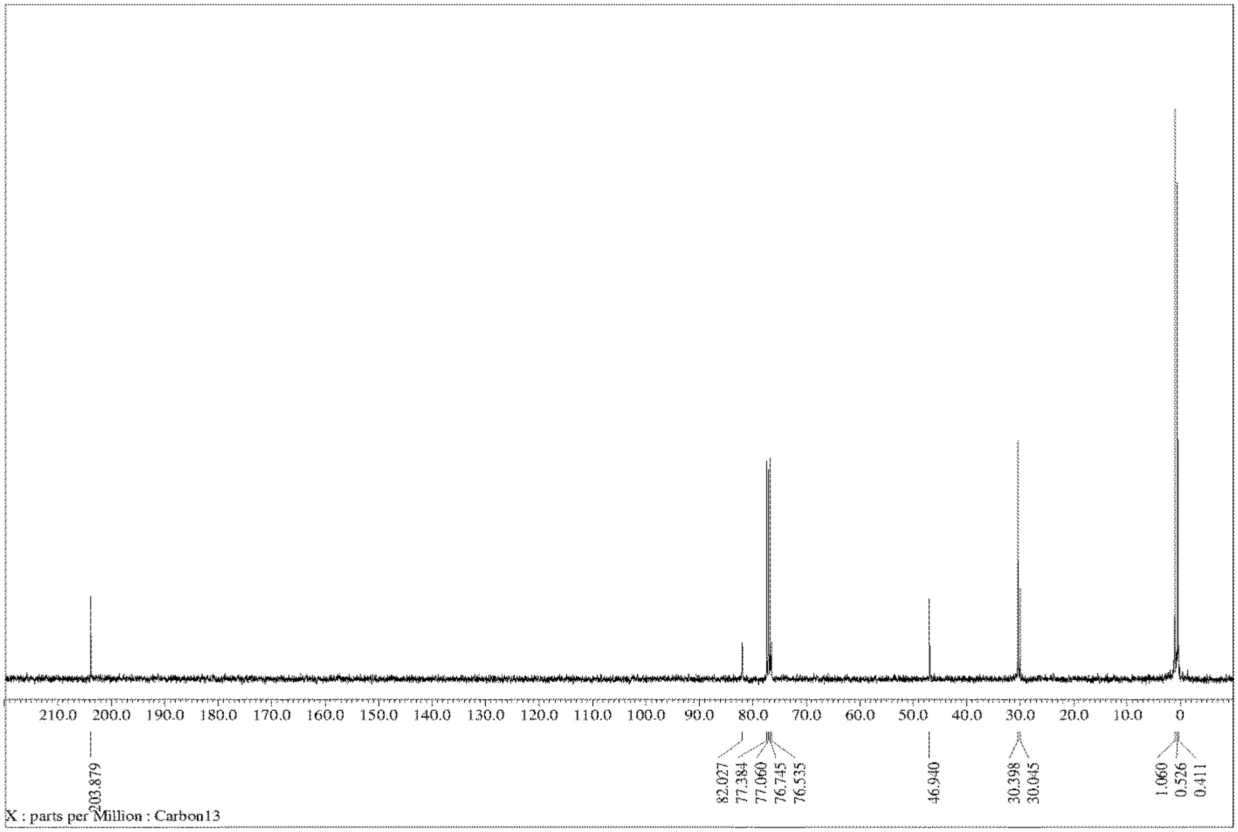

210.0 200.0 190.0 180.0 170.0 160.0 150.0 140.0 130.0 120.0 110.0 100.0 90.0 80.0 70.0 60.0 50.0 40.0 30.0 20.0 10.0 0
203.879
82.027 77.384 77.060 76.745 76.535
46.940
30.398 30.045
1.060 0.526 0.411
X : parts per Million : Carbon13

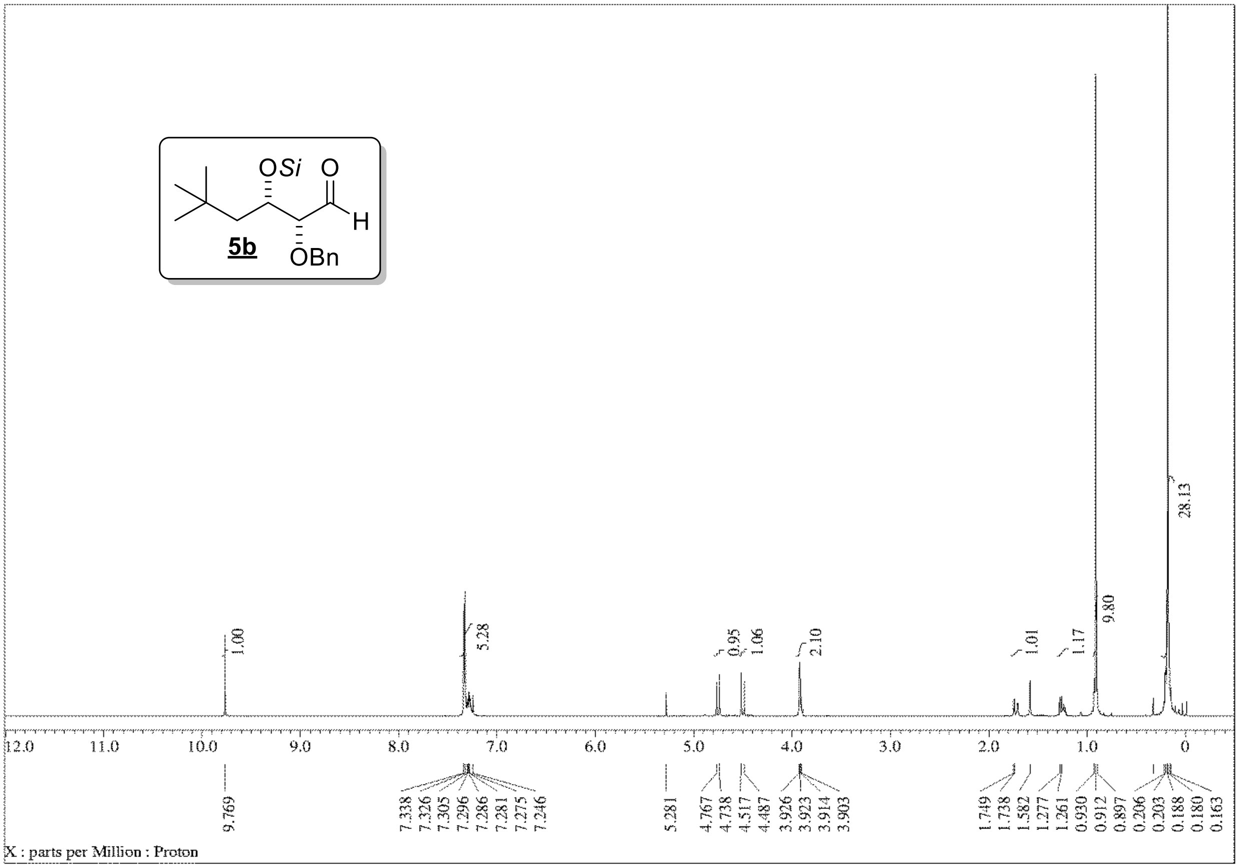
OSi
O
H
5b
OBn
1.00
5.28
0.95
1.06
2.10
1.01
1.17
9.80
28.13
9.769
7.338 7.326 7.305 7.296 7.286 7.281 7.275 7.246
5.281
4.767 4.738 4.517 4.487 3.926 3.923 3.914 3.903
1.749 1.738 1.582 1.277 1.261 0.930 0.912 0.897 0.206 0.203 0.188 0.180 0.163
X : parts per Million : Proton

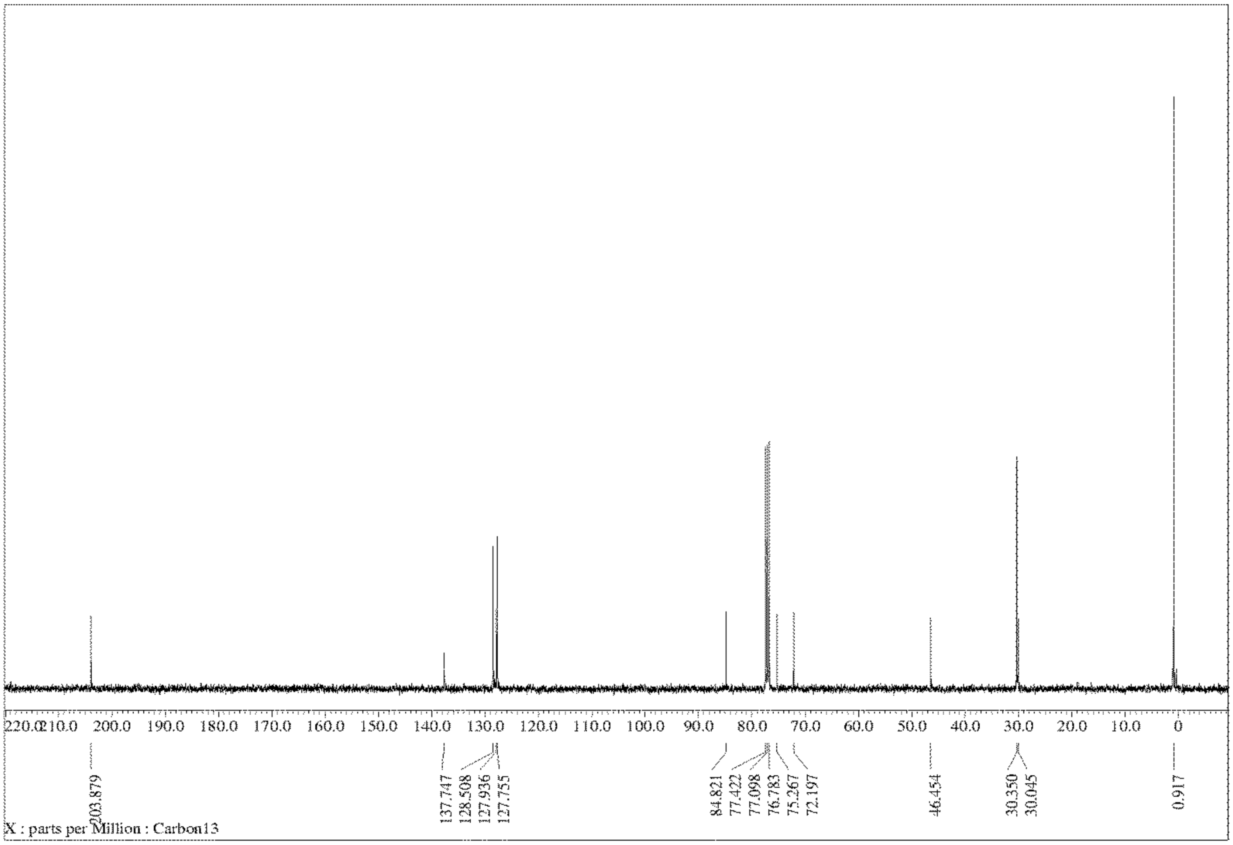
203.879
137.747 128.508 127.936 127.755
84.821 77.422 77.098 76.783 75.267 72.197
46.454
30.350 30.045
0.917
X : parts per Million : Carbon13

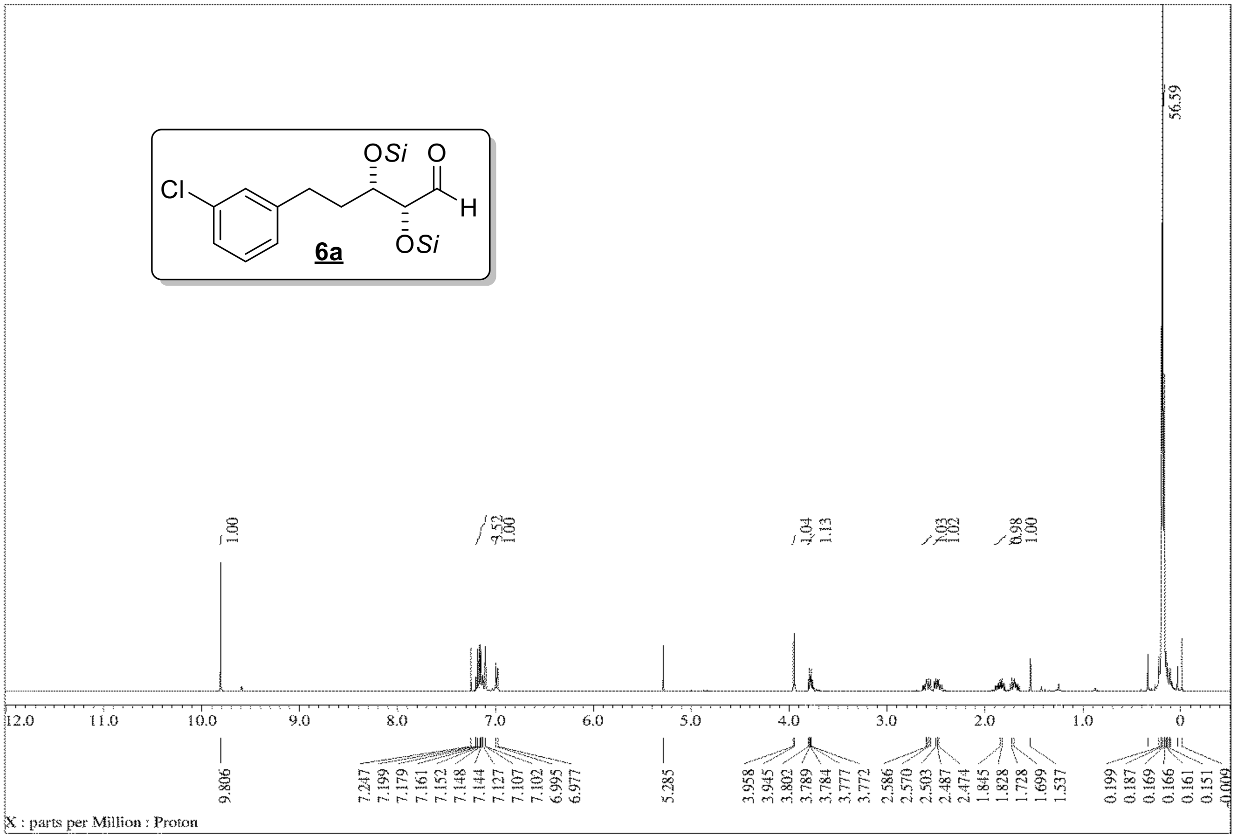
Cl
OSi
O
H
OSi
6a
56.59
1.00
3.52
1.00
1.04
1.13
1.03
1.02
0.98
1.00
12.0 11.0 10.0 9.0 8.0 7.0 6.0 5.0 4.0 3.0 2.0 1.0 0
9.806
7.247
7.199
7.179
7.161
7.152
7.148
7.144
7.127
7.107
7.102
6.995
6.977
5.285
3.958
3.945
3.802
3.789
3.784
3.777
3.772
2.586
2.570
2.503
2.487
2.474
1.845
1.828
1.728
1.699
1.537
0.199
0.187
0.169
0.166
0.161
0.151
0.009
X : parts per Million : Proton

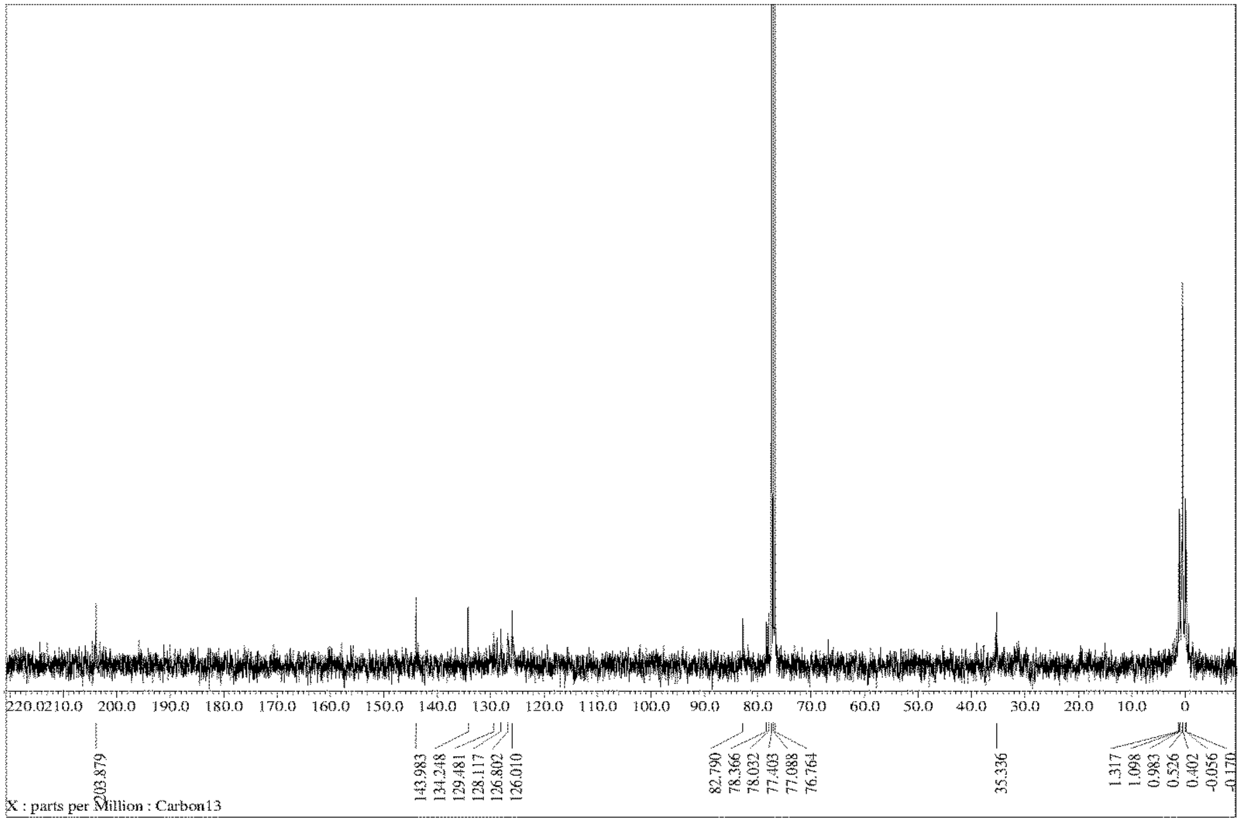
220.0 210.0 200.0 190.0 180.0 170.0 160.0 150.0 140.0 130.0 120.0 110.0 100.0 90.0 80.0 70.0 60.0 50.0 40.0 30.0 20.0 10.0 0
203.879
143.983
134.248
129.481
128.117
126.802
126.010
82.790
78.366
78.032
77.403
77.088
76.764
35.336
1.317
1.098
0.983
0.526
0.402
-0.056
-0.170
X : parts per Million : Carbon13

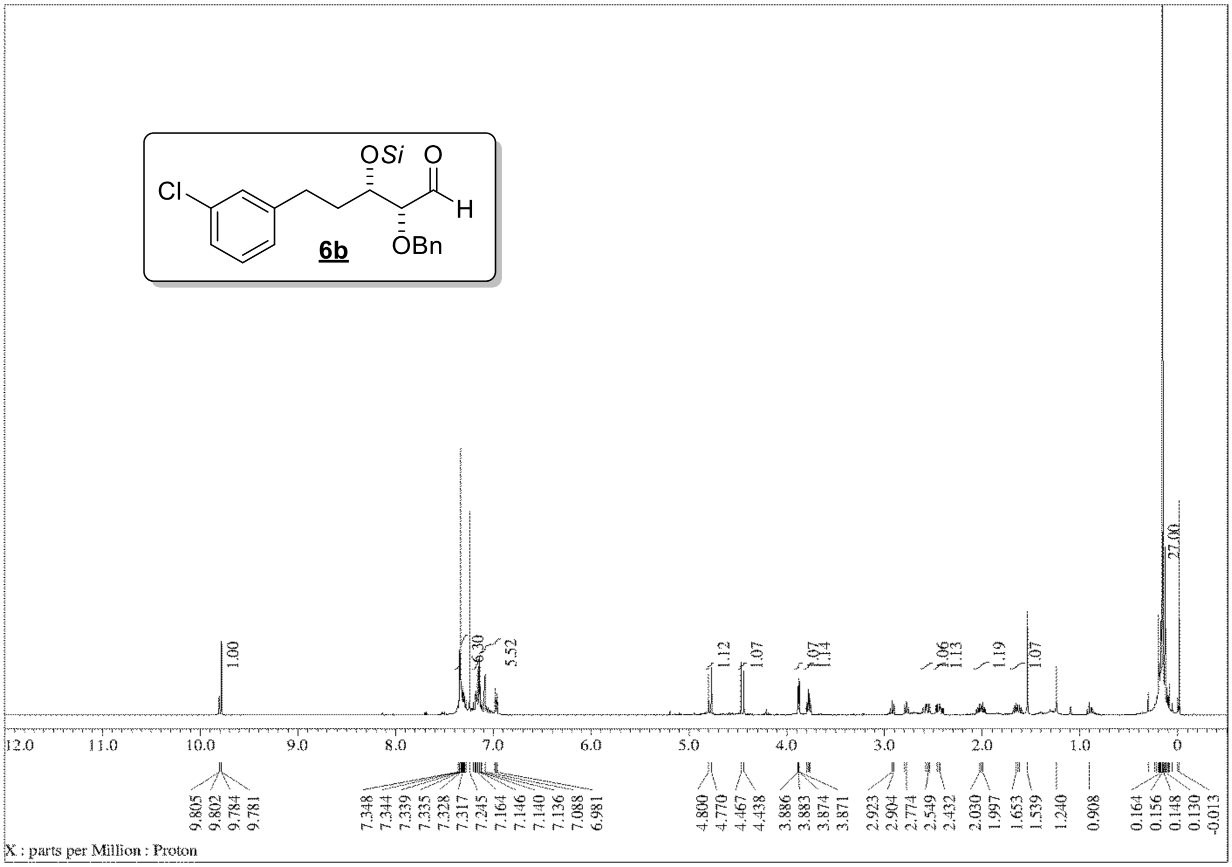
OSi
O
Cl
H
6b
OBn
X : parts per Million : Proton

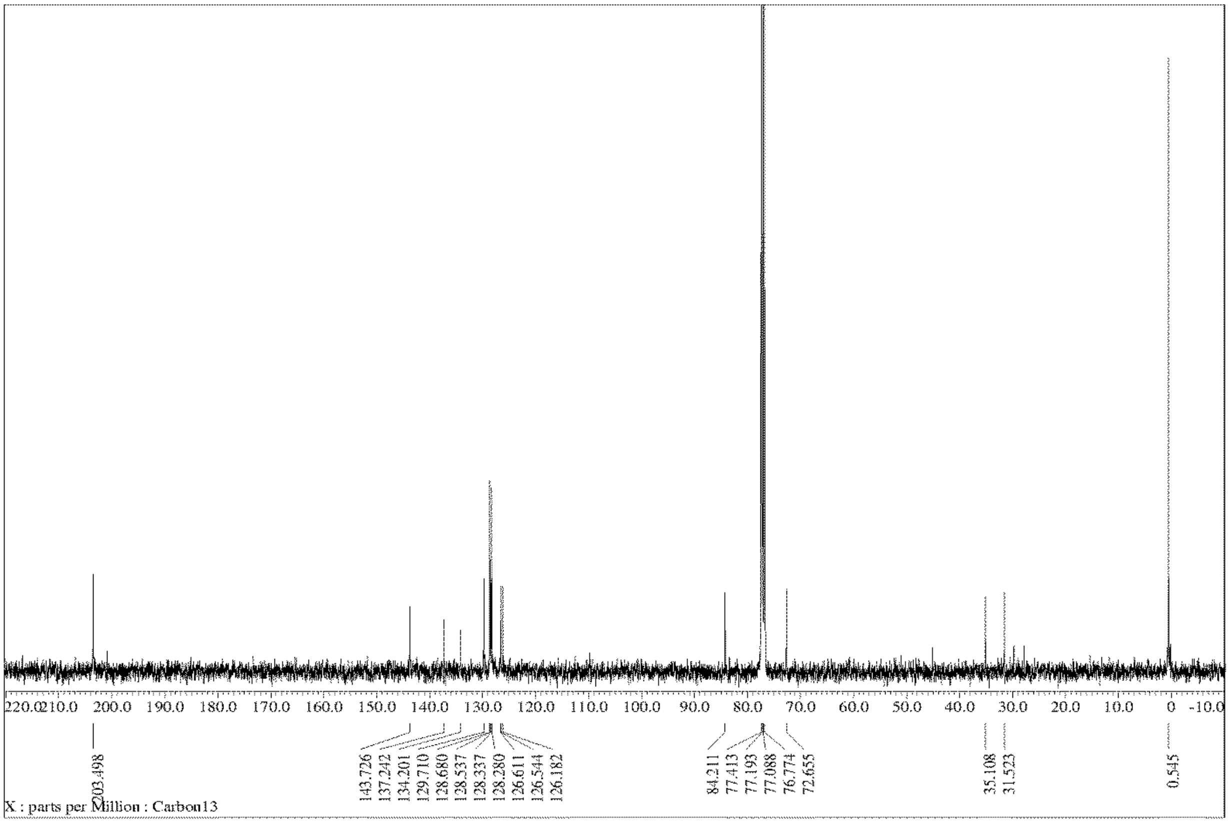
X : parts per Million : Carbon13

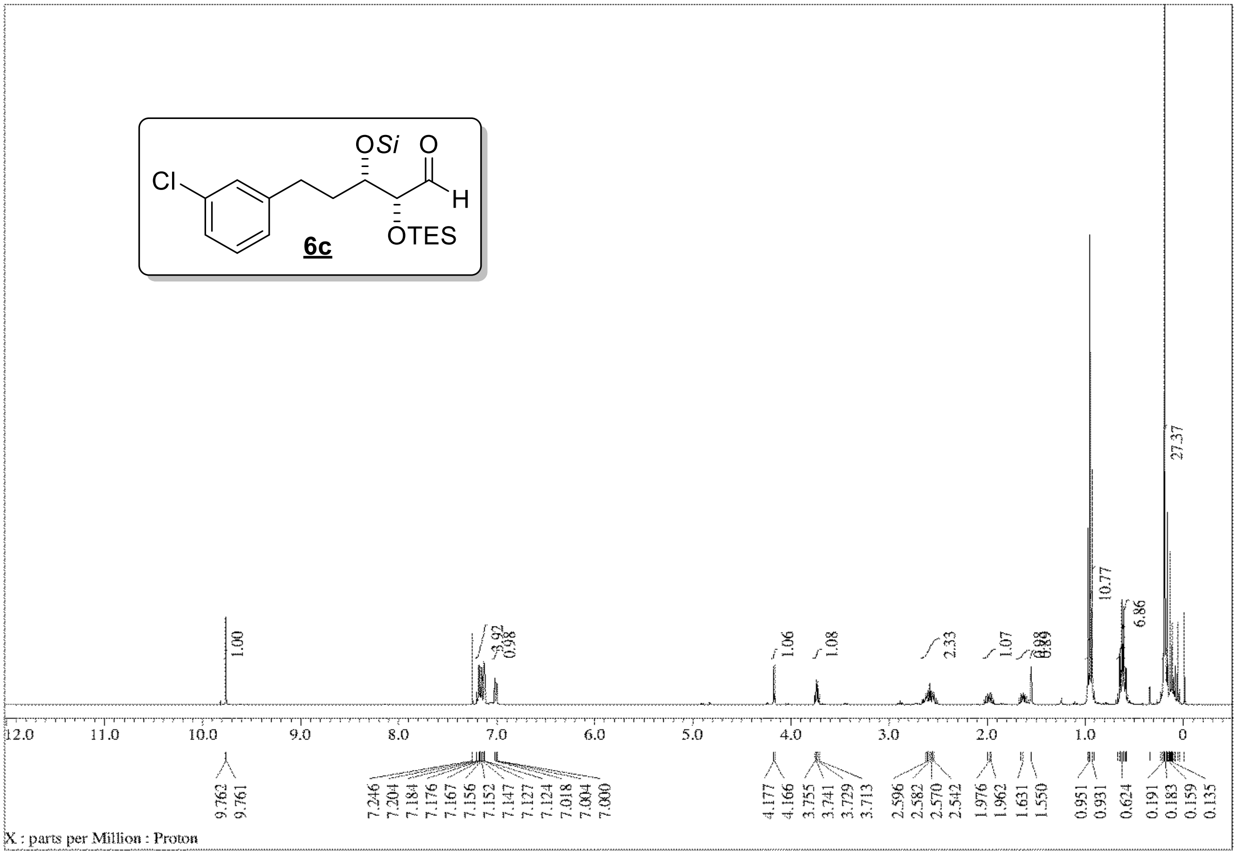
Cl
OSi
O
H
OTES
6c
1.00
3.92
0.98
1.06
1.08
2.33
1.07
10.77
6.86
27.37
9.762
9.761
7.246
7.204
7.184
7.176
7.167
7.156
7.152
7.147
7.127
7.124
7.018
7.004
7.000
4.177
4.166
3.755
3.741
3.729
3.713
2.596
2.582
2.570
2.542
1.976
1.962
1.631
1.550
0.951
0.931
0.624
0.191
0.183
0.159
0.135
X : parts per Million : Proton

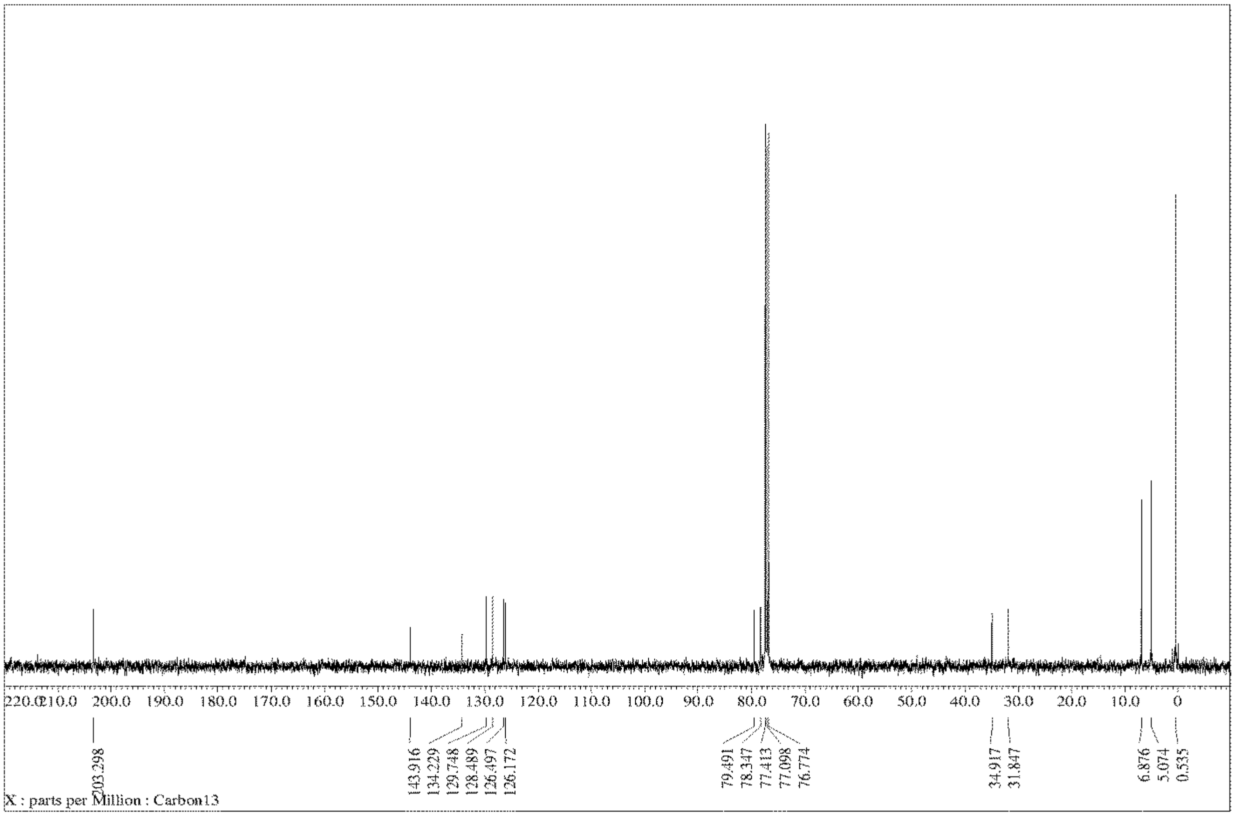
203.298
143.916
134.229
129.748
128.489
126.497
126.172
79.491
78.347
77.413
77.098
76.774
34.917
31.847
6.876
5.074
0.535
X : parts per Million : Carbon13

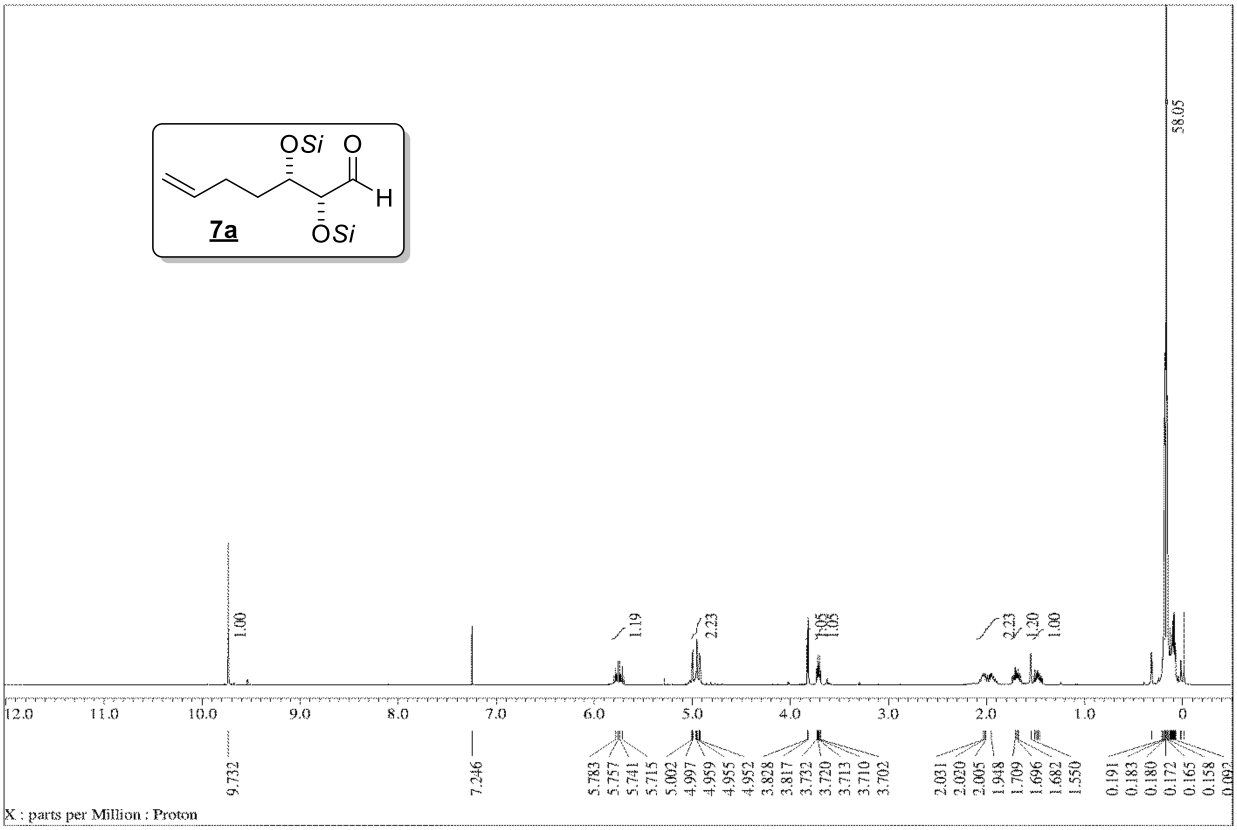
OSi
O
H
7a
OSi
58.05
1.00
1.19
2.23
1.05
1.05
2.23
1.20
1.00
12.0 11.0 10.0 9.0 8.0 7.0 6.0 5.0 4.0 3.0 2.0 1.0 0
9.732
7.246
5.783
5.757
5.741
5.715
5.002
4.997
4.959
4.955
4.952
3.828
3.817
3.732
3.720
3.713
3.710
3.702
2.031
2.020
2.005
1.948
1.709
1.696
1.682
1.550
0.191
0.183
0.180
0.172
0.165
0.158
0.092
X : parts per Million : Proton

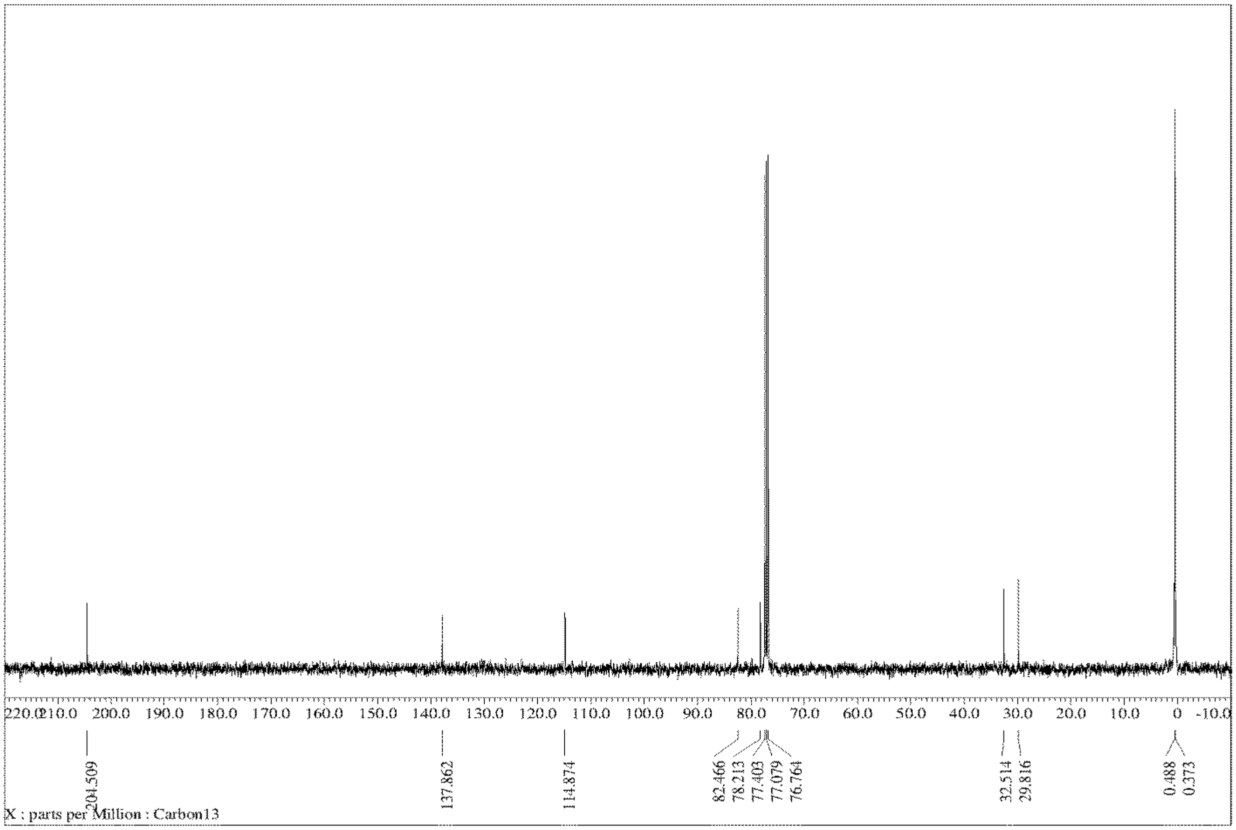
220.0 210.0 200.0 190.0 180.0 170.0 160.0 150.0 140.0 130.0 120.0 110.0 100.0 90.0 80.0 70.0 60.0 50.0 40.0 30.0 20.0 10.0 0 -10.0
204.509
137.862
114.874
82.466
78.213
77.403
77.079
76.764
32.514
29.816
0.488
0.373
X : parts per Million : Carbon13

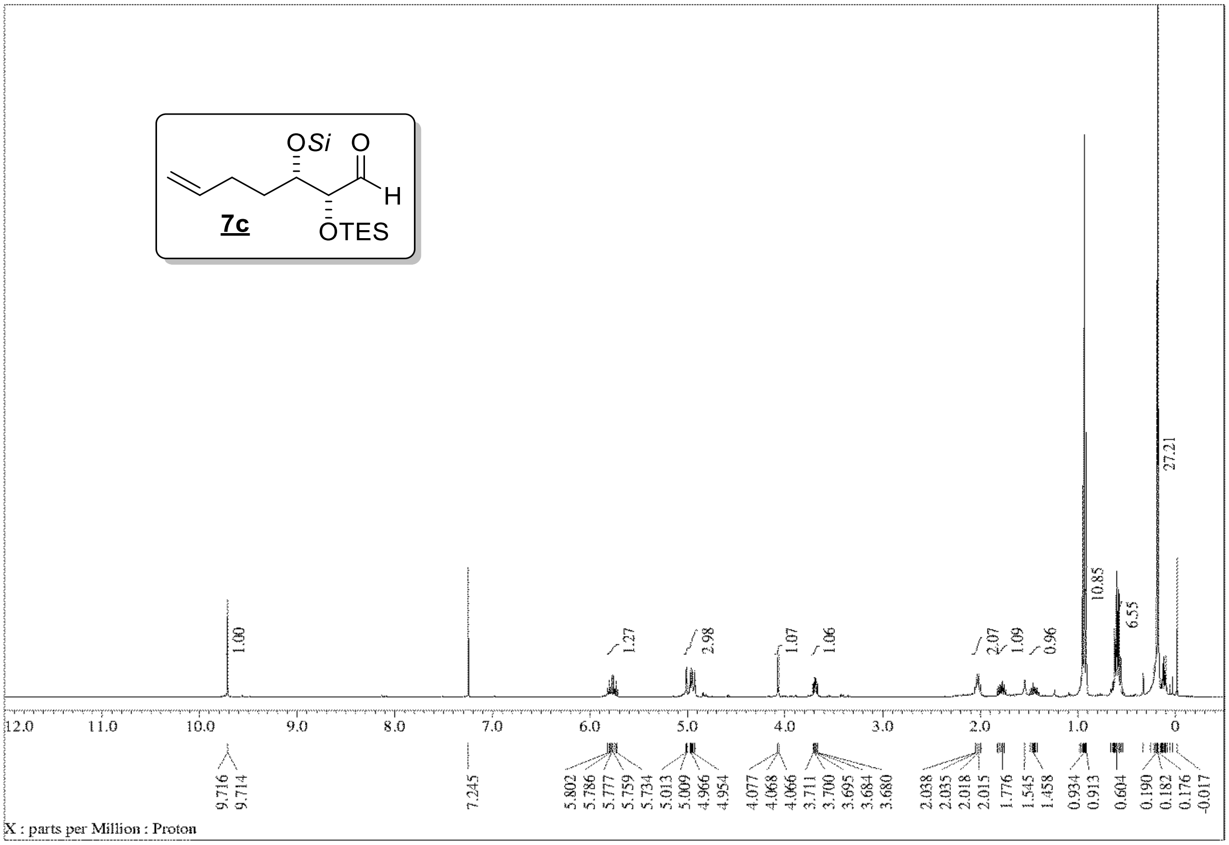
OSi O
H
7c
OTES
1.00
1.27
2.98
1.07
1.06
2.07
1.09
0.96
10.85
6.55
27.21
12.0 11.0 10.0 9.0 8.0 7.0 6.0 5.0 4.0 3.0 2.0 1.0 0
9.716
9.714
7.245
5.802
5.786
5.777
5.759
5.734
5.013
5.009
4.966
4.954
4.077
4.068
4.066
3.711
3.700
3.695
3.684
3.680
2.038
2.035
2.018
2.015
1.776
1.545
1.458
0.934
0.913
0.604
0.190
0.182
0.176
-0.017
X : parts per Million : Proton

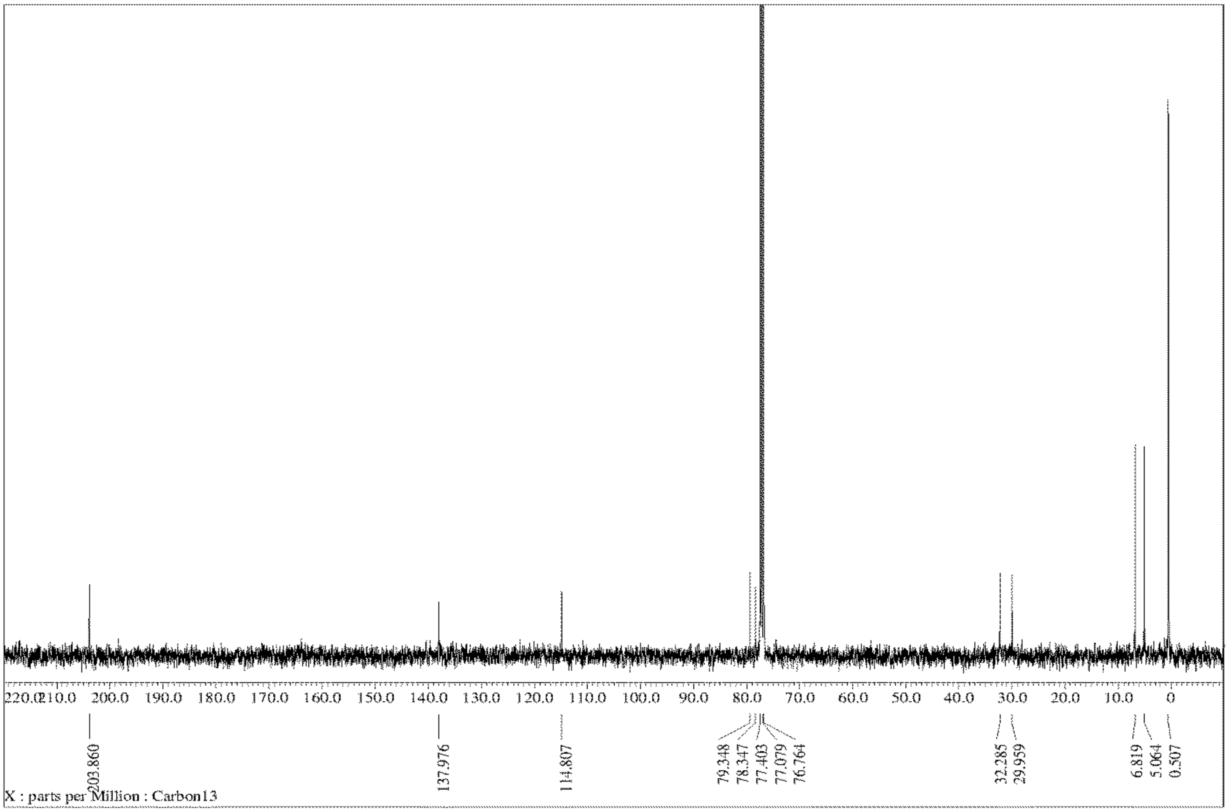
220.0 210.0 200.0 190.0 180.0 170.0 160.0 150.0 140.0 130.0 120.0 110.0 100.0 90.0 80.0 70.0 60.0 50.0 40.0 30.0 20.0 10.0 0
203.860
137.976
114.807
79.348
78.347
77.403
77.079
76.764
32.285
29.959
6.819
5.064
0.507
X : parts per Million : Carbon13

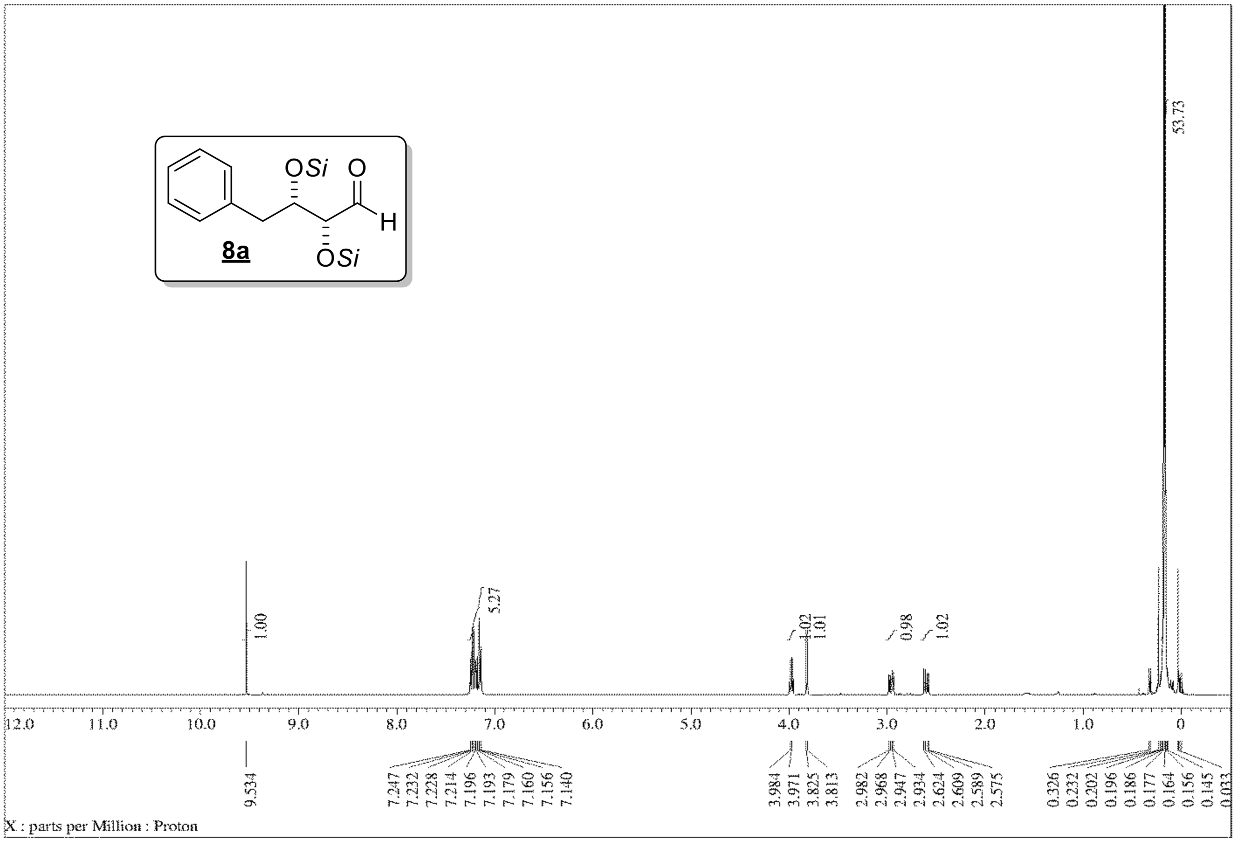

OSi
O
H
8a
OSi
53.73
1.00
5.27
1.02
1.01
0.98
1.02
12.0 11.0 10.0 9.0 8.0 7.0 6.0 5.0 4.0 3.0 2.0 1.0 0
9.534
7.247 7.232 7.228 7.214 7.196 7.193 7.179 7.160 7.156 7.140
3.984 3.971 3.825 3.813
2.982 2.968 2.947 2.934 2.624 2.609 2.589 2.575
0.326 0.232 0.202 0.196 0.186 0.177 0.164 0.156 0.145 0.033
X : parts per Million : Proton

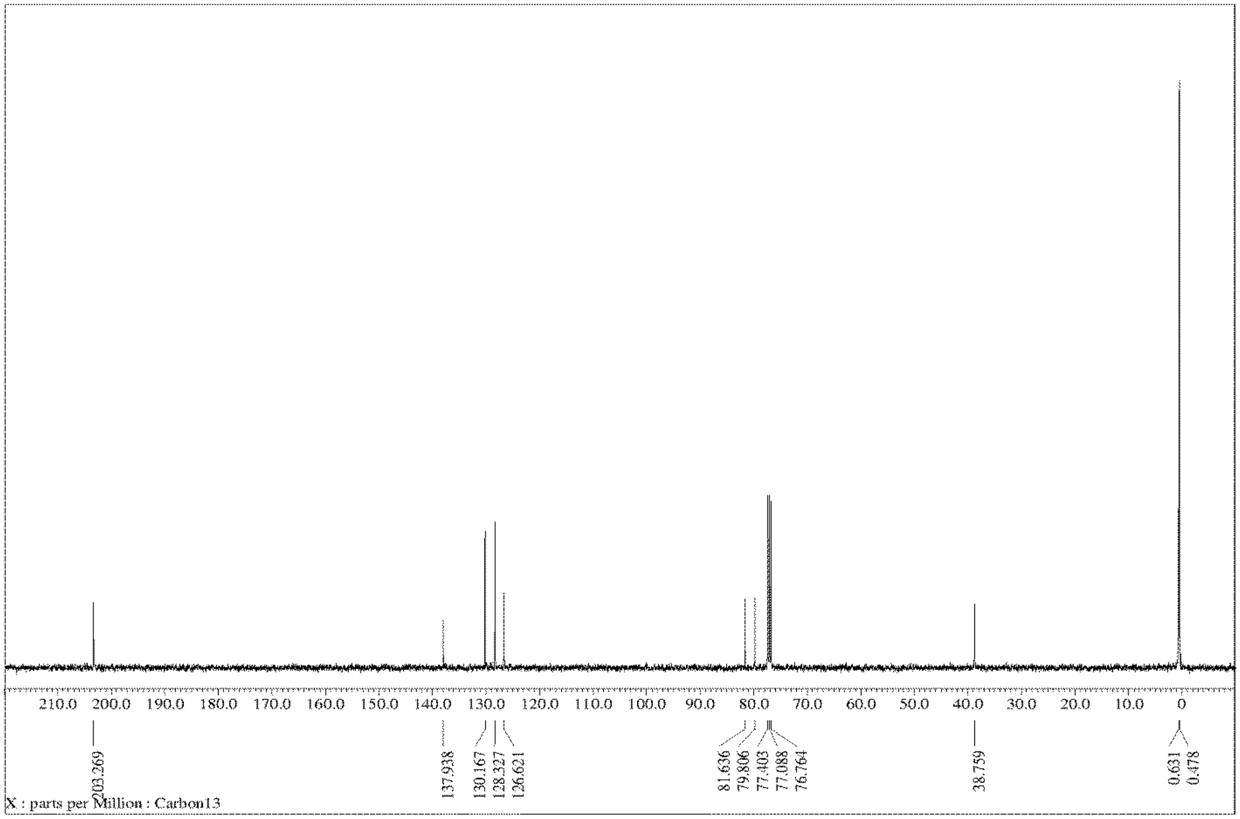

210.0 200.0 190.0 180.0 170.0 160.0 150.0 140.0 130.0 120.0 110.0 100.0 90.0 80.0 70.0 60.0 50.0 40.0 30.0 20.0 10.0 0
203.269
137.938
130.167 128.327 126.621
81.636 79.806 77.403 77.088 76.764
38.759
0.631 0.478
X : parts per Million : Carbon13

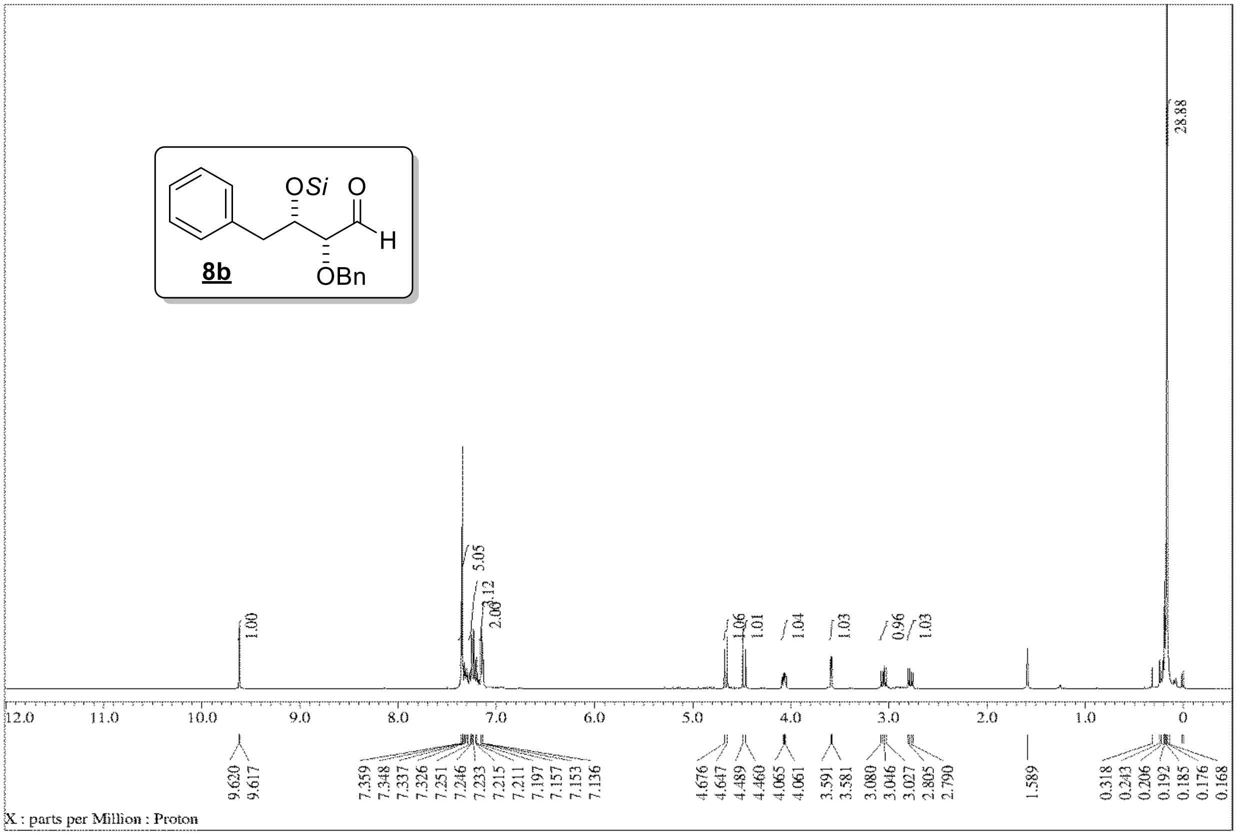

OSi
O
H
8b
OBn
28.88
5.05
3.12
2.03
1.00
1.06
1.01
1.04
1.03
0.96
1.03
12.0 11.0 10.0 9.0 8.0 7.0 6.0 5.0 4.0 3.0 2.0 1.0 0
9.620 9.617
7.359 7.348 7.337 7.326 7.251 7.246 7.233 7.215 7.211 7.197 7.157 7.153 7.136
4.676 4.647 4.489 4.460 4.065 4.061 3.591 3.581 3.080 3.046 3.027 2.805 2.790
1.589
0.318 0.243 0.206 0.192 0.185 0.176 0.168
X : parts per Million : Proton

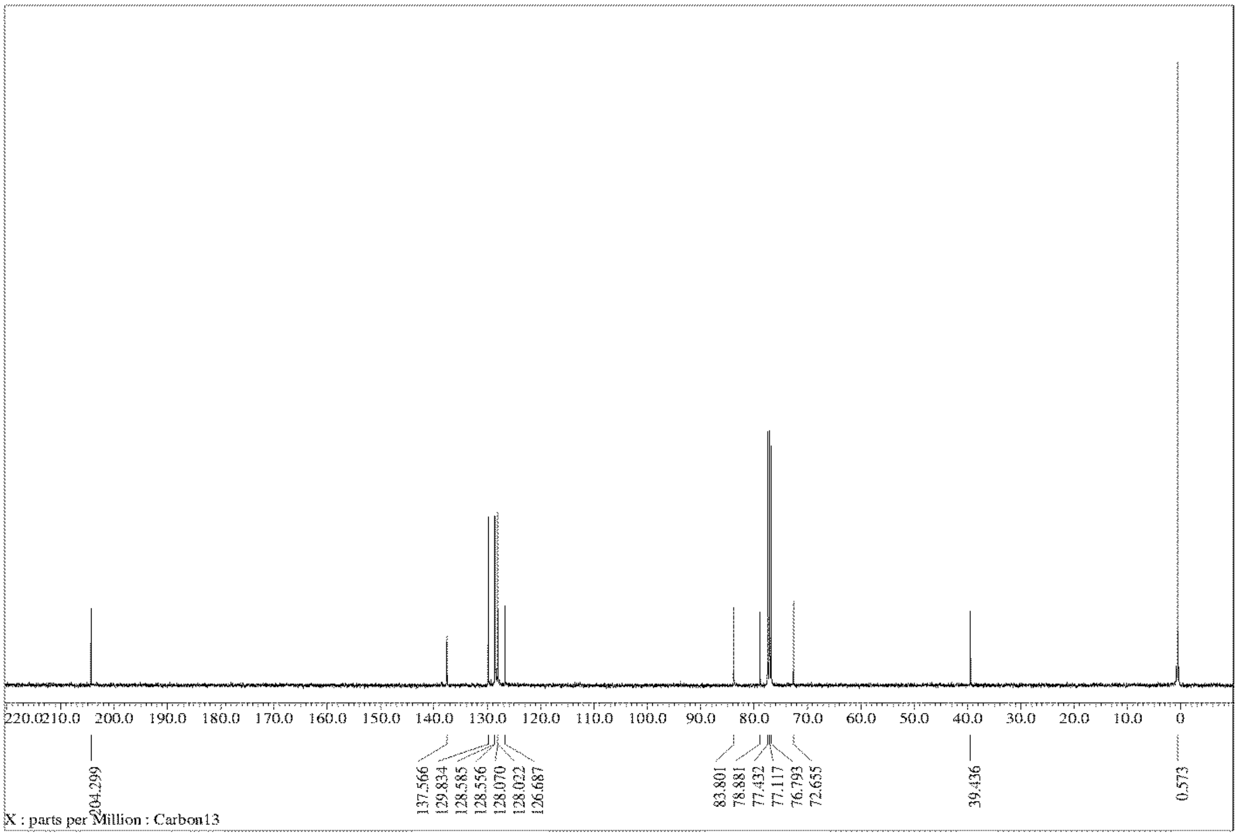

220.0 210.0 200.0 190.0 180.0 170.0 160.0 150.0 140.0 130.0 120.0 110.0 100.0 90.0 80.0 70.0 60.0 50.0 40.0 30.0 20.0 10.0 0
204.299
137.566 129.834 128.585 128.556 128.070 128.022 126.687
83.801 78.881 77.432 77.117 76.793 72.655
39.436
0.573
X : parts per Million : Carbon13

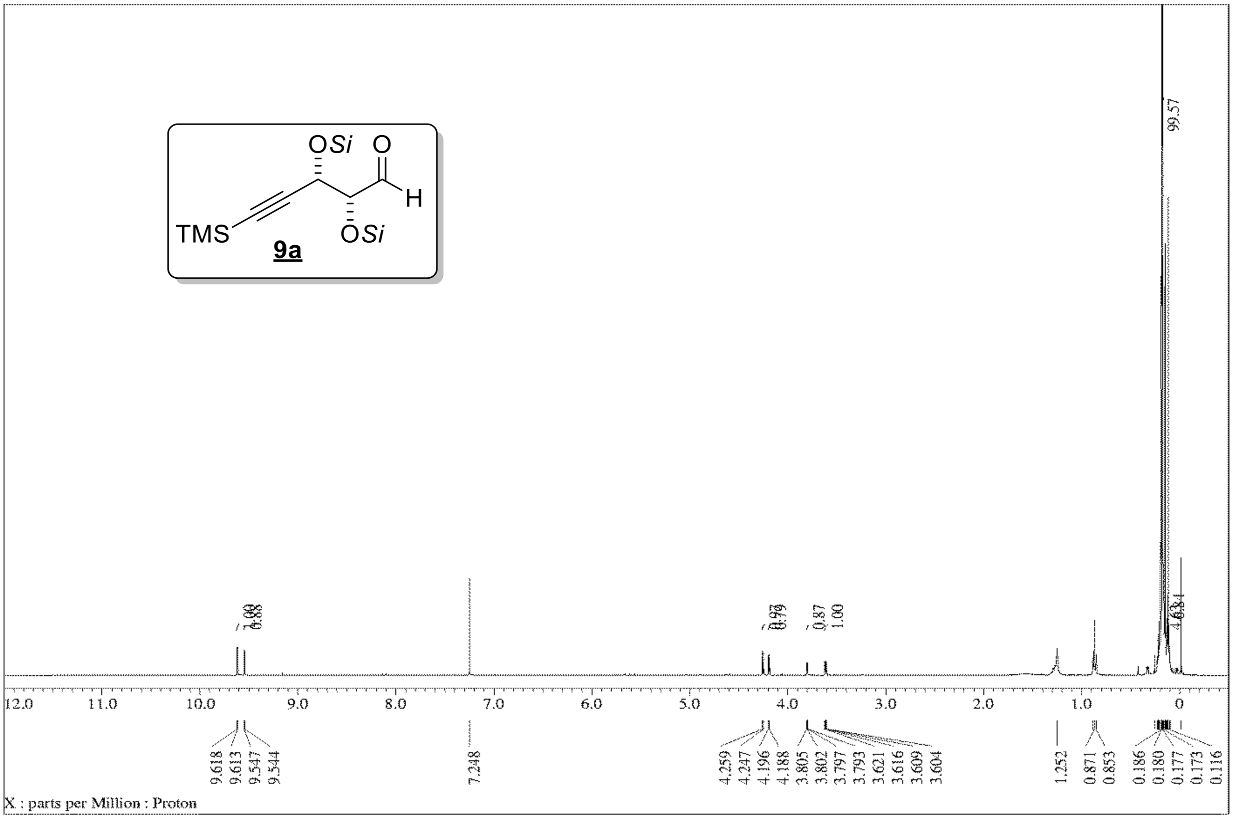
OSi
O
H
TMS
9a
OSi
99.57
12.0 11.0 10.0 9.0 8.0 7.0 6.0 5.0 4.0 3.0 2.0 1.0 0
9.618 9.613 9.547 9.544
7.248
4.259 4.247 4.196 4.188 3.805 3.802 3.797 3.793 3.621 3.616 3.609 3.604
0.87 1.00
1.252
0.871 0.853
0.186 0.180 0.177 0.173 0.116
X : parts per Million : Proton

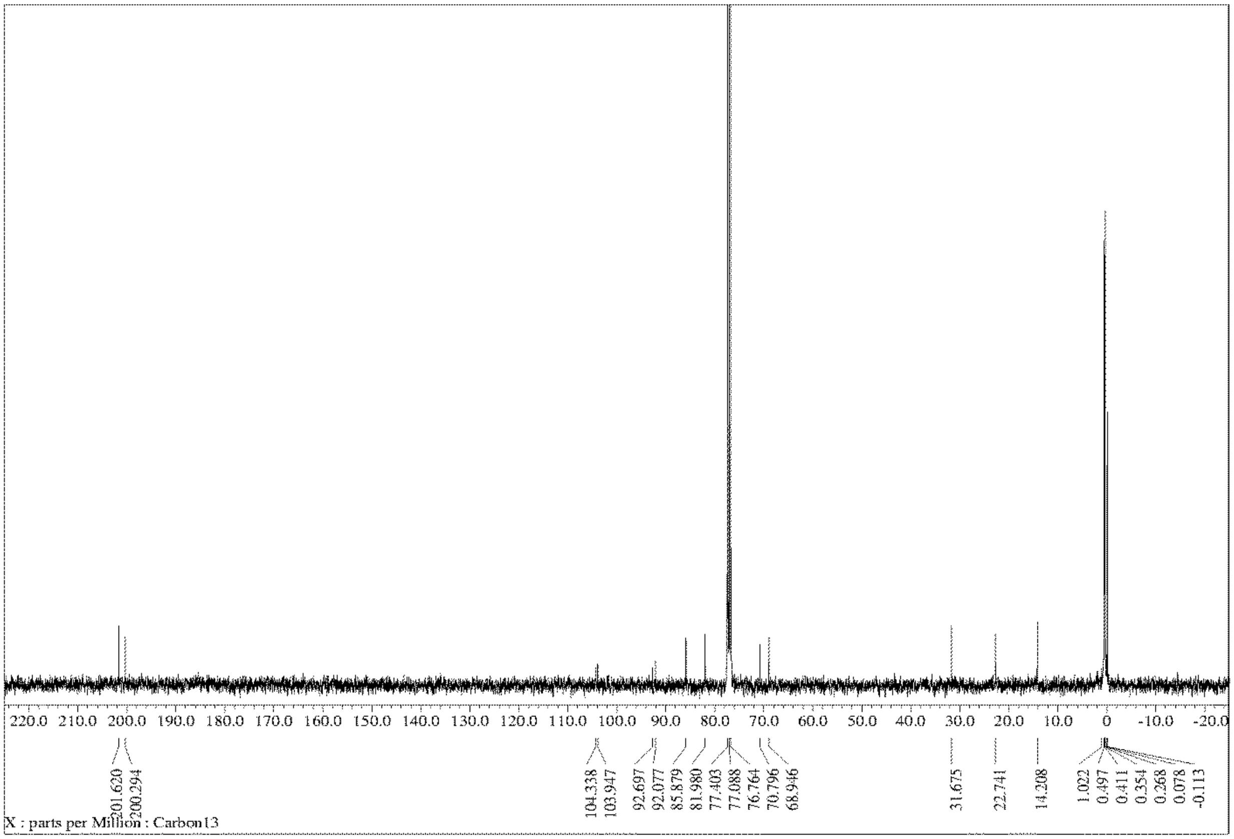
220.0 210.0 200.0 190.0 180.0 170.0 160.0 150.0 140.0 130.0 120.0 110.0 100.0 90.0 80.0 70.0 60.0 50.0 40.0 30.0 20.0 10.0 0 -10.0 -20.0
201.620 200.294
104.338 103.947
92.697 92.077 85.879 81.980 77.403 77.088 76.764 70.796 68.946
31.675
22.741
14.208
1.022 0.497 0.411 0.354 0.268 0.078 -0.113
X : parts per Million : Carbon13

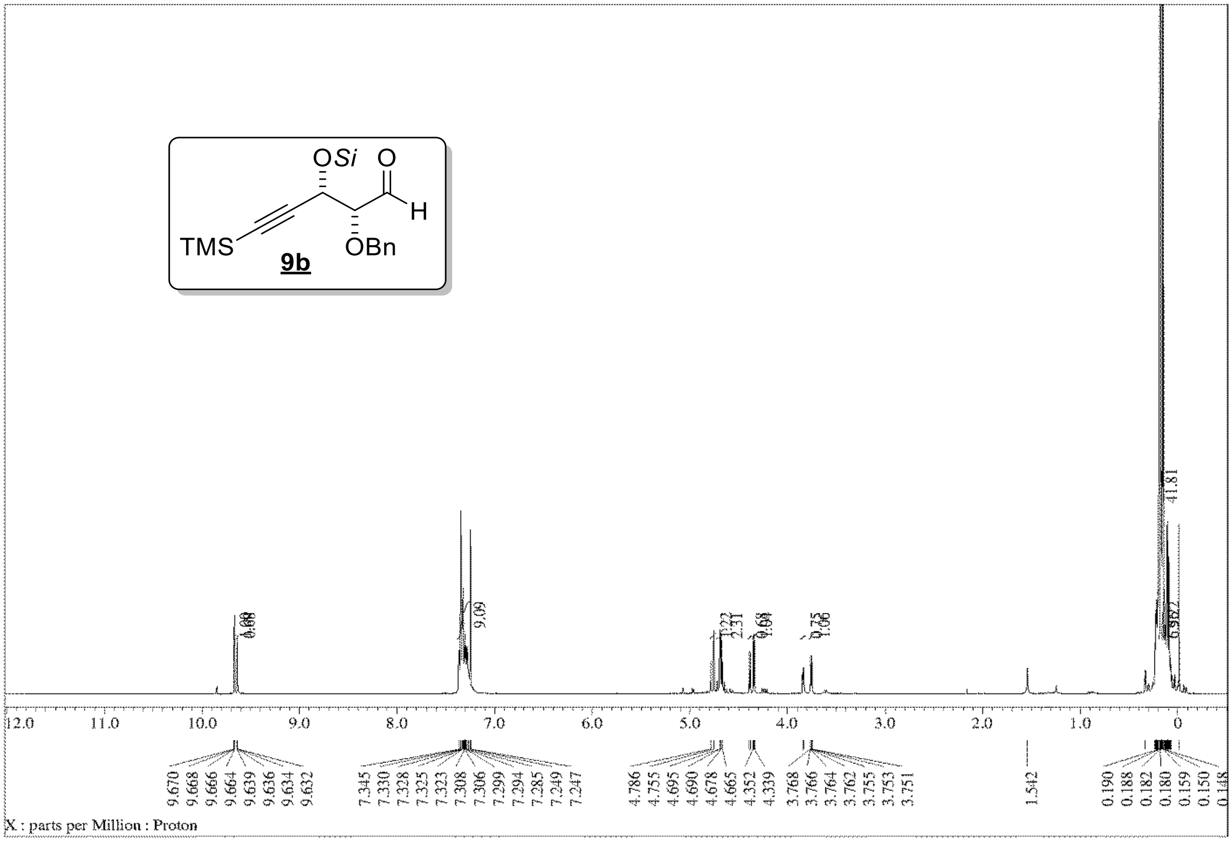
OSi
O
H
TMS
OBn
9b
X : parts per Million : Proton

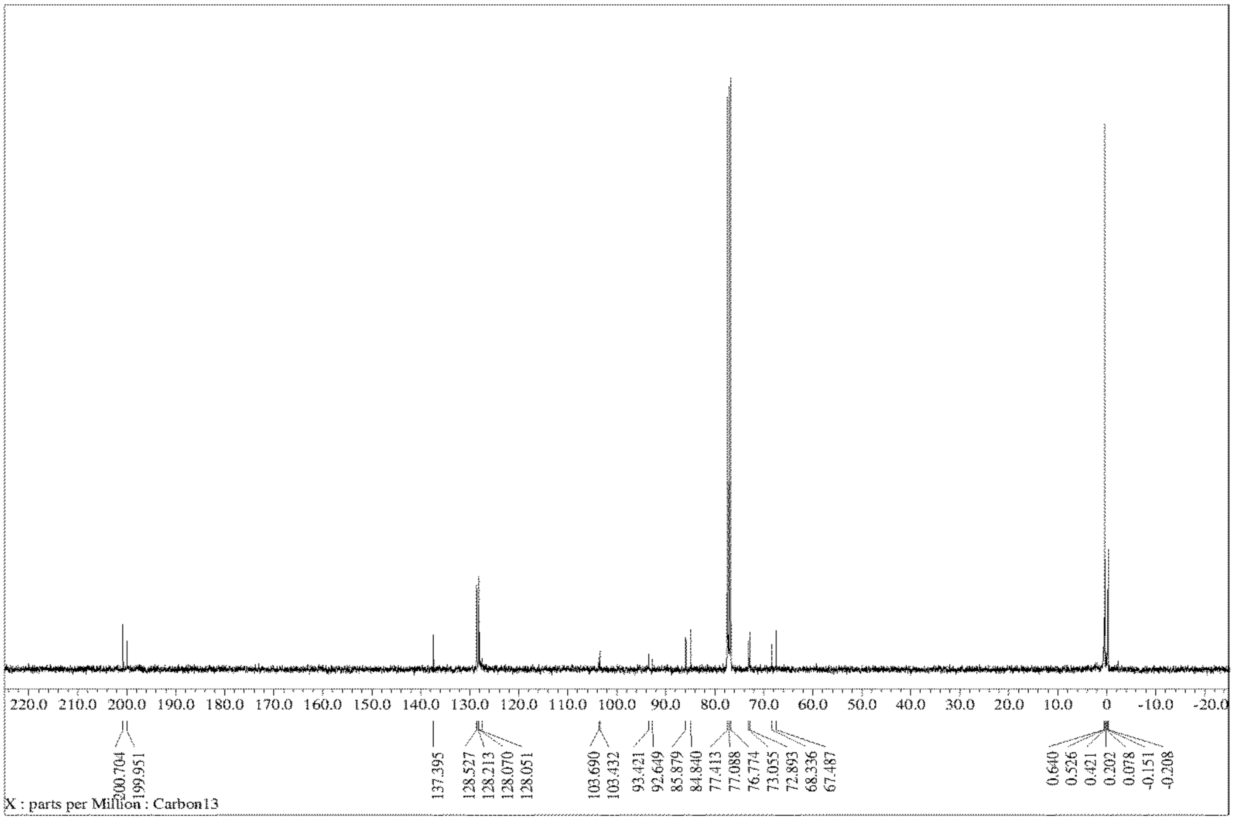
X : parts per Million : Carbon13

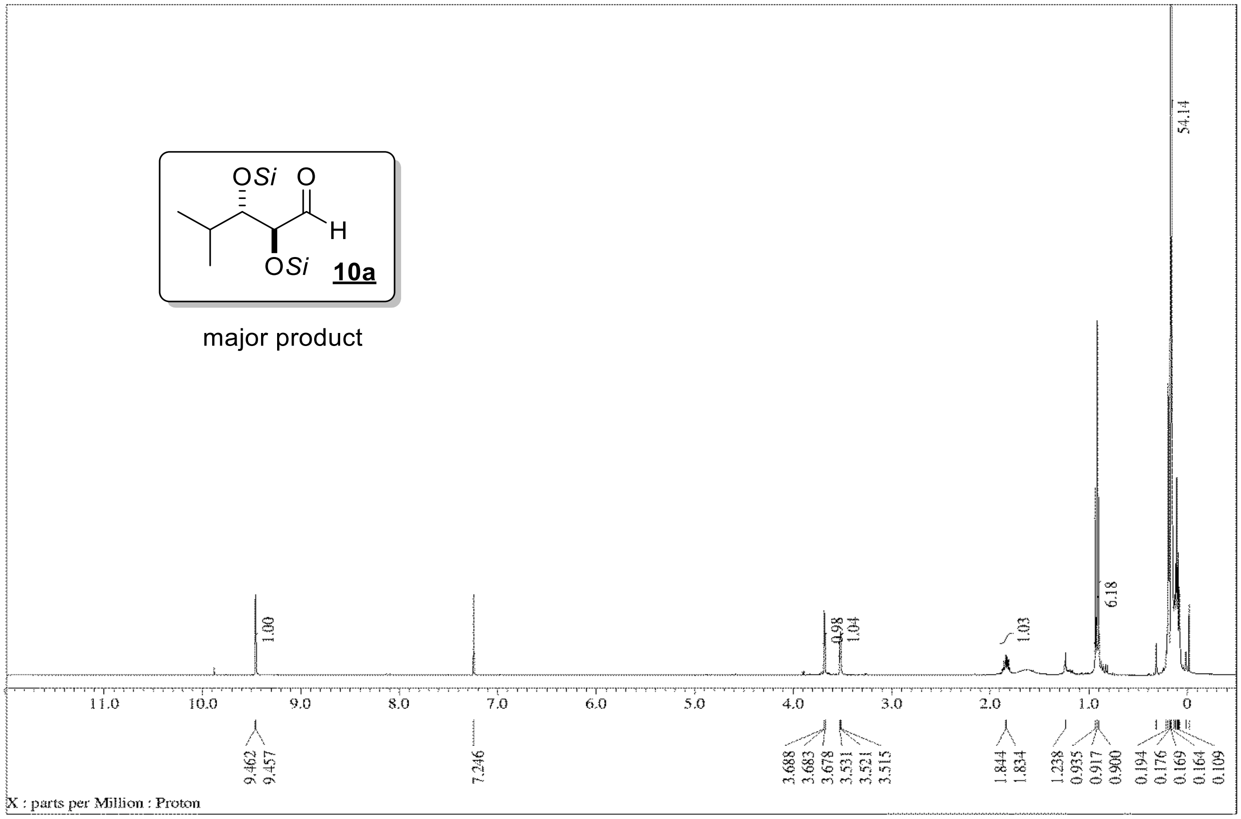
OSi
O
H
OSi
10a
major product
54.14
6.18
1.03
0.98
1.04
1.00
9.462
9.457
7.246
3.688
3.683
3.678
3.531
3.521
3.515
1.844
1.834
1.238
0.935
0.917
0.900
0.194
0.176
0.169
0.164
0.109
X : parts per Million : Proton

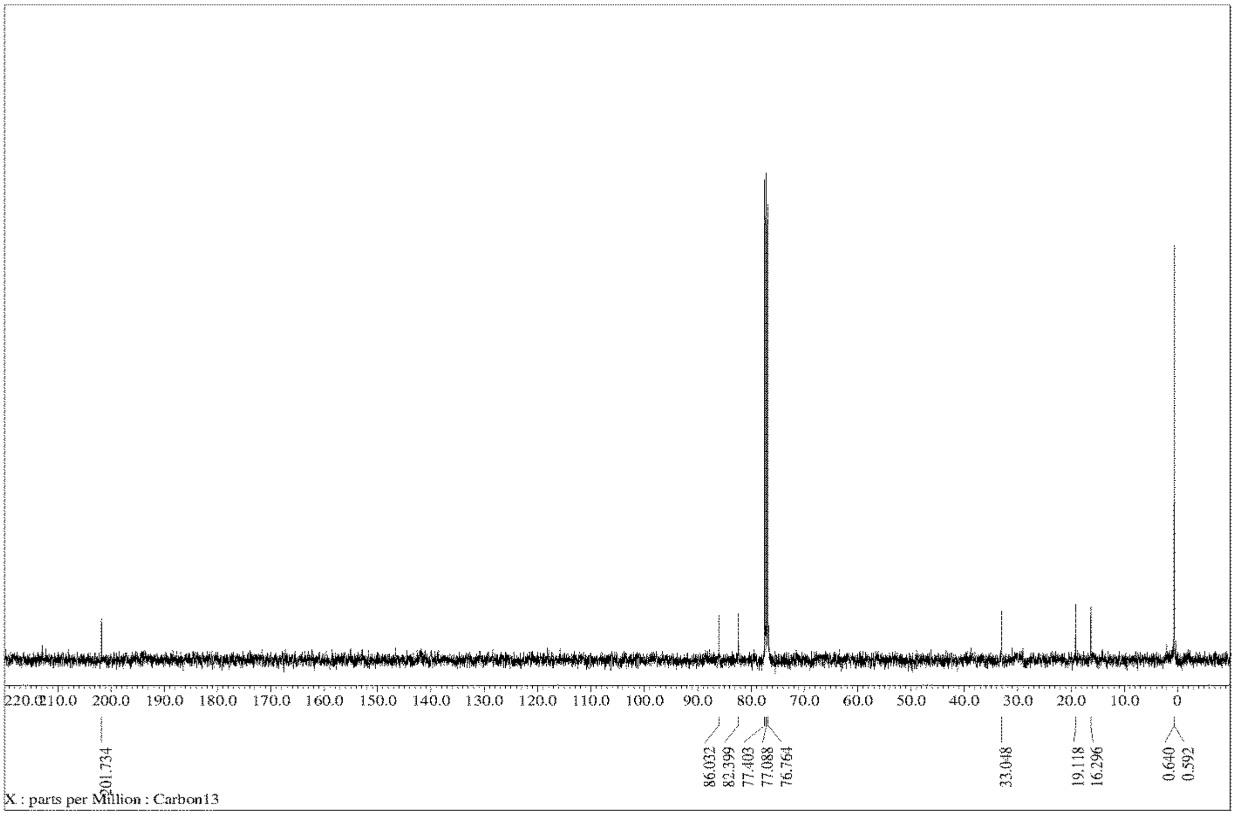
201.734
86.032
82.399
77.403
77.088
76.764
33.048
19.118
16.296
0.640
0.592
X : parts per Million : Carbon13

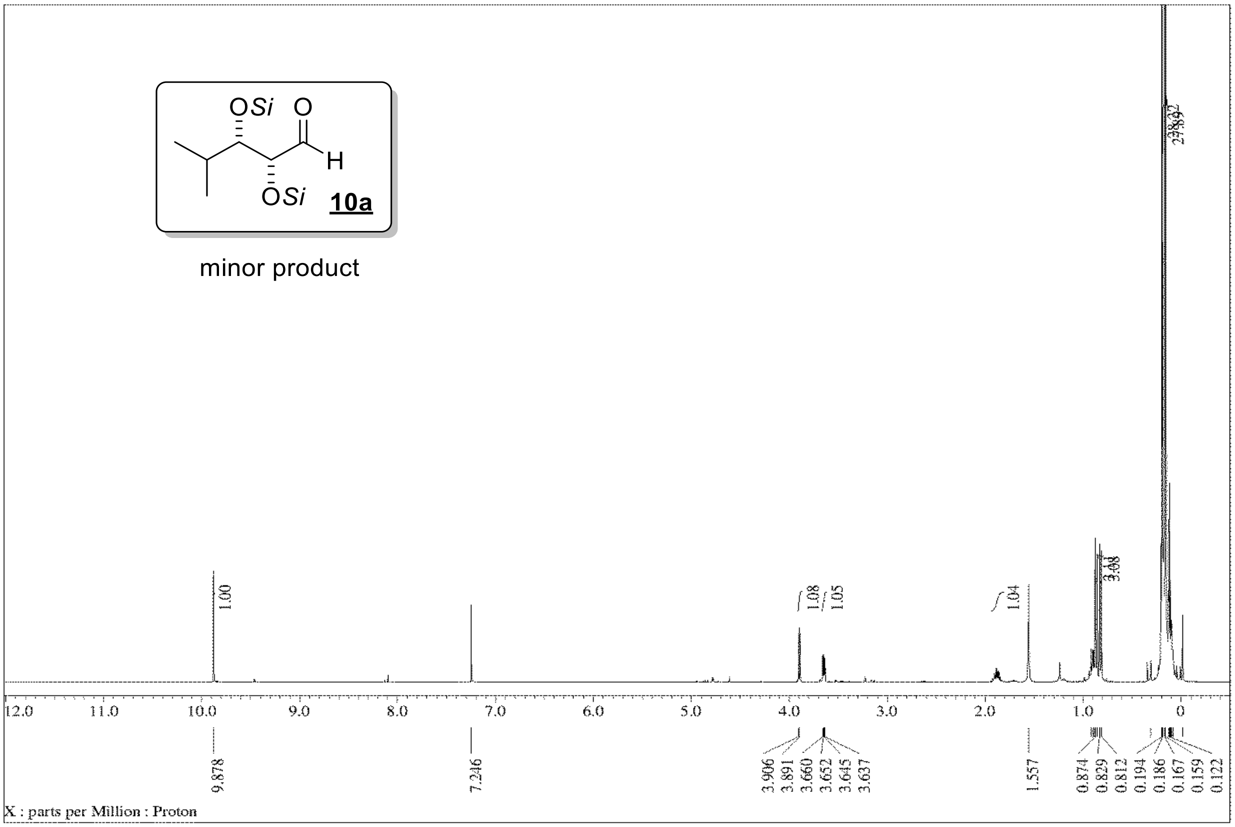
OSi
O
H
OSi
10a
minor product
1.00
1.08
1.05
1.04
3.11
3.08
12.0
11.0
10.0
9.0
8.0
7.0
6.0
5.0
4.0
3.0
2.0
1.0
0
9.878
7.246
3.906
3.891
3.660
3.652
3.645
3.637
1.557
0.874
0.829
0.812
0.194
0.186
0.167
0.159
0.122
X : parts per Million : Proton

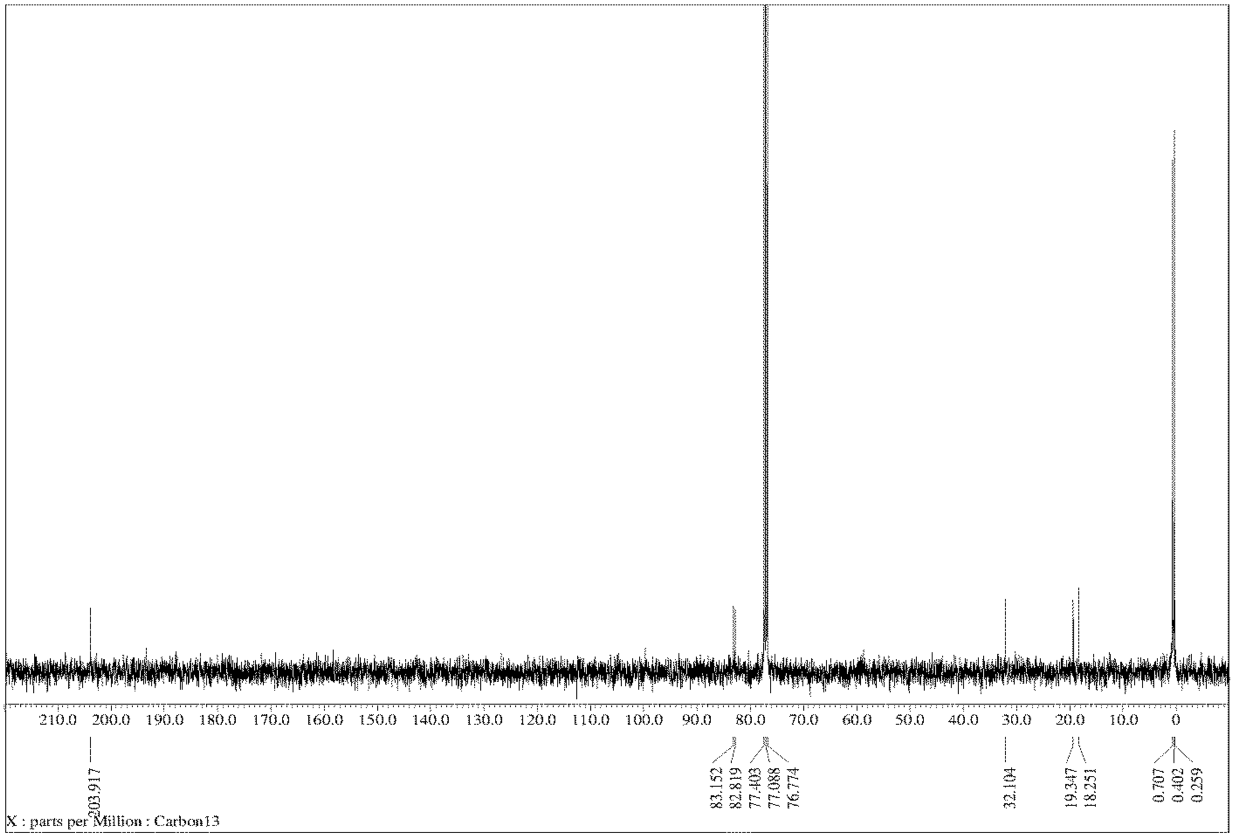
210.0
200.0
190.0
180.0
170.0
160.0
150.0
140.0
130.0
120.0
110.0
100.0
90.0
80.0
70.0
60.0
50.0
40.0
30.0
20.0
10.0
0
203.917
83.152
82.819
77.403
77.088
76.774
32.104
19.347
18.251
0.707
0.402
0.259
X : parts per Million : Carbon13

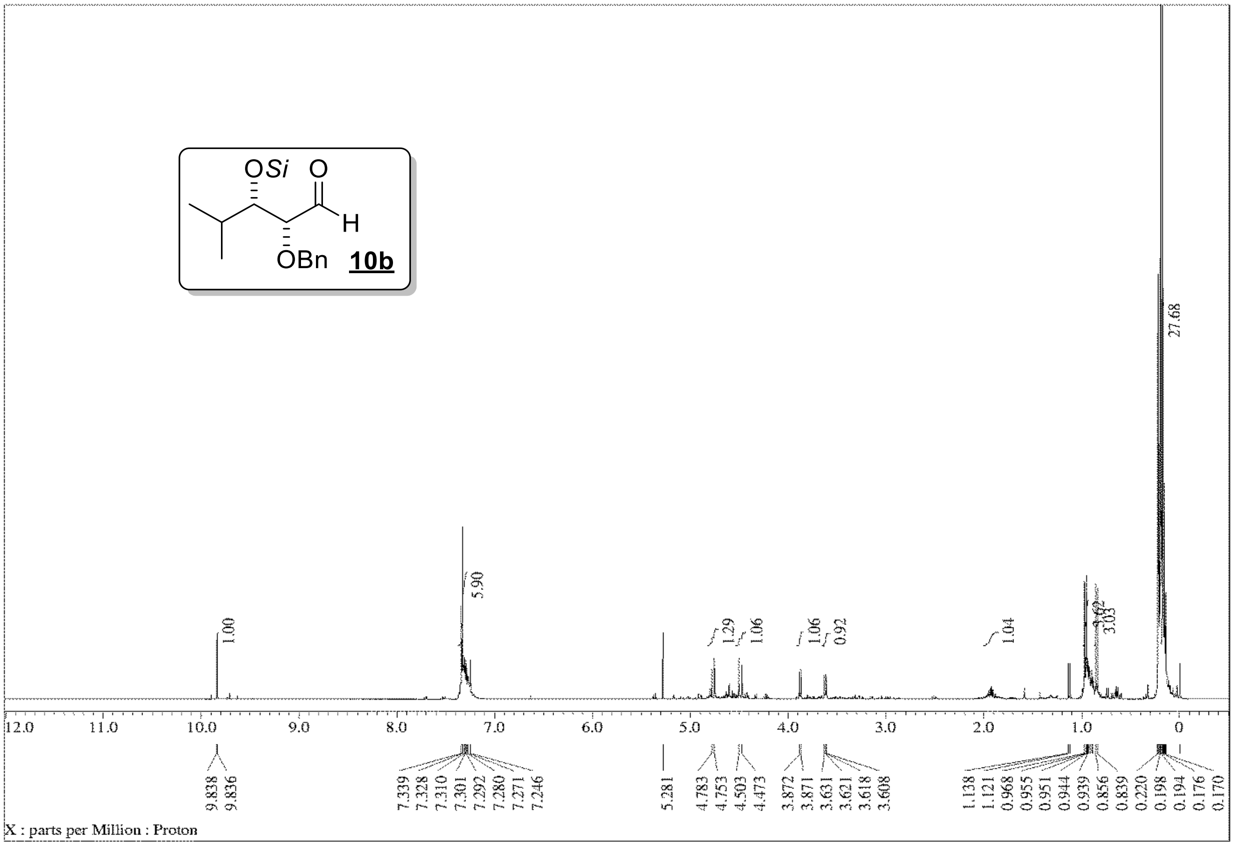

OSi
O
H
OBn
10b
1.00
5.90
1.29
1.06
1.06
0.92
1.04
3.03
27.68
9.838
9.836
7.339
7.328
7.310
7.301
7.292
7.280
7.271
7.246
5.281
4.783
4.753
4.503
4.473
3.872
3.871
3.631
3.621
3.618
3.608
1.138
1.121
0.968
0.955
0.951
0.944
0.939
0.856
0.839
0.220
0.198
0.194
0.176
0.170
X : parts per Million : Proton

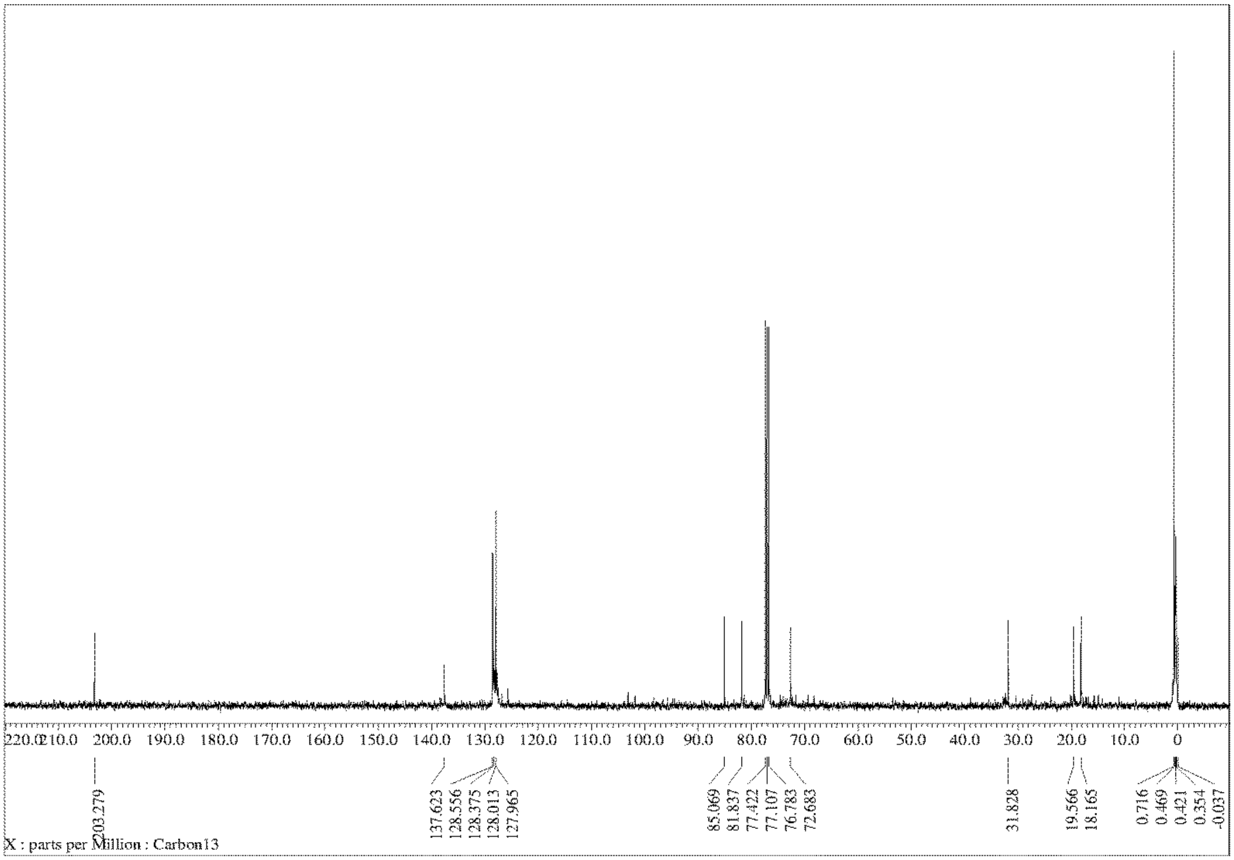

203.279
137.623
128.556
128.375
128.013
127.965
85.069
81.837
77.422
77.107
76.783
72.683
31.828
19.566
18.165
0.716
0.469
0.421
0.354
-0.037
X : parts per Million : Carbon13

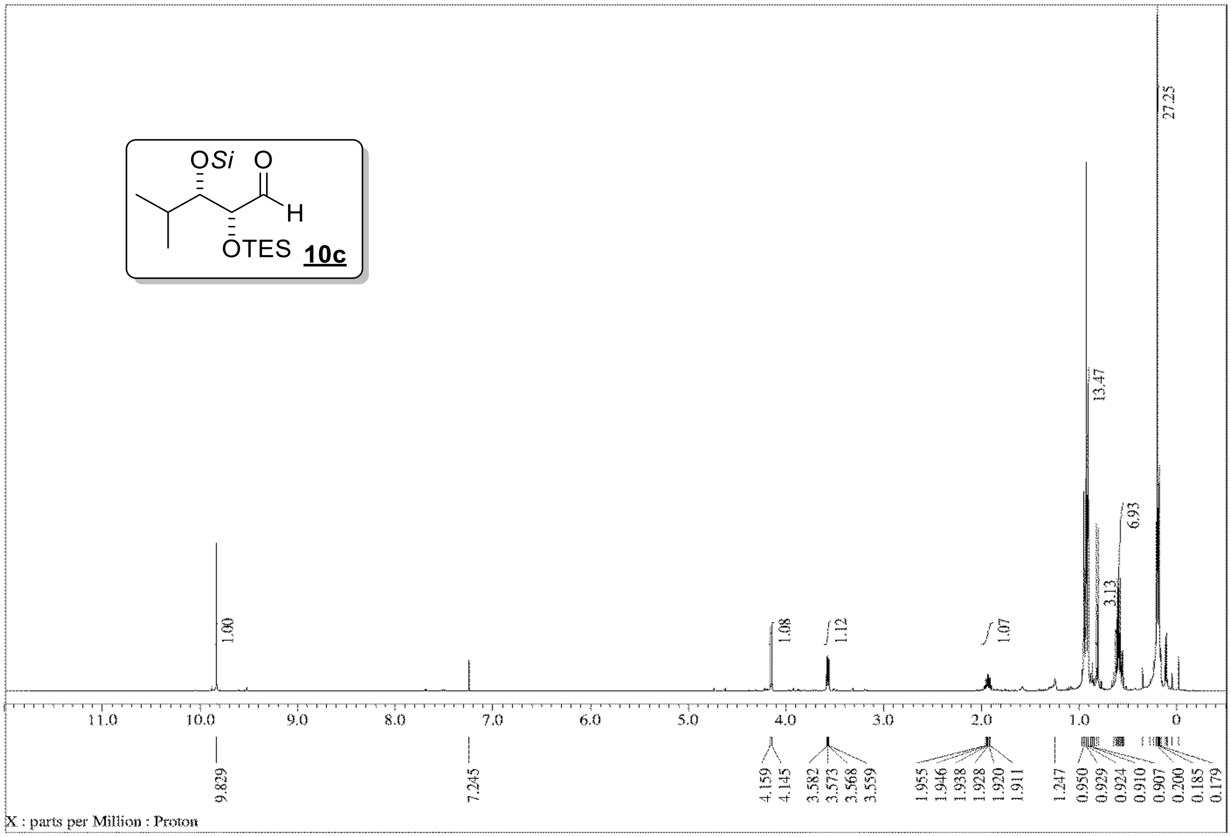
OSi O
H
OTES 10c
1.00
1.08
1.12
1.07
13.47
3.13
6.93
27.25
11.0 10.0 9.0 8.0 7.0 6.0 5.0 4.0 3.0 2.0 1.0 0
9.829
7.245
4.159
4.145
3.582
3.573
3.568
3.559
1.955
1.946
1.938
1.928
1.920
1.911
1.247
0.950
0.929
0.924
0.910
0.907
0.200
0.185
0.179
X : parts per Million : Proton

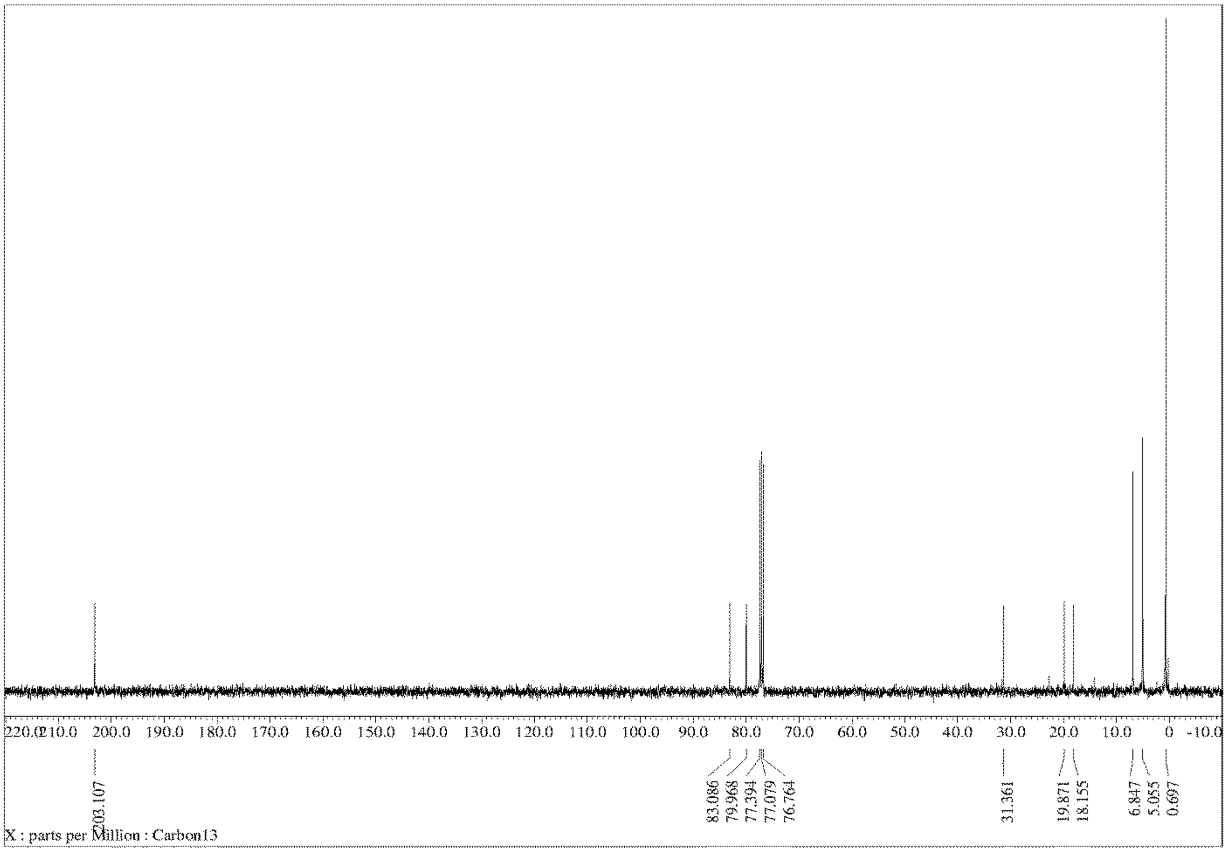
220.0 210.0 200.0 190.0 180.0 170.0 160.0 150.0 140.0 130.0 120.0 110.0 100.0 90.0 80.0 70.0 60.0 50.0 40.0 30.0 20.0 10.0 0 -10.0
203.107
83.086
79.968
77.394
77.079
76.764
31.361
19.871
18.155
6.847
5.055
0.697
X : parts per Million : Carbon13

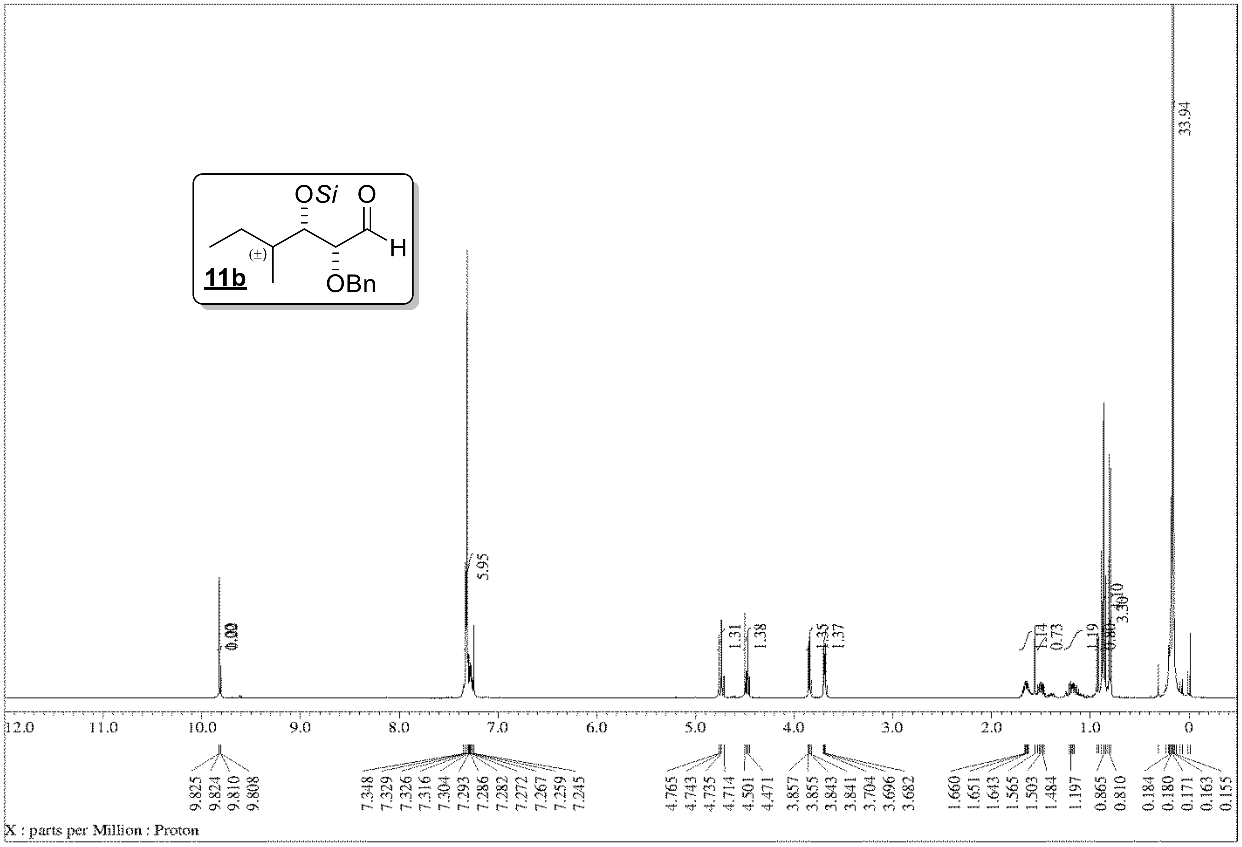
OSi O
H
(±)
11b
OBn
33.94
5.95
1.31
1.38
1.35
1.37
1.14
0.73
1.19
0.80
3.10
3.30
12.0 11.0 10.0 9.0 8.0 7.0 6.0 5.0 4.0 3.0 2.0 1.0 0
9.825 9.824 9.810 9.808
7.348 7.329 7.326 7.316 7.304 7.293 7.286 7.282 7.272 7.267 7.259 7.245
4.765 4.743 4.735 4.714 4.501 4.471
3.857 3.855 3.843 3.841 3.704 3.696 3.682
1.660 1.651 1.643 1.565 1.503 1.484 1.197
0.865 0.810
0.184 0.180 0.171 0.163 0.155
X : parts per Million : Proton

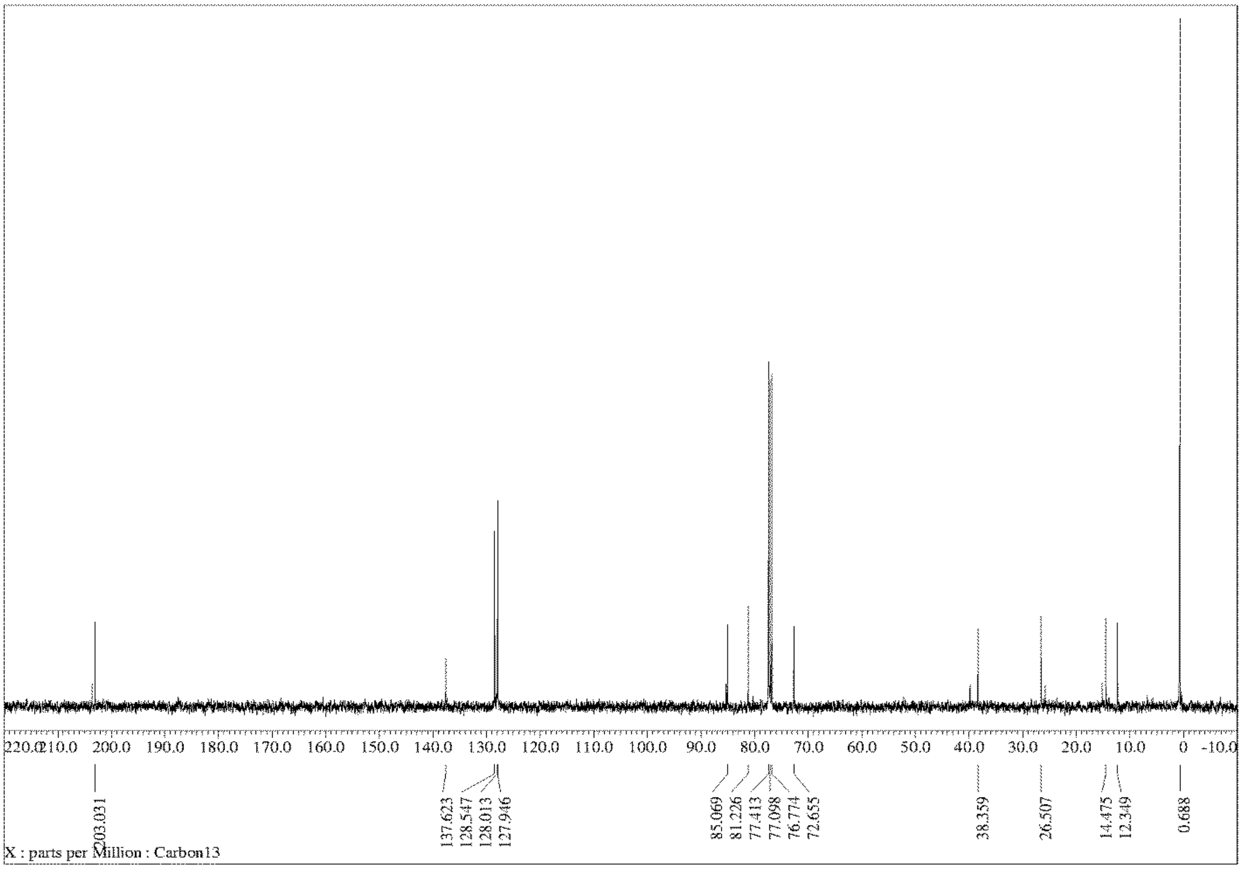
220.0 210.0 200.0 190.0 180.0 170.0 160.0 150.0 140.0 130.0 120.0 110.0 100.0 90.0 80.0 70.0 60.0 50.0 40.0 30.0 20.0 10.0 0 -10.0
203.031
137.623 128.547 128.013 127.946
85.069 81.226 77.413 77.098 76.774 72.655
38.359
26.507
14.475 12.349
0.688
X : parts per Million : Carbon13

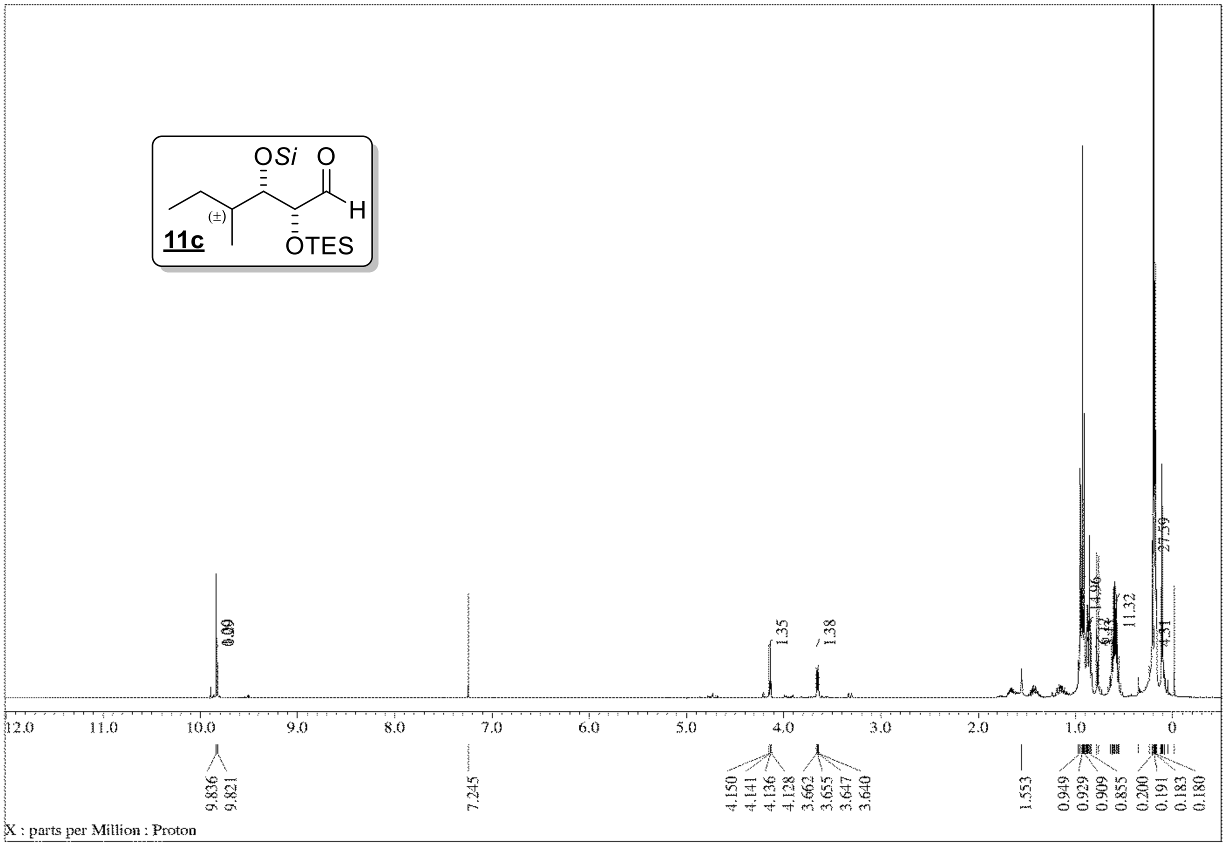
OSi O
H
(±)
11c
OTES
X : parts per Million : Proton
12.0 11.0 10.0 9.0 8.0 7.0 6.0 5.0 4.0 3.0 2.0 1.0 0
9.836 9.821
7.245
4.150 4.141 4.136 4.128 3.662 3.655 3.647 3.640
1.553
0.949 0.929 0.909 0.855 0.200 0.191 0.183 0.180
1.35
1.38
14.96
11.32
27.59
4.31

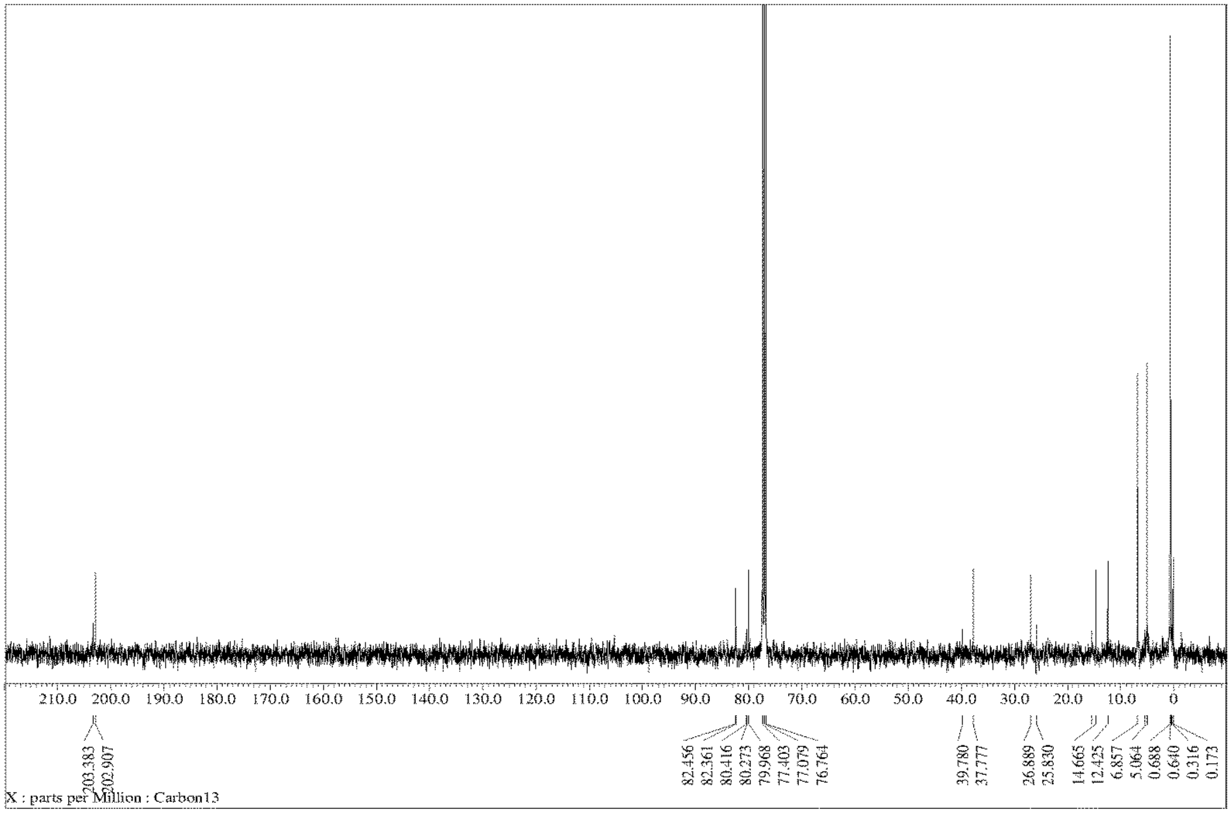
X : parts per Million : Carbon13
210.0 200.0 190.0 180.0 170.0 160.0 150.0 140.0 130.0 120.0 110.0 100.0 90.0 80.0 70.0 60.0 50.0 40.0 30.0 20.0 10.0 0
203.383 202.907
82.456 82.361 80.416 80.273 79.968 77.403 77.079 76.764
39.780 37.777
26.889 25.830
14.665 12.425 6.857 5.064 0.688 0.640 0.316 0.173

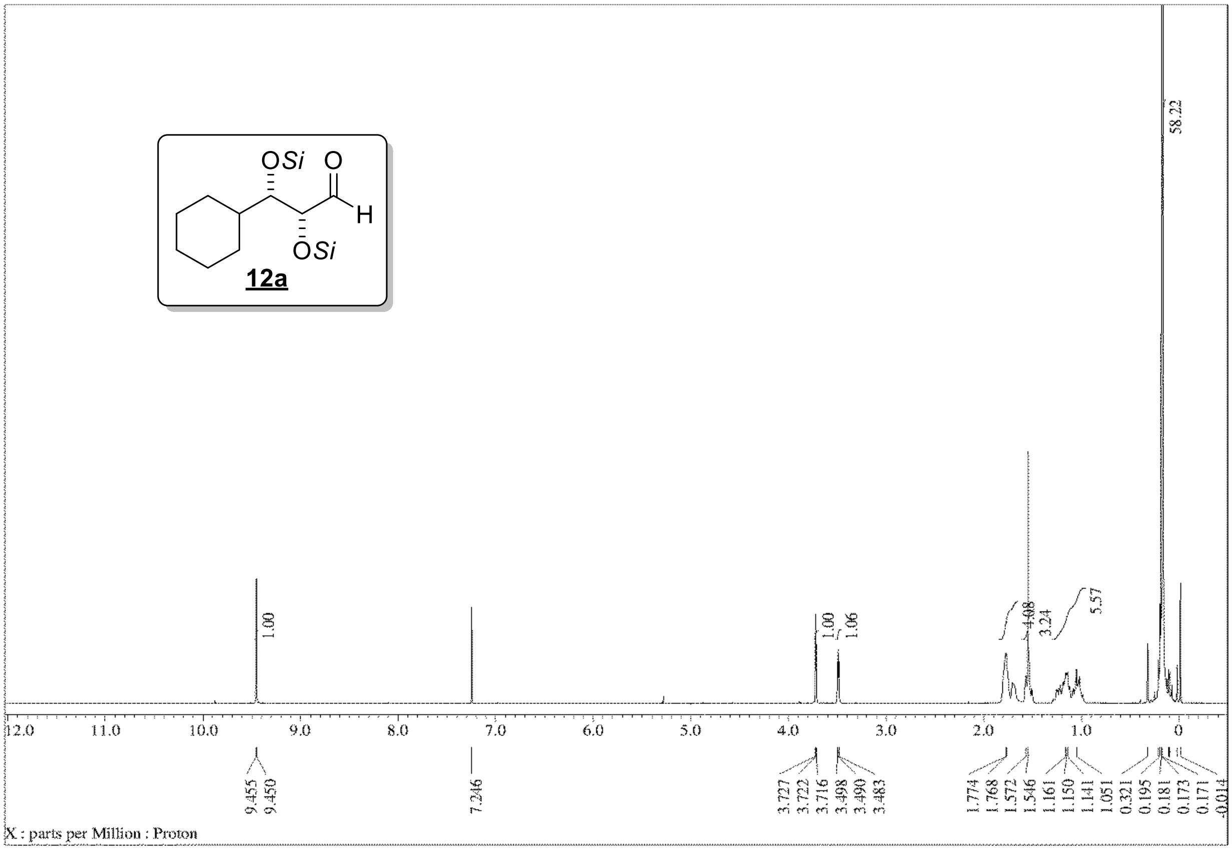
OSi
O
H
OSi
12a
58.22
1.00
1.00
1.06
4.08
3.24
5.57
12.0 11.0 10.0 9.0 8.0 7.0 6.0 5.0 4.0 3.0 2.0 1.0 0
9.455
9.450
7.246
3.727
3.722
3.716
3.498
3.490
3.483
1.774
1.768
1.572
1.546
1.161
1.150
1.141
1.051
0.321
0.195
0.181
0.173
0.171
-0.014
X : parts per Million : Proton

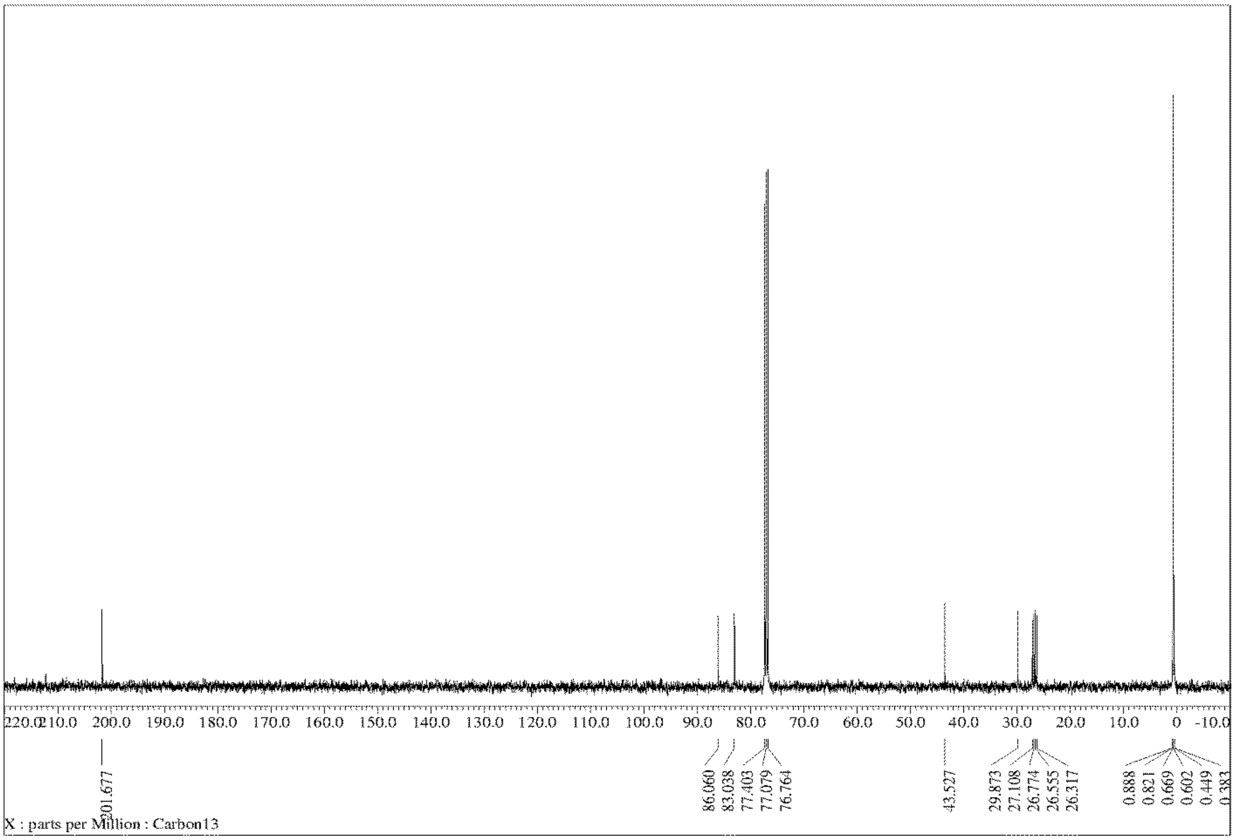
220.0 210.0 200.0 190.0 180.0 170.0 160.0 150.0 140.0 130.0 120.0 110.0 100.0 90.0 80.0 70.0 60.0 50.0 40.0 30.0 20.0 10.0 0 -10.0
201.677
86.060
83.038
77.403
77.079
76.764
43.527
29.873
27.108
26.774
26.555
26.317
0.888
0.821
0.669
0.602
0.449
0.383
X : parts per Million : Carbon13

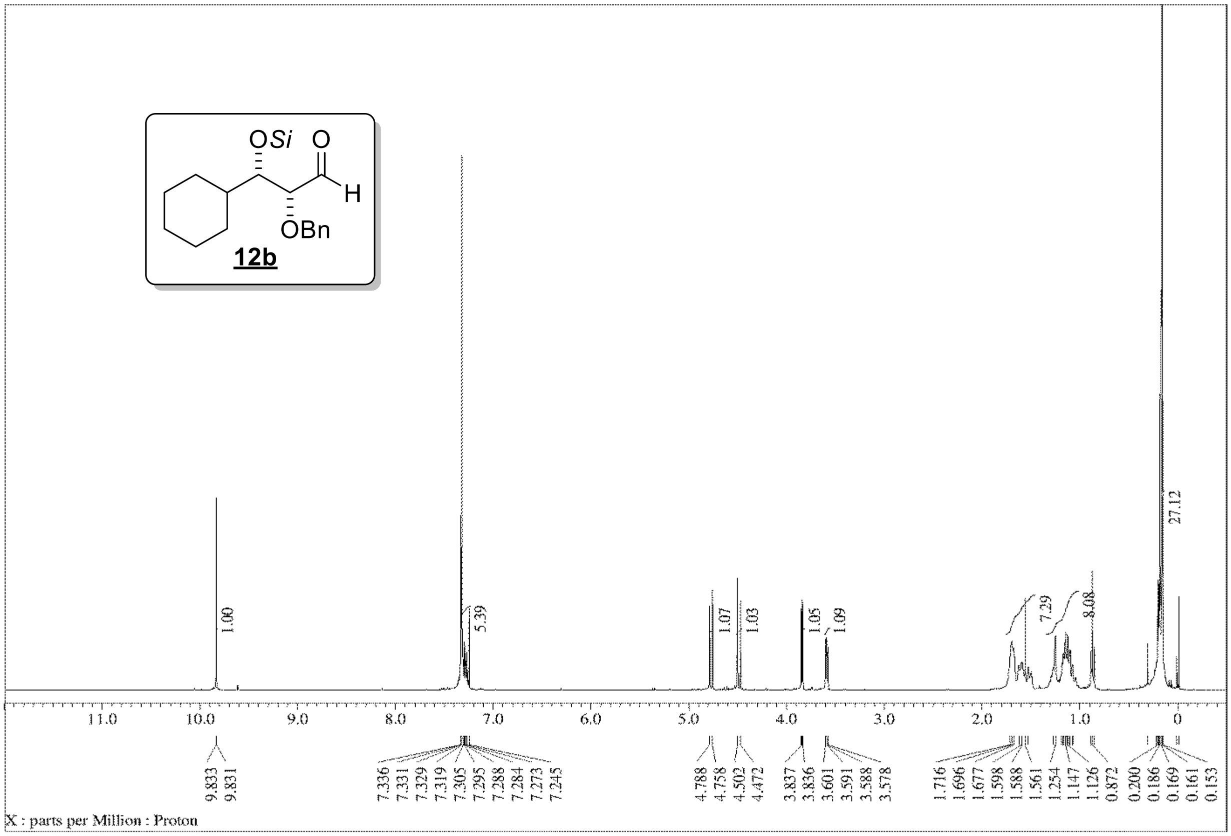

OSi O
H
OBn
12b
1.00
5.39
1.07
1.03
1.05
1.09
7.29
8.08
27.12
11.0 10.0 9.0 8.0 7.0 6.0 5.0 4.0 3.0 2.0 1.0 0
9.833 9.831
7.336 7.331 7.329 7.319 7.305 7.295 7.288 7.284 7.273 7.245
4.788 4.758 4.502 4.472
3.837 3.836 3.601 3.591 3.588 3.578
1.716 1.696 1.677 1.598 1.588 1.561 1.254 1.147 1.126 0.872
0.200 0.186 0.169 0.161 0.153
X : parts per Million : Proton

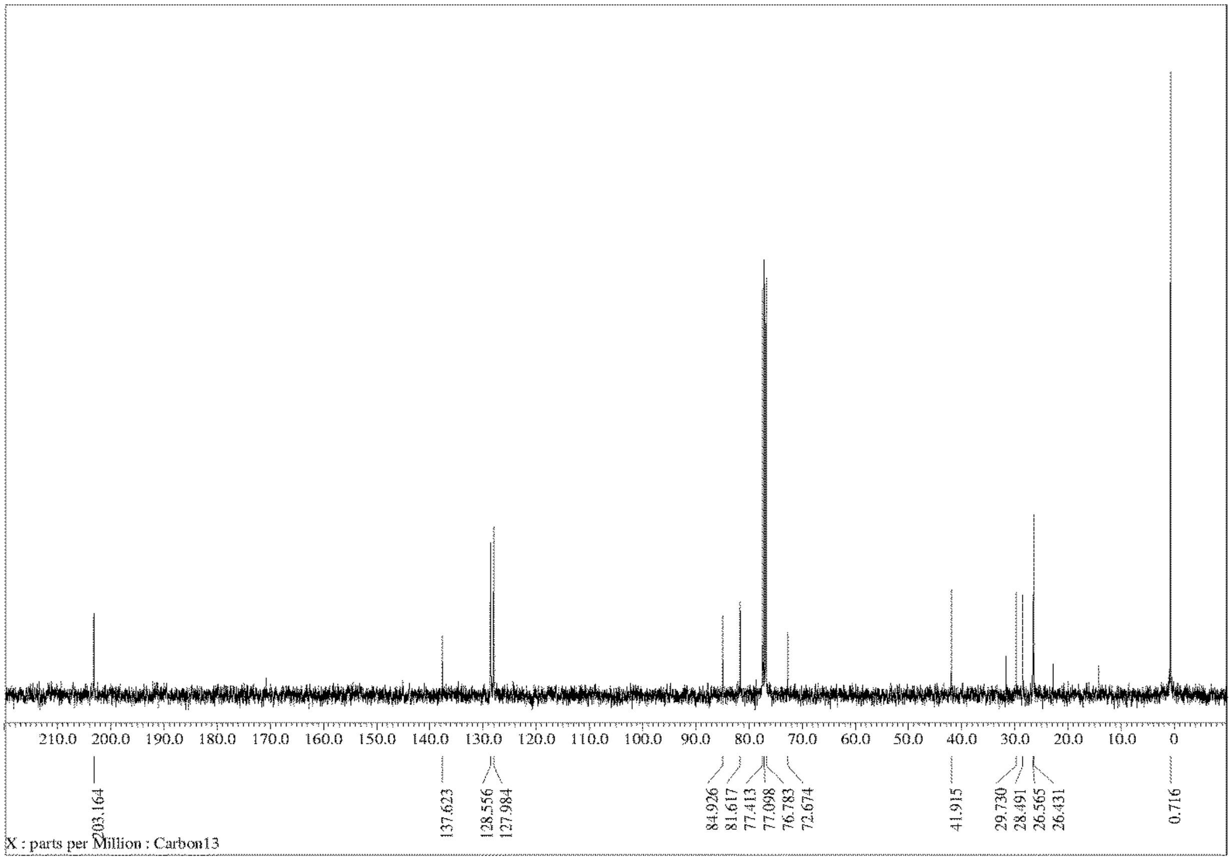

210.0 200.0 190.0 180.0 170.0 160.0 150.0 140.0 130.0 120.0 110.0 100.0 90.0 80.0 70.0 60.0 50.0 40.0 30.0 20.0 10.0 0
203.164
137.623
128.556 127.984
84.926 81.617 77.413 77.098 76.783 72.674
41.915
29.730 28.491 26.565 26.431
0.716
X : parts per Million : Carbon13

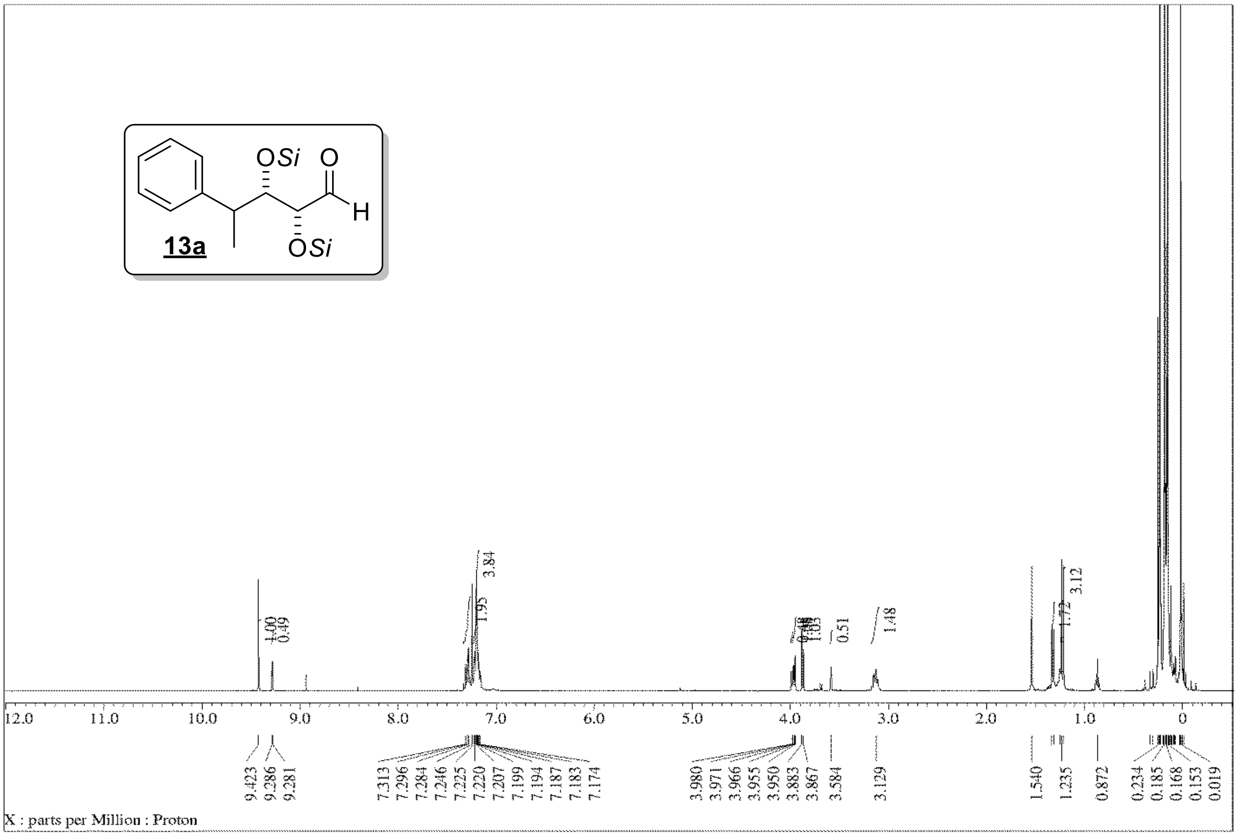
OSi
O
H
13a
OSi
X : parts per Million : Proton

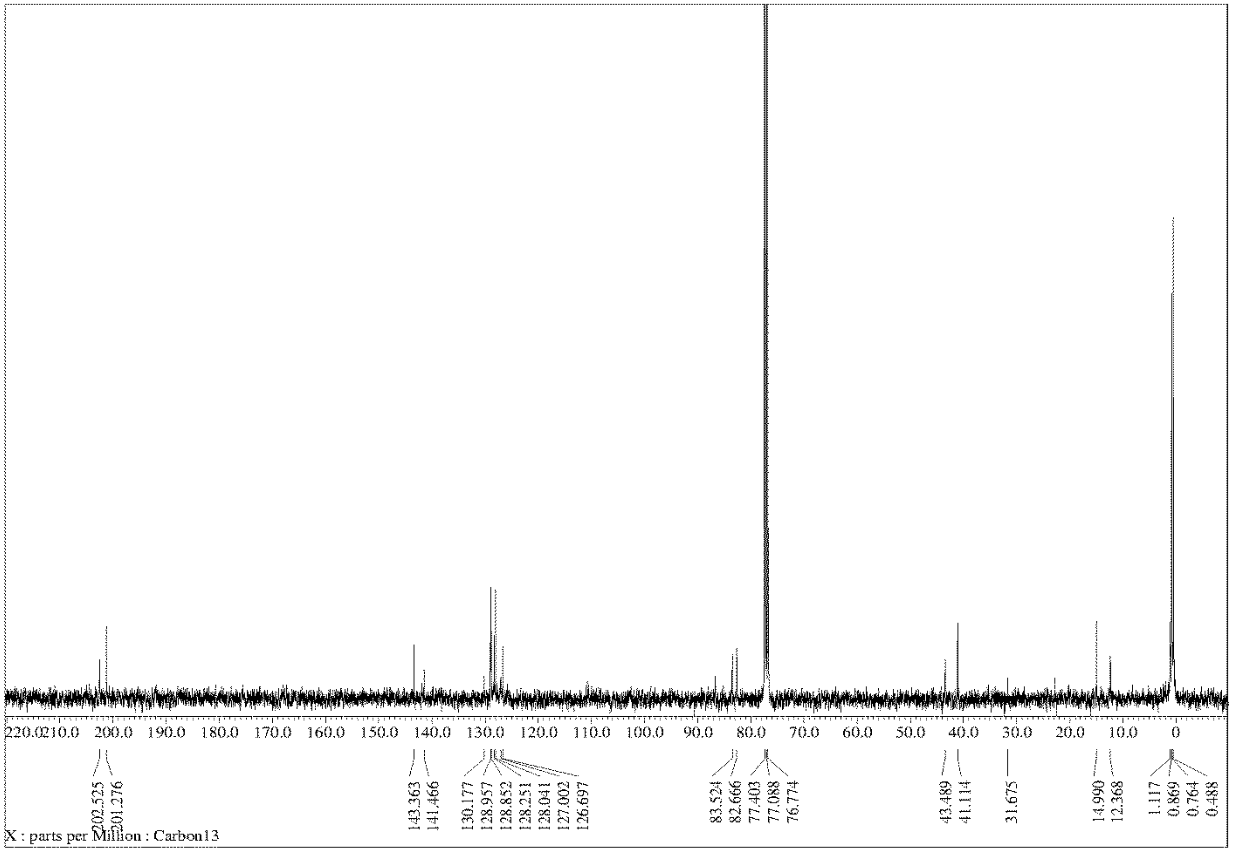
X : parts per Million : Carbon13

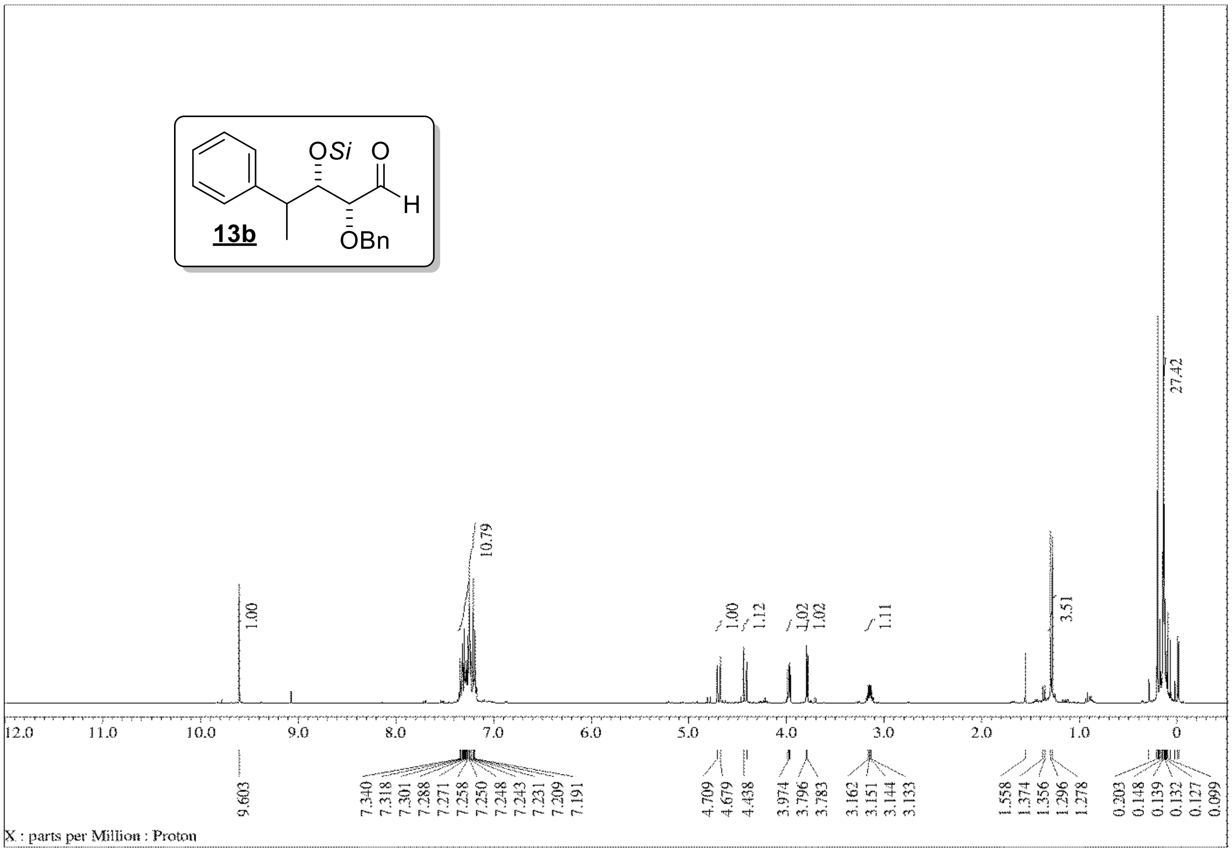
OSi
O
H
OBn
13b
X : parts per Million : Proton
9.603
7.340 7.318 7.301 7.288 7.271 7.258 7.250 7.248 7.243 7.231 7.209 7.191
4.709 4.679 4.438
3.974 3.796 3.783
3.162 3.151 3.144 3.133
1.558 1.374 1.356 1.296 1.278
0.203 0.148 0.139 0.132 0.127 0.099
1.00
10.79
1.00 1.12
1.02 1.02
1.11
3.51
27.42

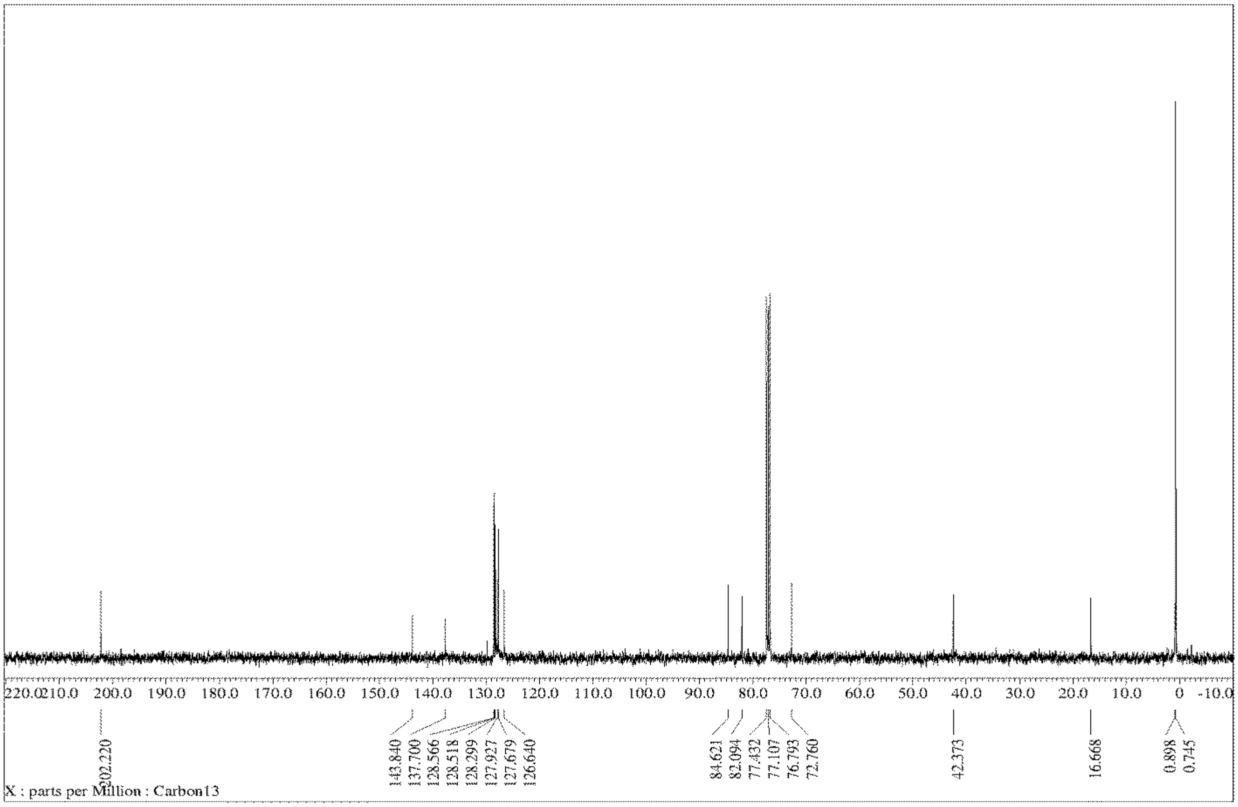
X : parts per Million : Carbon13
202.220
143.840 137.700 128.566 128.518 128.299 127.927 127.679 126.640
84.621 82.094 77.432 77.107 76.793 72.760
42.373
16.668
0.898 0.745

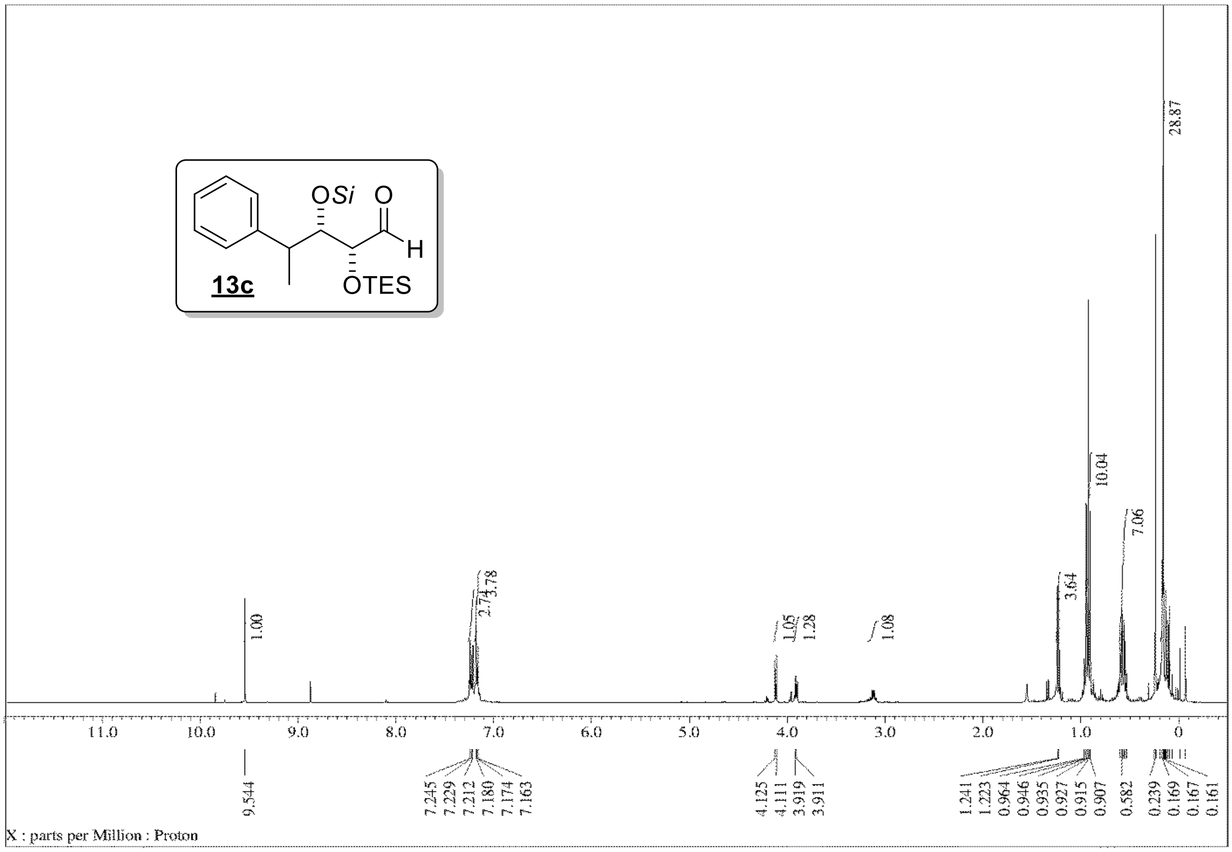
OSi
O
H
OTES
13c
X : parts per Million : Proton

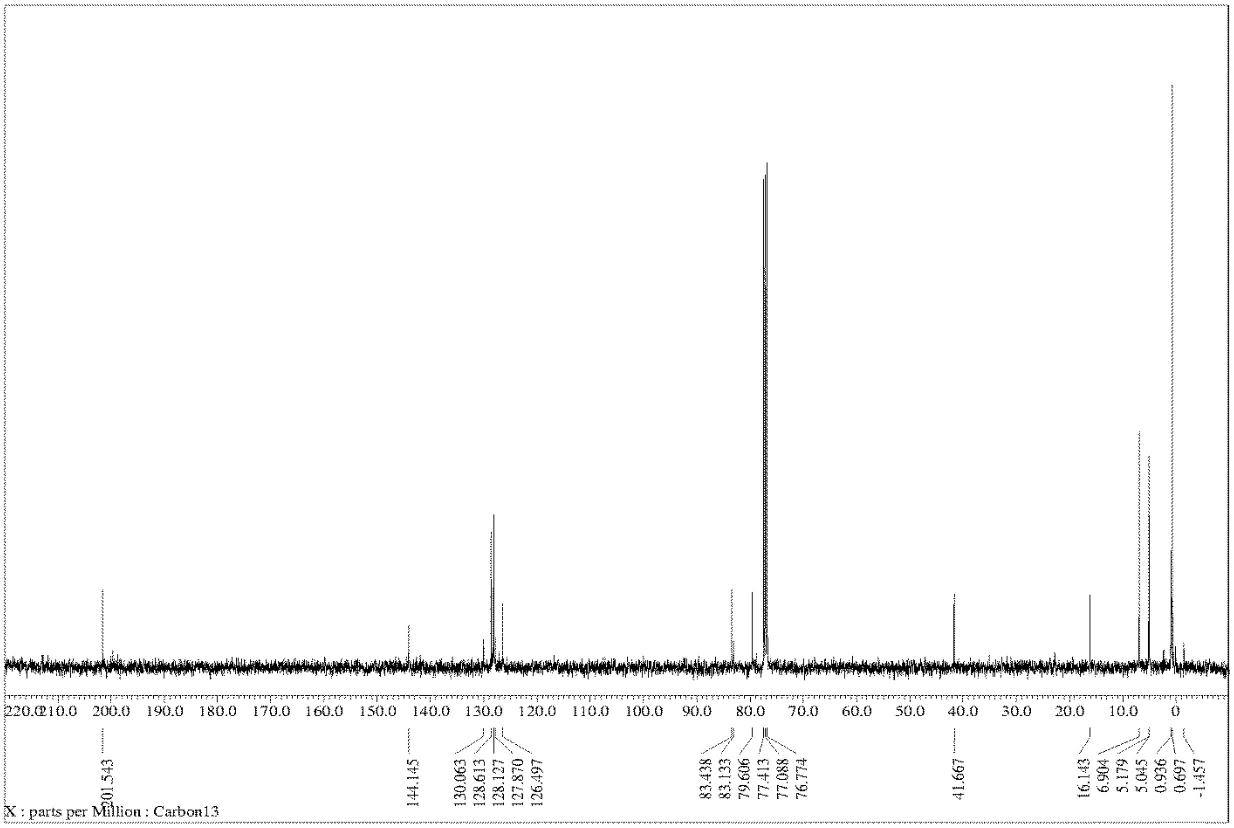
X : parts per Million : Carbon13

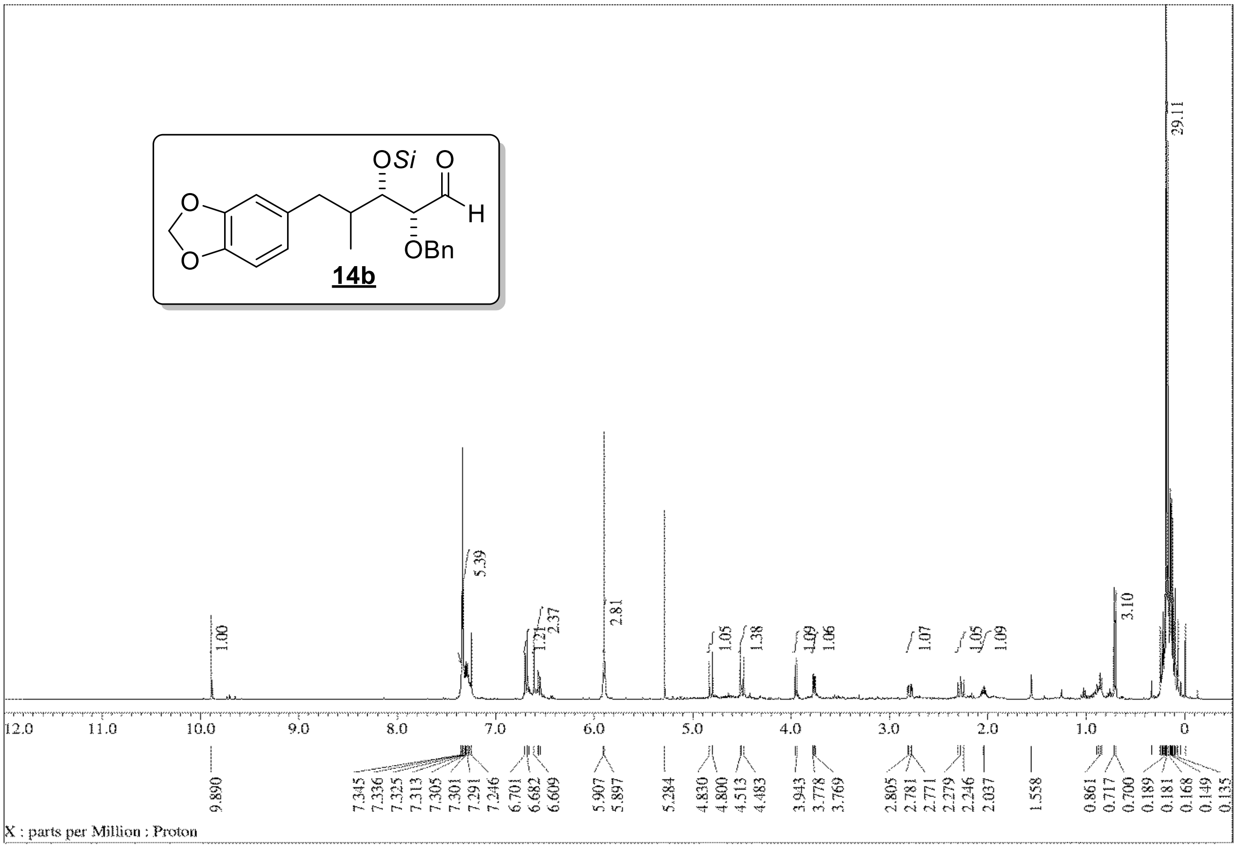
OSi
O
H
O
O
OBn
14b
X : parts per Million : Proton
9.890
7.345
7.336
7.325
7.313
7.305
7.301
7.291
7.246
6.701
6.682
6.609
5.907
5.897
5.284
4.830
4.800
4.513
4.483
3.943
3.778
3.769
2.805
2.781
2.771
2.279
2.246
2.037
1.558
0.861
0.717
0.700
0.189
0.181
0.168
0.149
0.135
1.00
5.39
1.21
2.37
2.81
1.05
1.38
1.09
1.06
1.07
1.05
1.09
3.10
29.11

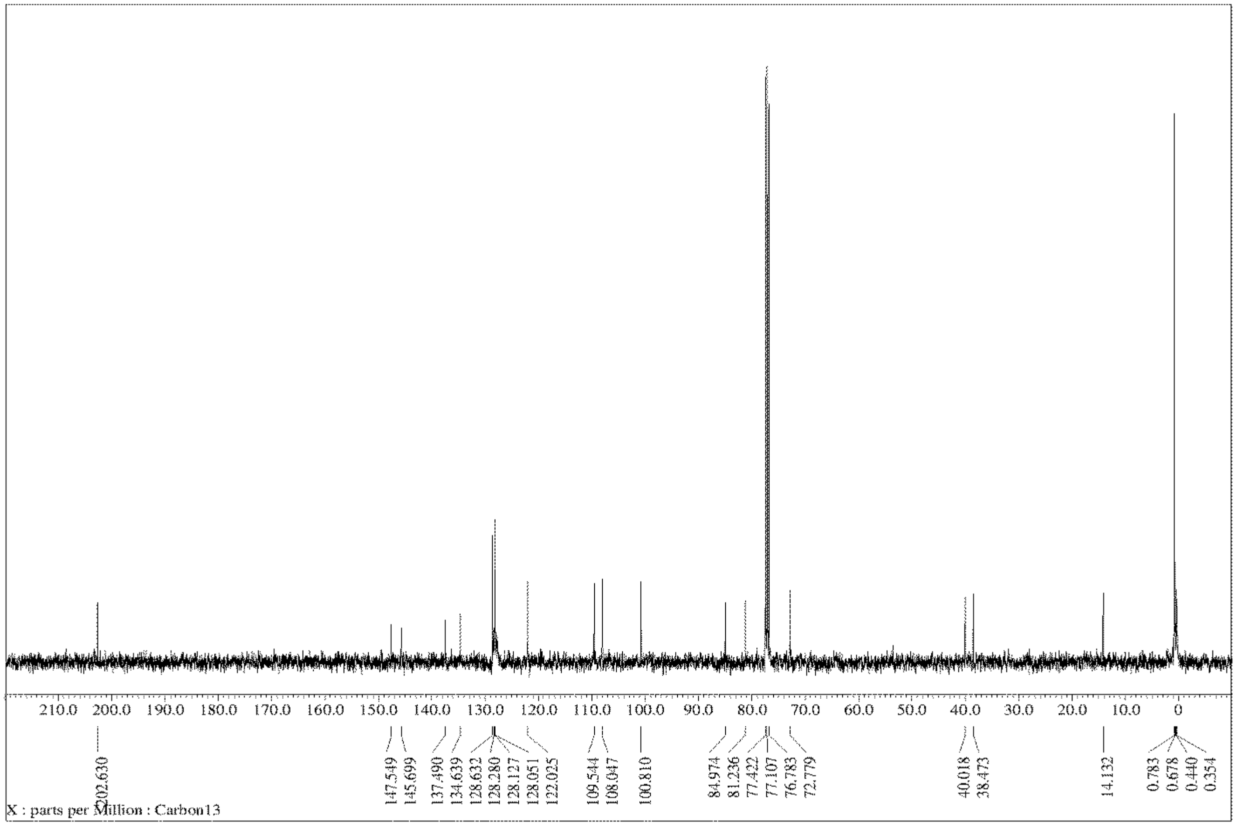
X : parts per Million : Carbon13
202.630
147.549
145.699
137.490
134.639
128.632
128.280
128.127
128.051
122.025
109.544
108.047
100.810
84.974
81.236
77.422
77.107
76.783
72.779
40.018
38.473
14.132
0.783
0.678
0.440
0.354

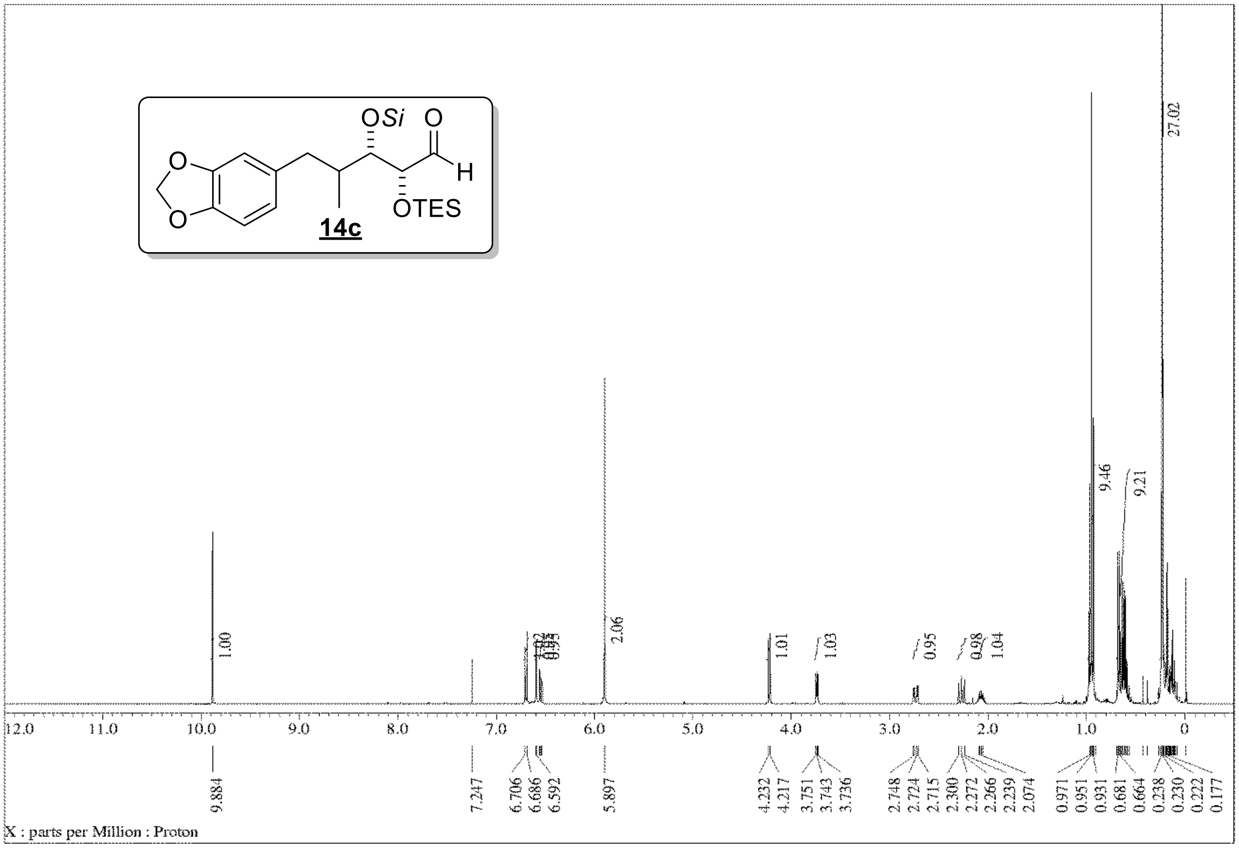
OSi
O
H
OTES
O
O
14c
1.00
2.06
1.01
1.03
0.95
0.98
1.04
9.46
9.21
27.02
12.0 11.0 10.0 9.0 8.0 7.0 6.0 5.0 4.0 3.0 2.0 1.0 0
9.884
7.247
6.706
6.686
6.592
5.897
4.232
4.217
3.751
3.743
3.736
2.748
2.724
2.715
2.300
2.272
2.266
2.239
2.074
0.971
0.951
0.931
0.681
0.664
0.238
0.230
0.222
0.177
X : parts per Million : Proton

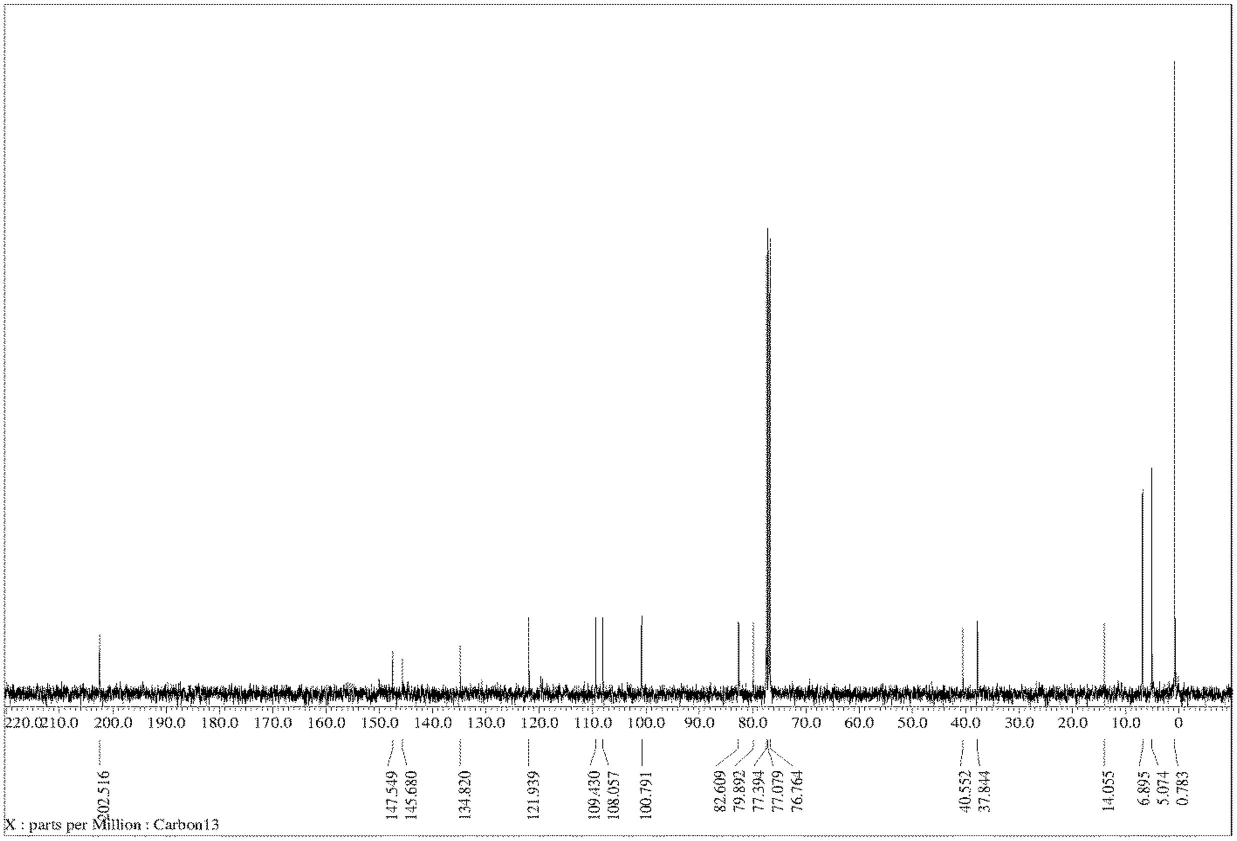
220.0 210.0 200.0 190.0 180.0 170.0 160.0 150.0 140.0 130.0 120.0 110.0 100.0 90.0 80.0 70.0 60.0 50.0 40.0 30.0 20.0 10.0 0
202.516
147.549
145.680
134.820
121.939
109.430
108.057
100.791
82.609
79.892
77.394
77.079
76.764
40.552
37.844
14.055
6.895
5.074
0.783
X : parts per Million : Carbon13

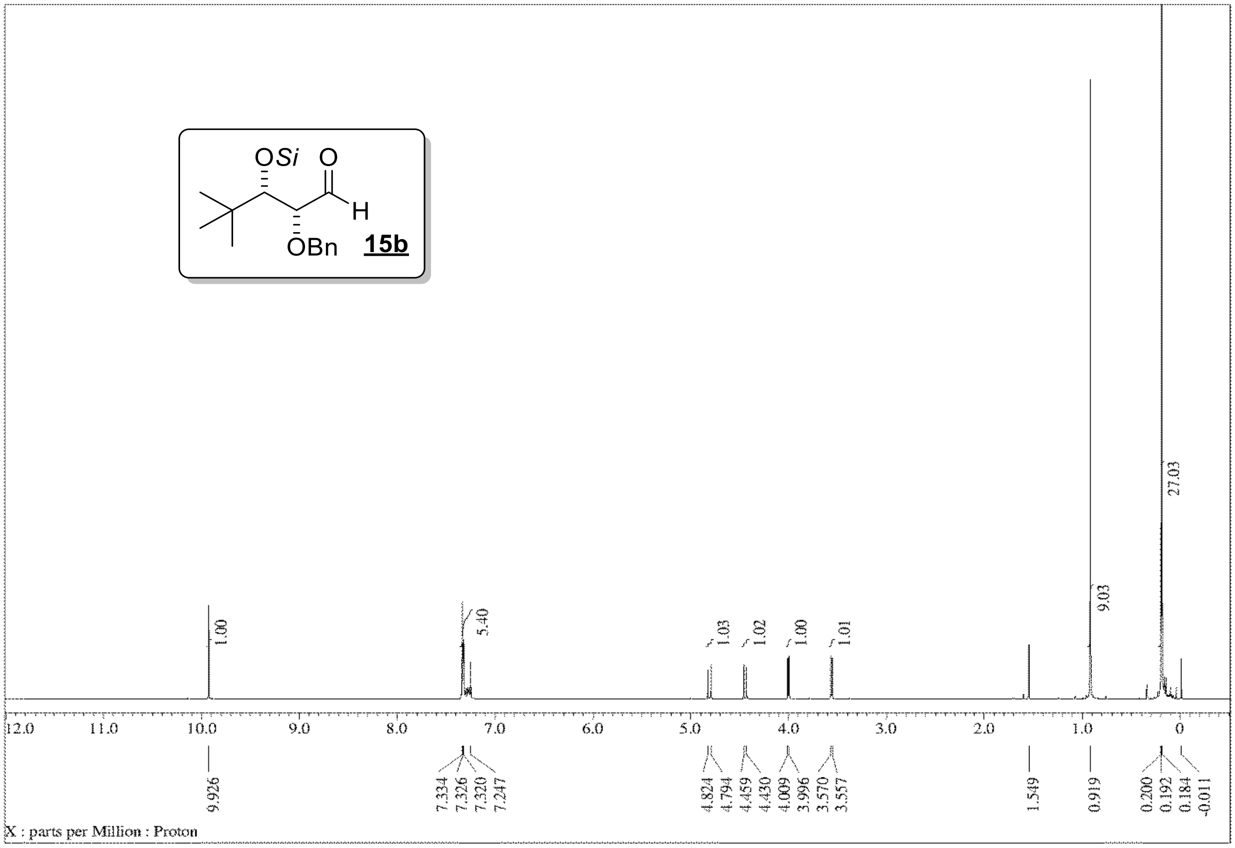
OSi
O
H
OBn
15b
1.00
5.40
1.03
1.02
1.00
1.01
9.03
27.03
12.0 11.0 10.0 9.0 8.0 7.0 6.0 5.0 4.0 3.0 2.0 1.0 0
9.926
7.334
7.326
7.320
7.247
4.824
4.794
4.459
4.430
4.009
3.996
3.570
3.557
1.549
0.919
0.200
0.192
0.184
-0.011
X : parts per Million : Proton

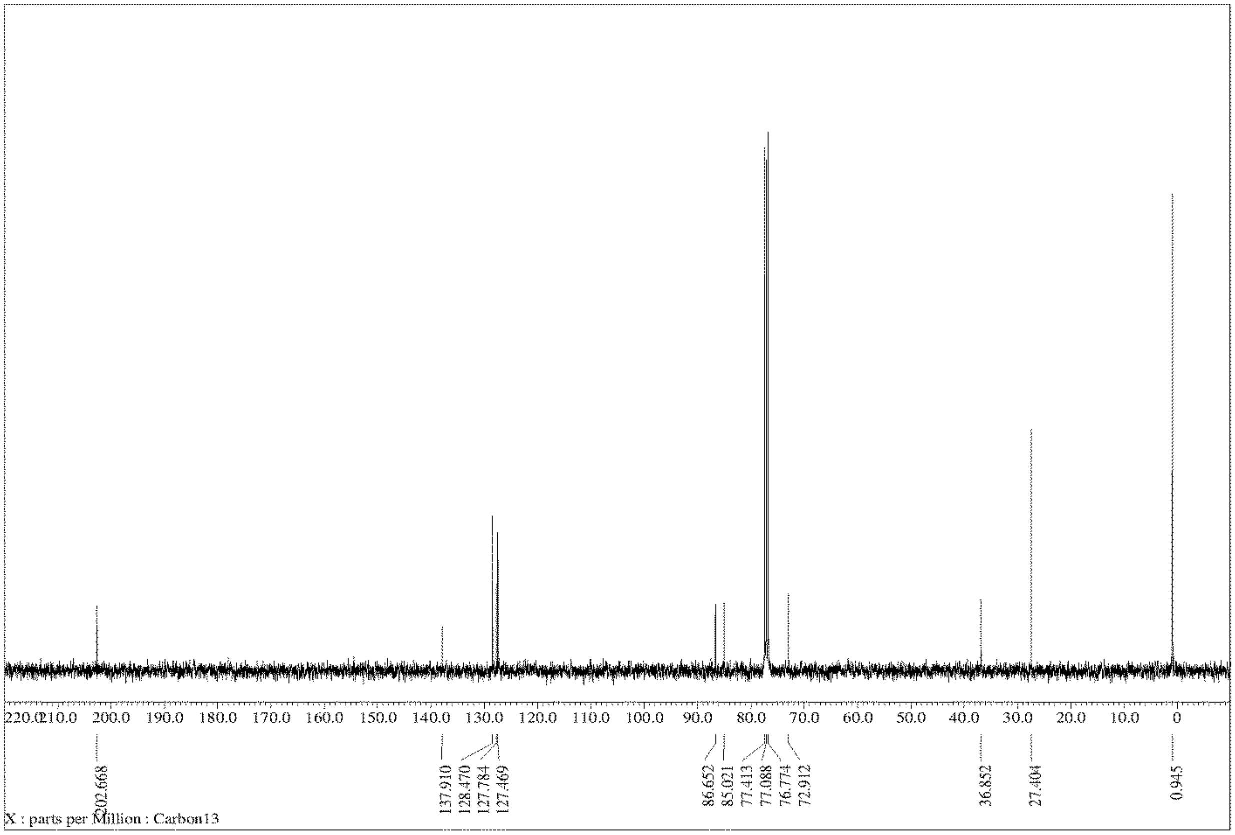
220.0 210.0 200.0 190.0 180.0 170.0 160.0 150.0 140.0 130.0 120.0 110.0 100.0 90.0 80.0 70.0 60.0 50.0 40.0 30.0 20.0 10.0 0
202.668
137.910
128.470
127.784
127.469
86.652
85.021
77.413
77.088
76.774
72.912
36.852
27.404
0.945
X : parts per Million : Carbon13

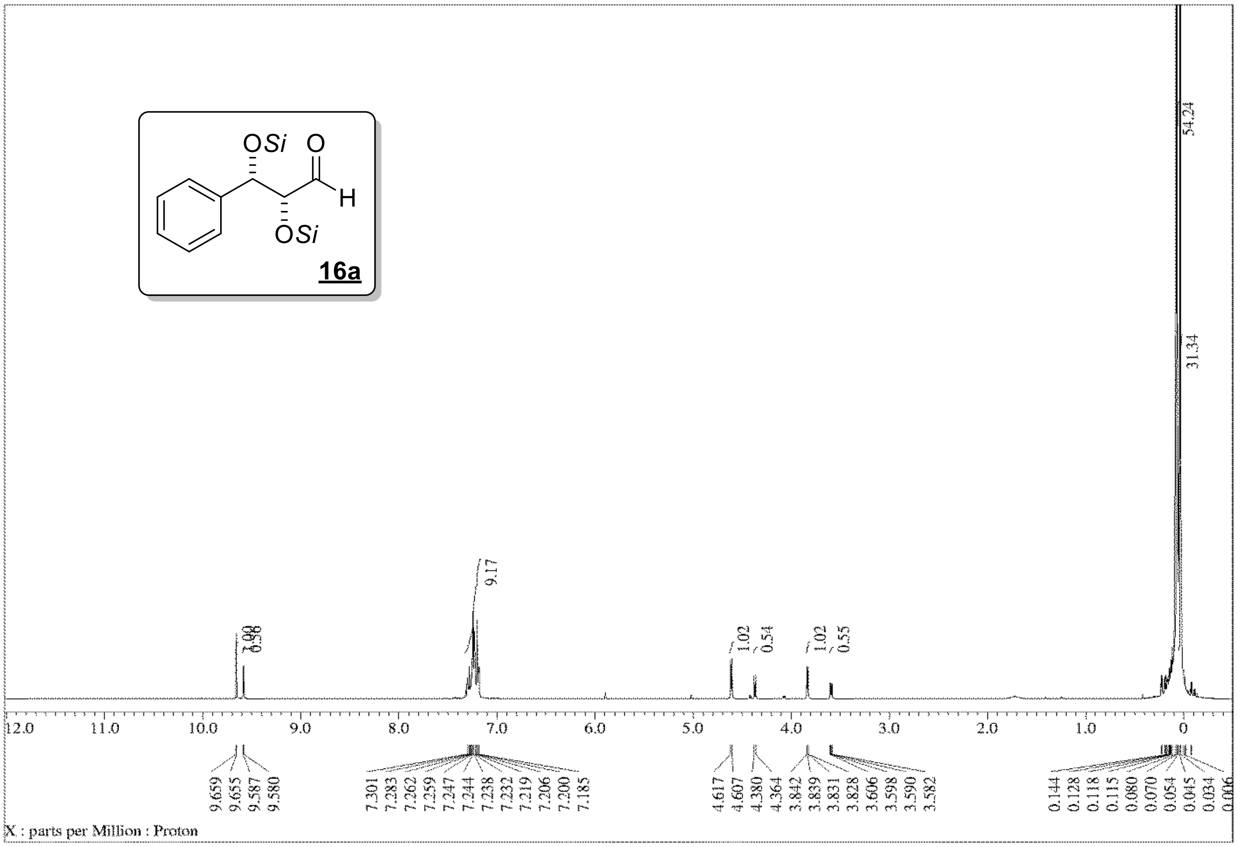
OSi
O
H
OSi
16a
9.17
1.02
0.54
1.02
0.55
54.24
31.34
12.0 11.0 10.0 9.0 8.0 7.0 6.0 5.0 4.0 3.0 2.0 1.0 0
9.659 9.655 9.587 9.580
7.301 7.283 7.262 7.259 7.247 7.244 7.238 7.232 7.219 7.206 7.200 7.185
4.617 4.607 4.380 4.364 3.842 3.839 3.831 3.828 3.606 3.598 3.590 3.582
0.144 0.128 0.118 0.115 0.080 0.070 0.054 0.045 0.034 0.006
X : parts per Million : Proton

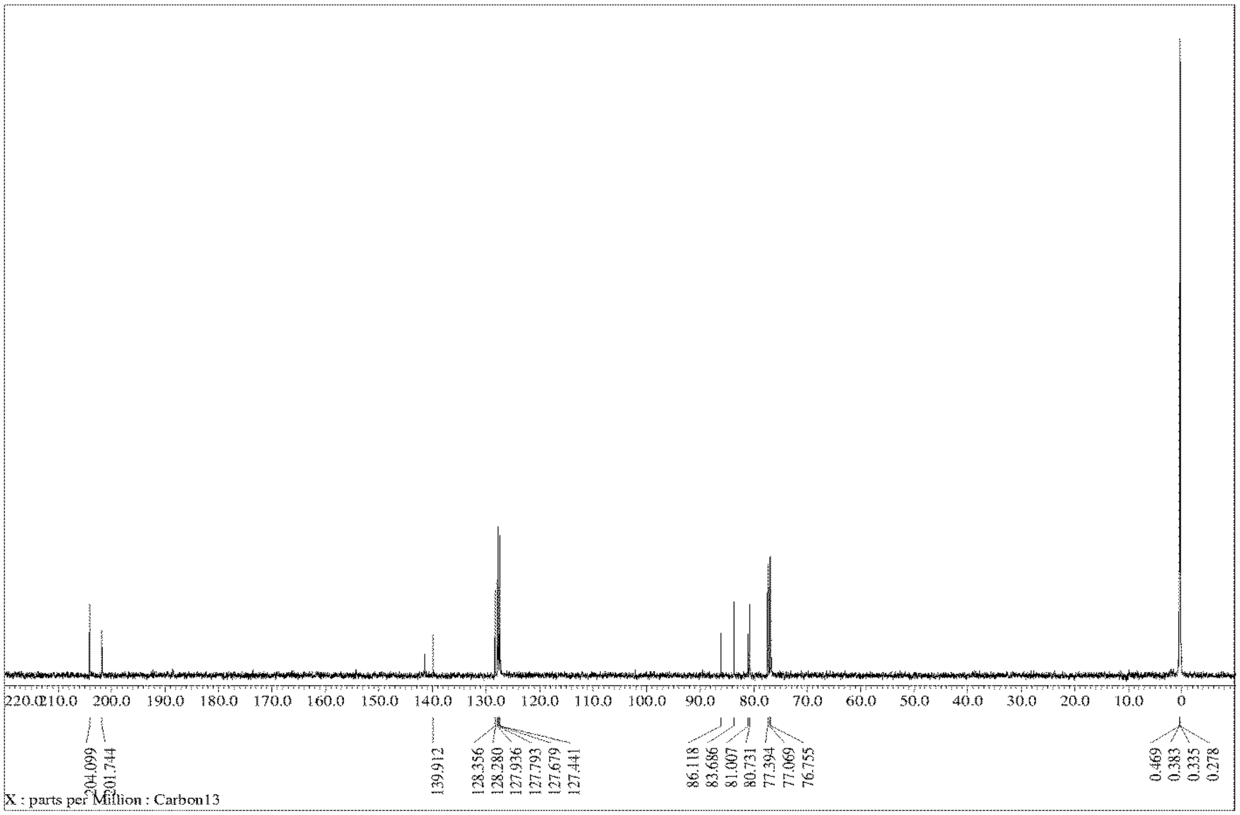
220.0 210.0 200.0 190.0 180.0 170.0 160.0 150.0 140.0 130.0 120.0 110.0 100.0 90.0 80.0 70.0 60.0 50.0 40.0 30.0 20.0 10.0 0
204.099 201.744
139.912
128.356 128.280 127.936 127.793 127.679 127.441
86.118 83.686 81.007 80.731 77.394 77.069 76.755
0.469 0.383 0.335 0.278
X : parts per Million : Carbon13

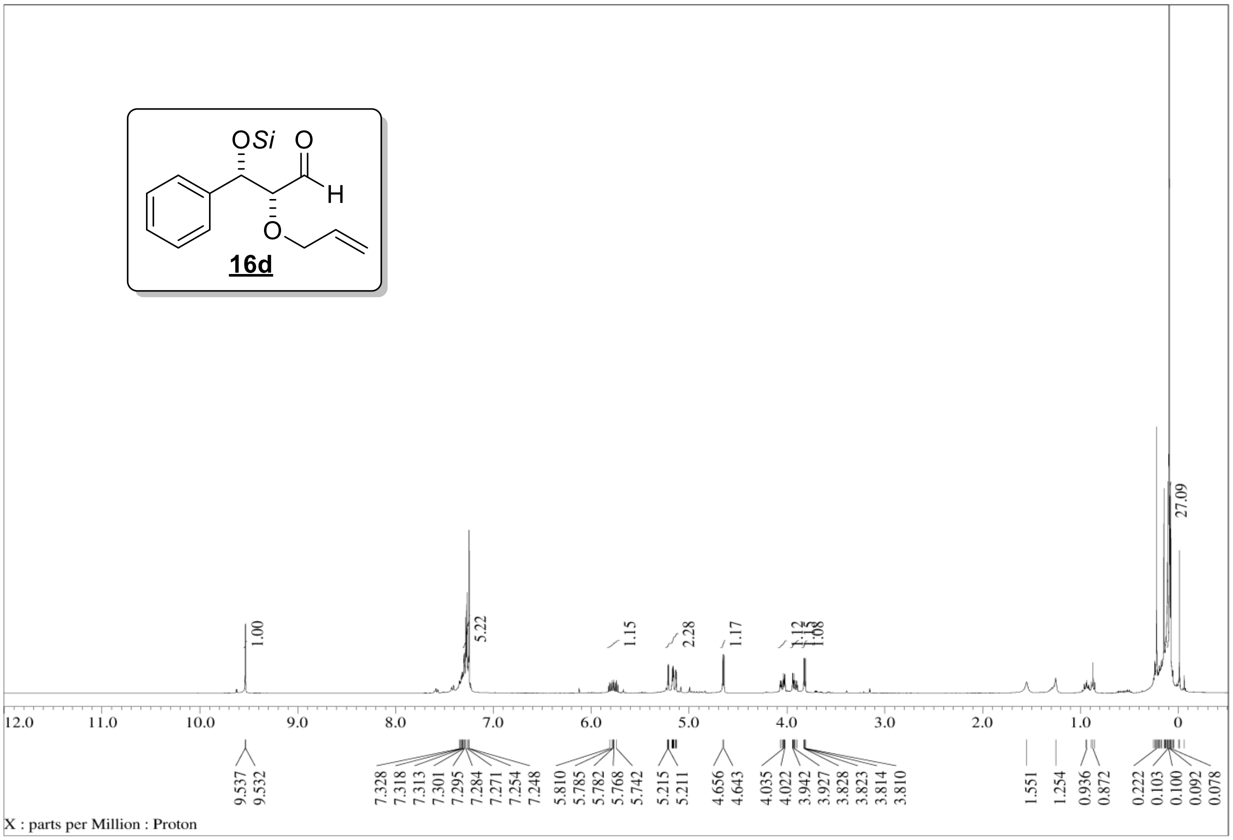
OSi
O
H
O
16d
1.00
5.22
1.15
2.28
1.17
1.12
1.15
1.08
27.09
12.0 11.0 10.0 9.0 8.0 7.0 6.0 5.0 4.0 3.0 2.0 1.0 0
9.537
9.532
7.328
7.318
7.313
7.301
7.295
7.284
7.271
7.254
7.248
5.810
5.785
5.782
5.768
5.742
5.215
5.211
4.656
4.643
4.035
4.022
3.942
3.927
3.828
3.823
3.814
3.810
1.551
1.254
0.936
0.872
0.222
0.103
0.100
0.092
0.078
X : parts per Million : Proton

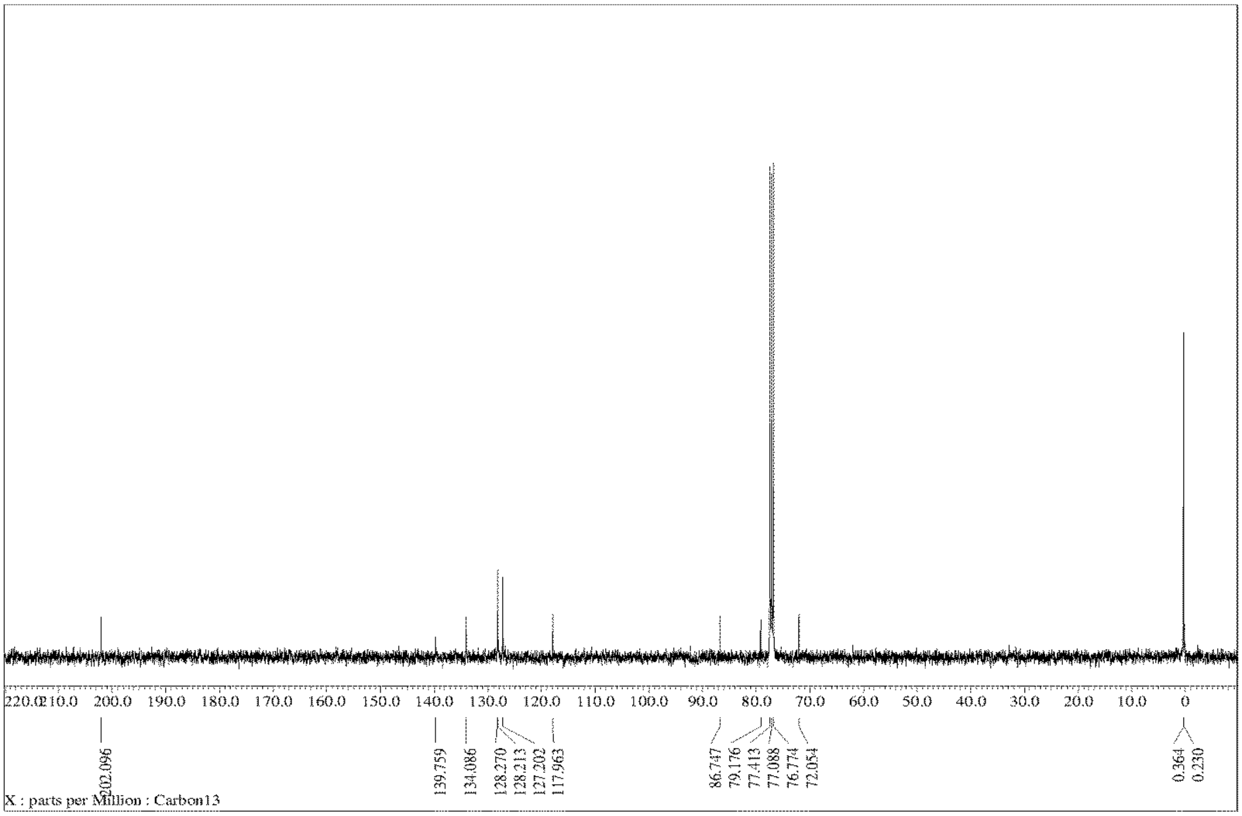
220.0 210.0 200.0 190.0 180.0 170.0 160.0 150.0 140.0 130.0 120.0 110.0 100.0 90.0 80.0 70.0 60.0 50.0 40.0 30.0 20.0 10.0 0
202.096
139.759
134.086
128.270
128.213
127.202
117.963
86.747
79.176
77.413
77.088
76.774
72.054
0.364
0.230
X : parts per Million : Carbon13

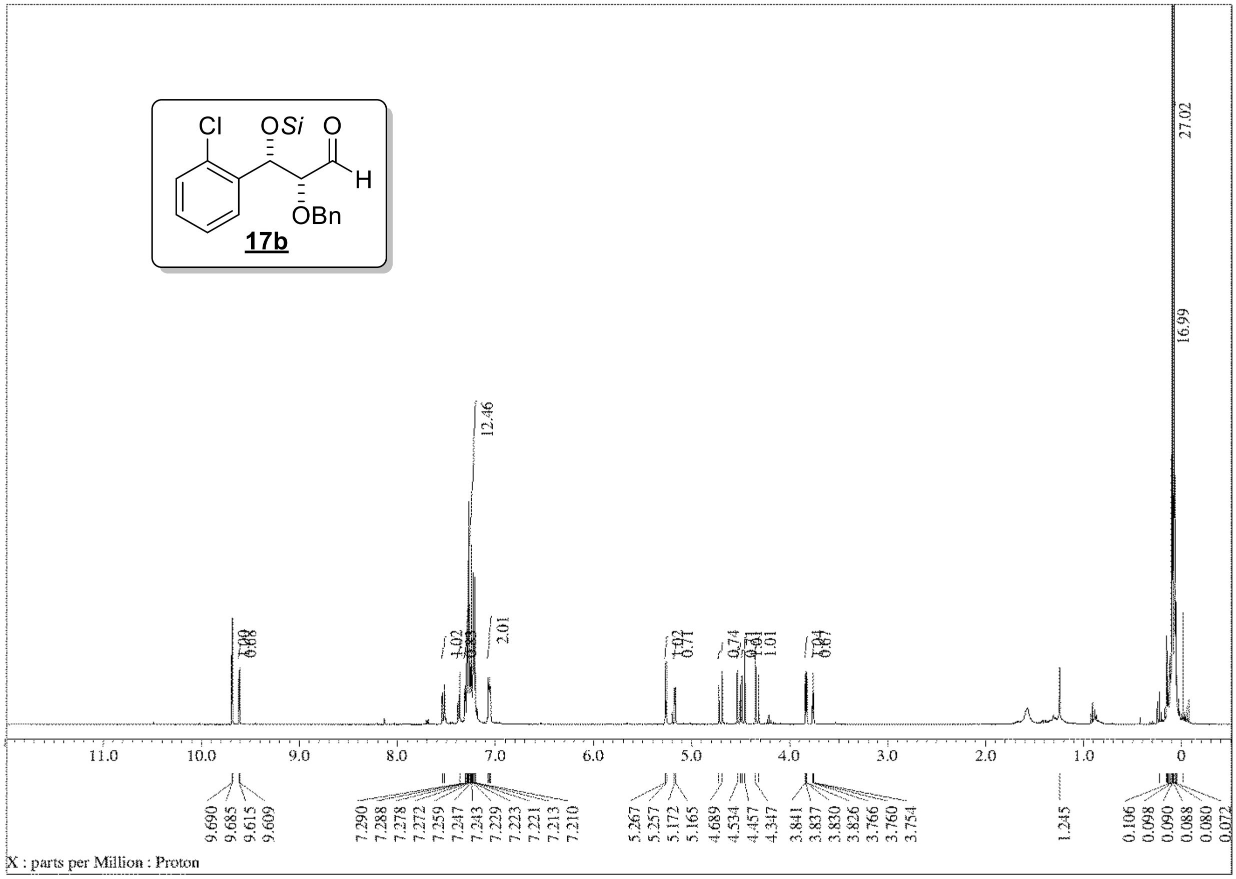
Cl
OSi
O
H
OBn
17b
27.02
16.99
12.46
2.01
X : parts per Million : Proton
9.690
9.685
9.615
9.609
7.290
7.288
7.278
7.272
7.259
7.247
7.243
7.229
7.223
7.221
7.213
7.210
5.267
5.257
5.172
5.165
4.689
4.534
4.457
4.347
3.841
3.837
3.830
3.826
3.766
3.760
3.754
1.245
0.106
0.098
0.090
0.088
0.080
0.072

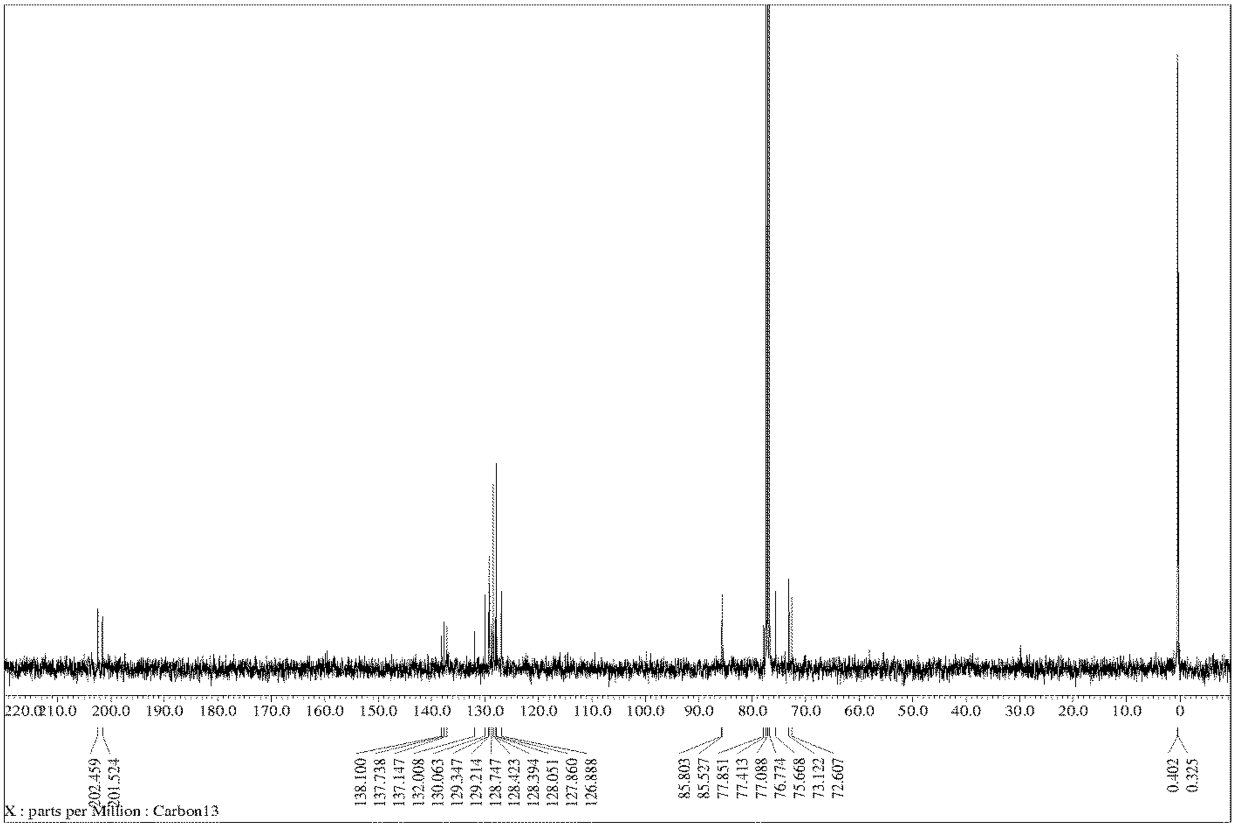
X : parts per Million : Carbon13
202.459
201.524
138.100
137.738
137.147
132.008
130.063
129.347
129.214
128.747
128.423
128.394
128.051
127.860
126.888
85.803
85.527
77.851
77.413
77.088
76.774
75.668
73.122
72.607
0.402
0.325

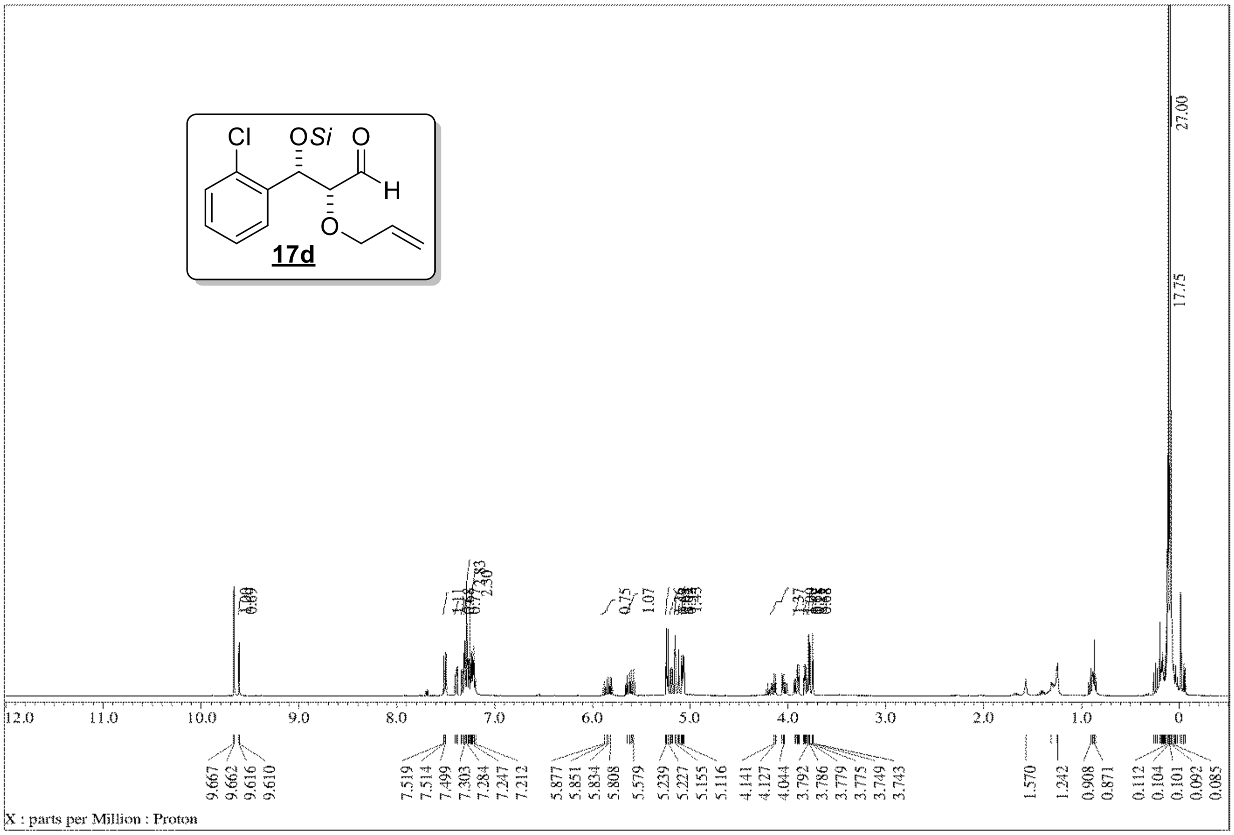
Cl
OSi
O
H
O
17d
27.00
17.75
1.07
9.667
9.662
9.616
9.610
7.519
7.514
7.499
7.303
7.284
7.247
7.212
5.877
5.851
5.834
5.808
5.579
5.239
5.227
5.155
5.116
4.141
4.127
4.044
3.792
3.786
3.779
3.775
3.749
3.743
1.570
1.242
0.908
0.871
0.112
0.104
0.101
0.092
0.085
X : parts per Million : Proton

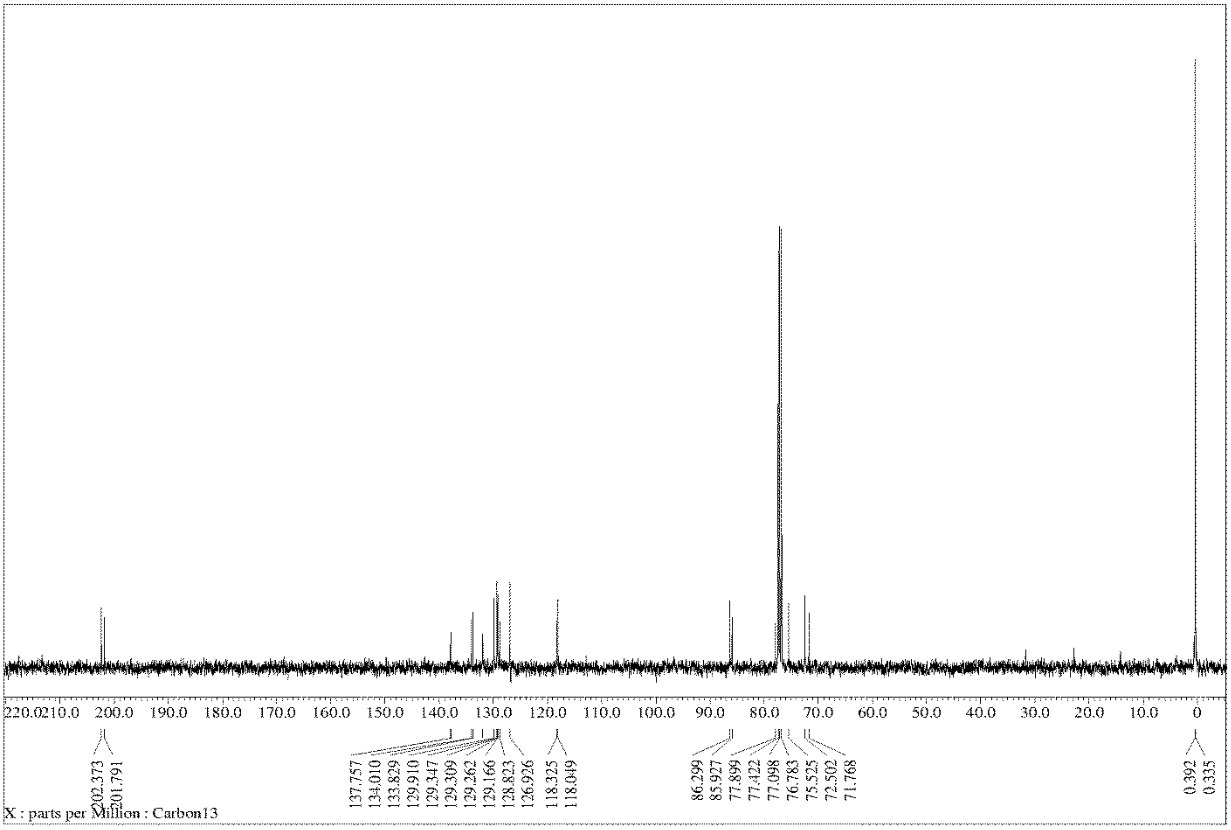
202.373
201.791
137.757
134.010
133.829
129.910
129.347
129.309
129.262
129.166
128.823
126.926
118.325
118.049
86.299
85.927
77.899
77.422
77.098
76.783
75.525
72.502
71.768
0.392
0.335
X : parts per Million : Carbon13

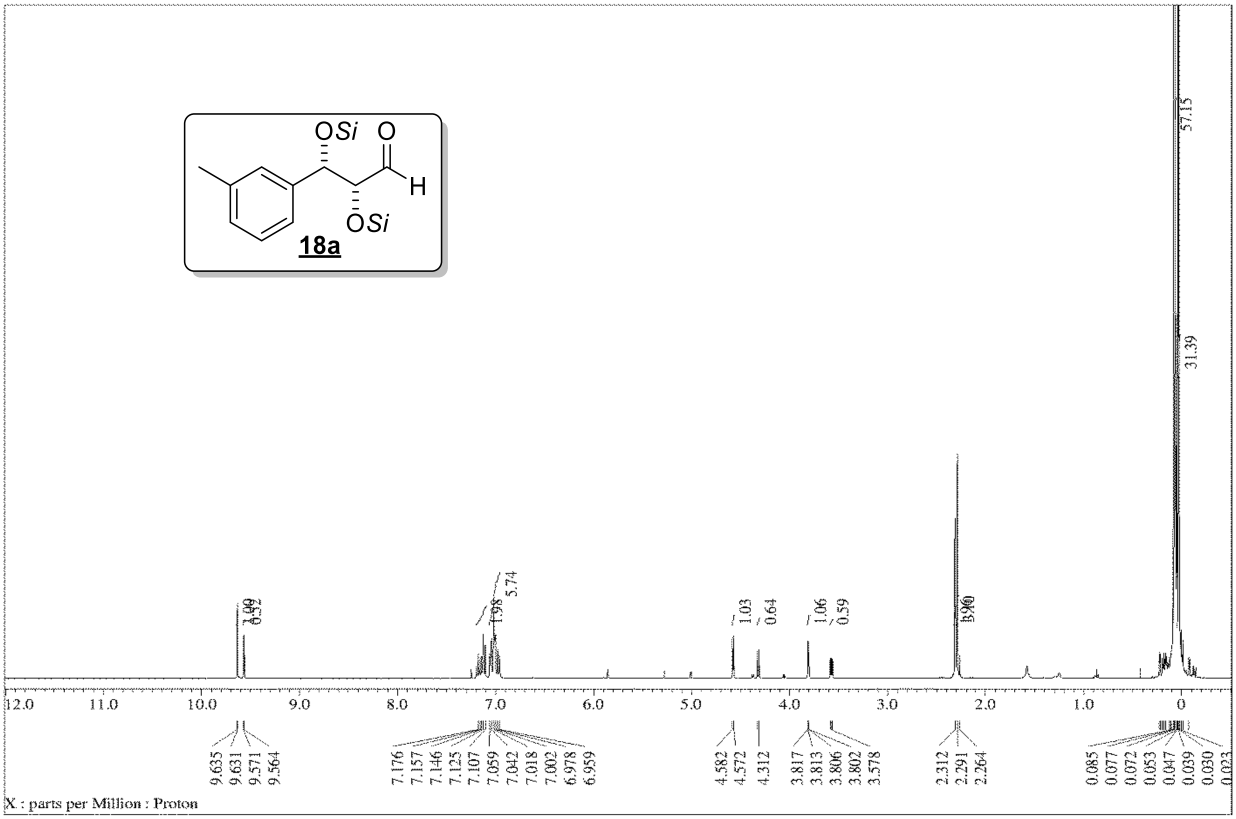

OSi
O
H
OSi
18a
57.15
31.39
5.74
1.98
1.03
0.64
1.06
0.59
12.0 11.0 10.0 9.0 8.0 7.0 6.0 5.0 4.0 3.0 2.0 1.0 0
9.635 9.631 9.571 9.564
7.176 7.157 7.146 7.125 7.107 7.059 7.042 7.018 7.002 6.978 6.959
4.582 4.572 4.312
3.817 3.813 3.806 3.802 3.578
2.312 2.291 2.264
0.085 0.077 0.072 0.053 0.047 0.039 0.030
X : parts per Million : Proton

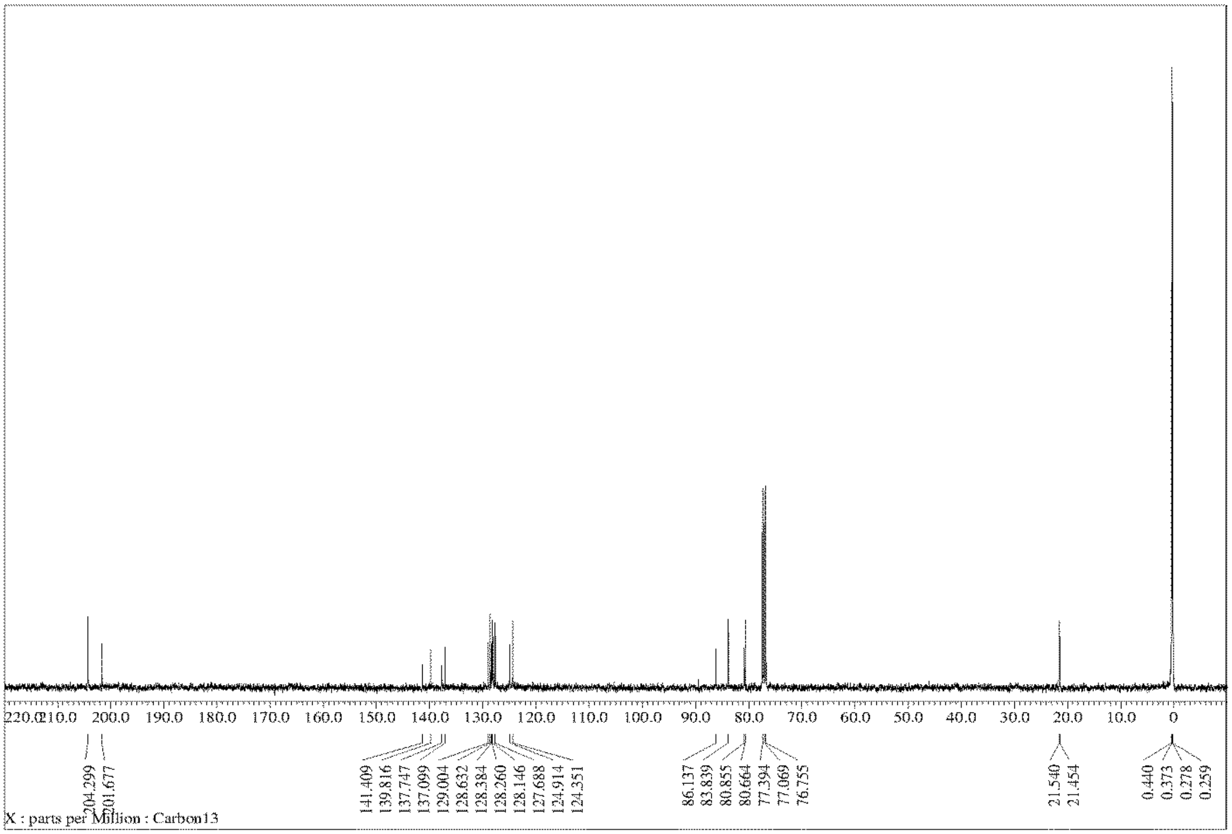

220.0 210.0 200.0 190.0 180.0 170.0 160.0 150.0 140.0 130.0 120.0 110.0 100.0 90.0 80.0 70.0 60.0 50.0 40.0 30.0 20.0 10.0 0
204.299
141.409 139.816 137.747 137.099 129.004 128.632 128.384 128.260 128.146 127.688 124.914 124.351
86.137 83.839 80.855 80.664 77.394 77.069 76.755
21.540 21.454
0.440 0.373 0.278 0.259
X : parts per Million : Carbon13

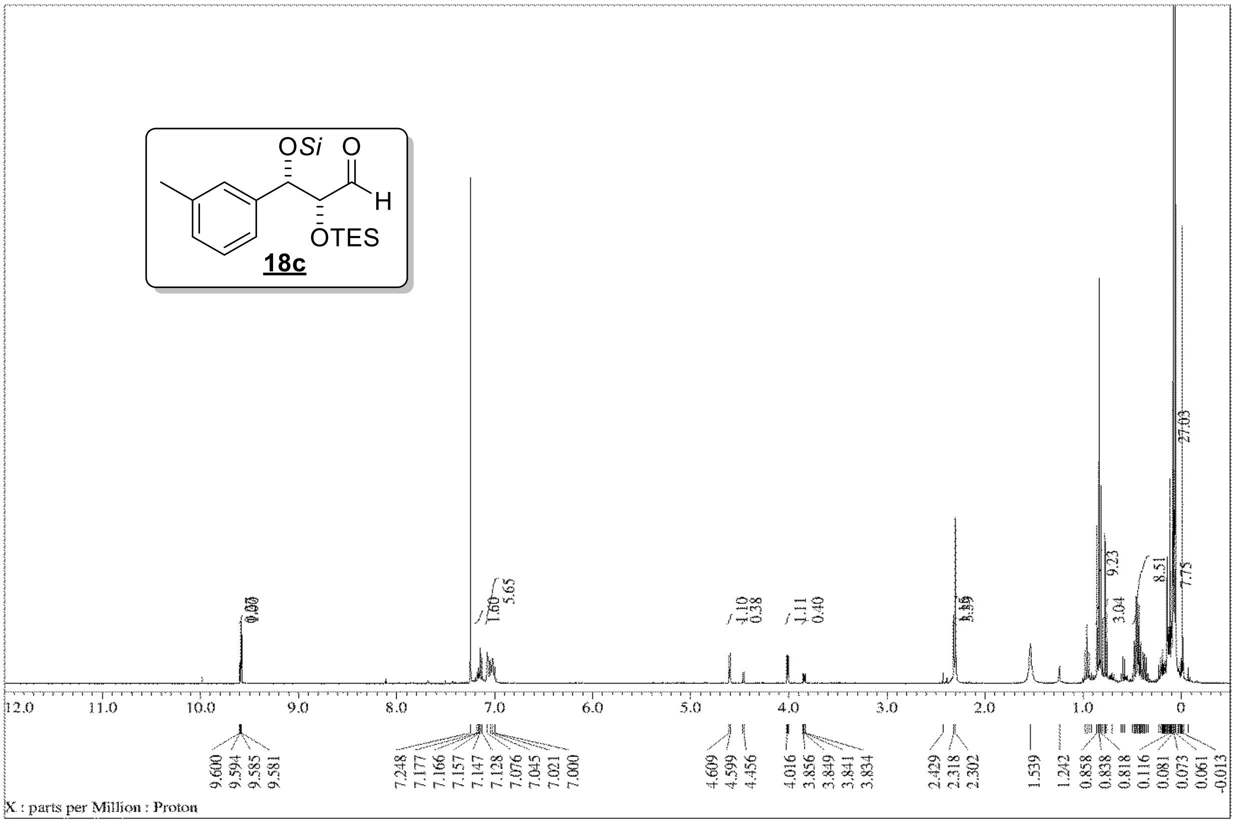
OSi
O
H
OTES
18c
X : parts per Million : Proton
9.600 9.594 9.585 9.581
7.248 7.177 7.166 7.157 7.147 7.128 7.076 7.045 7.021 7.000
4.609 4.599 4.456
4.016 3.856 3.849 3.841 3.834
2.429 2.318 2.302
1.539
1.242 0.858 0.838 0.818 0.116 0.081 0.073 0.061 -0.013

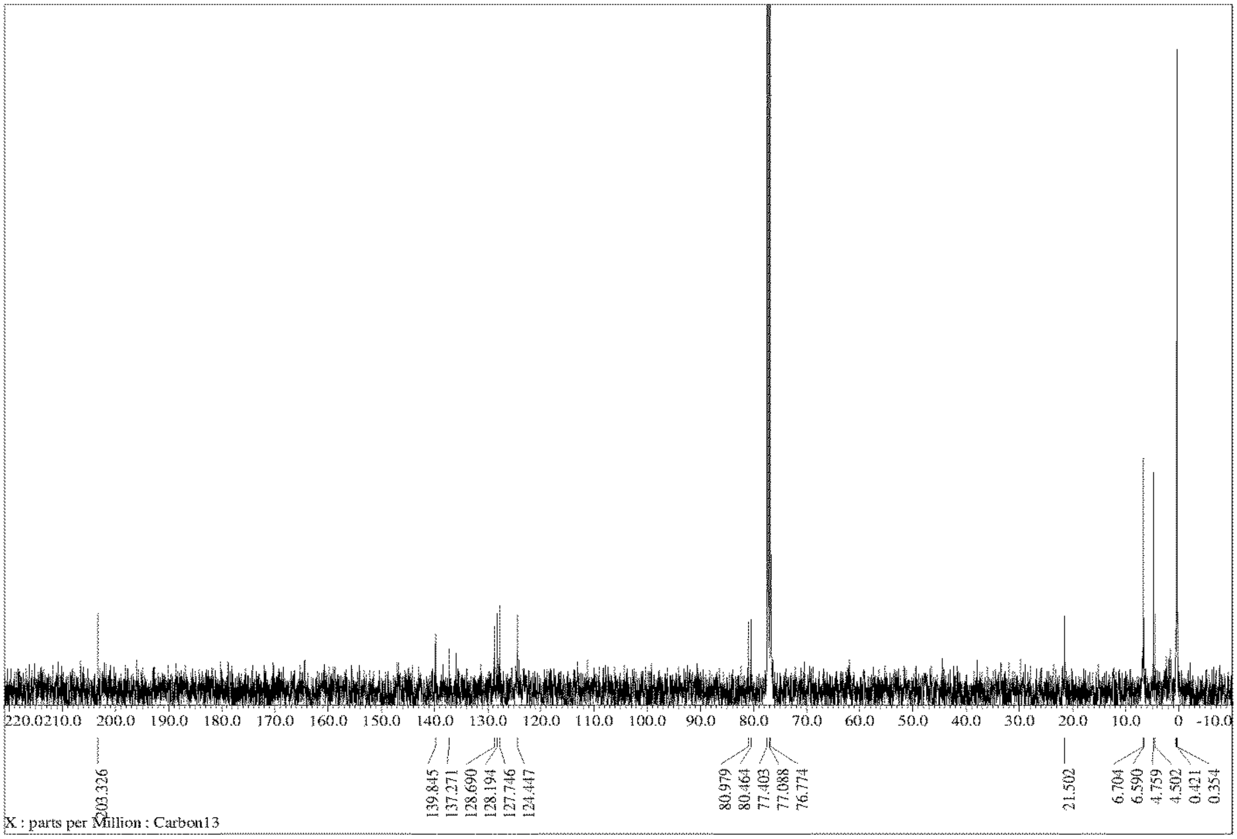
X : parts per Million : Carbon13
203.326
139.845 137.271 128.690 128.194 127.746 124.447
80.979 80.464 77.403 77.088 76.774
21.502
6.704 6.590 4.759 4.502 0.421 0.354

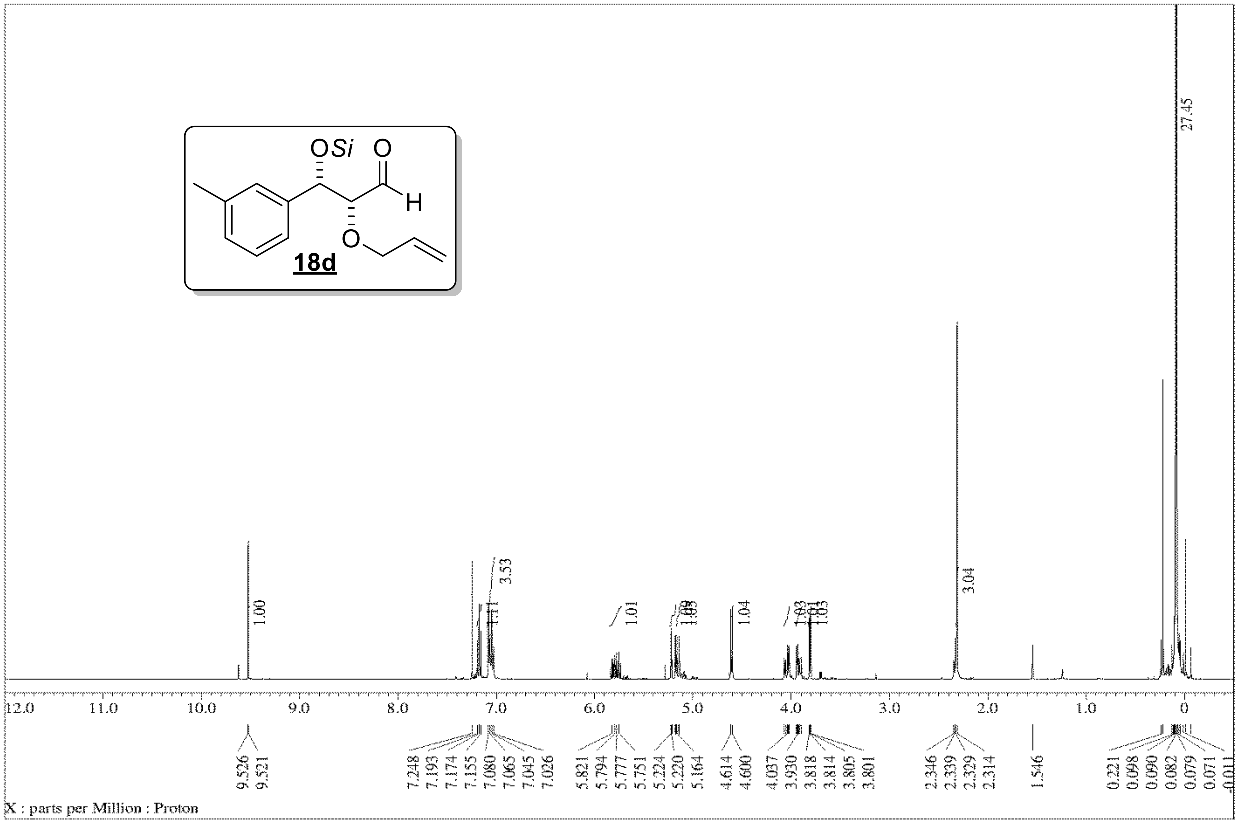
OSi
O
H
O
18d
27.45
3.53
1.00
1.11
1.01
1.04
3.04
9.526
9.521
7.248
7.193
7.174
7.155
7.080
7.065
7.045
7.026
5.821
5.794
5.777
5.751
5.224
5.220
5.164
4.614
4.600
4.037
3.930
3.818
3.814
3.805
3.801
2.346
2.339
2.329
2.314
1.546
0.221
0.098
0.090
0.082
0.079
0.071
-0.011
X : parts per Million : Proton

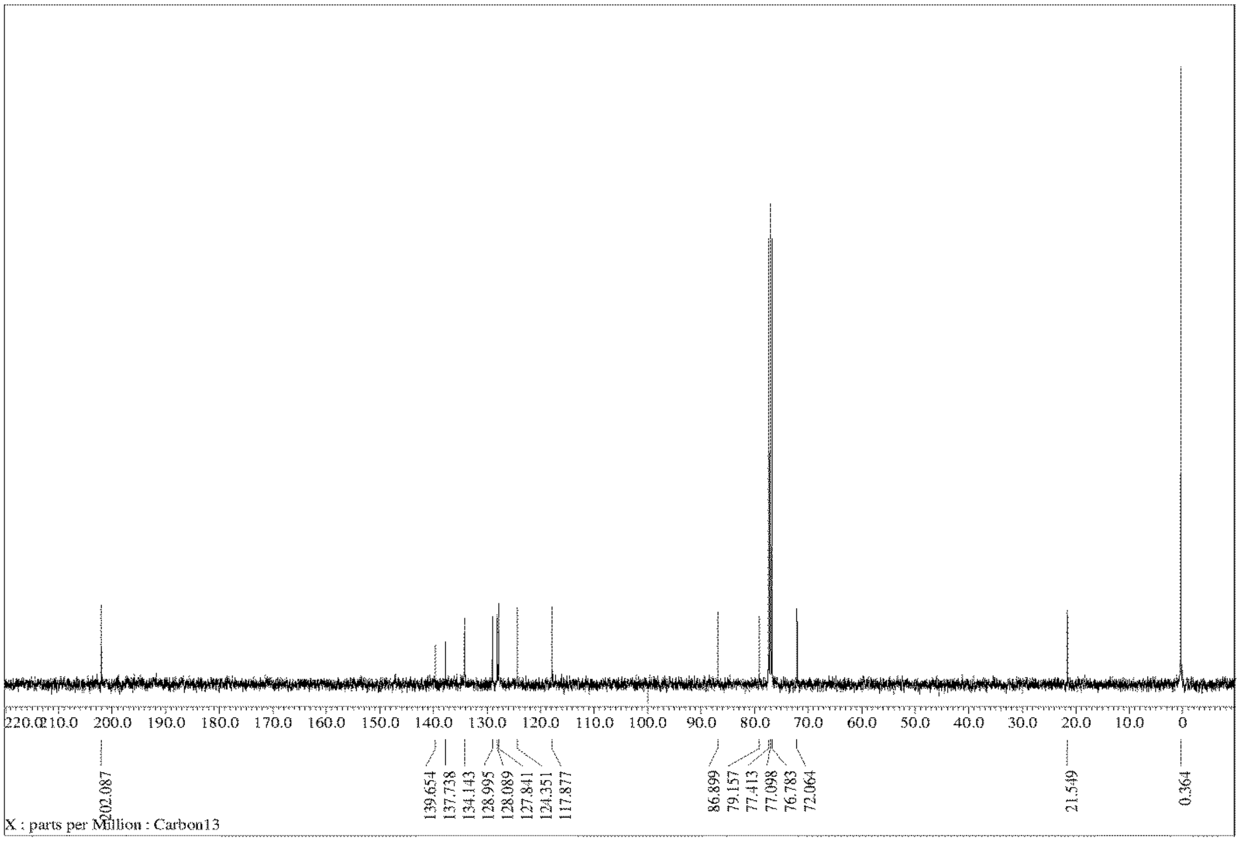
202.087
139.654
137.738
134.143
128.995
128.089
127.841
124.351
117.877
86.899
79.157
77.413
77.098
76.783
72.064
21.549
0.364
X : parts per Million : Carbon13

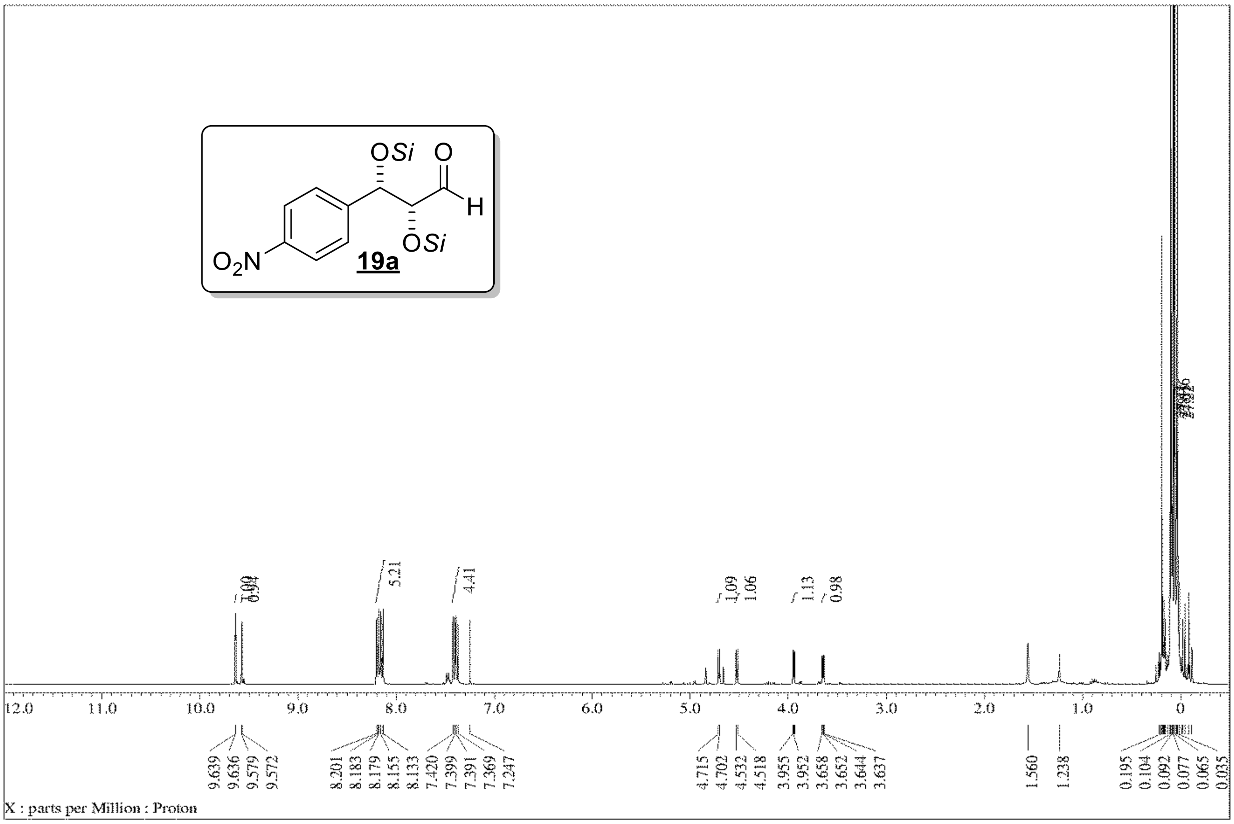
OSi
O
H
OSi
O2N
19a
9.639
9.636
9.579
9.572
8.201
8.183
8.179
8.155
8.133
7.420
7.399
7.391
7.369
7.247
4.715
4.702
4.532
4.518
3.955
3.952
3.658
3.652
3.644
3.637
1.560
1.238
0.195
0.104
0.092
0.077
0.065
0.035
5.21
4.41
1.09
1.06
1.13
0.98
12.0 11.0 10.0 9.0 8.0 7.0 6.0 5.0 4.0 3.0 2.0 1.0 0
X : parts per Million : Proton

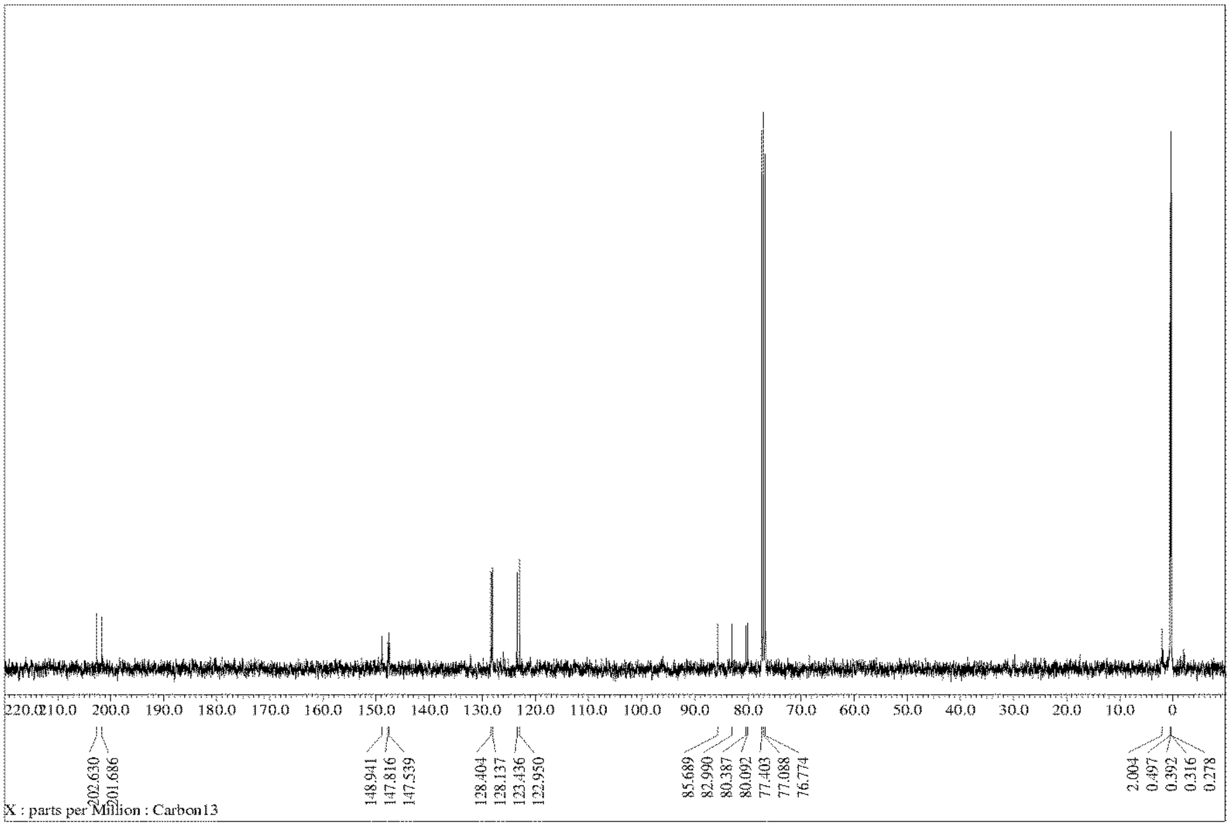
202.630
201.686
148.941
147.816
147.539
128.404
128.137
123.436
122.950
85.689
82.990
80.387
80.092
77.403
77.088
76.774
2.004
0.497
0.392
0.316
0.278
220.0 210.0 200.0 190.0 180.0 170.0 160.0 150.0 140.0 130.0 120.0 110.0 100.0 90.0 80.0 70.0 60.0 50.0 40.0 30.0 20.0 10.0 0
X : parts per Million : Carbon13

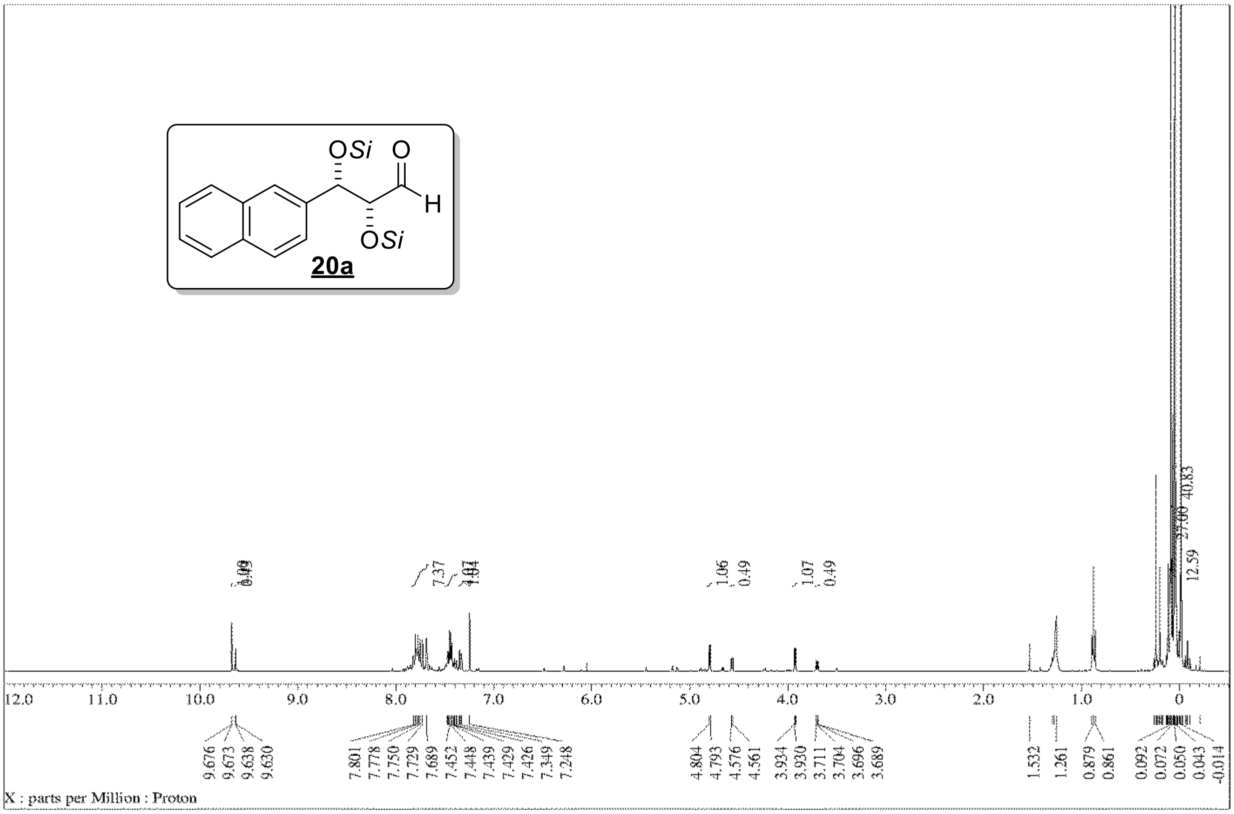
OSi
O
H
OSi
20a
7.37
1.06
0.49
1.07
0.49
12.59
27.00
40.83
9.676
9.673
9.638
9.630
7.801
7.778
7.750
7.729
7.689
7.452
7.448
7.439
7.429
7.426
7.349
7.248
4.804
4.793
4.576
4.561
3.934
3.930
3.711
3.704
3.696
3.689
1.532
1.261
0.879
0.861
0.092
0.072
0.050
0.043
-0.014
X : parts per Million : Proton

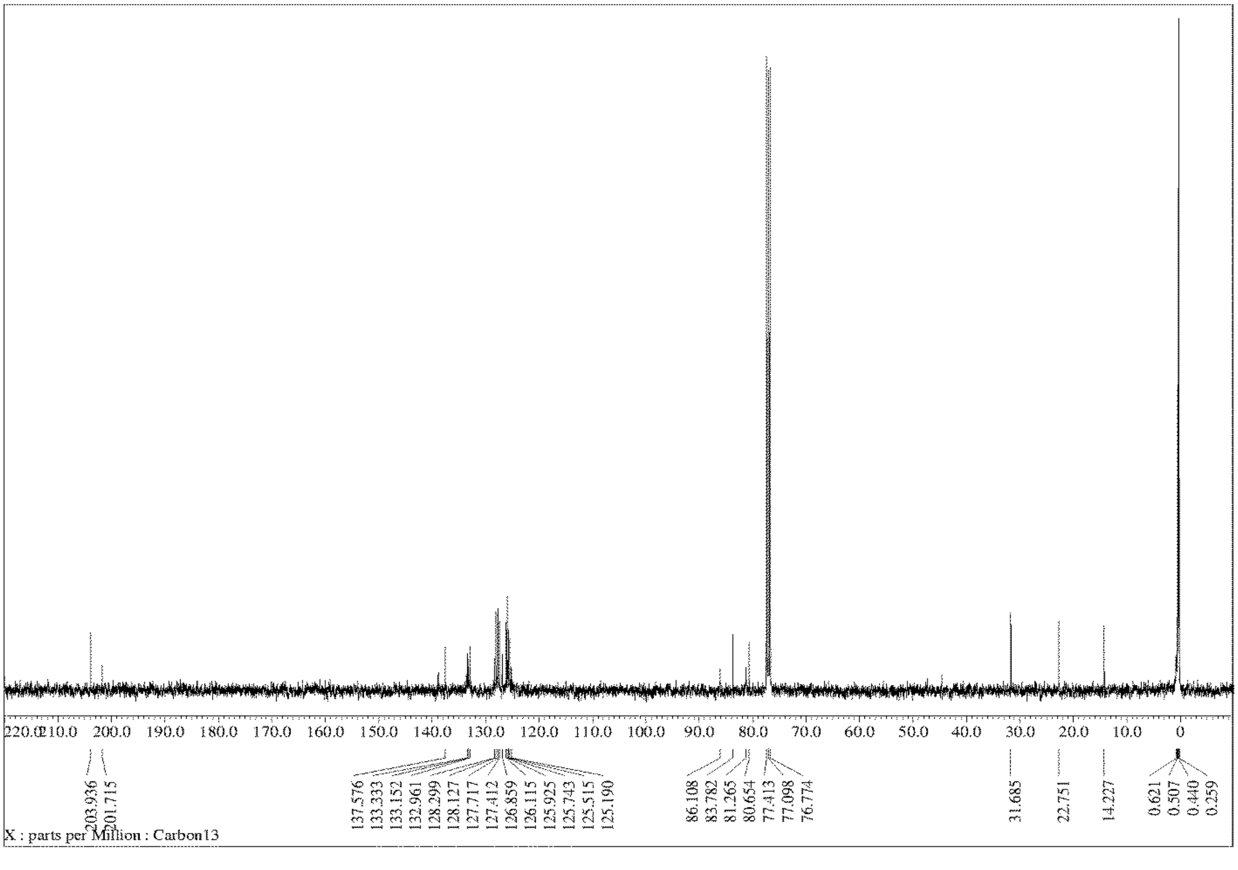
203.936
201.715
137.576
133.333
133.152
132.961
128.299
128.127
127.717
127.412
126.859
126.115
125.925
125.743
125.515
125.190
86.108
83.782
81.265
80.654
77.413
77.098
76.774
31.685
22.751
14.227
0.621
0.507
0.440
0.259
X : parts per Million : Carbon13

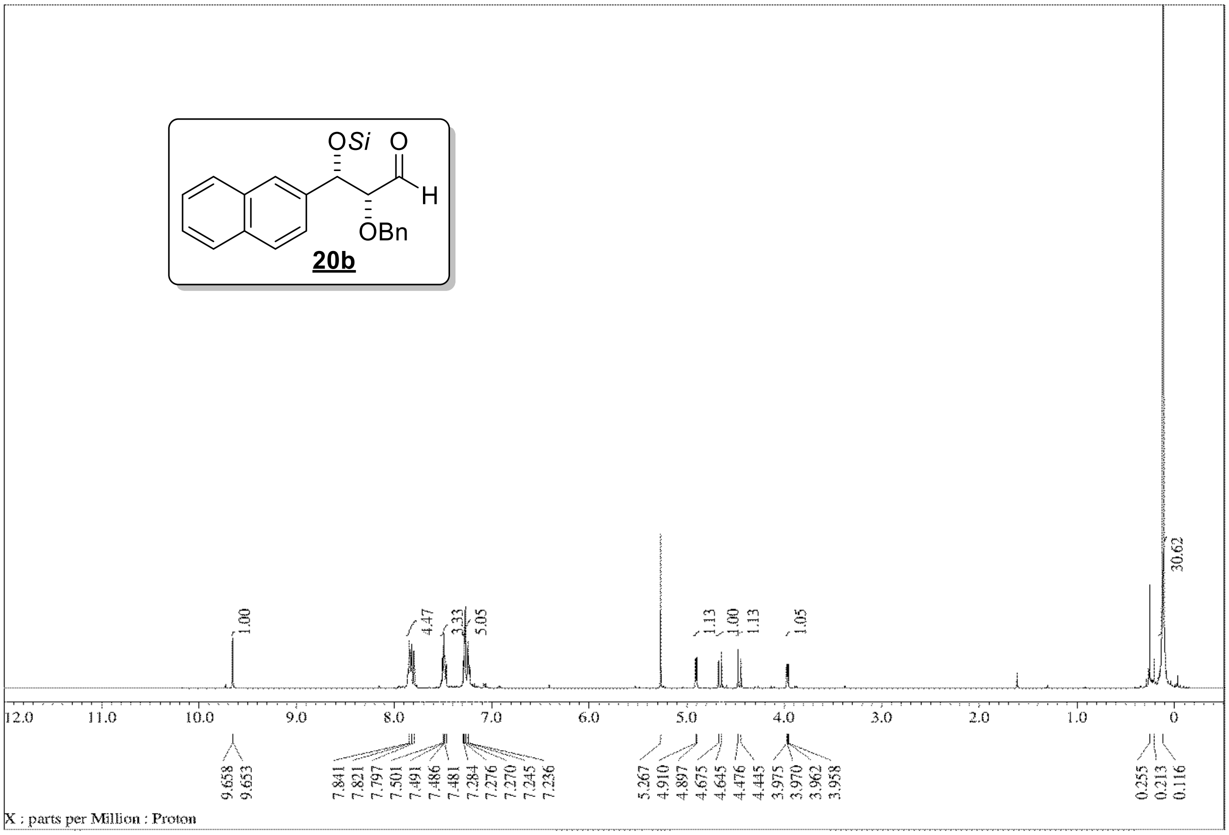
OSi
O
H
OBn
20b
1.00
4.47
3.33
5.05
1.13
1.00
1.13
1.05
30.62
12.0 11.0 10.0 9.0 8.0 7.0 6.0 5.0 4.0 3.0 2.0 1.0 0
9.658
9.653
7.841
7.821
7.797
7.501
7.491
7.486
7.481
7.284
7.276
7.270
7.245
7.236
5.267
4.910
4.897
4.675
4.645
4.476
4.445
3.975
3.970
3.962
3.958
0.255
0.213
0.116
X : parts per Million : Proton

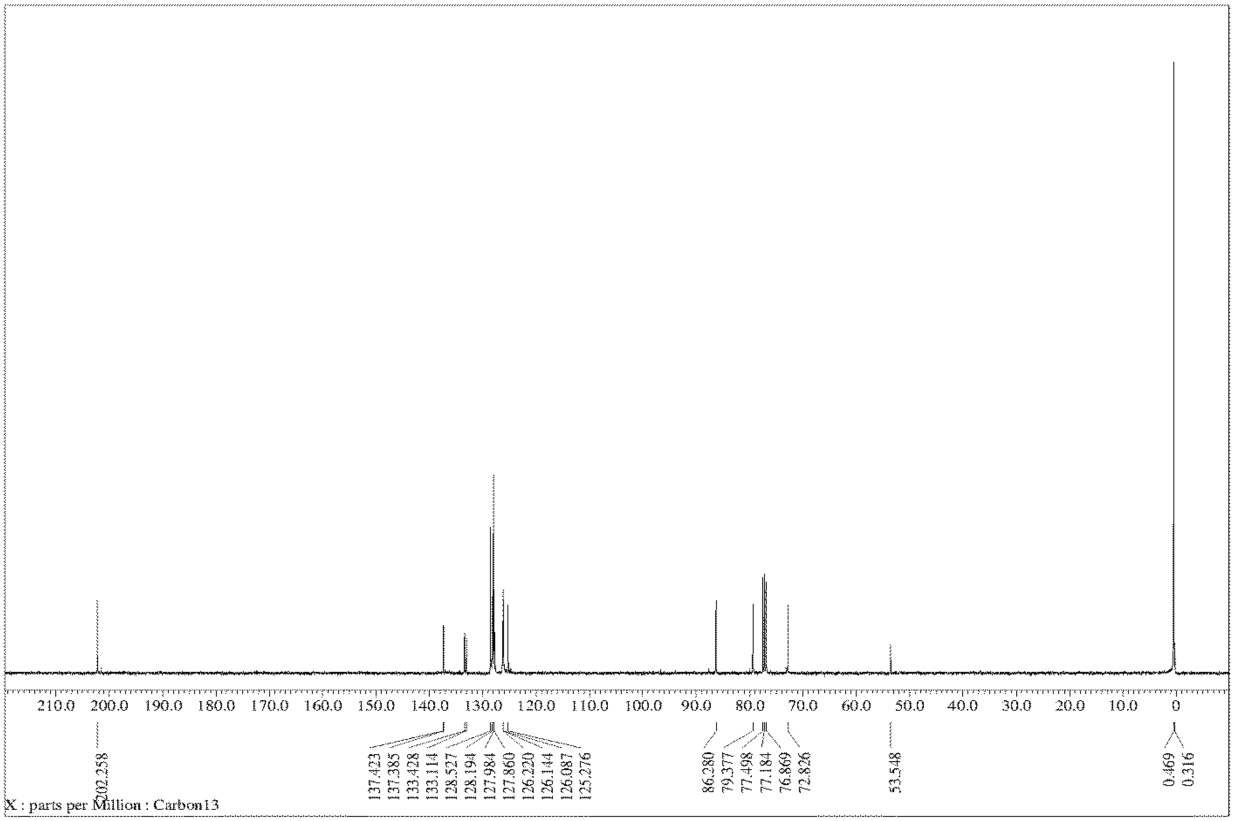
210.0 200.0 190.0 180.0 170.0 160.0 150.0 140.0 130.0 120.0 110.0 100.0 90.0 80.0 70.0 60.0 50.0 40.0 30.0 20.0 10.0 0
202.258
137.423
137.385
133.428
133.114
128.527
128.194
127.984
127.860
126.220
126.144
126.087
125.276
86.280
79.377
77.498
77.184
76.869
72.826
53.548
0.469
0.316
X : parts per Million : Carbon13

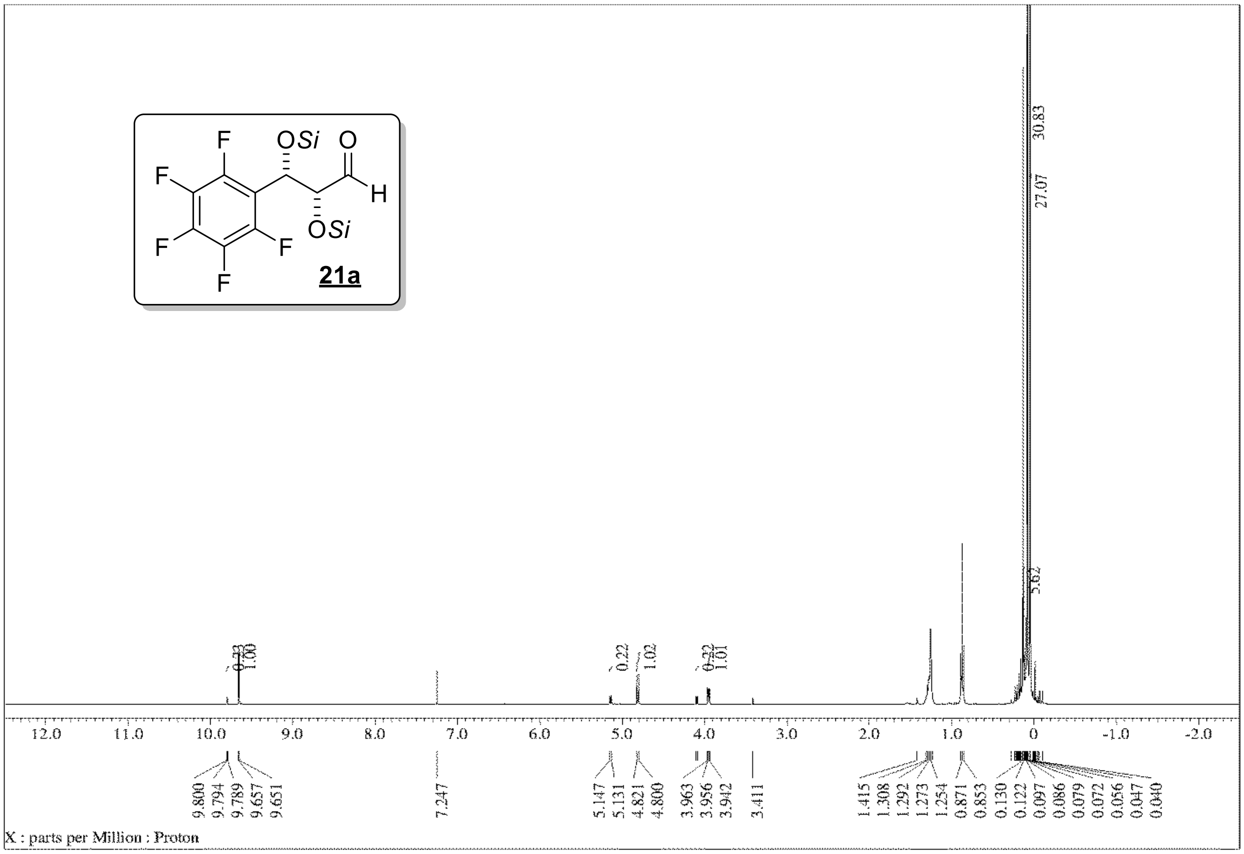
F
OSi
O
H
F
F
F
F
OSi
21a
30.83
27.07
5.62
0.22
1.02
1.01
1.00
12.0 11.0 10.0 9.0 8.0 7.0 6.0 5.0 4.0 3.0 2.0 1.0 0 -1.0 -2.0
9.800
9.794
9.789
9.657
9.651
7.247
5.147
5.131
4.821
4.800
3.963
3.956
3.942
3.411
1.415
1.308
1.292
1.273
1.254
0.871
0.853
0.130
0.122
0.097
0.086
0.079
0.072
0.056
0.047
0.040
X : parts per Million : Proton

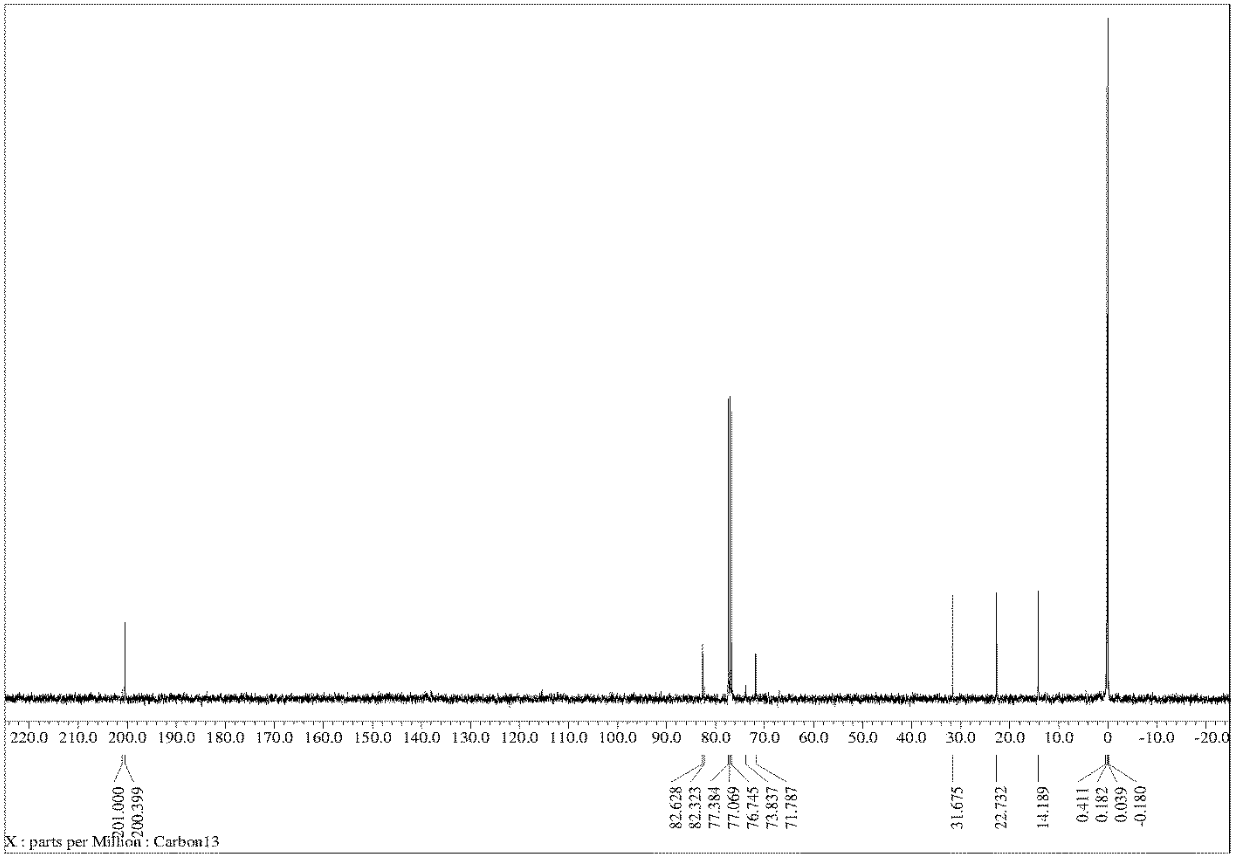
220.0 210.0 200.0 190.0 180.0 170.0 160.0 150.0 140.0 130.0 120.0 110.0 100.0 90.0 80.0 70.0 60.0 50.0 40.0 30.0 20.0 10.0 0 -10.0 -20.0
201.000
200.399
82.628
82.323
77.384
77.069
76.745
73.837
71.787
31.675
22.732
14.189
0.411
0.182
0.039
-0.180
X : parts per Million : Carbon13

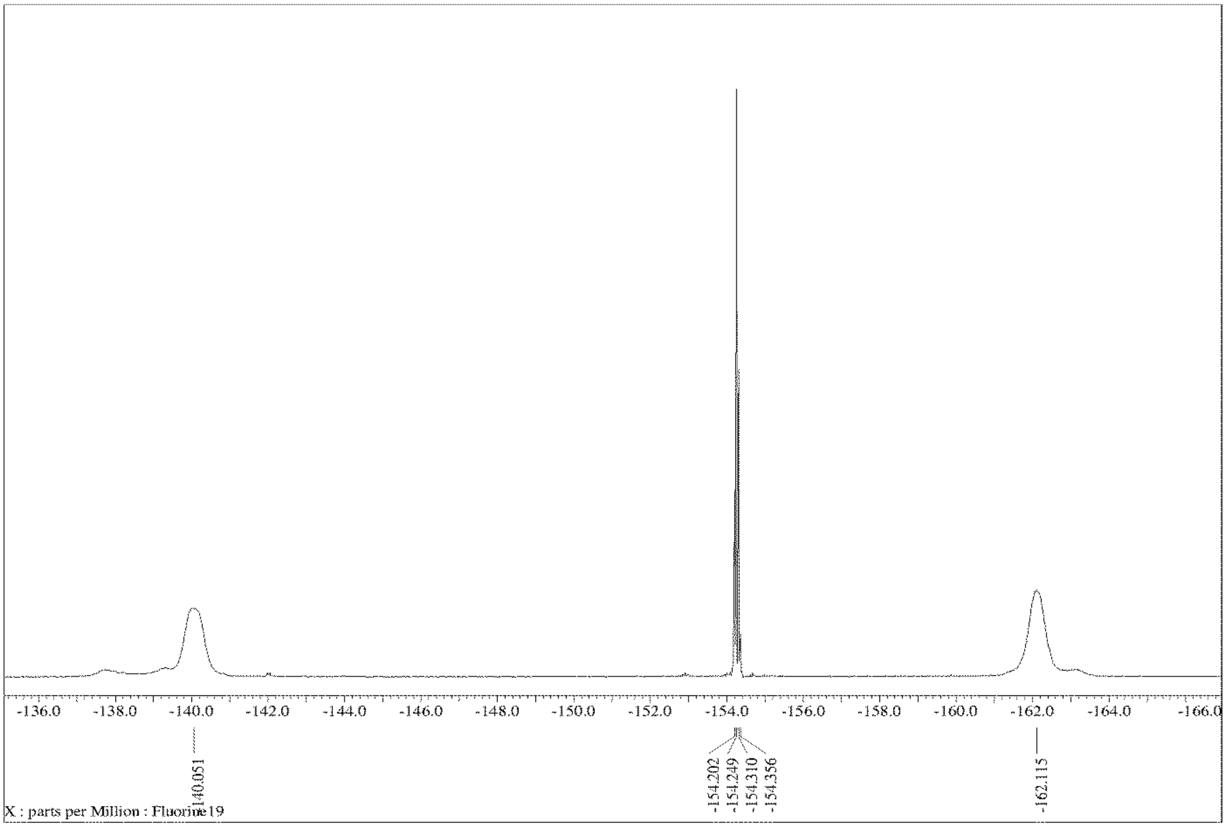
-136.0
-138.0
-140.0
-142.0
-144.0
-146.0
-148.0
-150.0
-152.0
-154.0
-156.0
-158.0
-160.0
-162.0
-164.0
-166.0
-140.051
-154.202
-154.249
-154.310
-154.356
-162.115
X : parts per Million : Fluorine19

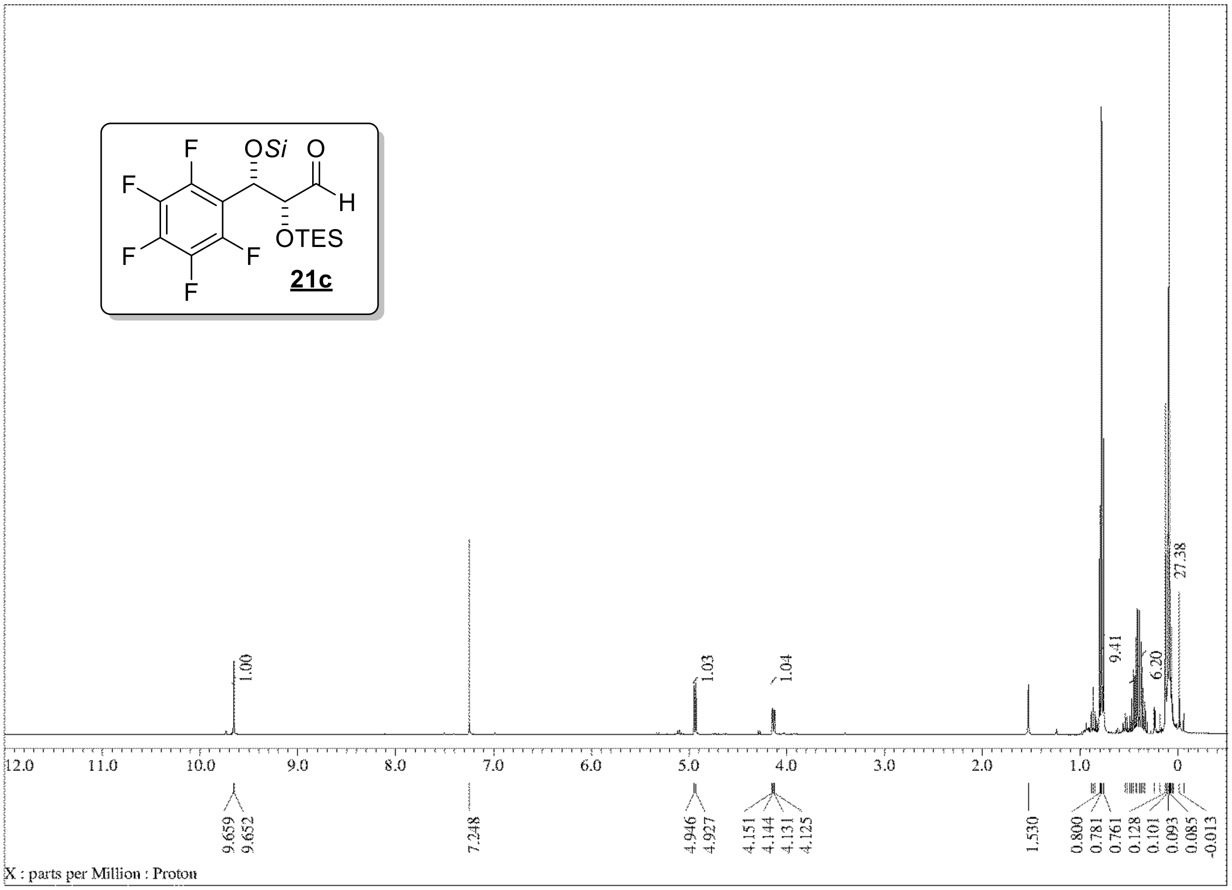
F
OSi
O
H
OTES
21c
1.00
1.03
1.04
9.41
6.20
27.38
12.0
11.0
10.0
9.0
8.0
7.0
6.0
5.0
4.0
3.0
2.0
1.0
0
9.659
9.652
7.248
4.946
4.927
4.151
4.144
4.131
4.125
1.530
0.800
0.781
0.761
0.128
0.101
0.093
0.085
-0.013
X : parts per Million : Proton

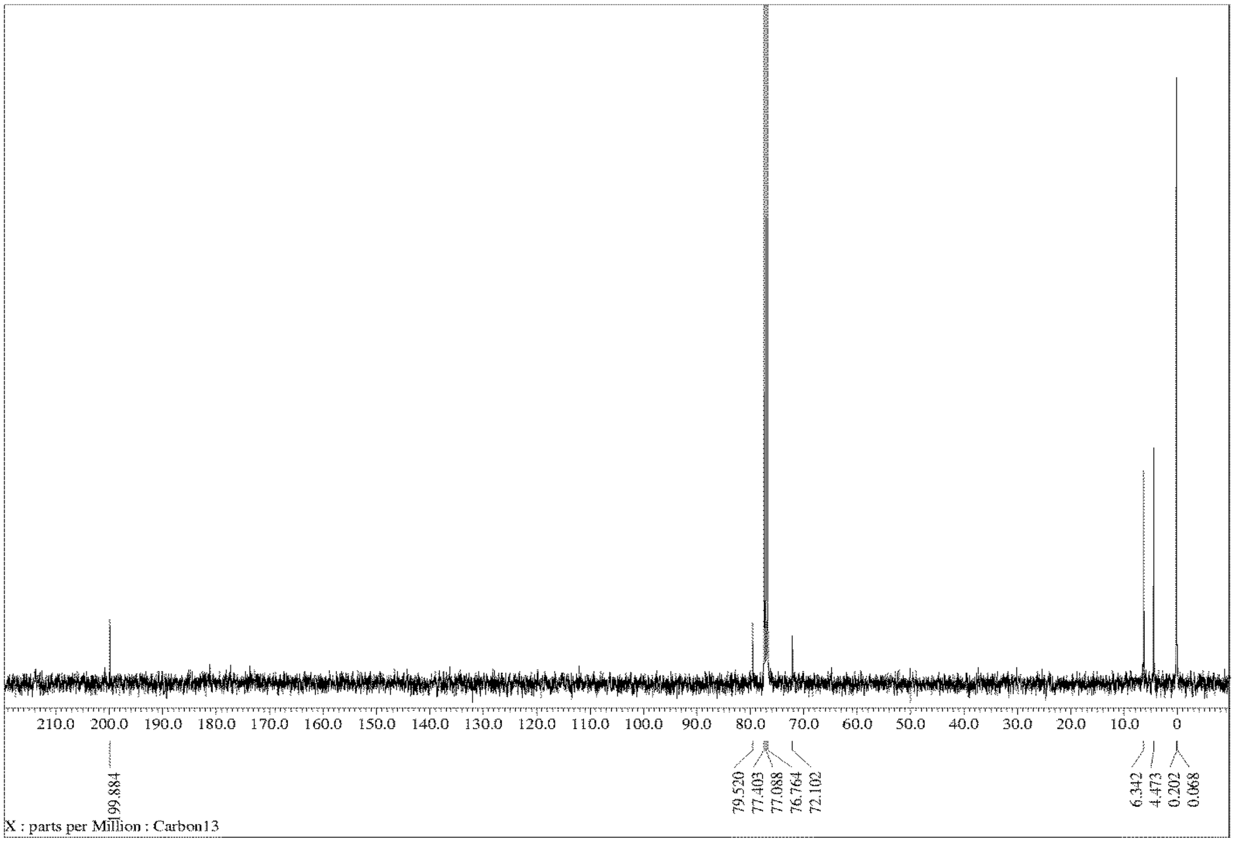
210.0 200.0 190.0 180.0 170.0 160.0 150.0 140.0 130.0 120.0 110.0 100.0 90.0 80.0 70.0 60.0 50.0 40.0 30.0 20.0 10.0 0
199.884
79.520
77.403
77.088
76.764
72.102
6.342
4.473
0.202
0.068
X : parts per Million : Carbon13

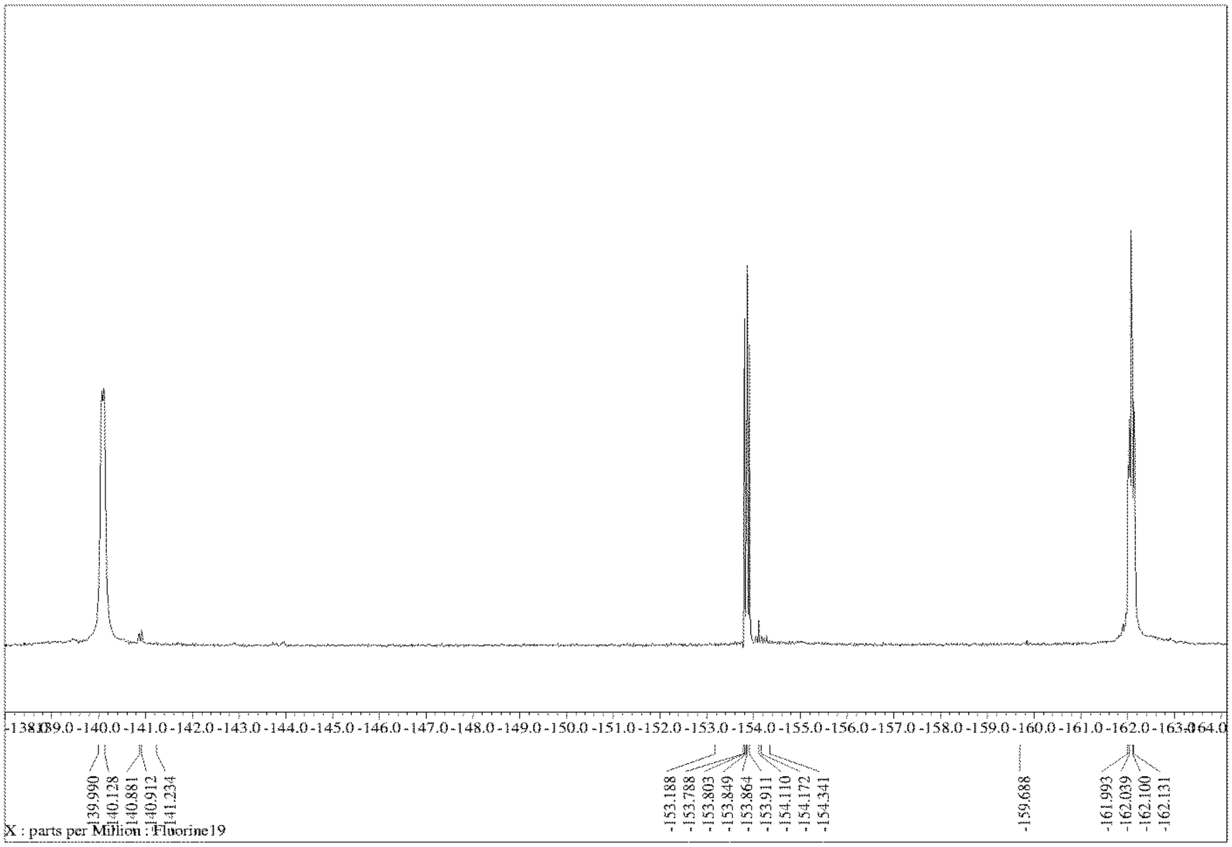
-140.0 -141.0 -142.0 -143.0 -144.0 -145.0 -146.0 -147.0 -148.0 -149.0 -150.0 -151.0 -152.0 -153.0 -154.0 -155.0 -156.0 -157.0 -158.0 -159.0 -160.0 -161.0 -162.0
-139.990
-140.128
-140.881
-140.912
-141.234
-153.188
-153.788
-153.803
-153.849
-153.864
-153.911
-154.110
-154.172
-154.341
-159.688
-161.993
-162.039
-162.100
-162.131
X : parts per Million : Fluorine19

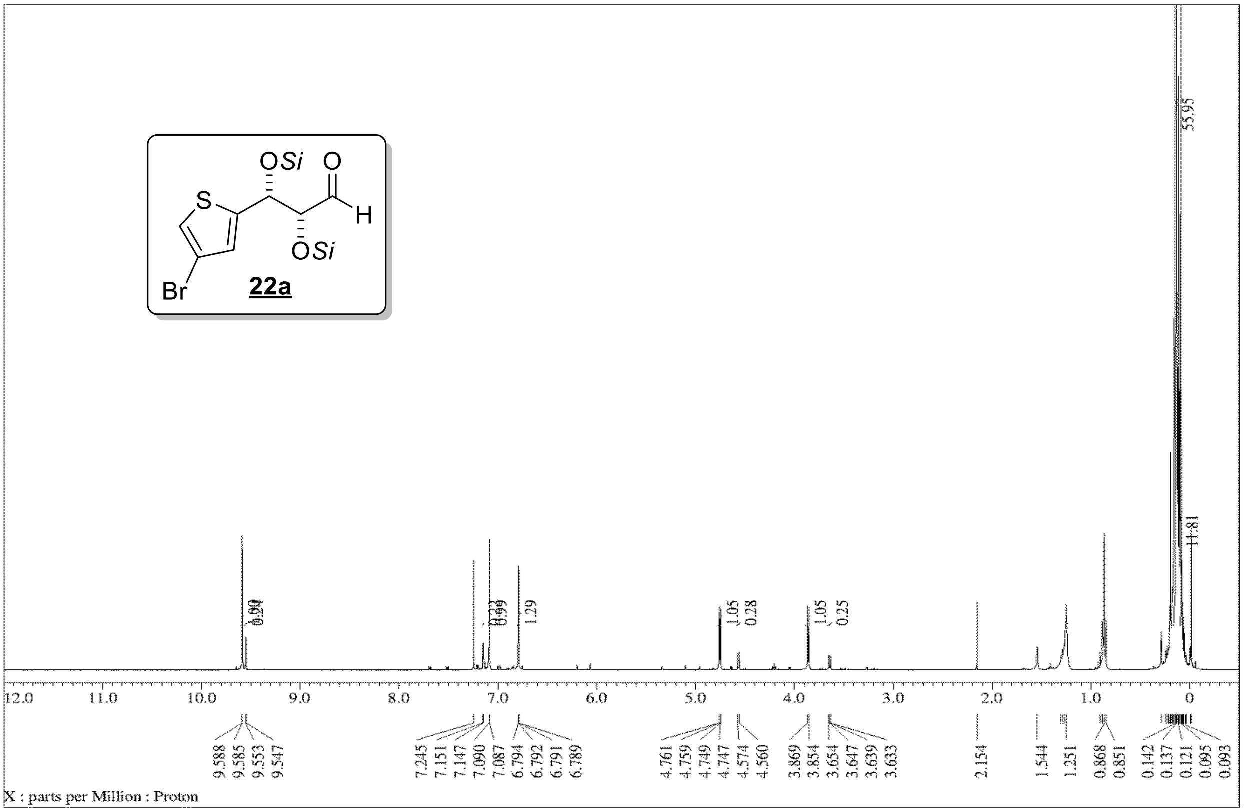

OSi
O
S
H
OSi
Br
22a
55.95
11.81
1.00
0.24
0.22
0.99
1.29
1.05
0.28
1.05
0.25
12.0 11.0 10.0 9.0 8.0 7.0 6.0 5.0 4.0 3.0 2.0 1.0 0
9.588 9.585 9.553 9.547
7.245 7.151 7.147 7.090 7.087 6.794 6.792 6.791 6.789
4.761 4.759 4.749 4.747 4.574 4.560
3.869 3.854 3.654 3.647 3.639 3.633
2.154
1.544
1.251
0.868 0.851
0.142 0.137 0.121 0.095 0.093
X : parts per Million : Proton

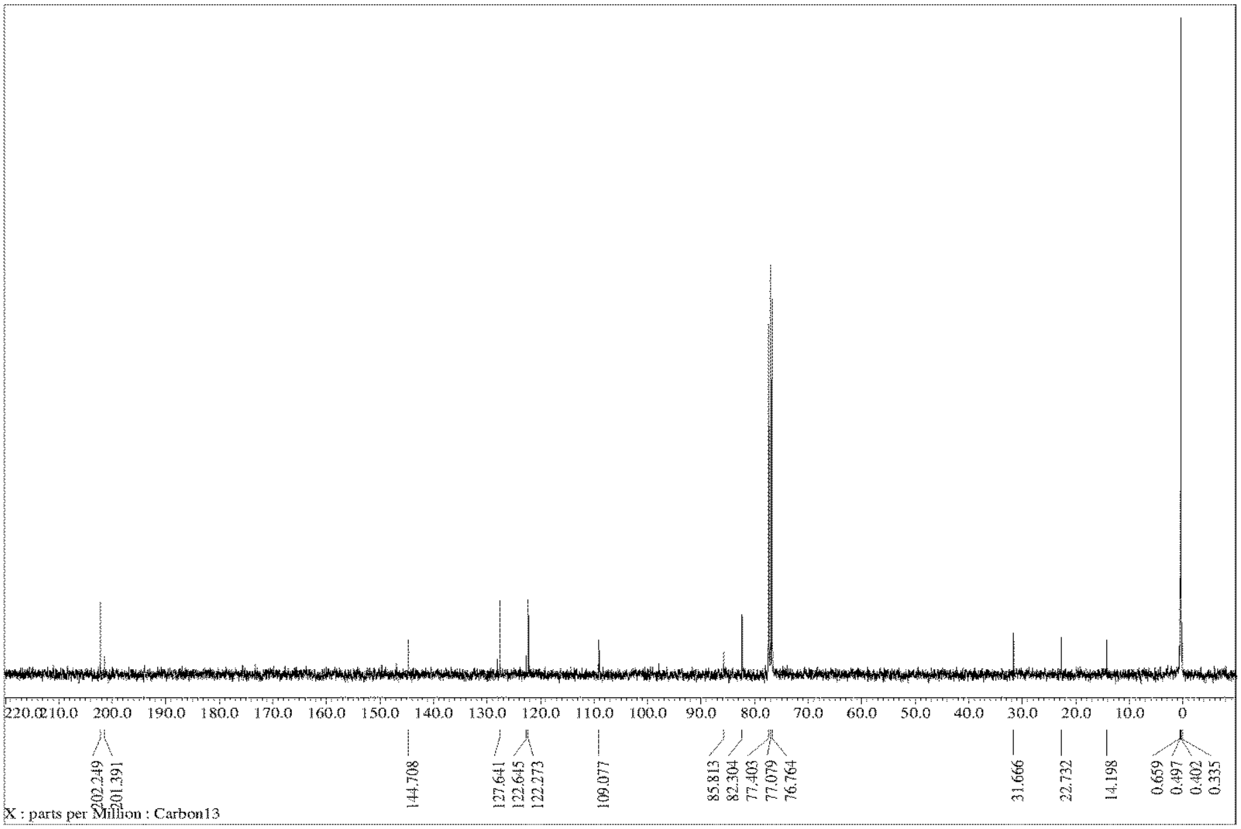

220.0 210.0 200.0 190.0 180.0 170.0 160.0 150.0 140.0 130.0 120.0 110.0 100.0 90.0 80.0 70.0 60.0 50.0 40.0 30.0 20.0 10.0 0
202.249 201.391
144.708
127.641 122.645 122.273
109.077
85.813 82.304 77.403 77.079 76.764
31.666
22.732
14.198
0.659 0.497 0.402 0.335
X : parts per Million : Carbon13

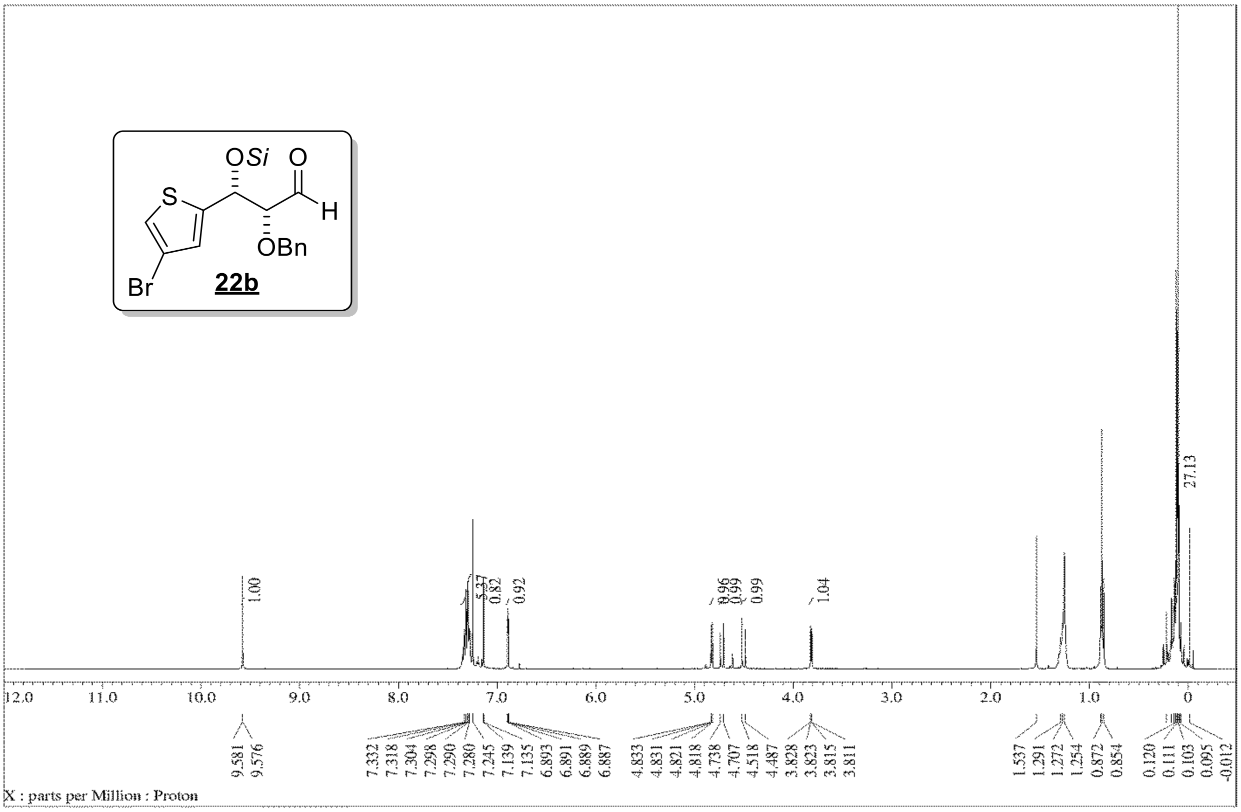
OSi O
S
H
OBn
Br
22b
1.00
5.37
0.82
0.92
0.96
0.99
0.99
1.04
27.13
12.0 11.0 10.0 9.0 8.0 7.0 6.0 5.0 4.0 3.0 2.0 1.0 0
9.581
9.576
7.332
7.318
7.304
7.298
7.290
7.280
7.245
7.139
7.135
6.893
6.891
6.889
6.887
4.833
4.831
4.821
4.818
4.738
4.707
4.518
4.487
3.828
3.823
3.815
3.811
1.537
1.291
1.272
1.254
0.872
0.854
0.120
0.111
0.103
0.095
-0.012
X : parts per Million : Proton

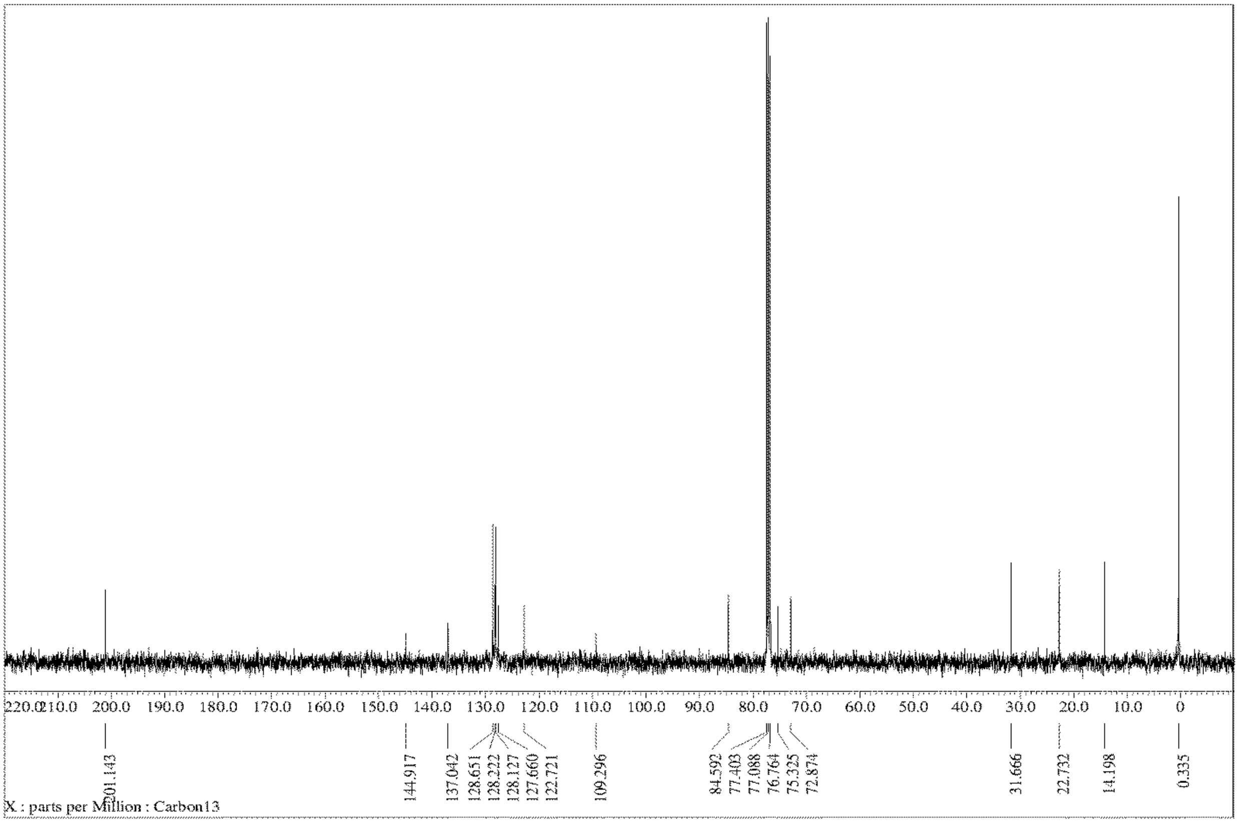
220.0 210.0 200.0 190.0 180.0 170.0 160.0 150.0 140.0 130.0 120.0 110.0 100.0 90.0 80.0 70.0 60.0 50.0 40.0 30.0 20.0 10.0 0
201.143
144.917
137.042
128.651
128.222
128.127
127.660
122.721
109.296
84.592
77.403
77.088
76.764
75.325
72.874
31.666
22.732
14.198
0.335
X : parts per Million : Carbon13

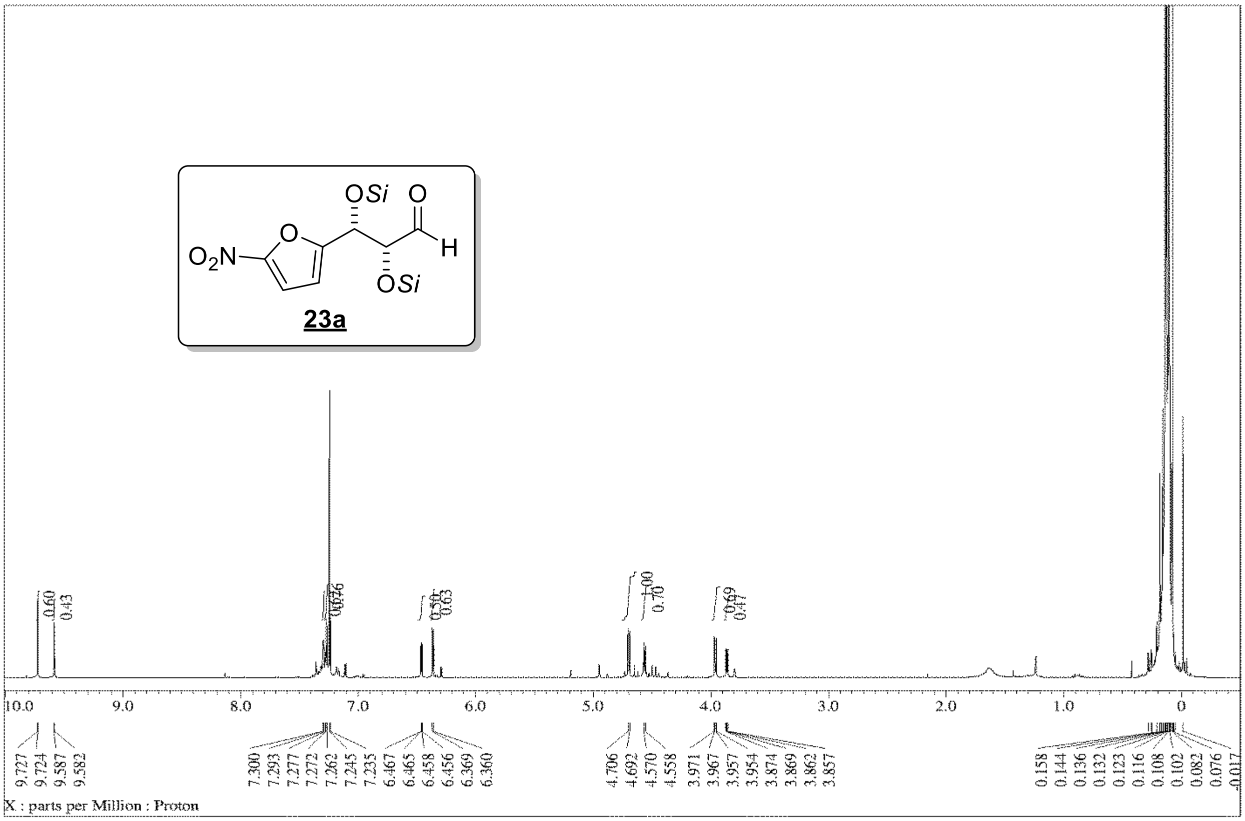
OSi
O
O2N
H
OSi
23a
10.0
9.0
8.0
7.0
6.0
5.0
4.0
3.0
2.0
1.0
0
X : parts per Million : Proton

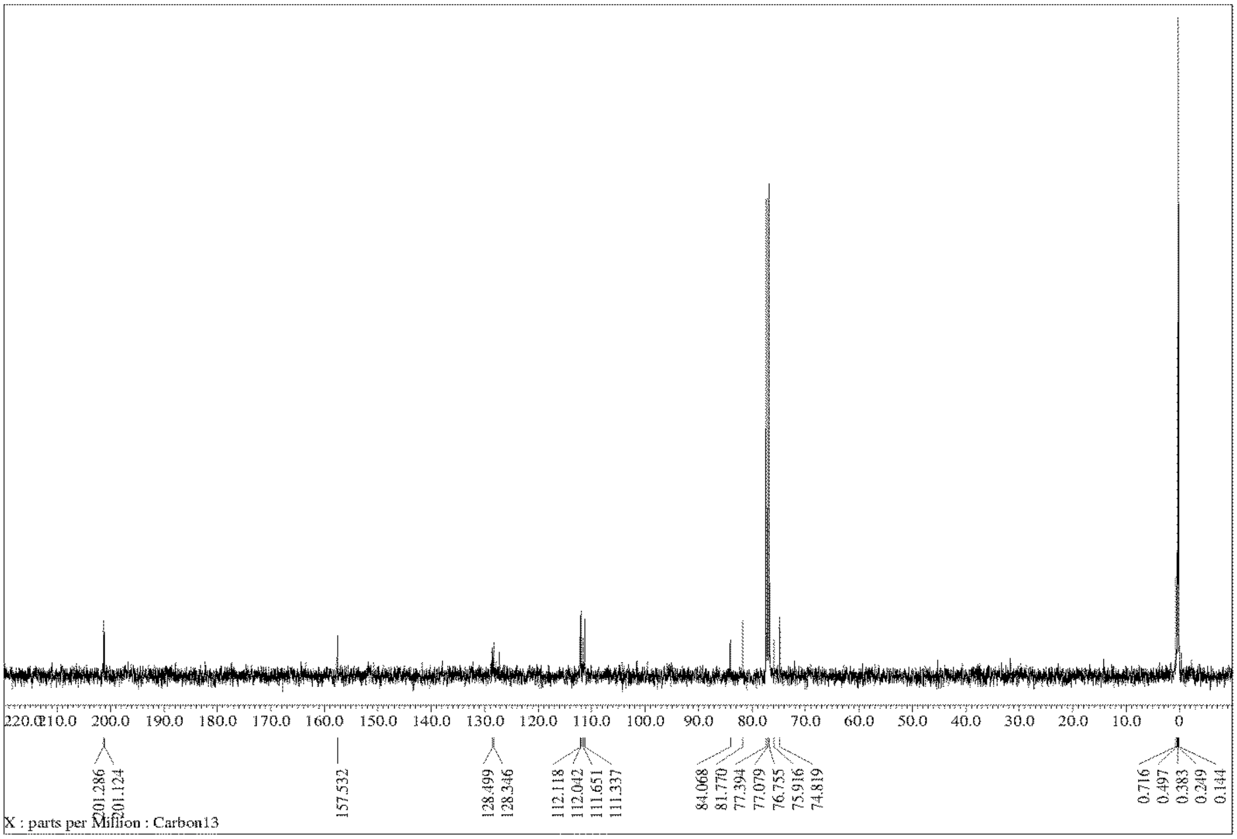
220.0 210.0 200.0 190.0 180.0 170.0 160.0 150.0 140.0 130.0 120.0 110.0 100.0 90.0 80.0 70.0 60.0 50.0 40.0 30.0 20.0 10.0 0
201.286
201.124
157.532
128.499
128.346
112.118
112.042
111.651
111.337
84.068
81.770
77.394
77.079
76.755
75.916
74.819
0.716
0.497
0.383
0.249
0.144
X : parts per Million : Carbon13

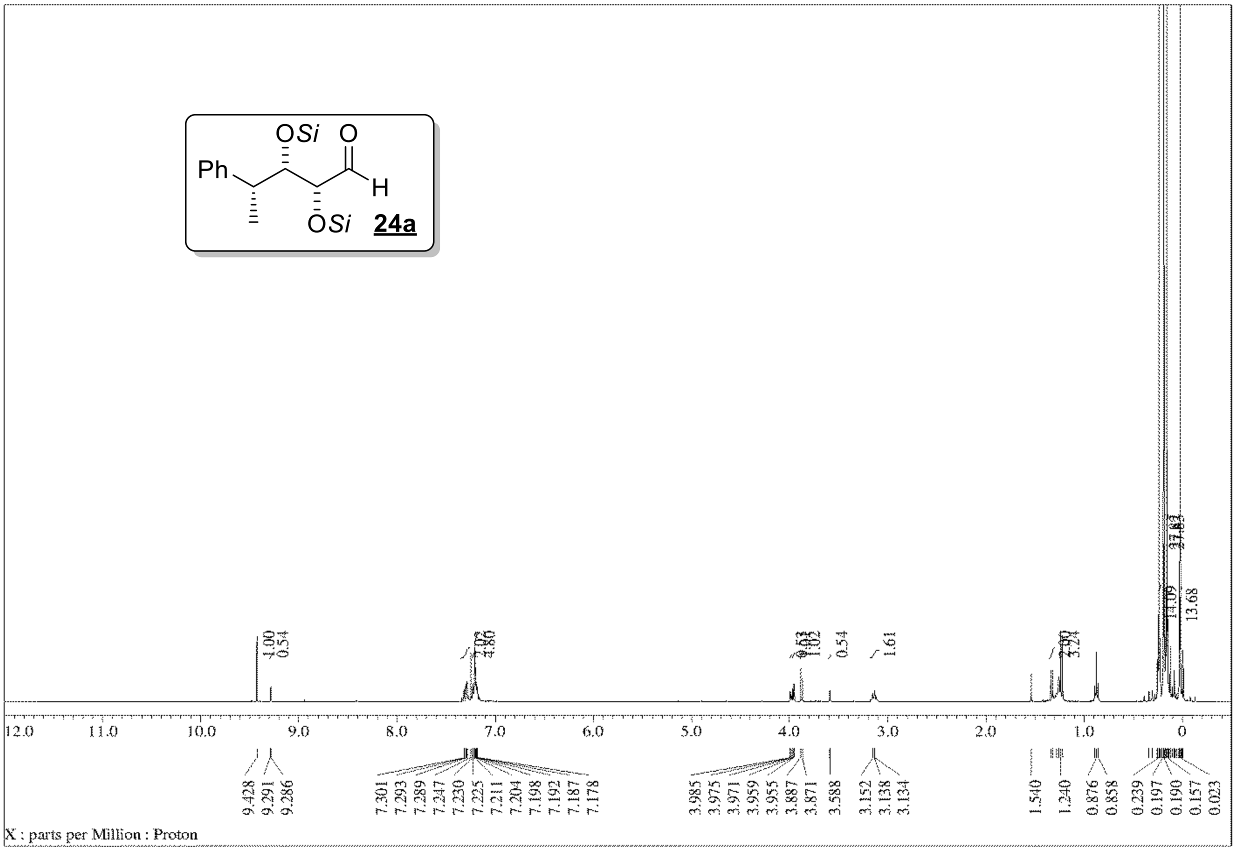
OSi
O
Ph
H
OSi
24a
1.00
0.54
4.80
1.02
0.54
1.61
3.24
14.09
13.68
12.0 11.0 10.0 9.0 8.0 7.0 6.0 5.0 4.0 3.0 2.0 1.0 0
9.428
9.291
9.286
7.301
7.293
7.289
7.247
7.230
7.225
7.211
7.204
7.198
7.192
7.187
7.178
3.985
3.975
3.971
3.959
3.955
3.887
3.871
3.588
3.152
3.138
3.134
1.540
1.240
0.876
0.858
0.239
0.197
0.190
0.157
0.023
X : parts per Million : Proton

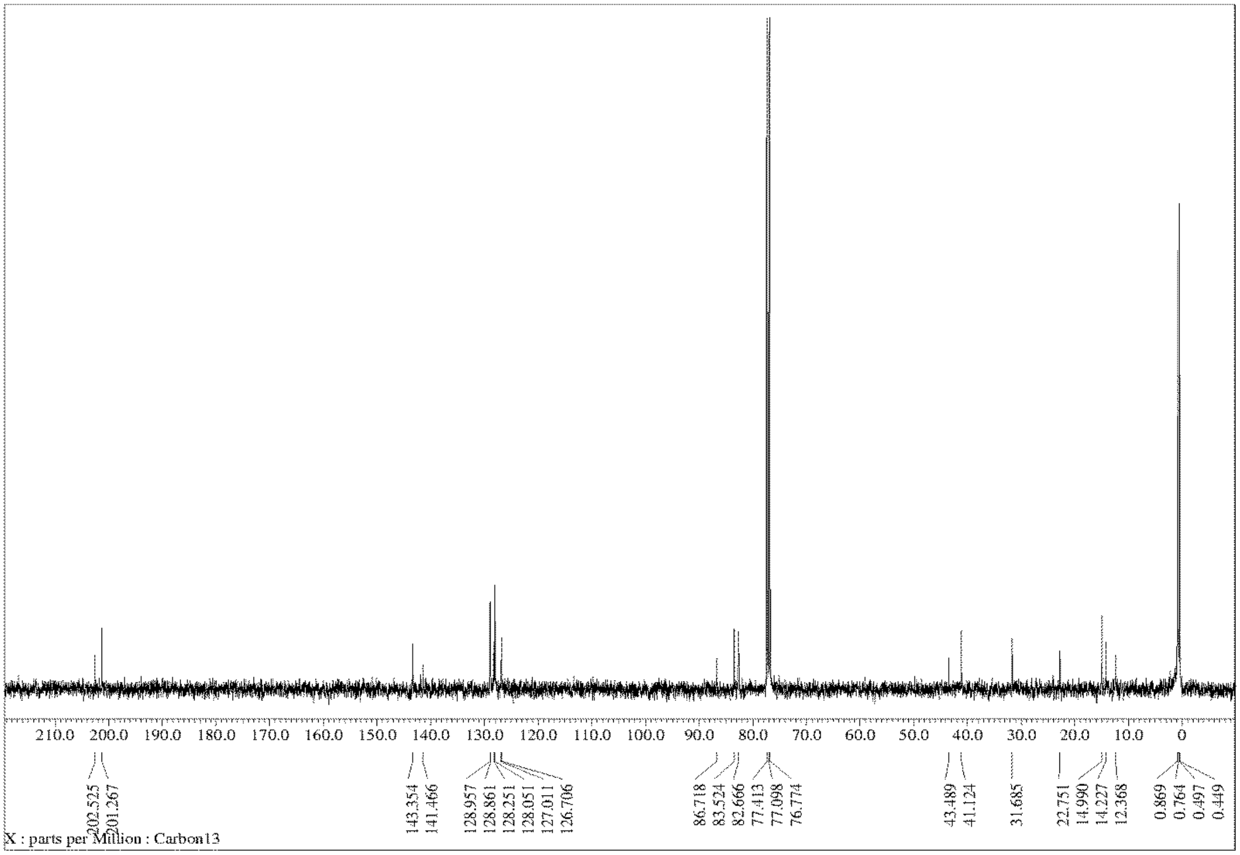
210.0 200.0 190.0 180.0 170.0 160.0 150.0 140.0 130.0 120.0 110.0 100.0 90.0 80.0 70.0 60.0 50.0 40.0 30.0 20.0 10.0 0
202.525
201.267
143.354
141.466
128.957
128.861
128.251
128.051
127.011
126.706
86.718
83.524
82.666
77.413
77.098
76.774
43.489
41.124
31.685
22.751
14.990
14.227
12.368
0.869
0.764
0.497
0.449
X : parts per Million : Carbon13

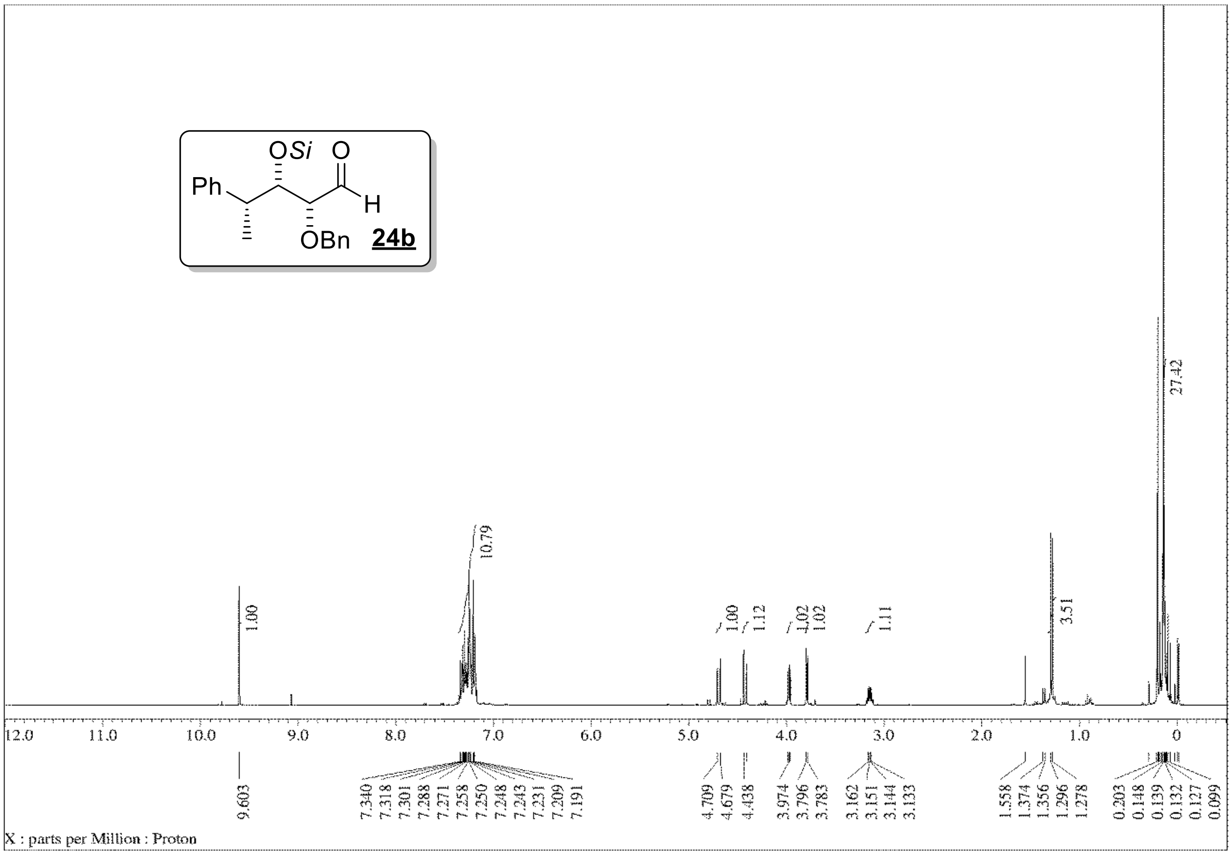
OSi
O
Ph
H
OBn
24b
1.00
10.79
1.00
1.12
1.02
1.02
1.11
3.51
27.42
12.0 11.0 10.0 9.0 8.0 7.0 6.0 5.0 4.0 3.0 2.0 1.0 0
9.603
7.340 7.318 7.301 7.288 7.271 7.258 7.250 7.248 7.243 7.231 7.209 7.191
4.709 4.679 4.438
3.974 3.796 3.783
3.162 3.151 3.144 3.133
1.558 1.374 1.356 1.296 1.278
0.203 0.148 0.139 0.132 0.127 0.099
X : parts per Million : Proton

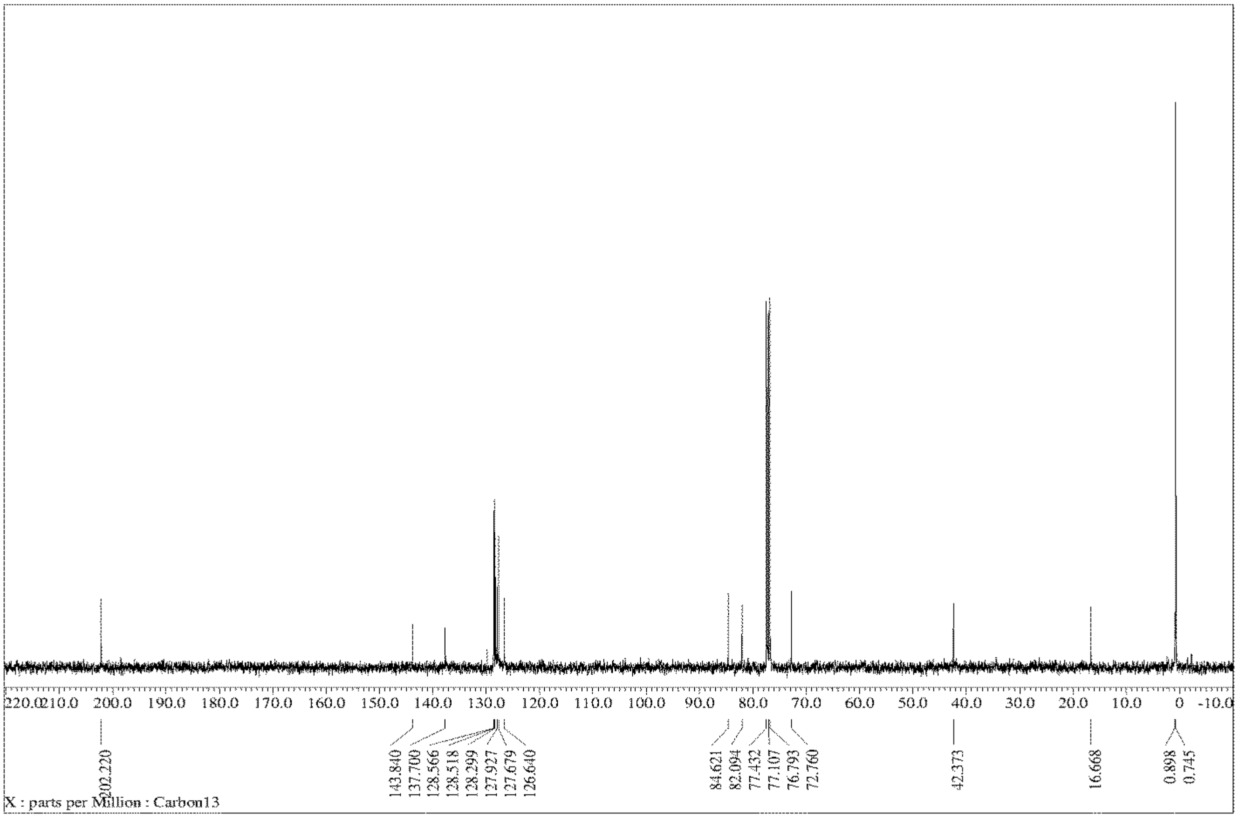
220.0 210.0 200.0 190.0 180.0 170.0 160.0 150.0 140.0 130.0 120.0 110.0 100.0 90.0 80.0 70.0 60.0 50.0 40.0 30.0 20.0 10.0 0 -10.0
202.220
143.840 137.709 128.566 128.518 128.299 127.927 127.679 126.640
84.621 82.094 77.432 77.107 76.793 72.760
42.373
16.668
0.898 0.745
X : parts per Million : Carbon13

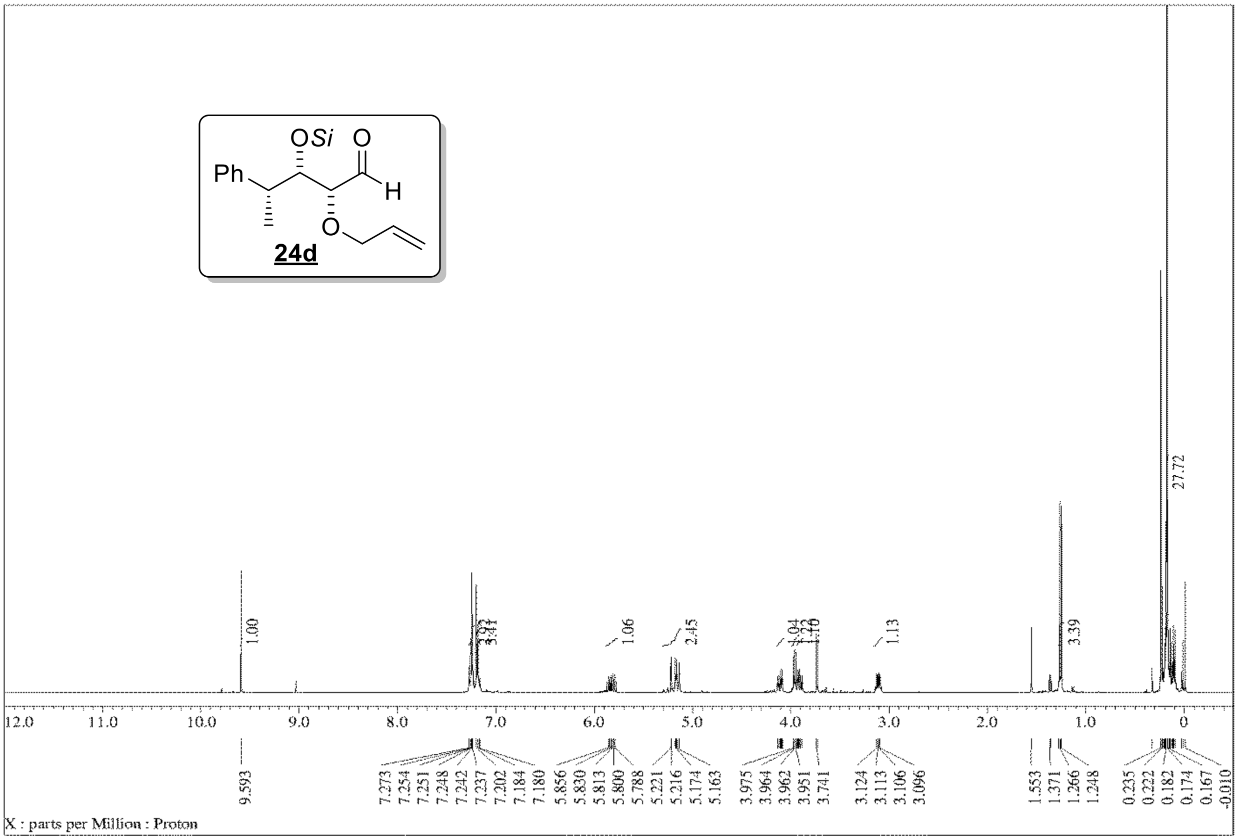
OSi
O
Ph
H
O
24d
1.00
3.92
3.41
1.06
2.45
1.04
1.23
1.10
1.13
3.39
27.72
12.0 11.0 10.0 9.0 8.0 7.0 6.0 5.0 4.0 3.0 2.0 1.0 0
9.593
7.273 7.254 7.251 7.248 7.242 7.237 7.202 7.184 7.180
5.856 5.830 5.813 5.800 5.788
5.221 5.216 5.174 5.163
3.975 3.964 3.962 3.951 3.741
3.124 3.113 3.106 3.096
1.553 1.371 1.266 1.248
0.235 0.222 0.182 0.174 0.167 -0.010
X : parts per Million : Proton

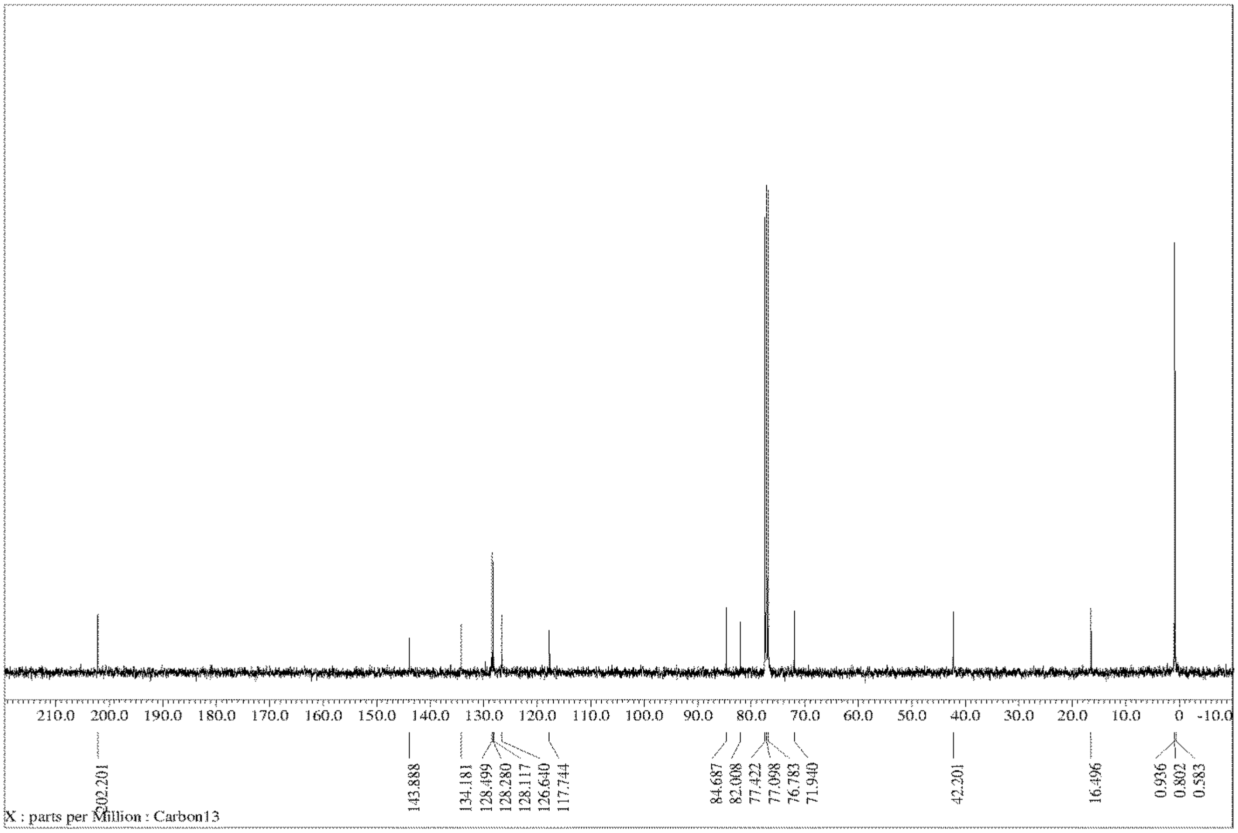
210.0 200.0 190.0 180.0 170.0 160.0 150.0 140.0 130.0 120.0 110.0 100.0 90.0 80.0 70.0 60.0 50.0 40.0 30.0 20.0 10.0 0 -10.0
202.201
143.888
134.181 128.499 128.280 128.117 126.640 117.744
84.687 82.008 77.422 77.098 76.783 71.940
42.201
16.496
0.936 0.802 0.583
X : parts per Million : Carbon13

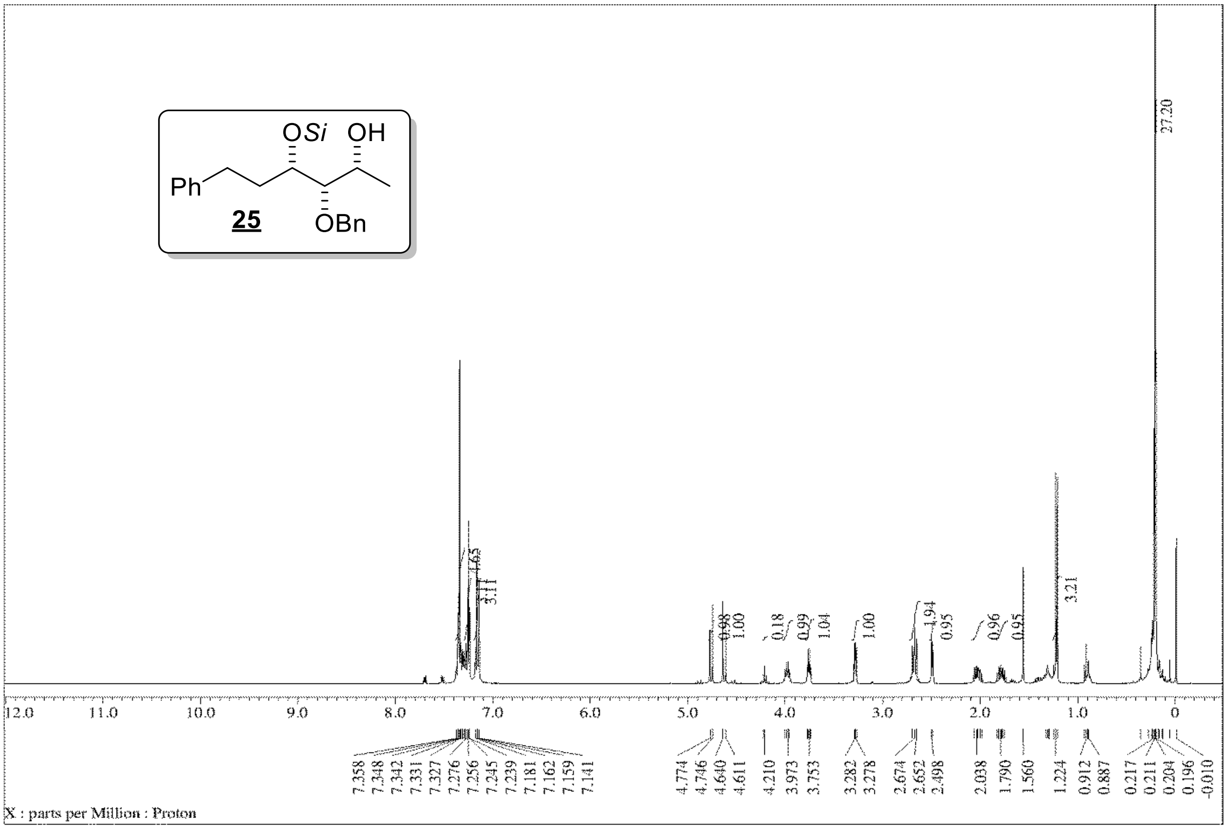
OSi OH
Ph
25
OBn
27.20
1.65
3.11
0.98
1.00
0.18
0.99
1.04
1.00
1.94
0.95
0.96
0.95
3.21
12.0 11.0 10.0 9.0 8.0 7.0 6.0 5.0 4.0 3.0 2.0 1.0 0
7.358 7.348 7.342 7.331 7.327 7.276 7.256 7.245 7.239 7.181 7.162 7.159 7.141
4.774 4.746 4.640 4.611
4.210 3.973 3.753
3.282 3.278
2.674 2.652 2.498
2.038 1.790 1.560
1.224 0.912 0.887
0.217 0.211 0.204 0.196 -0.010
X : parts per Million : Proton

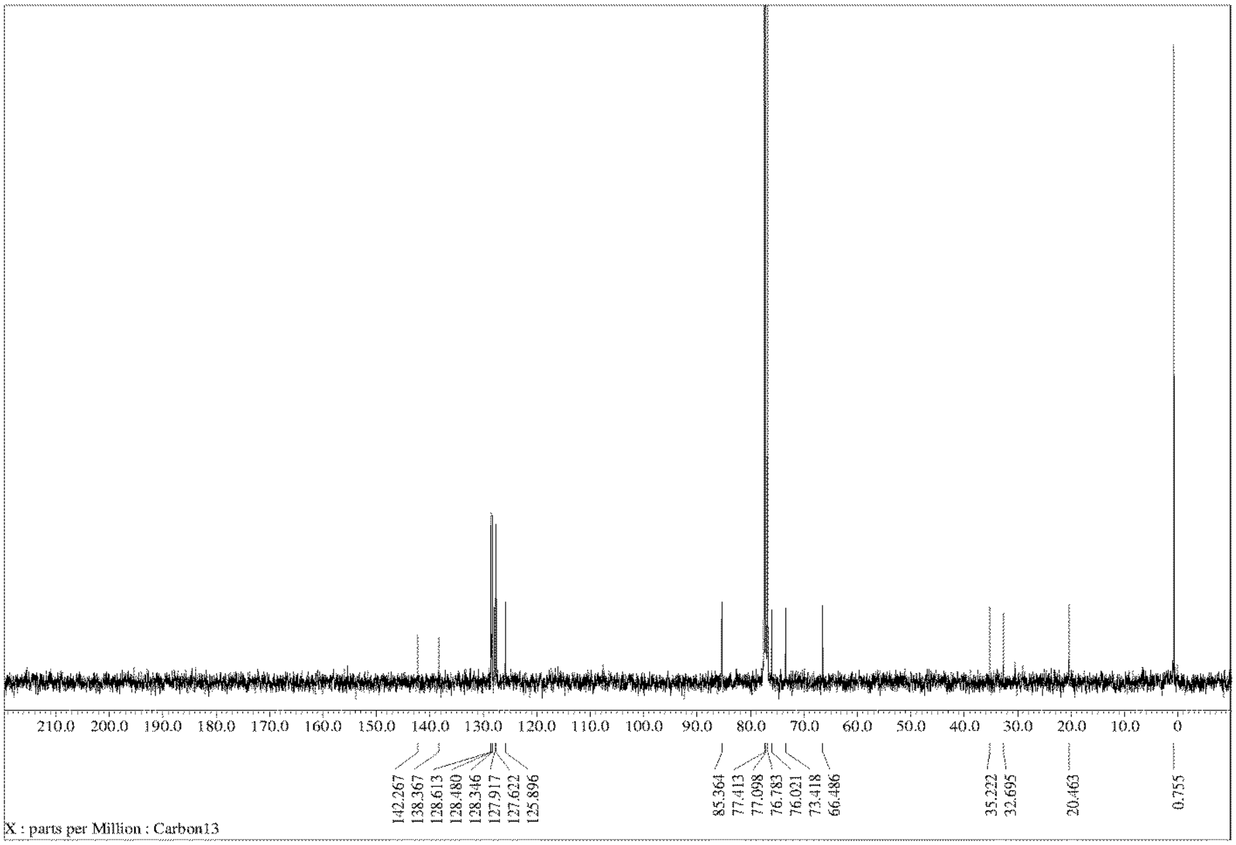
210.0 200.0 190.0 180.0 170.0 160.0 150.0 140.0 130.0 120.0 110.0 100.0 90.0 80.0 70.0 60.0 50.0 40.0 30.0 20.0 10.0 0
142.267 138.367 128.613 128.480 128.346 127.917 127.622 125.896
85.364 77.413 77.098 76.783 76.021 73.418 66.486
35.222 32.695
20.463
0.755
X : parts per Million : Carbon13

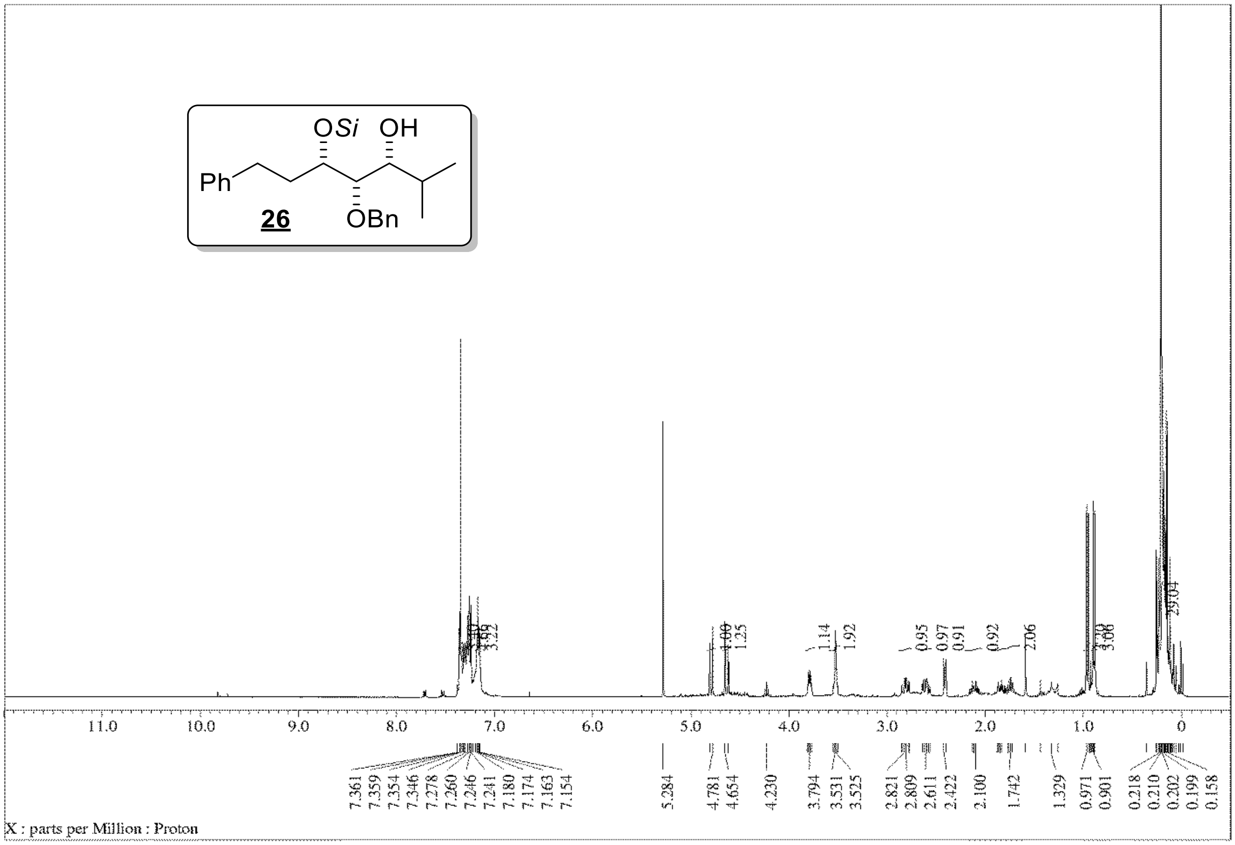
OSi OH
Ph
26
OBn
1.14
1.92
0.95
0.97
0.91
0.92
2.06
29.04
11.0 10.0 9.0 8.0 7.0 6.0 5.0 4.0 3.0 2.0 1.0 0
7.361 7.359 7.354 7.346 7.278 7.260 7.246 7.241 7.180 7.174 7.163 7.154
5.284
4.781 4.654
4.230
3.794 3.531 3.525
2.821 2.809 2.611 2.422
2.100
1.742
1.329
0.971 0.901
0.218 0.210 0.202 0.199 0.158
X : parts per Million : Proton

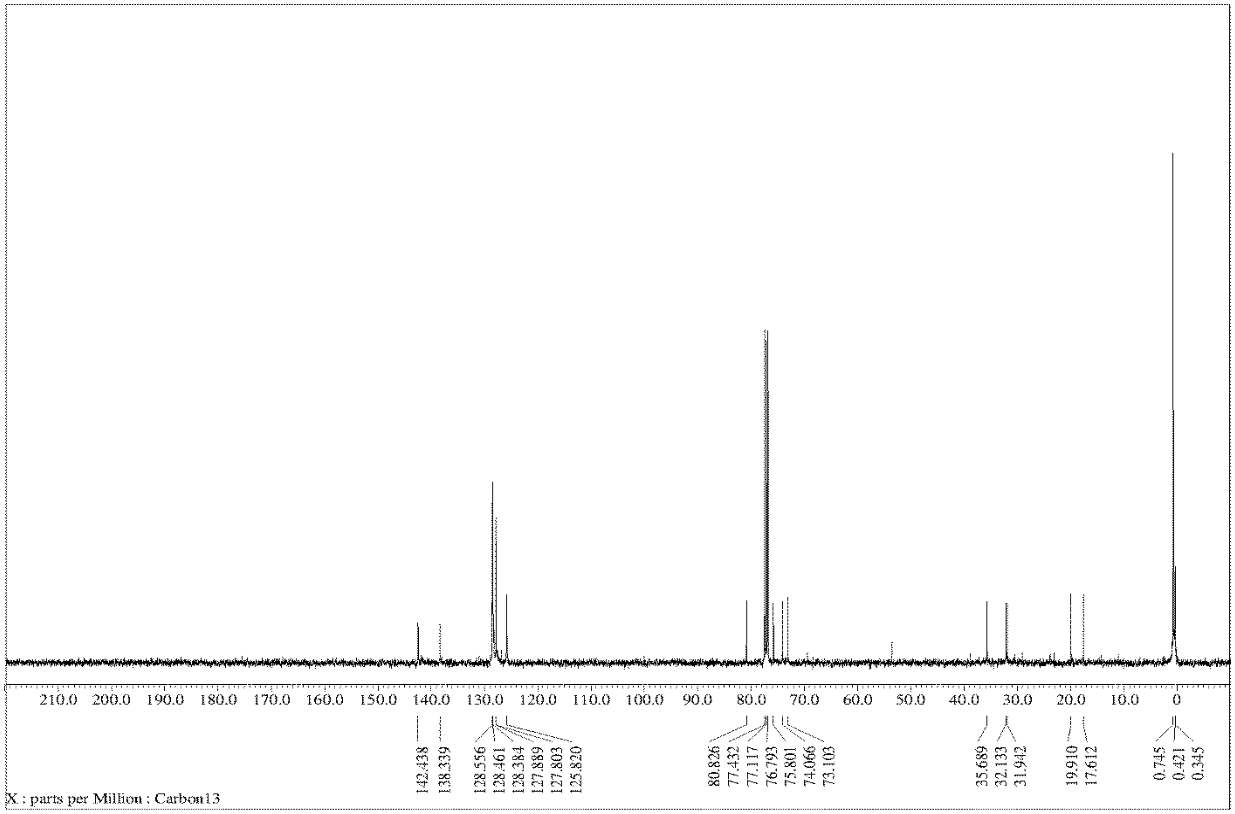
210.0 200.0 190.0 180.0 170.0 160.0 150.0 140.0 130.0 120.0 110.0 100.0 90.0 80.0 70.0 60.0 50.0 40.0 30.0 20.0 10.0 0
142.438 138.339
128.556 128.461 128.384 127.889 127.803 125.820
80.826 77.432 77.117 76.793 75.801 74.066 73.103
35.689 32.133 31.942
19.910 17.612
0.745 0.421 0.345
X : parts per Million : Carbon13

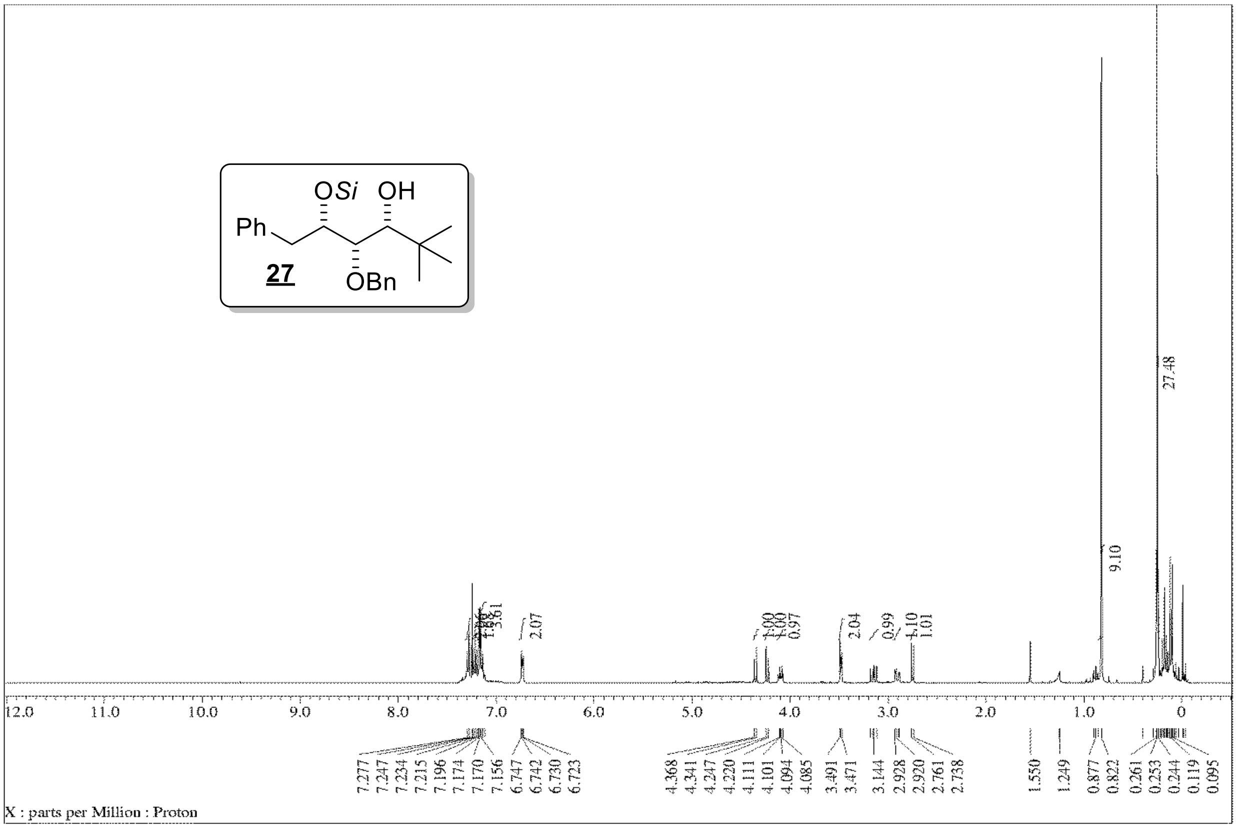
OSi OH
Ph
27
OBn
27.48
9.10
2.07
1.00
1.00
0.97
2.04
0.99
1.10
1.01
12.0 11.0 10.0 9.0 8.0 7.0 6.0 5.0 4.0 3.0 2.0 1.0 0
7.277 7.247 7.234 7.215 7.196 7.174 7.170 7.156 6.747 6.742 6.730 6.723
4.368 4.341 4.247 4.220 4.111 4.101 4.094 4.085 3.491 3.471 3.144 2.928 2.920 2.761 2.738
1.550 1.249 0.877 0.822 0.261 0.253 0.244 0.119 0.095
X : parts per Million : Proton

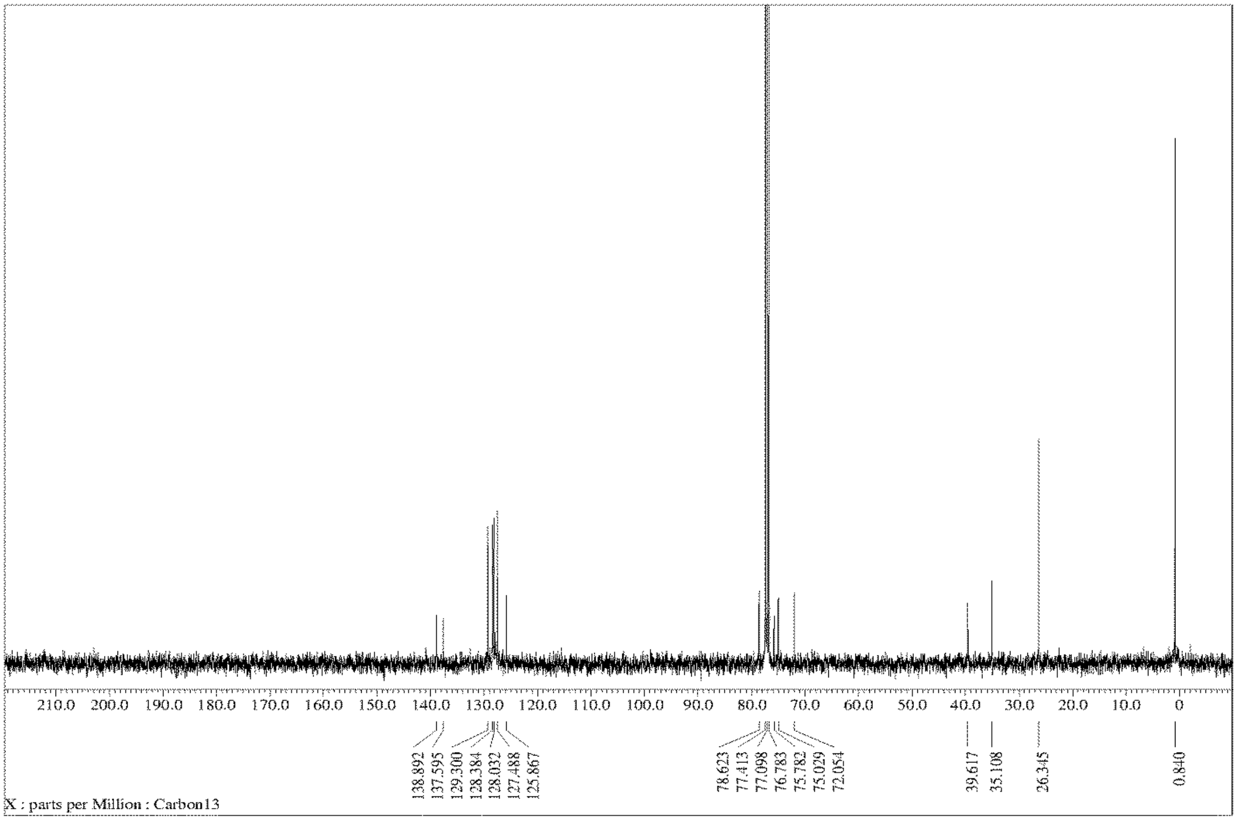
210.0 200.0 190.0 180.0 170.0 160.0 150.0 140.0 130.0 120.0 110.0 100.0 90.0 80.0 70.0 60.0 50.0 40.0 30.0 20.0 10.0 0
138.892 137.595 129.300 128.384 128.032 127.488 125.867
78.623 77.413 77.098 76.783 75.782 75.029 72.054
39.617 35.108 26.345 0.840
X : parts per Million : Carbon13

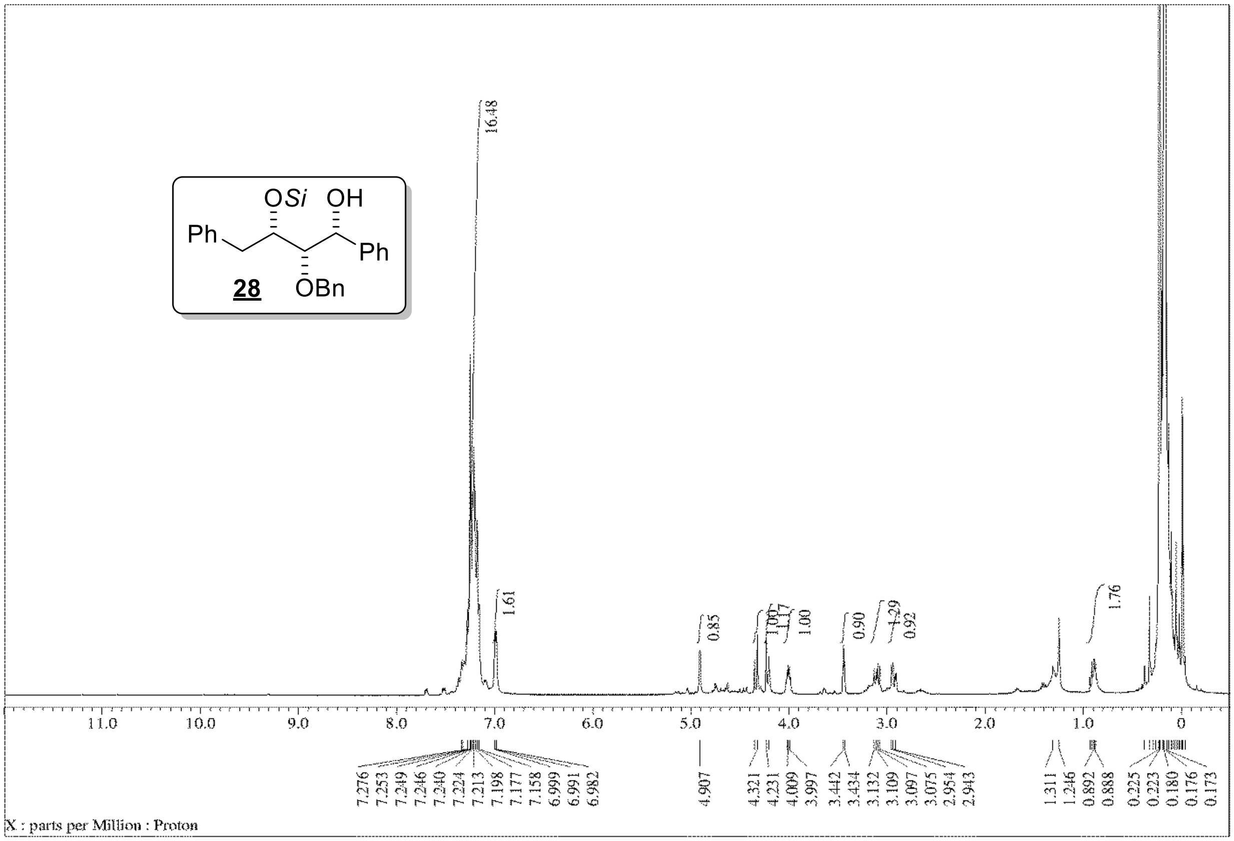
OSi OH
Ph
Ph
OBn
28
16.48
1.61
0.85
1.00
1.17
1.00
0.90
1.29
0.92
1.76
11.0 10.0 9.0 8.0 7.0 6.0 5.0 4.0 3.0 2.0 1.0 0
7.276 7.253 7.249 7.246 7.240 7.224 7.213 7.198 7.177 7.158 6.999 6.991 6.982
4.907
4.321 4.231 4.009 3.997 3.442 3.434 3.132 3.109 3.097 3.075 2.954 2.943
1.311 1.246 0.892 0.888 0.225 0.223 0.180 0.176 0.173
X : parts per Million : Proton

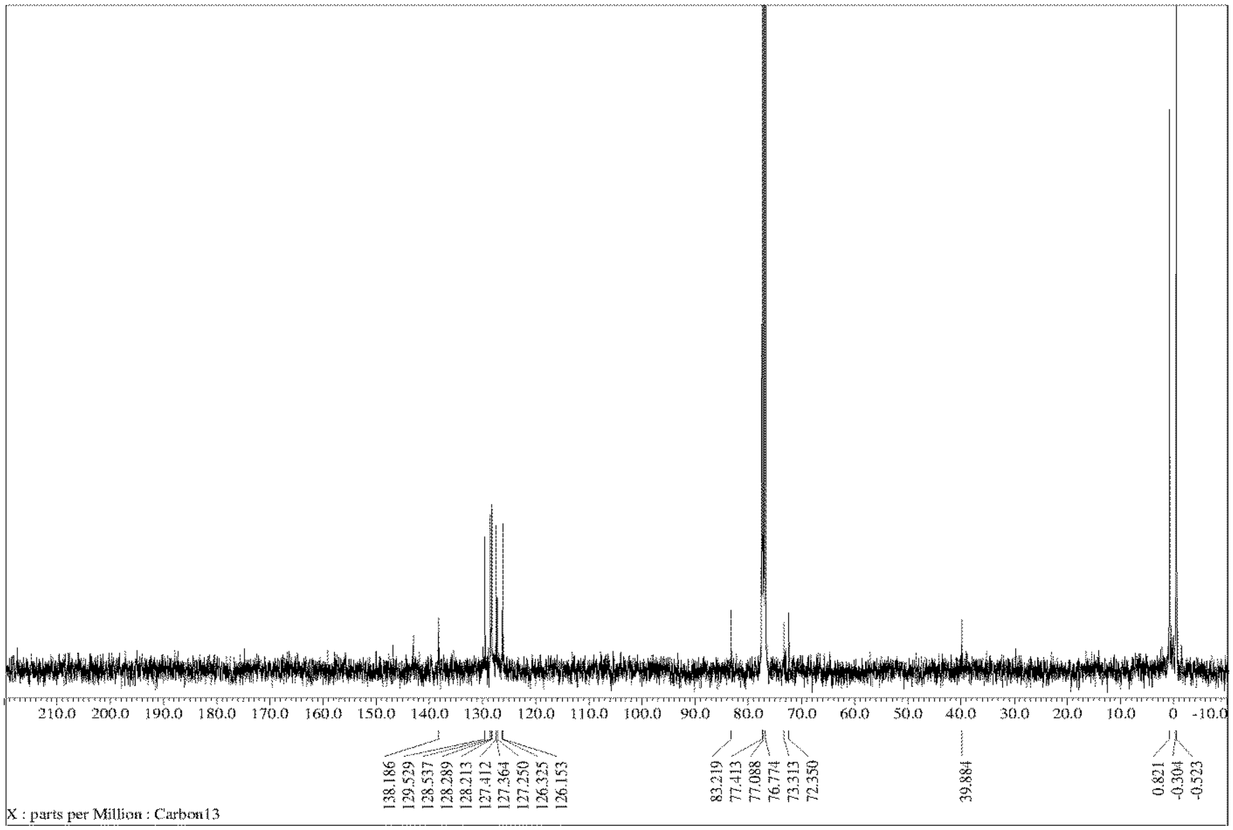
210.0 200.0 190.0 180.0 170.0 160.0 150.0 140.0 130.0 120.0 110.0 100.0 90.0 80.0 70.0 60.0 50.0 40.0 30.0 20.0 10.0 0 -10.0
138.186 129.529 128.537 128.289 128.213 127.412 127.364 127.250 126.325 126.153
83.219 77.413 77.088 76.774 73.313 72.350
39.884
0.821 -0.304 -0.523
X : parts per Million : Carbon13

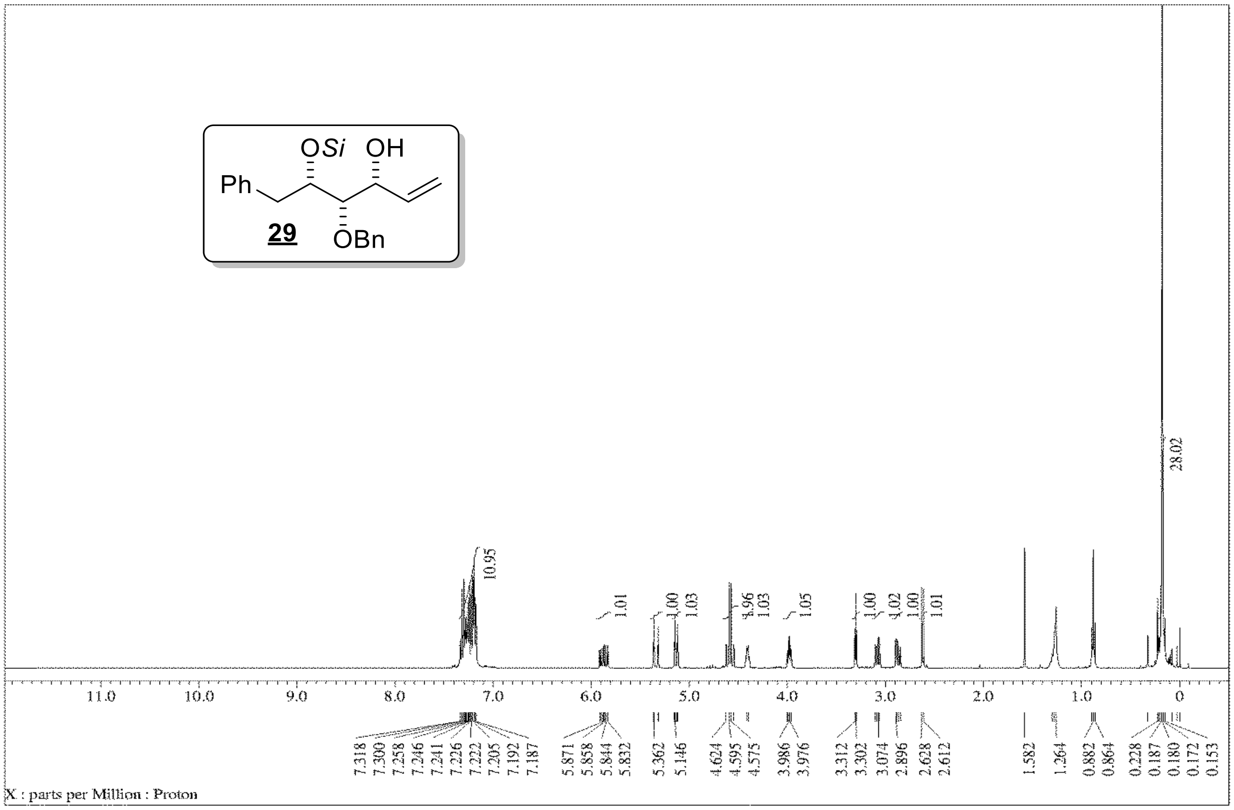
OSi OH
Ph
29
OBn
10.95
1.01
1.00
1.03
1.96
1.03
1.05
1.00
1.02
1.00
1.01
28.02
7.318 7.300 7.258 7.246 7.241 7.226 7.222 7.205 7.192 7.187
5.871 5.858 5.844 5.832
5.362 5.146
4.624 4.595 4.575
3.986 3.976
3.312 3.302 3.074 2.896 2.628 2.612
1.582 1.264 0.882 0.864
0.228 0.187 0.180 0.172 0.153
X : parts per Million : Proton

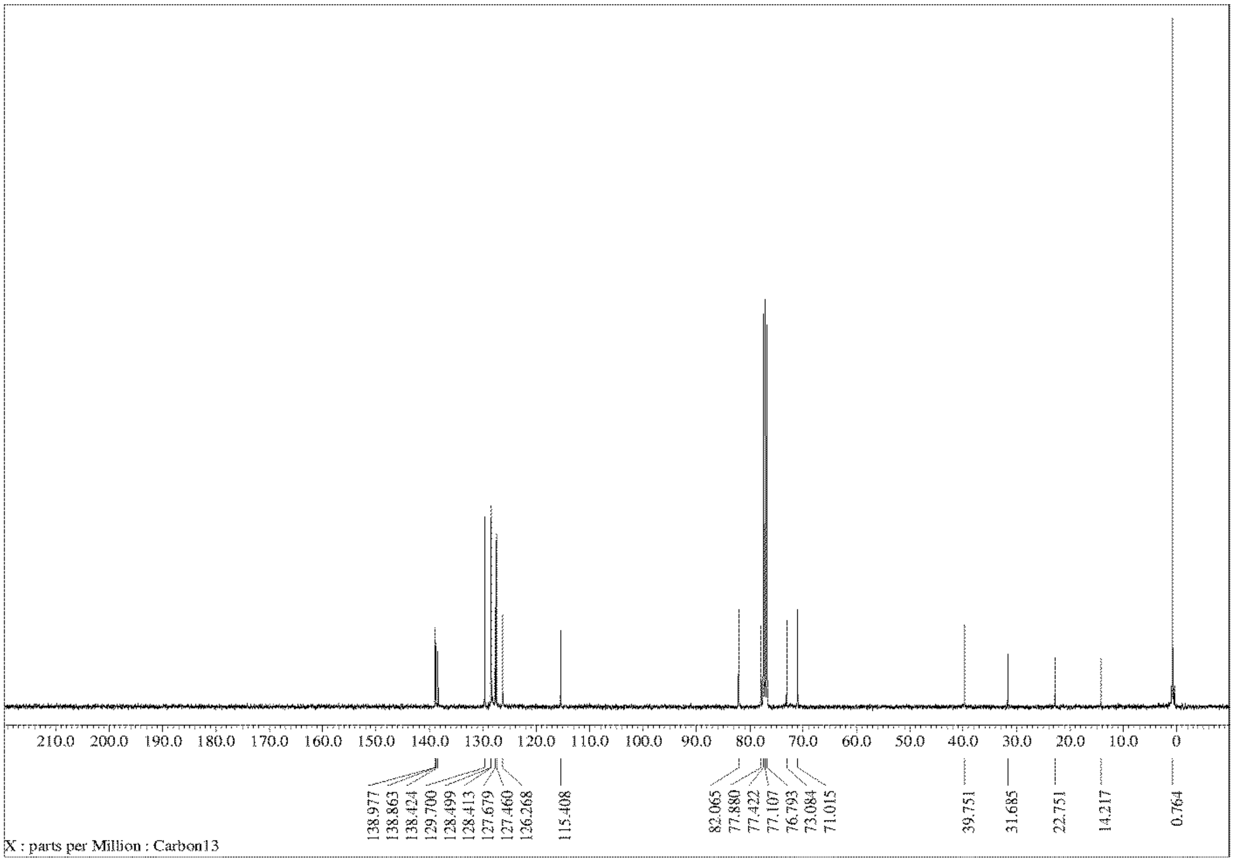
138.977 138.863 138.424 129.700 128.499 128.413 127.679 127.460 126.268
115.408
82.065 77.880 77.422 77.107 76.793 73.084 71.015
39.751 31.685 22.751 14.217 0.764
X : parts per Million : Carbon13

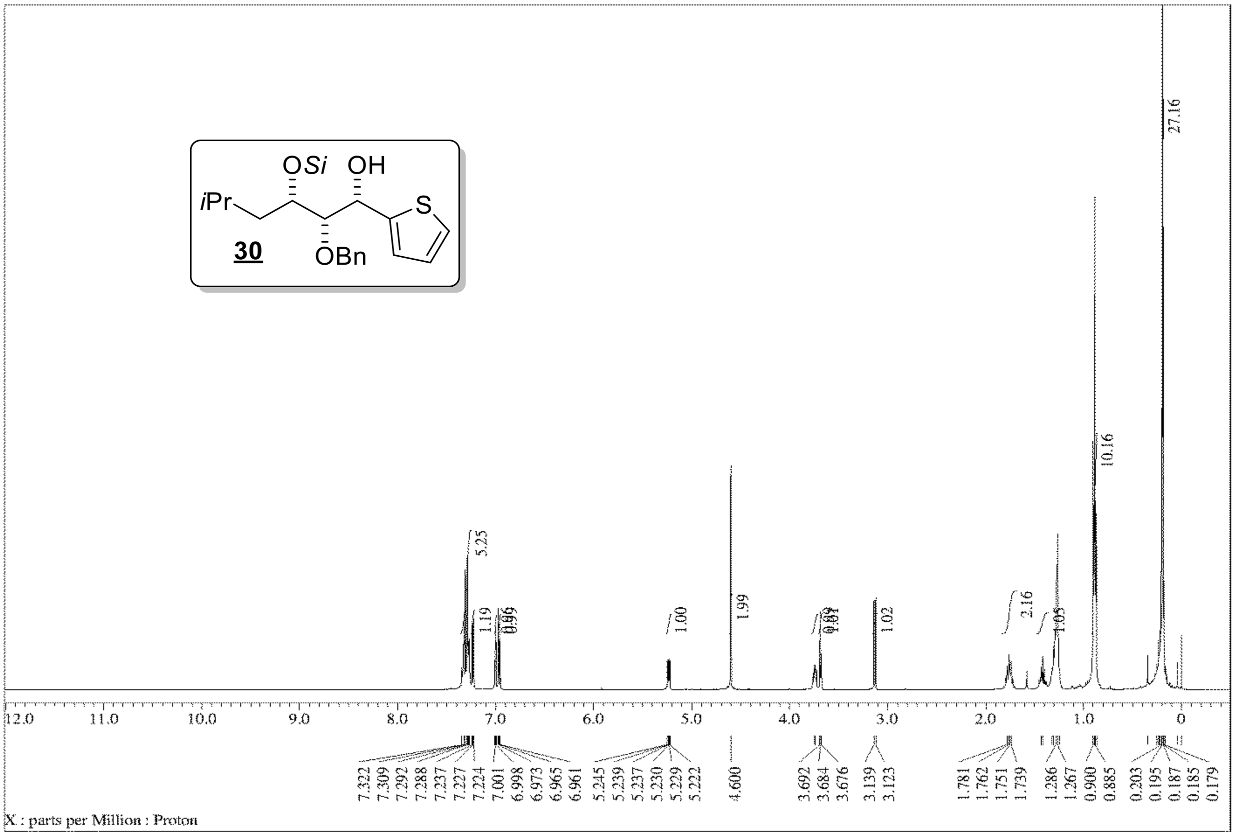

OSi OH
iPr
S
30
OBn
27.16
10.16
5.25
1.19
0.96
1.00
1.99
1.02
2.16
1.05
12.0 11.0 10.0 9.0 8.0 7.0 6.0 5.0 4.0 3.0 2.0 1.0 0
7.322 7.309 7.292 7.288 7.237 7.227 7.224 7.001 6.998 6.973 6.965 6.961
5.245 5.239 5.237 5.230 5.229 5.222
4.600
3.692 3.684 3.676
3.139 3.123
1.781 1.762 1.751 1.739
1.286 1.267 0.900 0.885
0.203 0.195 0.187 0.185 0.179
X : parts per Million : Proton

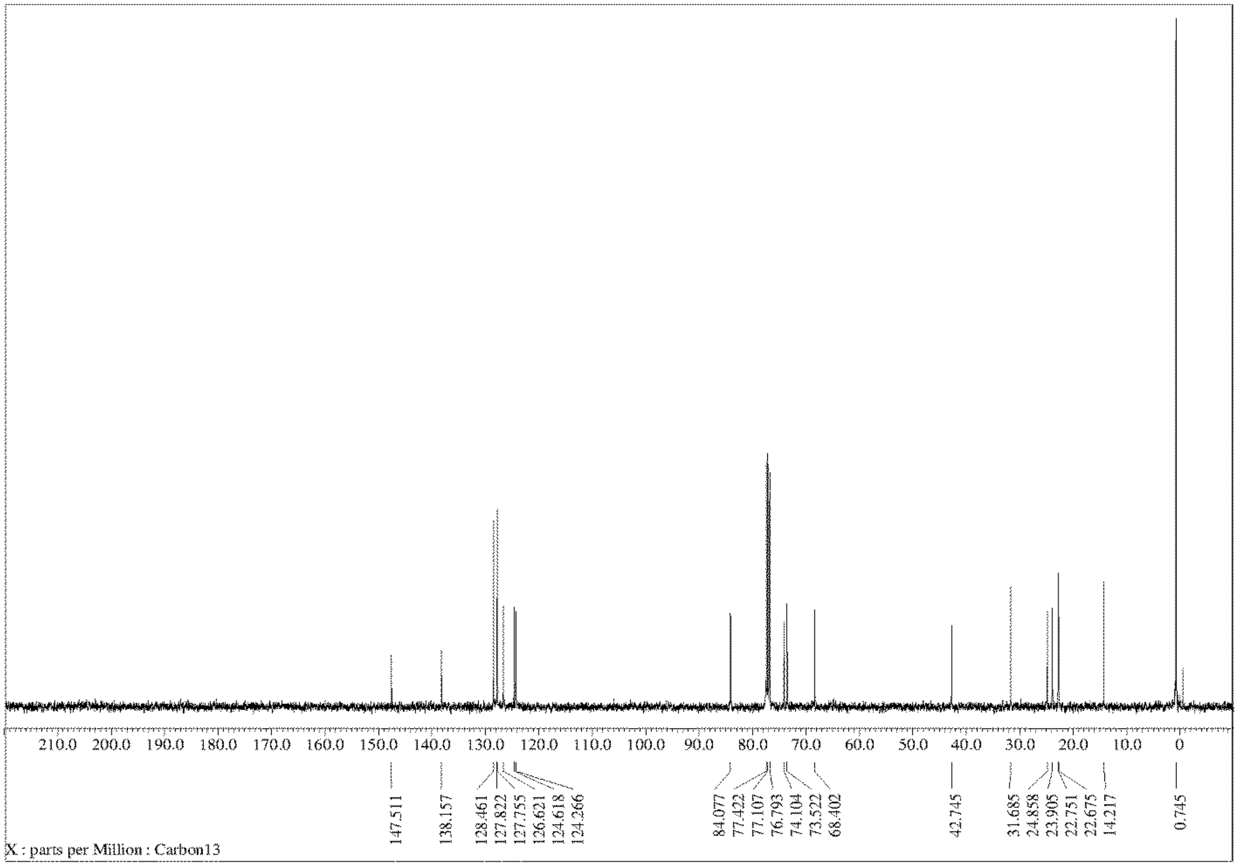

210.0 200.0 190.0 180.0 170.0 160.0 150.0 140.0 130.0 120.0 110.0 100.0 90.0 80.0 70.0 60.0 50.0 40.0 30.0 20.0 10.0 0
147.511
138.157
128.461 127.822 127.755 126.621 124.618 124.266
84.077 77.422 77.107 76.793 74.104 73.522 68.402
42.745
31.685 24.858 23.905 22.751 22.675 14.217
0.745
X : parts per Million : Carbon13

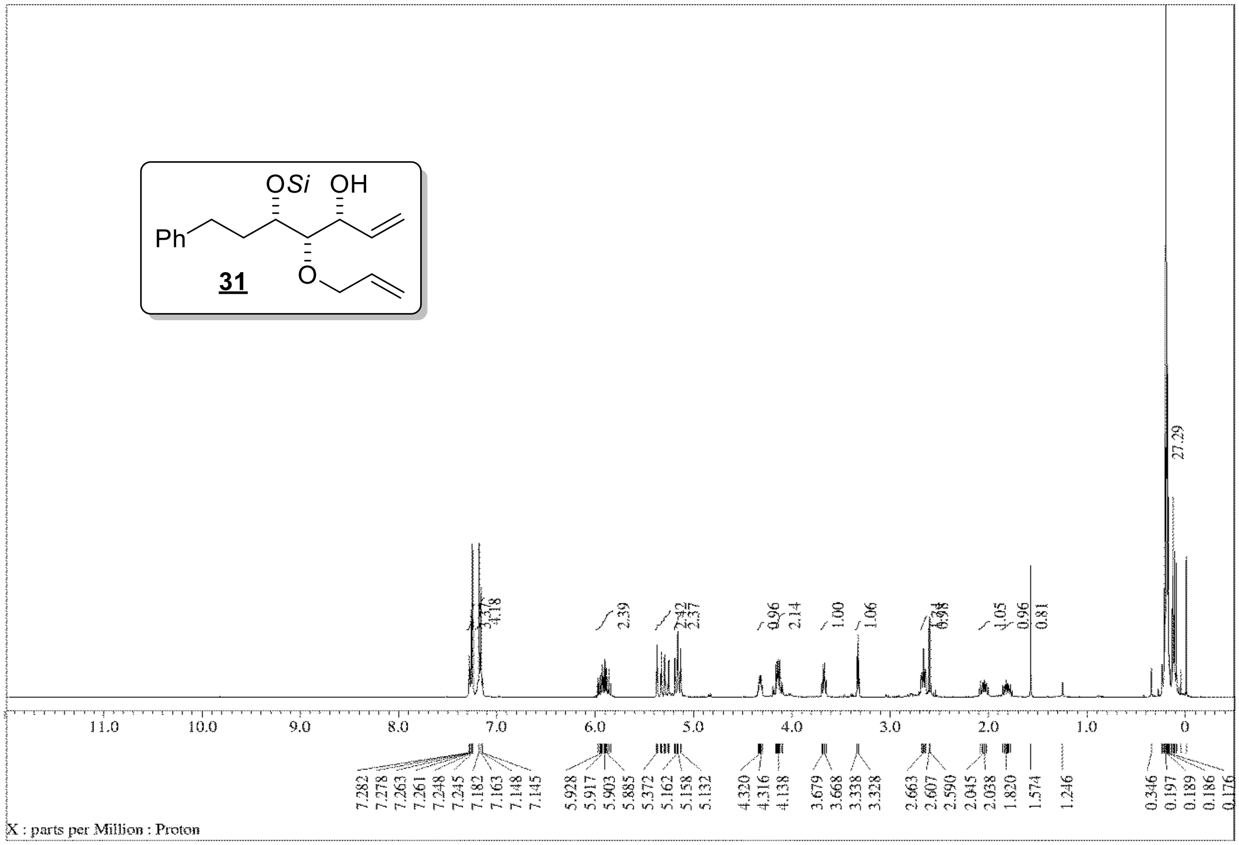
OSi OH
Ph
31
27.29
2.39
2.14
1.00
1.06
1.05
0.96
0.81
7.282 7.278 7.263 7.261 7.248 7.245 7.182 7.163 7.148 7.145
5.928 5.917 5.903 5.885 5.372 5.162 5.158 5.132
4.320 4.316 4.138
3.679 3.668 3.338 3.328
2.663 2.607 2.590 2.045 2.038 1.820 1.574 1.246
0.346 0.197 0.189 0.186 0.176
X : parts per Million : Proton

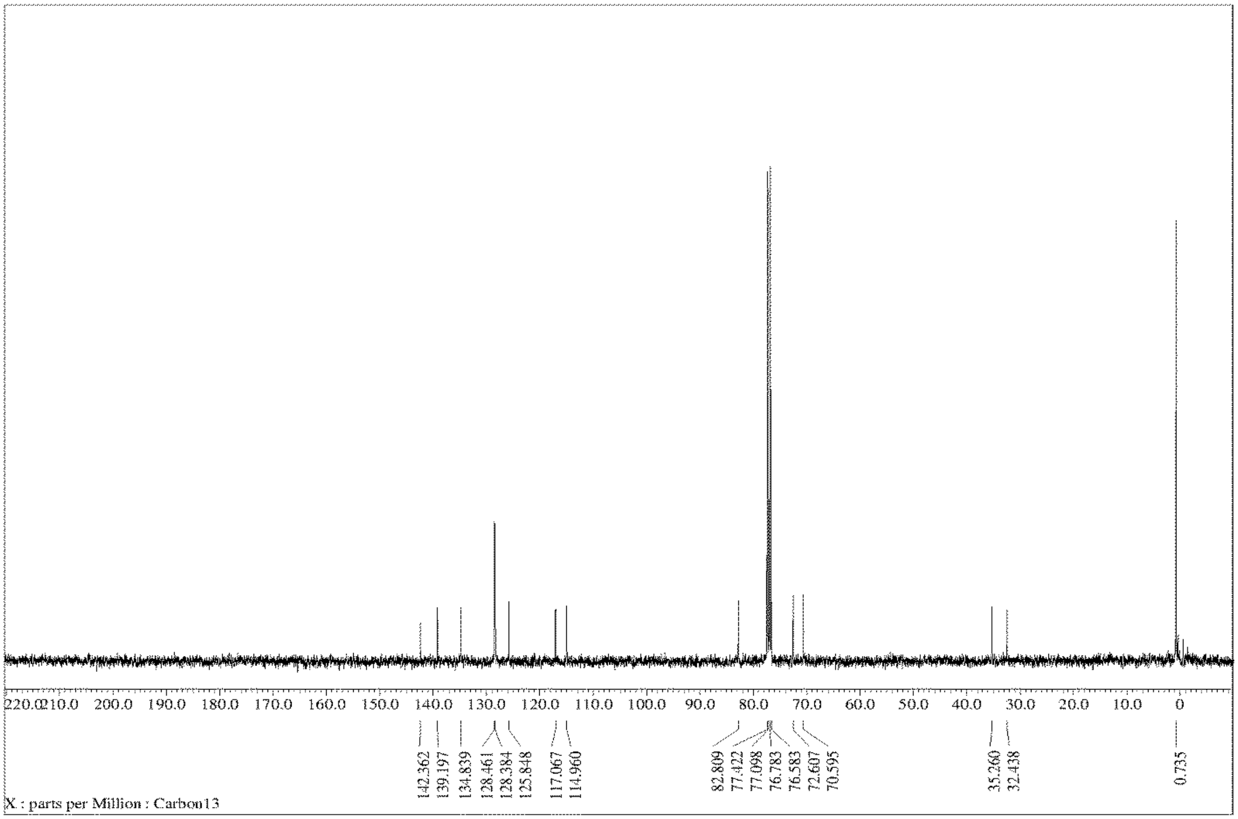
142.362 139.197 134.839 128.461 128.384 125.848 117.067 114.960
82.809 77.422 77.098 76.783 76.583 72.607 70.595
35.260 32.438
0.735
X : parts per Million : Carbon13

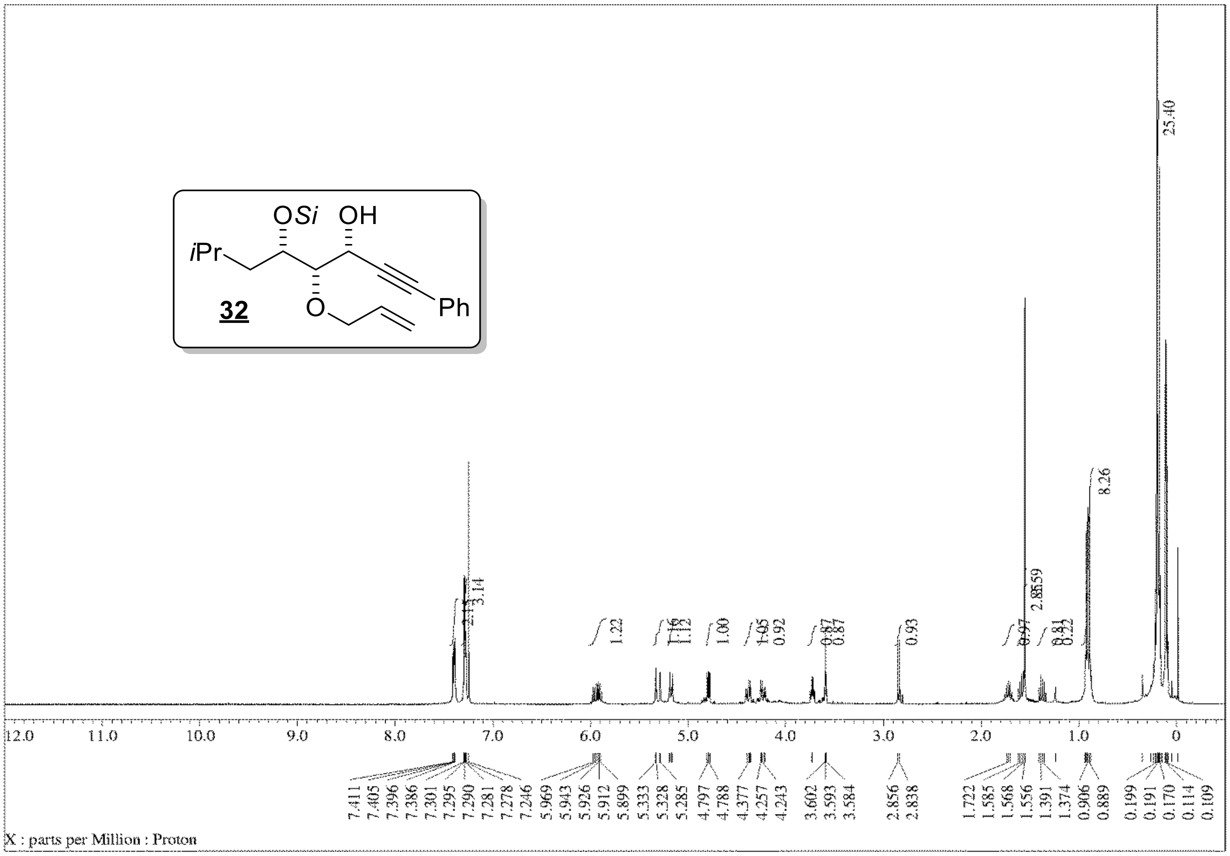
OSi OH
iPr
32
O
Ph
25.40
8.26
3.14
2.85
1.22
1.16
1.12
1.00
1.05
0.92
0.87
0.87
0.93
0.97
0.81
0.22
12.0 11.0 10.0 9.0 8.0 7.0 6.0 5.0 4.0 3.0 2.0 1.0 0
7.411 7.405 7.396 7.386 7.301 7.295 7.290 7.281 7.278 7.246 5.969 5.943 5.926 5.912 5.899 5.333 5.328 5.285 4.797 4.788 4.377 4.257 4.243 3.602 3.593 3.584 2.856 2.838 1.722 1.585 1.568 1.556 1.391 1.374 0.906 0.889 0.199 0.191 0.170 0.114 0.109
X : parts per Million : Proton

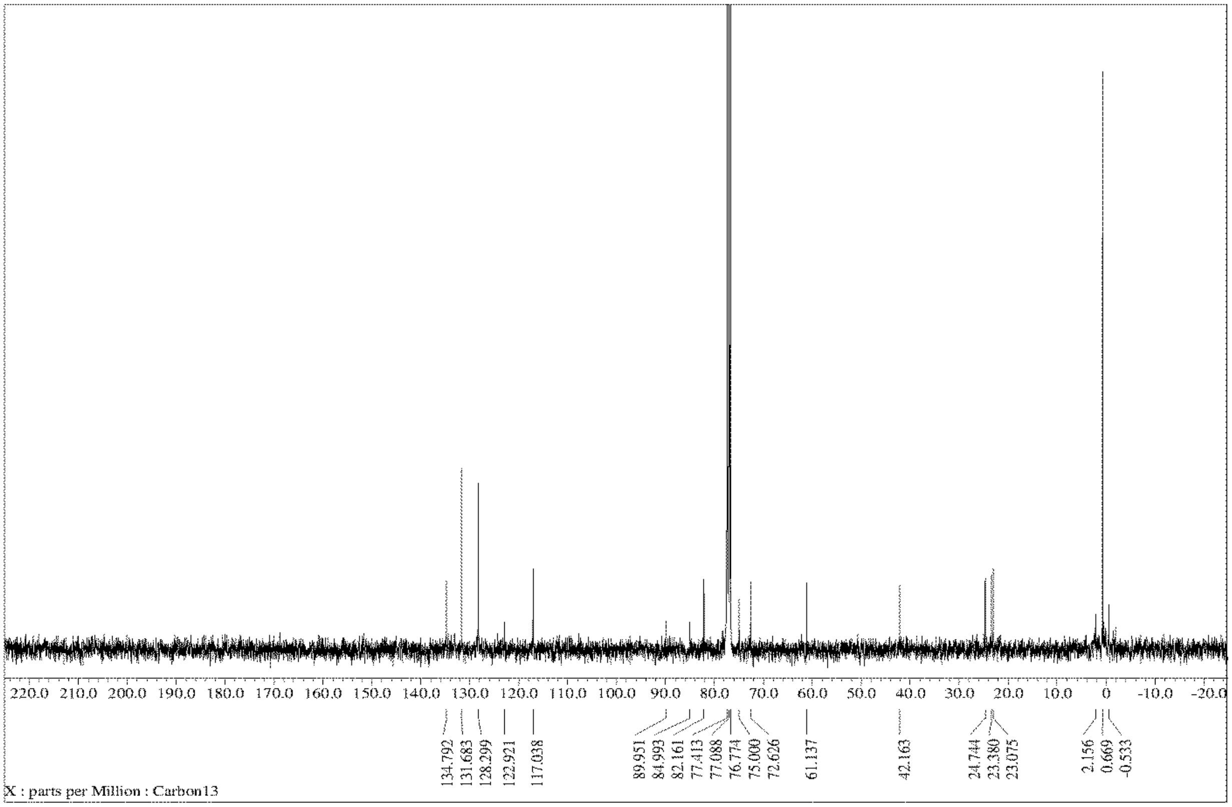
220.0 210.0 200.0 190.0 180.0 170.0 160.0 150.0 140.0 130.0 120.0 110.0 100.0 90.0 80.0 70.0 60.0 50.0 40.0 30.0 20.0 10.0 0 -10.0 -20.0
134.792 131.683 128.299 122.921 117.038 89.951 84.993 82.161 77.413 77.088 76.774 75.000 72.626 61.137 42.163 24.744 23.380 23.075 2.156 0.669 -0.533
X : parts per Million : Carbon13

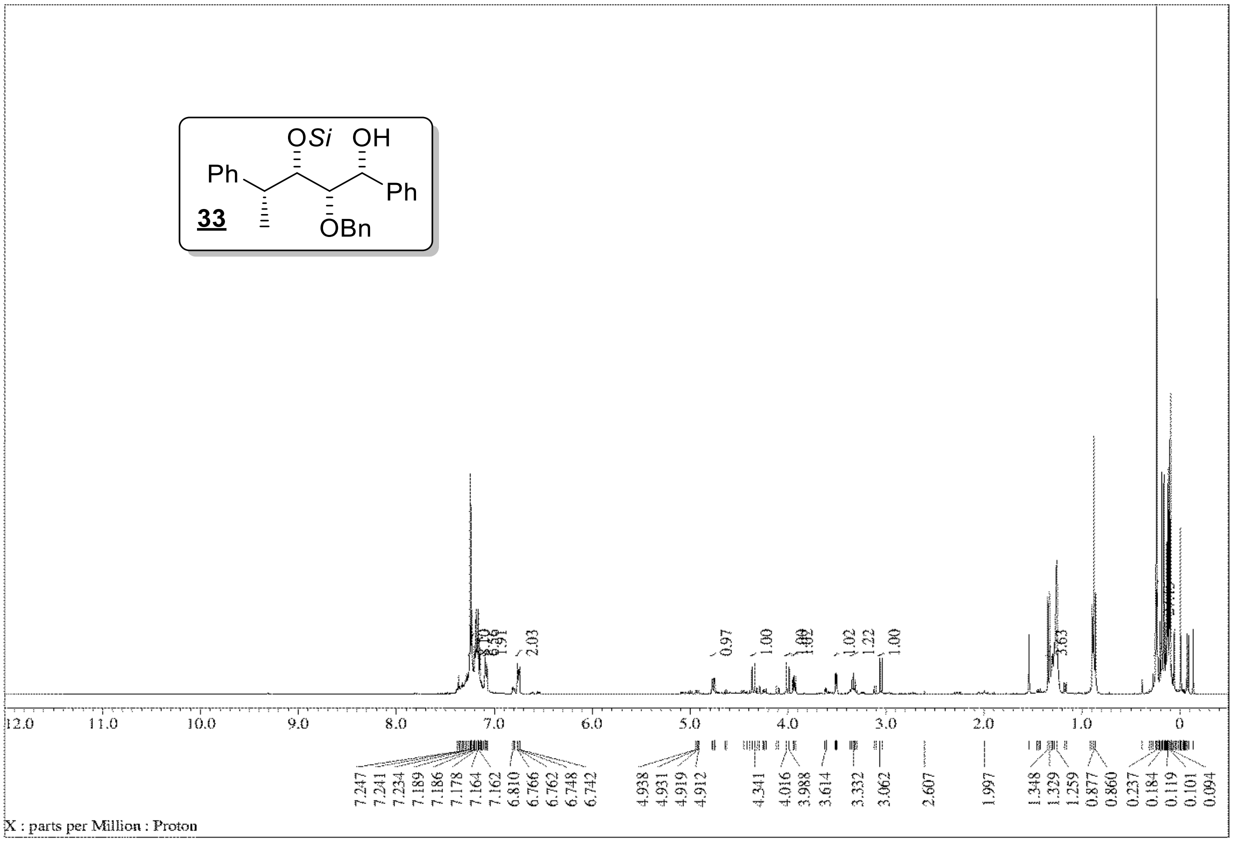
OSi OH
Ph
Ph
33
OBn
0.97
1.00
1.00
1.02
1.02
1.22
1.00
3.63
2.03
1.91
12.0 11.0 10.0 9.0 8.0 7.0 6.0 5.0 4.0 3.0 2.0 1.0 0
7.247
7.241
7.234
7.189
7.186
7.178
7.164
7.162
6.810
6.766
6.762
6.748
6.742
4.938
4.931
4.919
4.912
4.341
4.016
3.988
3.614
3.332
3.062
2.607
1.997
1.348
1.329
1.259
0.877
0.860
0.237
0.184
0.119
0.101
0.094
X : parts per Million : Proton

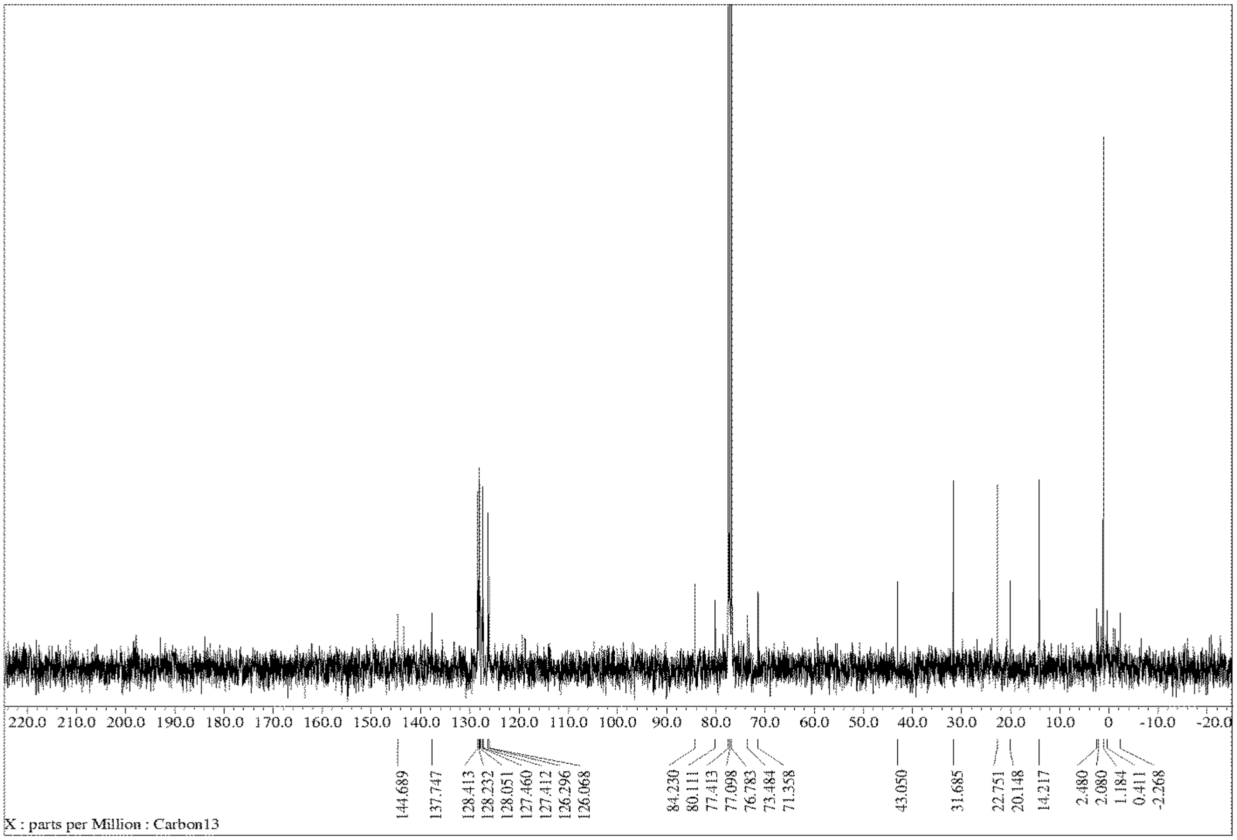
220.0 210.0 200.0 190.0 180.0 170.0 160.0 150.0 140.0 130.0 120.0 110.0 100.0 90.0 80.0 70.0 60.0 50.0 40.0 30.0 20.0 10.0 0 -10.0 -20.0
144.689
137.747
128.413
128.232
128.051
127.460
127.412
126.296
126.068
84.230
80.111
77.413
77.098
76.783
73.484
71.358
43.050
31.685
22.751
20.148
14.217
2.480
2.080
1.184
0.411
-2.268
X : parts per Million : Carbon13

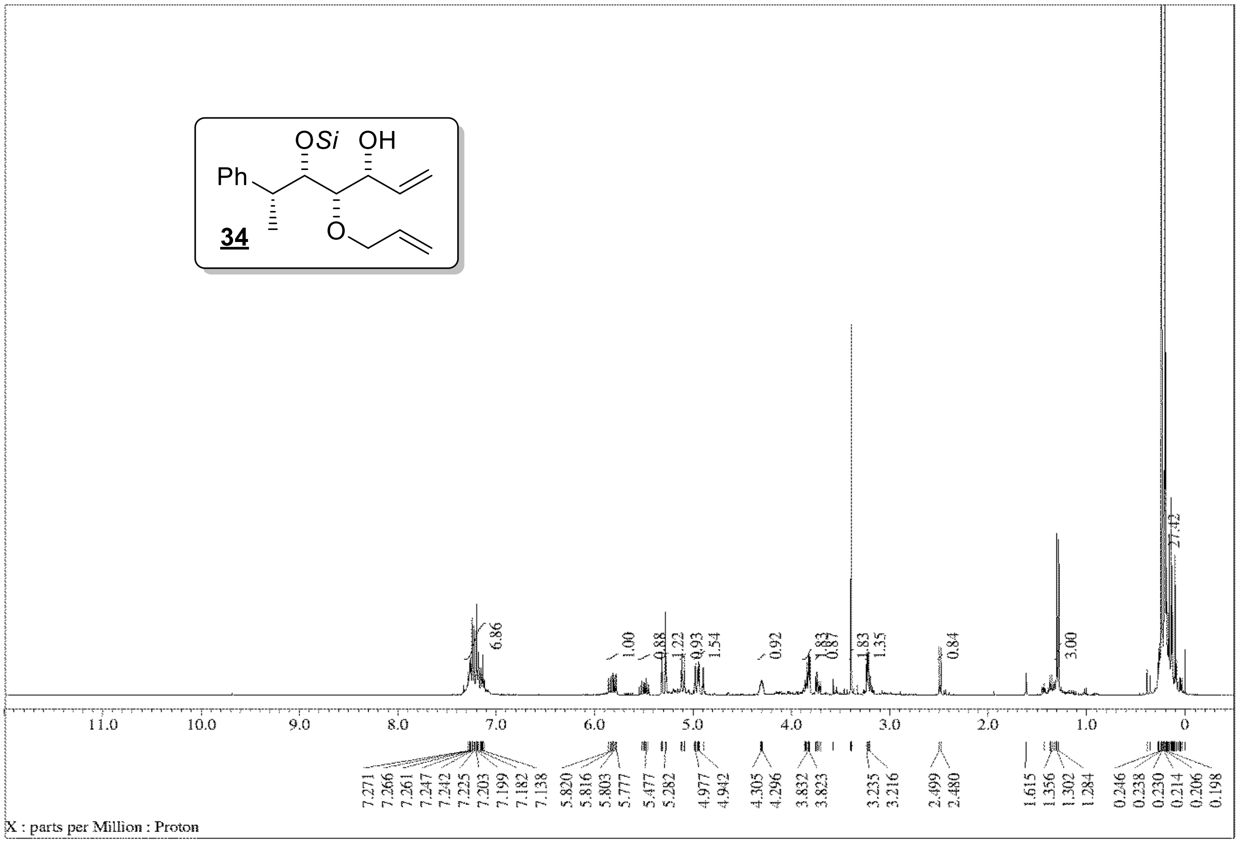
OSi OH
Ph
34
6.86
1.00
0.88
1.22
0.93
1.54
0.92
1.83
0.87
1.83
1.35
0.84
3.00
27.42
11.0 10.0 9.0 8.0 7.0 6.0 5.0 4.0 3.0 2.0 1.0 0
7.271 7.266 7.261 7.247 7.242 7.225 7.203 7.199 7.182 7.138
5.820 5.816 5.803 5.777
5.477 5.282
4.977 4.942
4.305 4.296
3.832 3.823
3.235 3.216
2.499 2.480
1.615 1.356 1.302 1.284
0.246 0.238 0.230 0.214 0.206 0.198
X : parts per Million : Proton

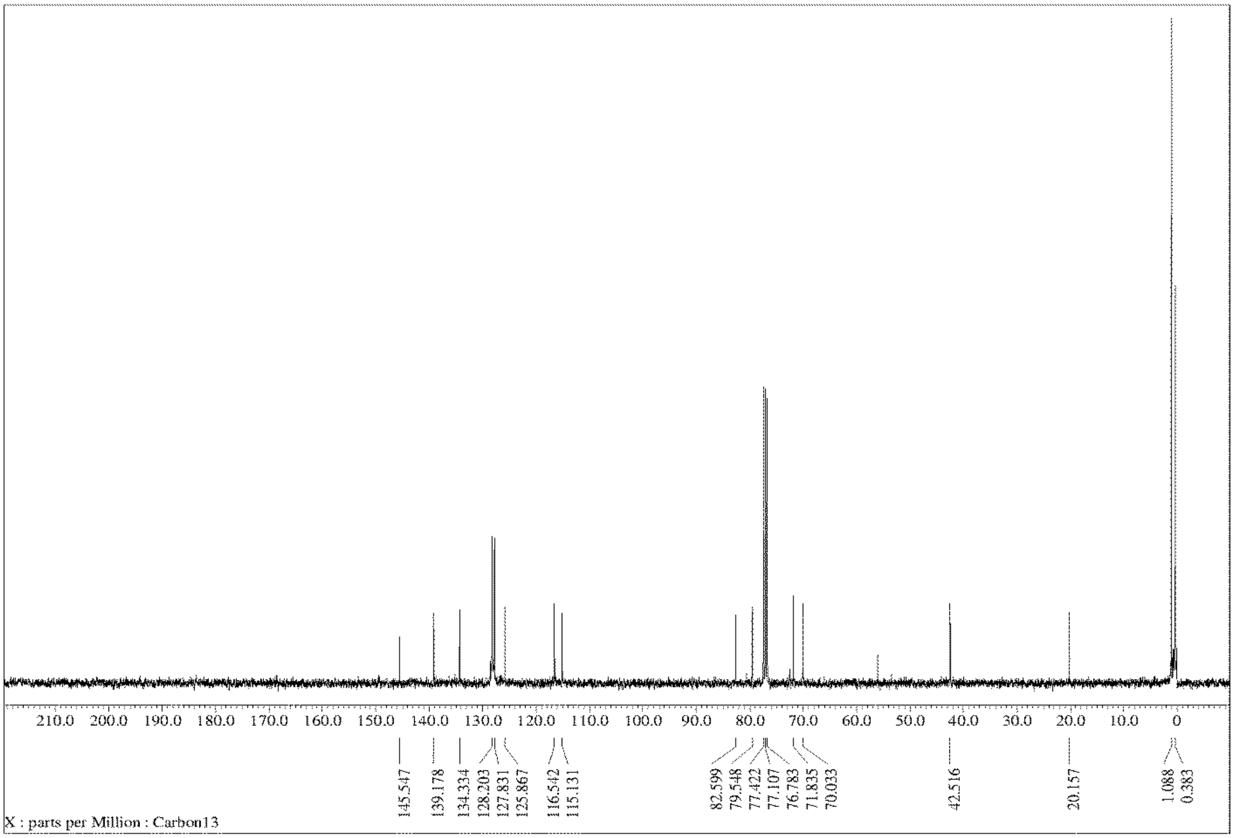
210.0 200.0 190.0 180.0 170.0 160.0 150.0 140.0 130.0 120.0 110.0 100.0 90.0 80.0 70.0 60.0 50.0 40.0 30.0 20.0 10.0 0
145.547
139.178
134.334
128.203
127.831
125.867
116.542
115.131
82.599
79.548
77.422
77.107
76.783
71.835
70.033
42.516
20.157
1.088
0.383
X : parts per Million : Carbon13

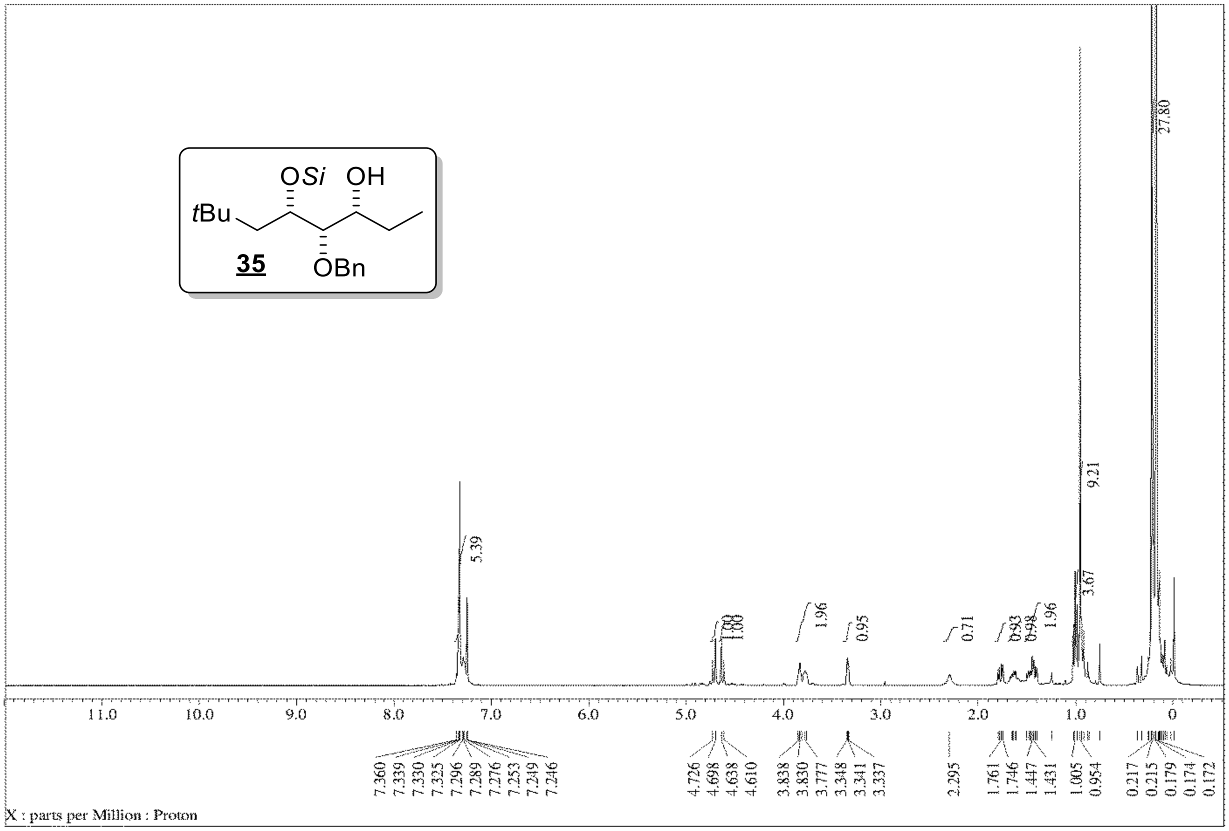
OSi OH
tBu
35
OBn
5.39
1.96
0.95
0.71
0.93
0.98
1.96
9.21
3.67
27.80
11.0 10.0 9.0 8.0 7.0 6.0 5.0 4.0 3.0 2.0 1.0 0
7.360 7.339 7.330 7.325 7.296 7.289 7.276 7.253 7.249 7.246
4.726 4.698 4.638 4.610
3.838 3.830 3.777 3.348 3.341 3.337
2.295
1.761 1.746 1.447 1.431
1.005 0.954
0.217 0.215 0.179 0.174 0.172
X : parts per Million : Proton

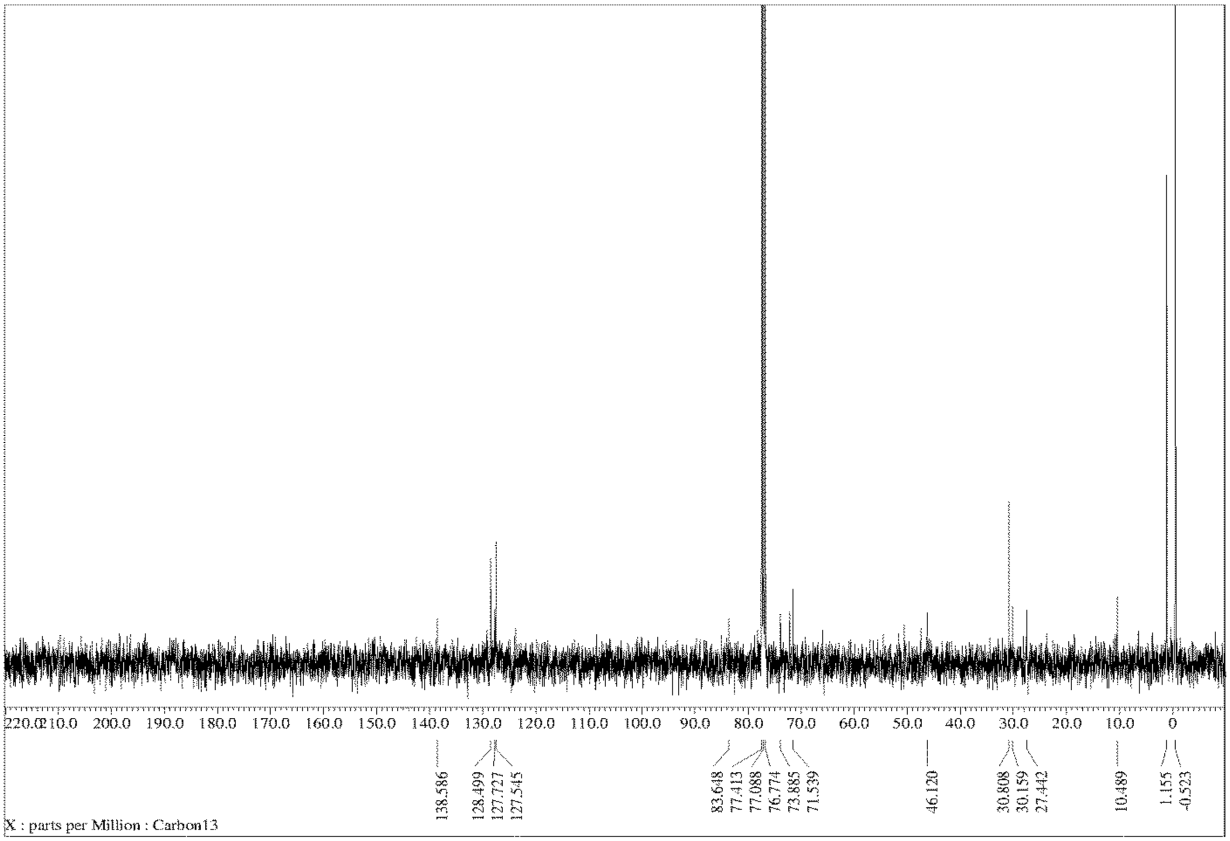
220.0 210.0 200.0 190.0 180.0 170.0 160.0 150.0 140.0 130.0 120.0 110.0 100.0 90.0 80.0 70.0 60.0 50.0 40.0 30.0 20.0 10.0 0
138.586
128.499 127.727 127.545
83.648 77.413 77.088 76.774 73.885 71.539
46.120
30.808 30.159 27.442
10.489
1.155 -0.523
X : parts per Million : Carbon13

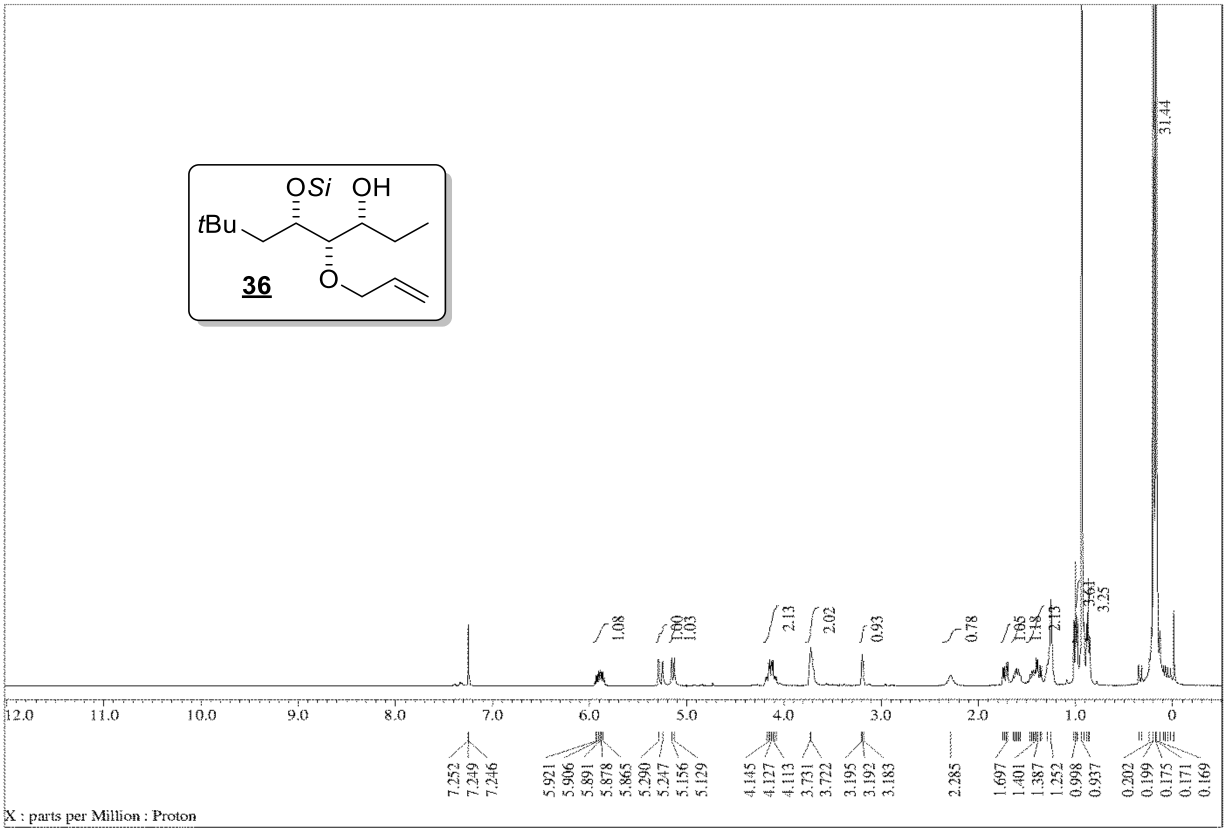

OSi
OH
tBu
36
O
1.08
1.00
1.03
2.13
2.02
0.93
0.78
1.05
1.18
2.15
3.61
3.25
31.44
12.0 11.0 10.0 9.0 8.0 7.0 6.0 5.0 4.0 3.0 2.0 1.0 0
7.252 7.249 7.246
5.921 5.906 5.891 5.878 5.865 5.290 5.247 5.156 5.129
4.145 4.127 4.113 3.731 3.722 3.195 3.192 3.183
2.285
1.697 1.401 1.387 1.252 0.998 0.937
0.202 0.199 0.175 0.171 0.169
X : parts per Million : Proton

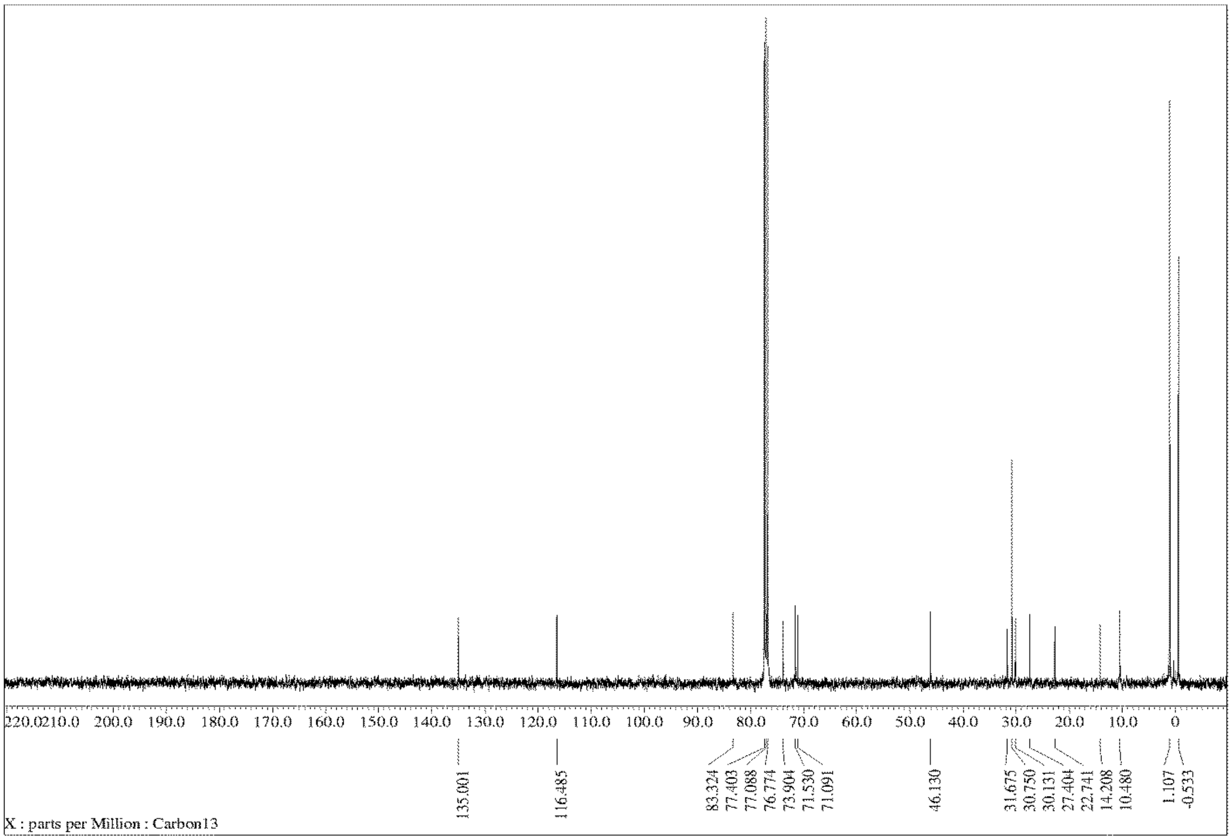

220.0 210.0 200.0 190.0 180.0 170.0 160.0 150.0 140.0 130.0 120.0 110.0 100.0 90.0 80.0 70.0 60.0 50.0 40.0 30.0 20.0 10.0 0
135.001
116.485
83.324 77.403 77.088 76.774 73.904 71.530 71.091
46.130
31.675 30.750 30.131 27.404 22.741 14.208 10.480
1.107 -0.533
X : parts per Million : Carbon13

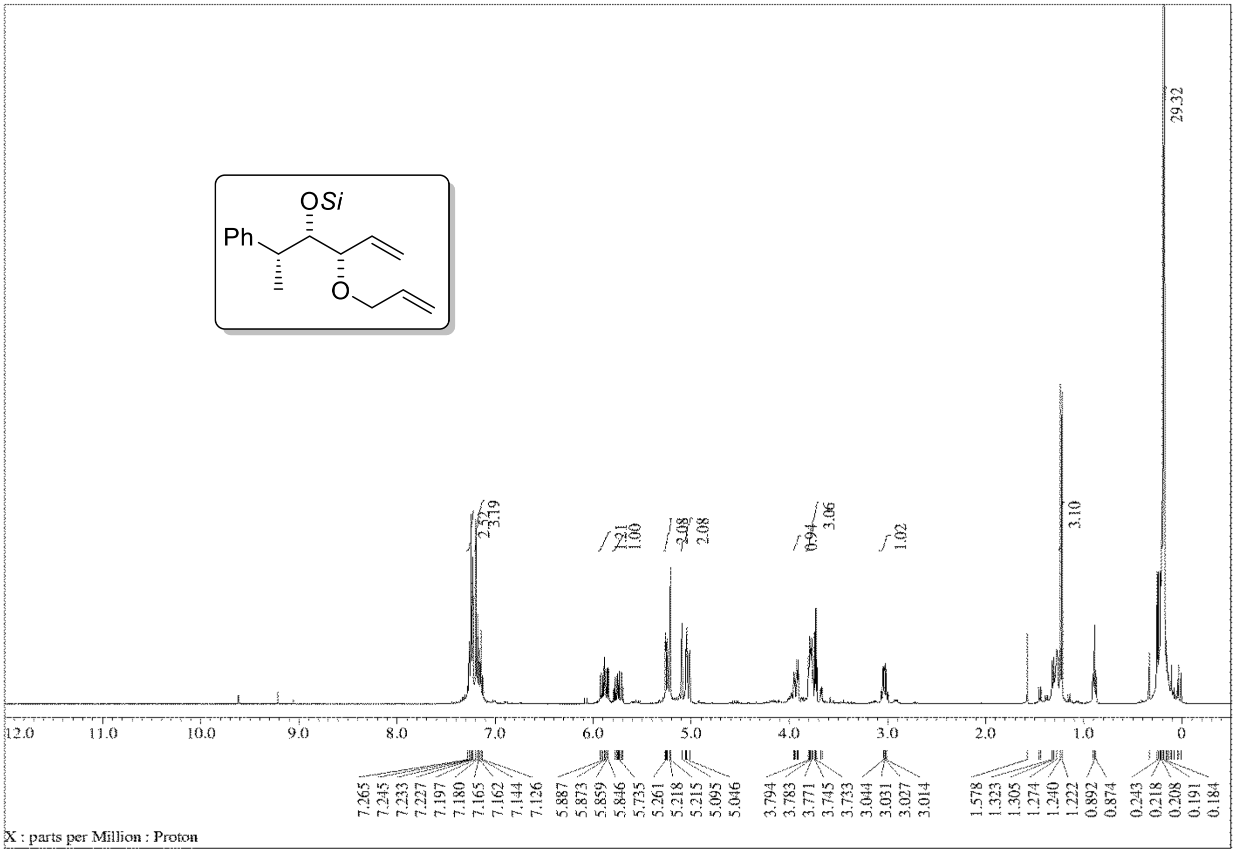
OSi
Ph
O
29.32
3.10
2.52
3.19
1.21
1.00
2.08
2.08
0.94
3.06
1.02
12.0 11.0 10.0 9.0 8.0 7.0 6.0 5.0 4.0 3.0 2.0 1.0 0
7.265 7.245 7.233 7.227 7.197 7.180 7.165 7.162 7.144 7.126
5.887 5.873 5.859 5.846 5.735 5.261 5.218 5.215 5.095 5.046
3.794 3.783 3.771 3.745 3.733 3.044 3.031 3.027 3.014
1.578 1.323 1.305 1.274 1.240 1.222 0.892 0.874
0.243 0.218 0.208 0.191 0.184
X : parts per Million : Proton

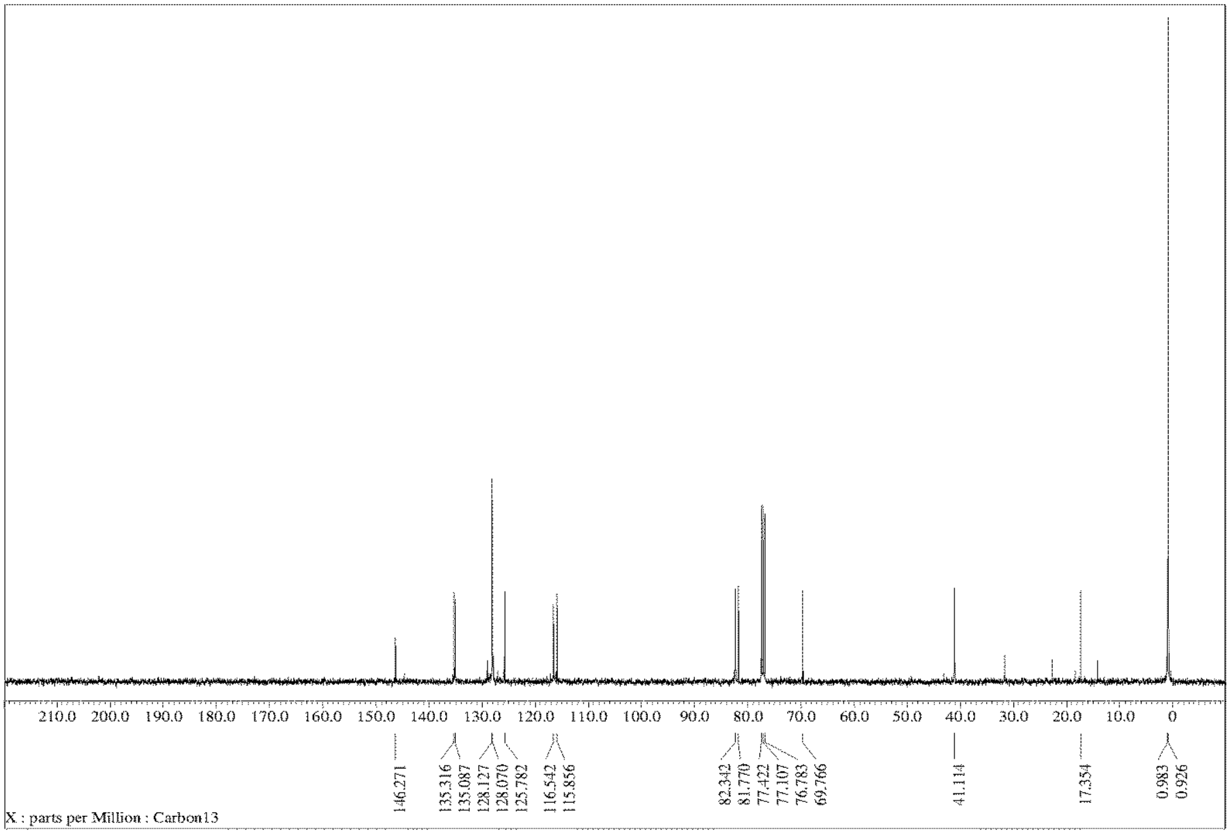
210.0 200.0 190.0 180.0 170.0 160.0 150.0 140.0 130.0 120.0 110.0 100.0 90.0 80.0 70.0 60.0 50.0 40.0 30.0 20.0 10.0 0
146.271
135.316 135.087 128.127 128.070 125.782
116.542 115.856
82.342 81.770 77.422 77.107 76.783 69.766
41.114
17.354
0.983 0.926
X : parts per Million : Carbon13

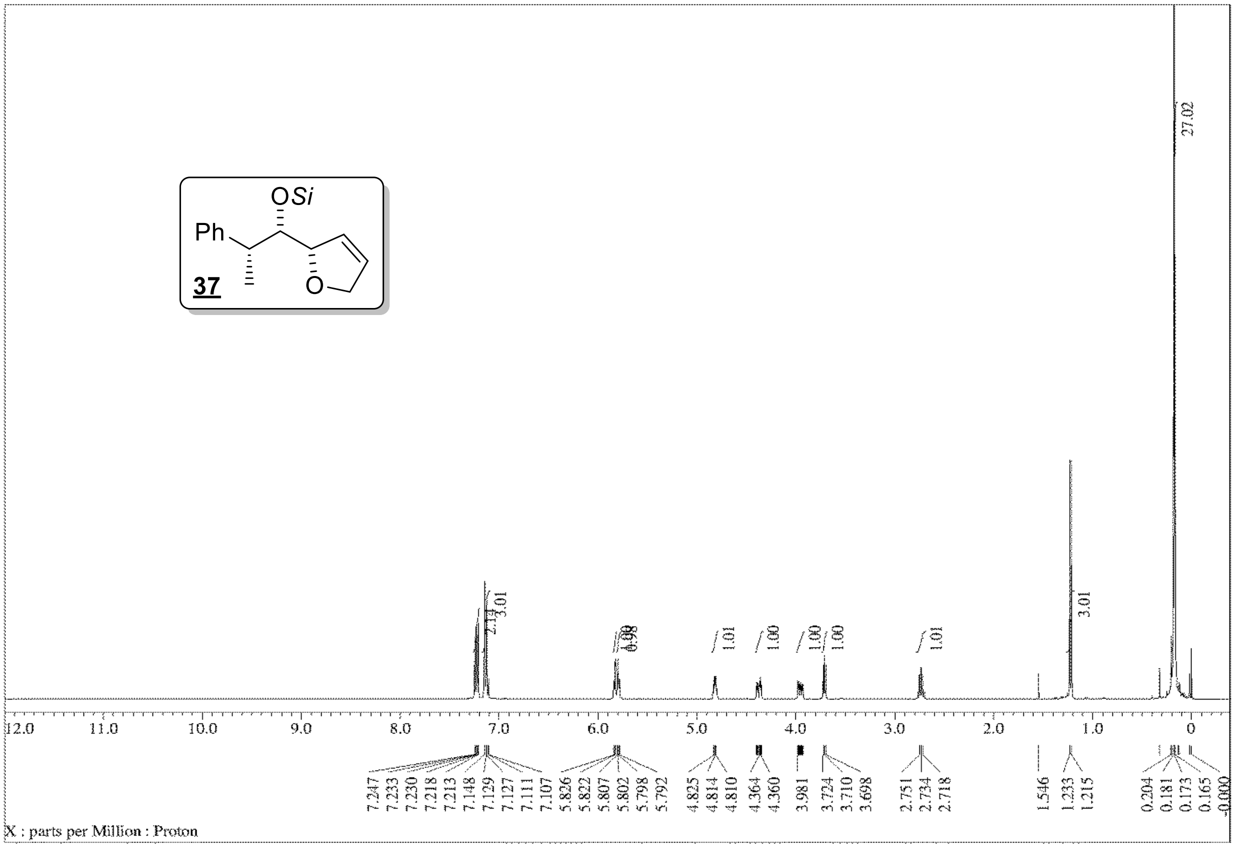
OSi
Ph
O
37
27.02
3.01
2.13
3.01
1.01
1.00
1.00
1.00
1.01
12.0 11.0 10.0 9.0 8.0 7.0 6.0 5.0 4.0 3.0 2.0 1.0 0
7.247 7.233 7.230 7.218 7.213 7.148 7.129 7.127 7.111 7.107
5.826 5.822 5.807 5.802 5.798 5.792
4.825 4.814 4.810
4.364 4.360
3.981
3.724 3.710 3.698
2.751 2.734 2.718
1.546
1.233 1.215
0.204 0.181 0.173 0.165 -0.000
X : parts per Million : Proton

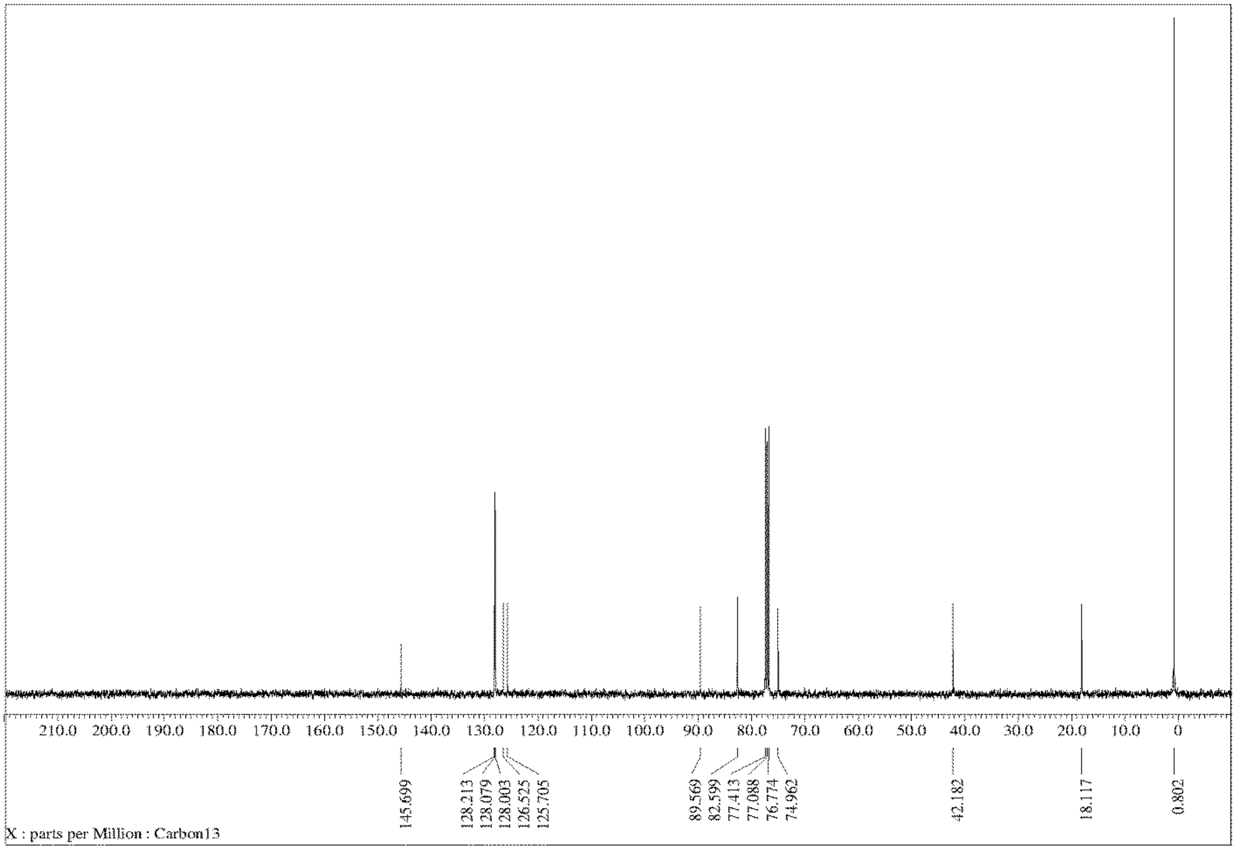
210.0 200.0 190.0 180.0 170.0 160.0 150.0 140.0 130.0 120.0 110.0 100.0 90.0 80.0 70.0 60.0 50.0 40.0 30.0 20.0 10.0 0
145.699
128.213 128.079 128.003 126.525 125.705
89.569 82.599 77.413 77.088 76.774 74.962
42.182
18.117
0.802
X : parts per Million : Carbon13

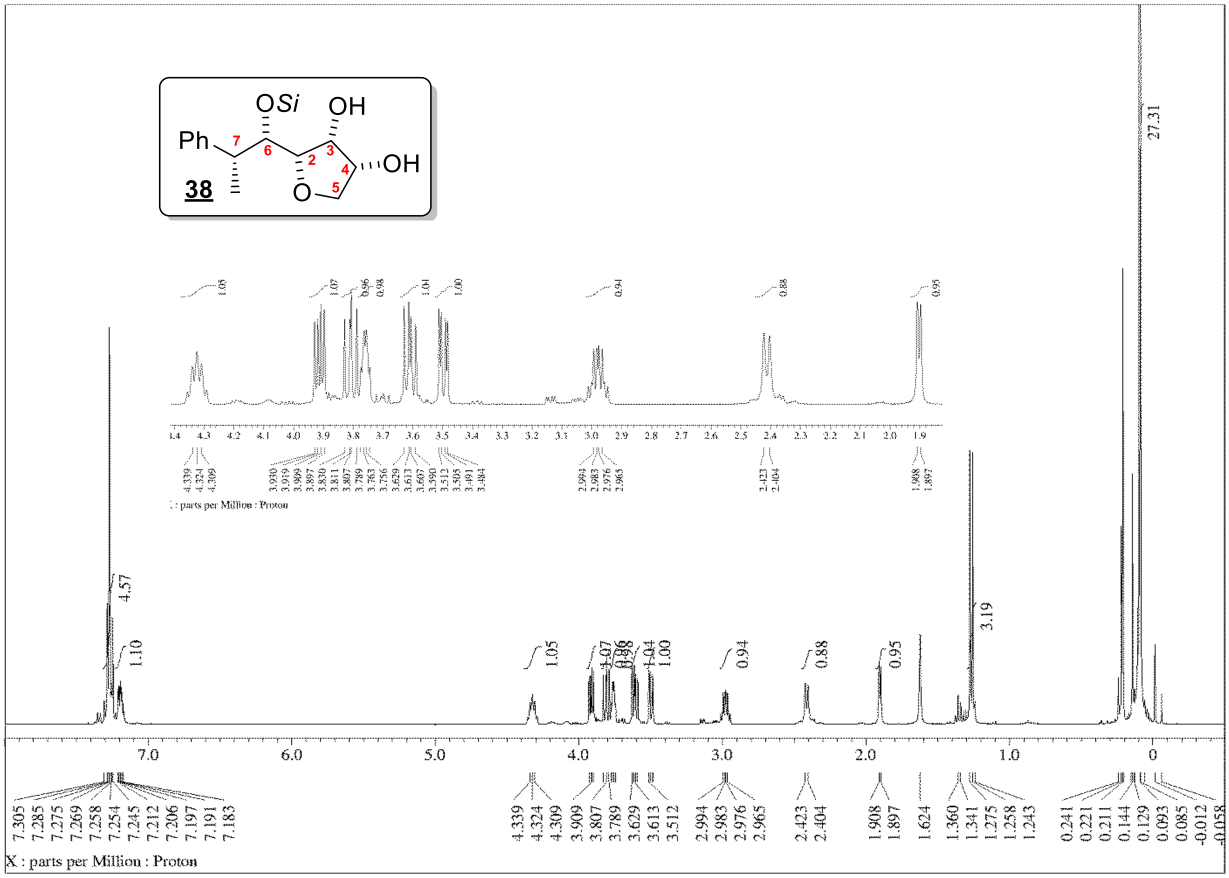

OSi
OH
Ph
7
6
2
3
4
OH
O
5
38
1.05
1.07
0.96
0.98
1.04
1.00
0.94
0.88
0.95
4.339 4.324 4.309
3.930 3.919 3.909 3.897 3.830 3.811 3.807 3.789 3.763 3.756 3.629 3.613 3.607 3.590 3.512 3.505 3.491 3.484
2.994 2.983 2.976 2.965
2.423 2.404
1.908 1.897
27.31
4.57
1.10
1.05
1.07
0.98
1.04
1.00
0.94
0.88
0.95
3.19
7.0
6.0
5.0
4.0
3.0
2.0
1.0
0
7.305 7.285 7.275 7.269 7.258 7.254 7.245 7.212 7.206 7.197 7.191 7.183
4.339 4.324 4.309 3.909 3.807 3.789 3.629 3.613 3.512
2.994 2.983 2.976 2.965
2.423 2.404
1.908 1.897
1.624
1.360 1.341 1.275 1.258 1.243
0.241 0.221 0.211 0.144 0.129 0.093 0.085 -0.012 -0.058
X : parts per Million : Proton

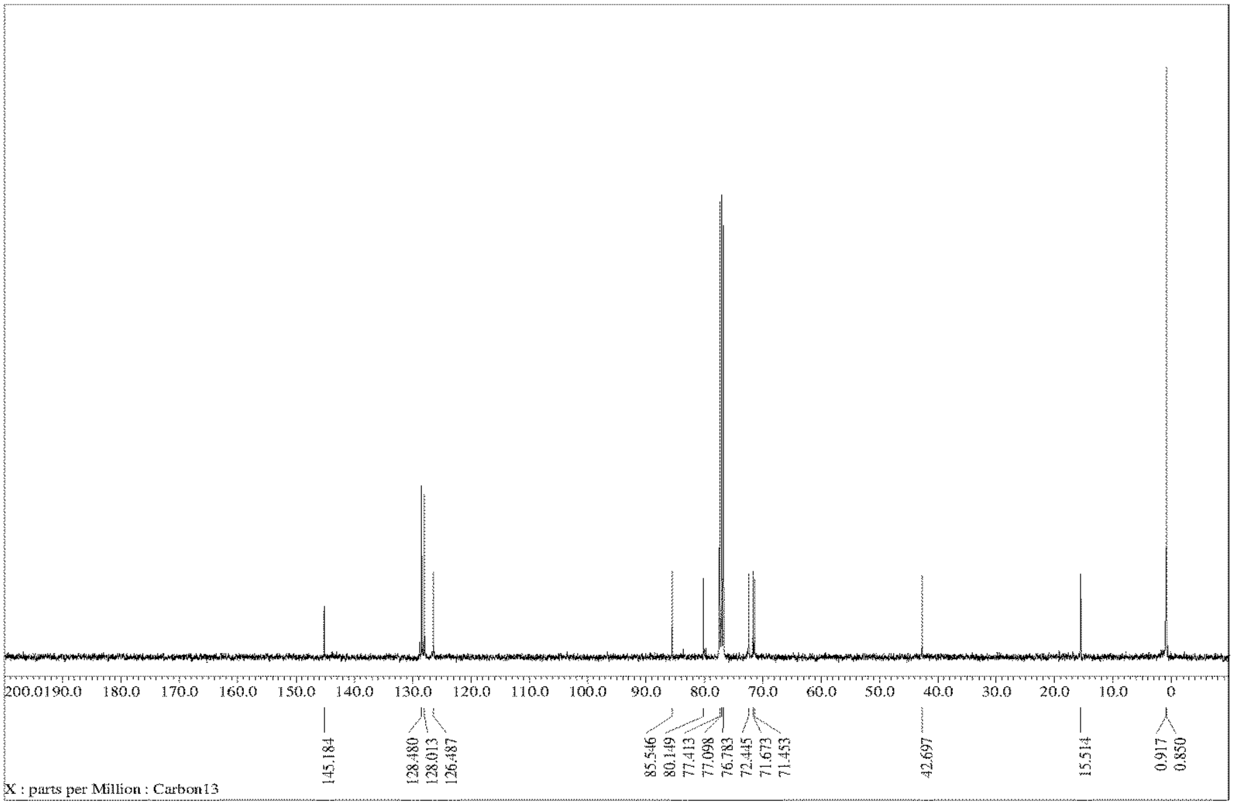

200.0 190.0 180.0 170.0 160.0 150.0 140.0 130.0 120.0 110.0 100.0 90.0 80.0 70.0 60.0 50.0 40.0 30.0 20.0 10.0 0
145.184
128.480 128.013 126.487
85.546 80.149 77.413 77.098 76.783 72.445 71.673 71.453
42.697
15.514
0.917 0.850
X : parts per Million : Carbon13

NOE Experiment :

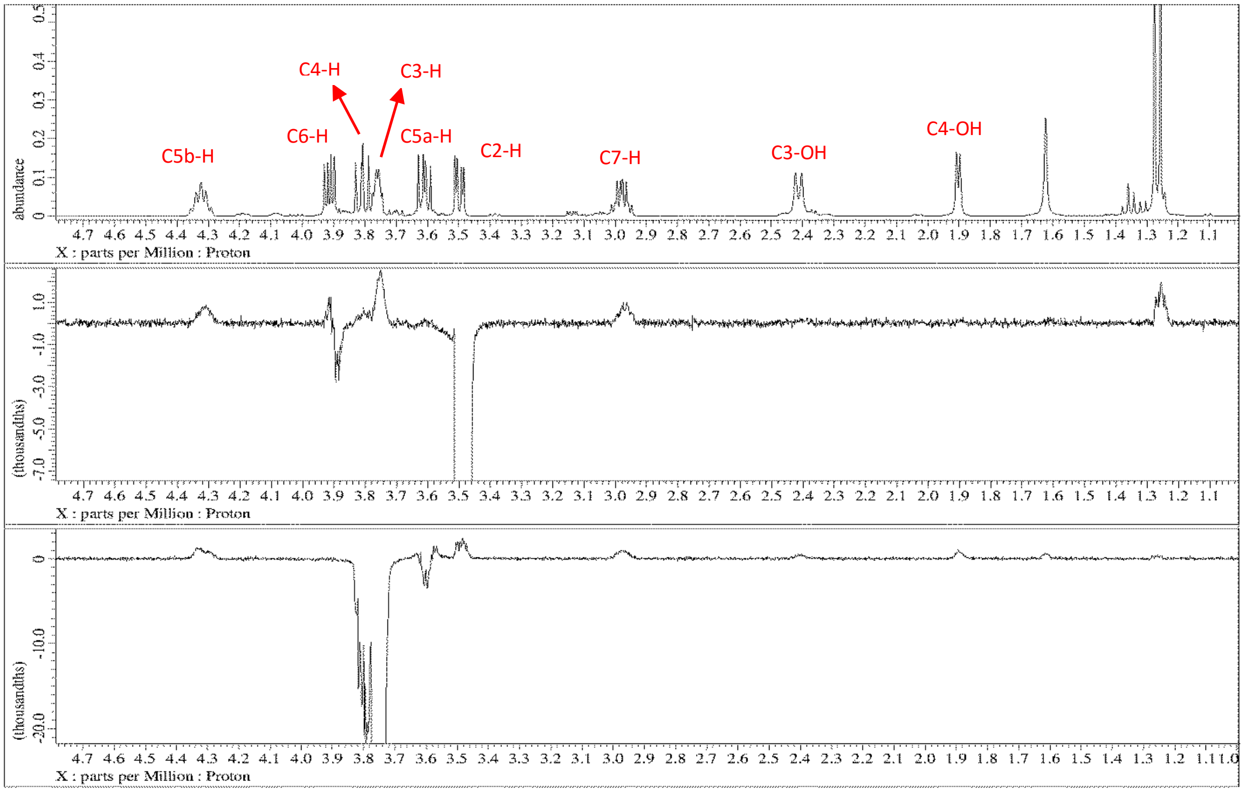

NOESY Experiment :

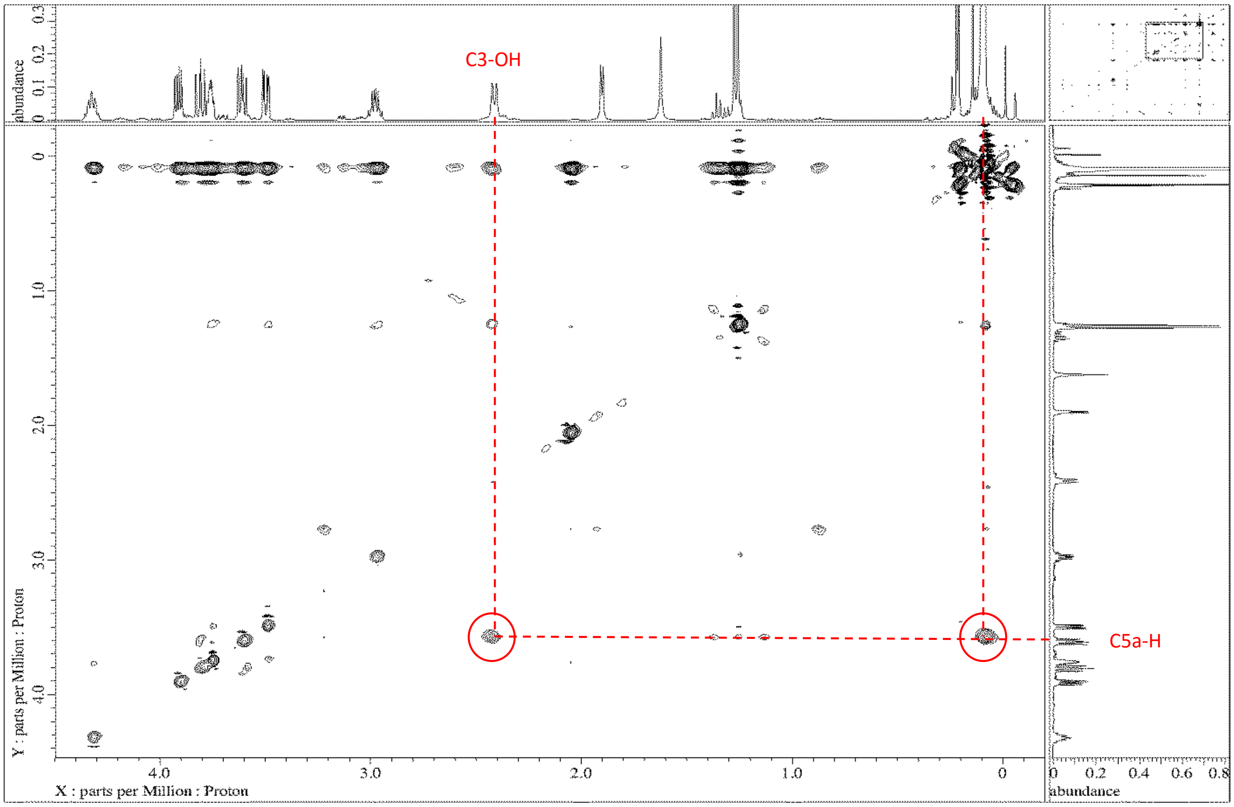

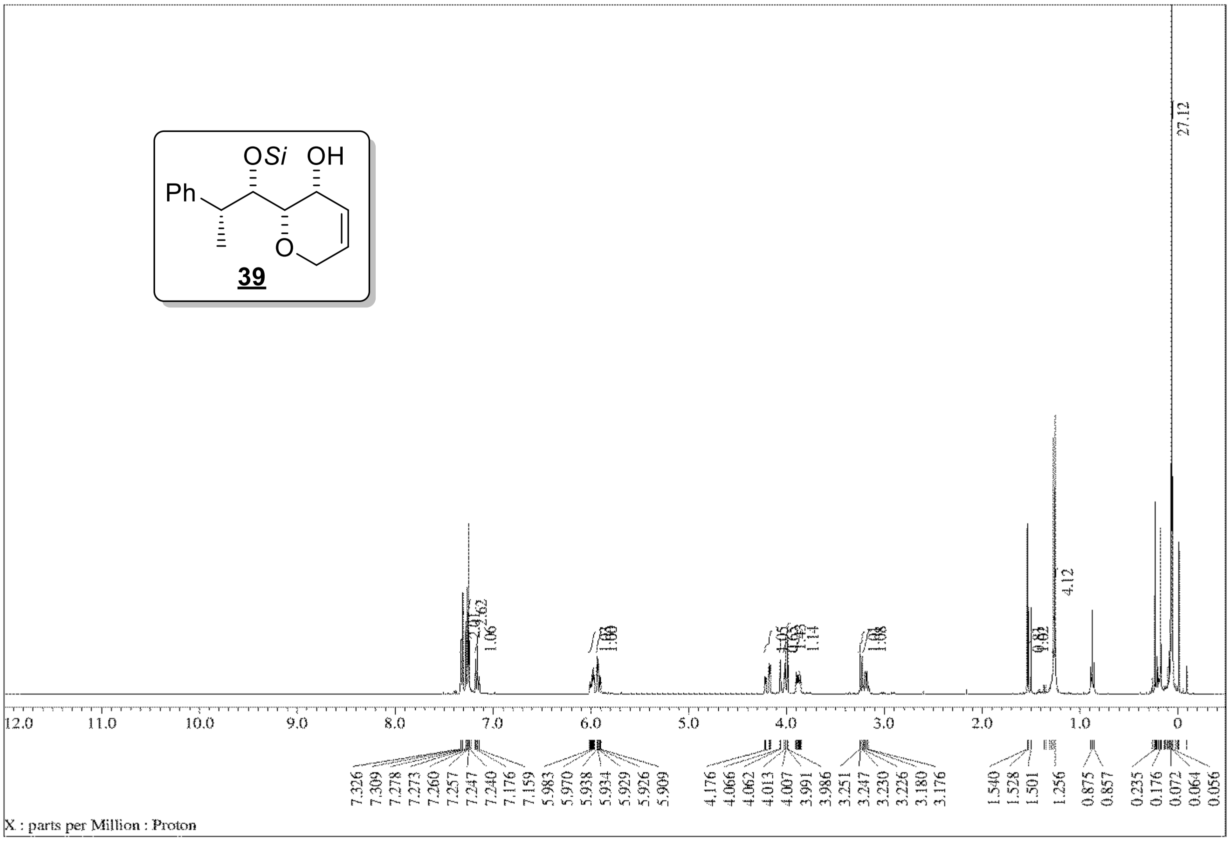
OSi OH
Ph
O
39
27.12
4.12
7.326
7.309
7.278
7.273
7.260
7.257
7.247
7.240
7.176
7.159
5.983
5.970
5.938
5.934
5.929
5.926
5.909
4.176
4.066
4.062
4.013
4.007
3.991
3.986
3.251
3.247
3.230
3.226
3.180
3.176
1.540
1.528
1.501
1.256
0.875
0.857
0.235
0.176
0.072
0.064
0.056
X : parts per Million : Proton

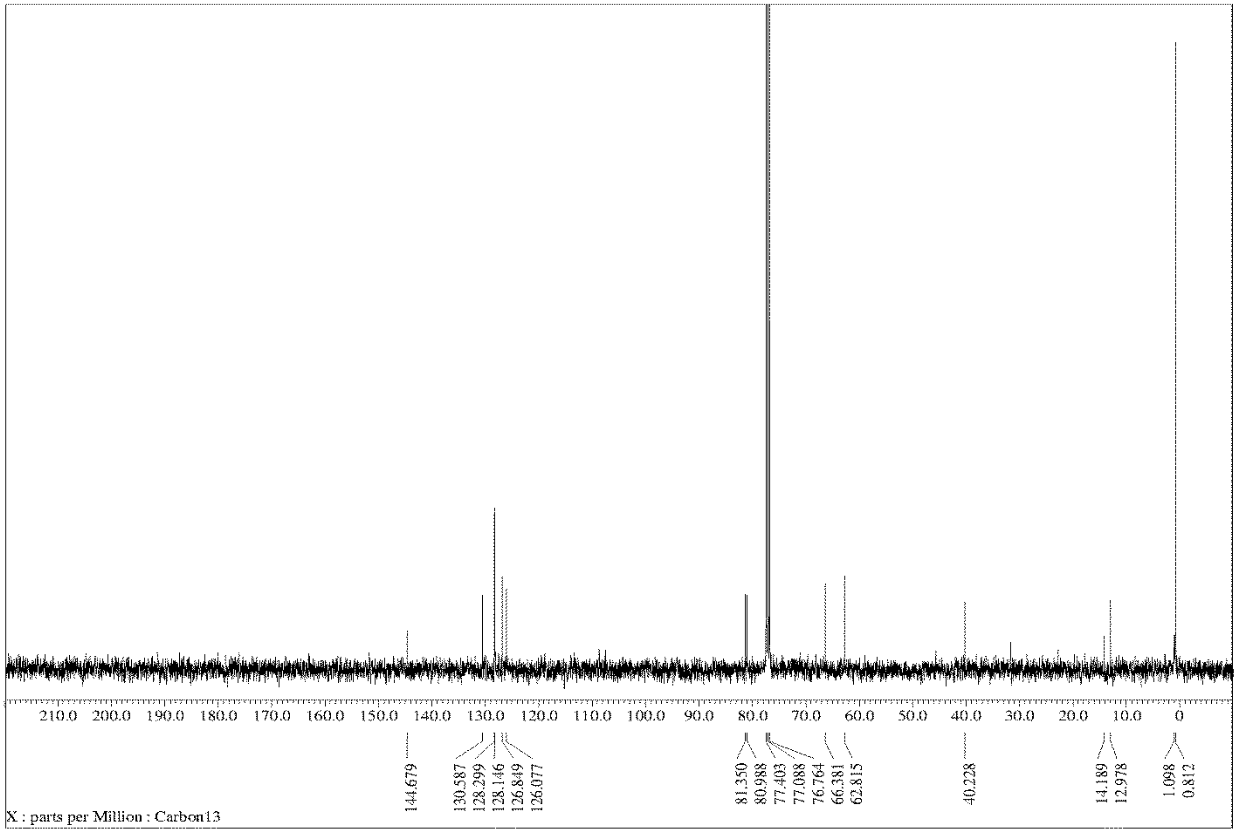
144.679
130.587
128.299
128.146
126.849
126.077
81.350
80.988
77.403
77.088
76.764
66.381
62.815
40.228
14.189
12.978
1.098
0.812
X : parts per Million : Carbon13

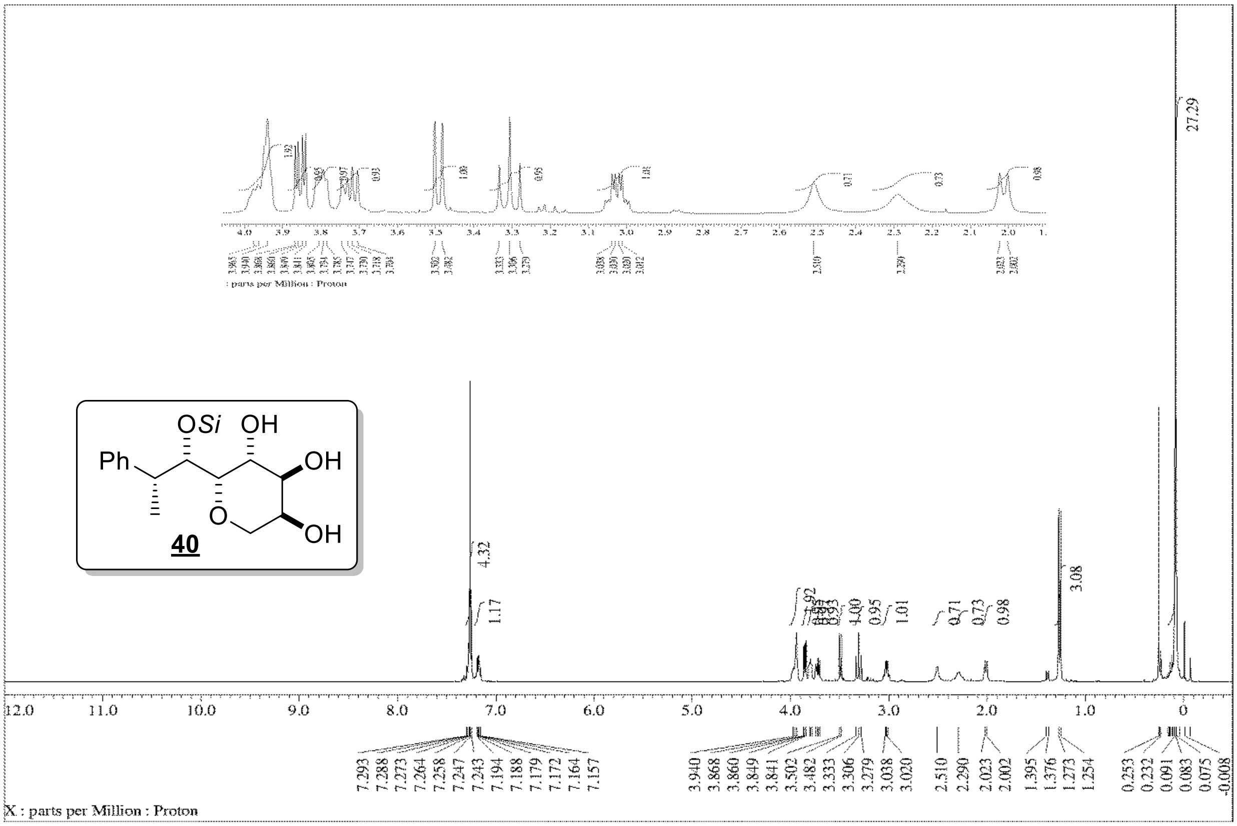
OSi OH
Ph
OH
O
OH
40
4.32
1.17
1.92
4.00
0.95
1.01
0.71
0.73
0.98
3.08
27.29
12.0 11.0 10.0 9.0 8.0 7.0 6.0 5.0 4.0 3.0 2.0 1.0 0
7.293 7.288 7.273 7.264 7.258 7.247 7.243 7.194 7.188 7.179 7.172 7.164 7.157
3.940 3.868 3.860 3.849 3.841 3.502 3.482 3.333 3.306 3.279 3.038 3.020
2.510 2.290 2.023 2.002
1.395 1.376 1.273 1.254
0.253 0.232 0.091 0.083 0.075 -0.008
X : parts per Million : Proton

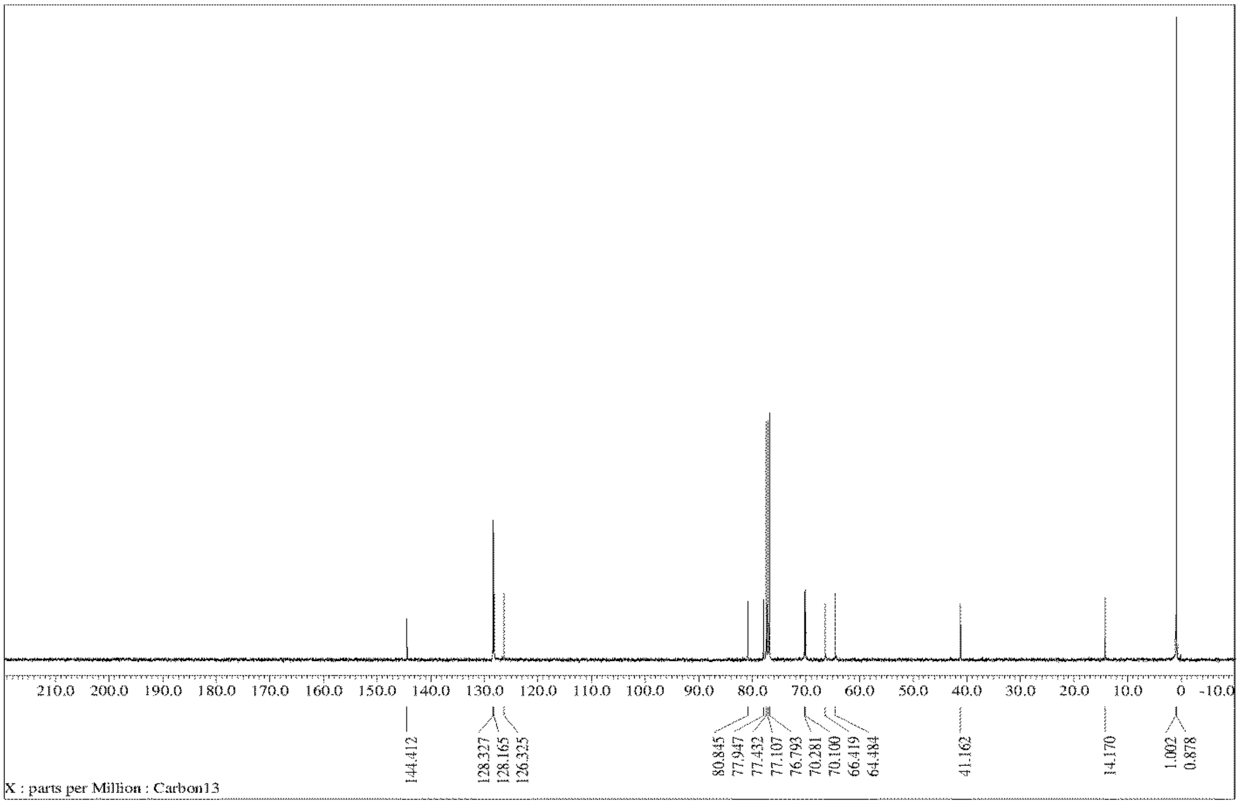
210.0 200.0 190.0 180.0 170.0 160.0 150.0 140.0 130.0 120.0 110.0 100.0 90.0 80.0 70.0 60.0 50.0 40.0 30.0 20.0 10.0 0 -10.0
144.412
128.327 128.165 126.325
80.845 77.947 77.432 77.107 76.793 70.281 70.100 66.419 64.484
41.162
14.170
1.002 0.878
X : parts per Million : Carbon13

8. ^{29}Si NMR study on the influence of iodobenzene

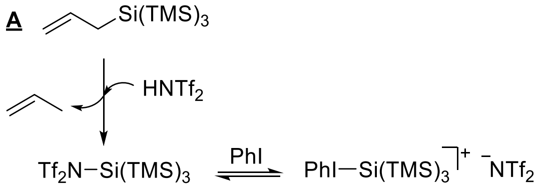

Influence of stoicheometrie

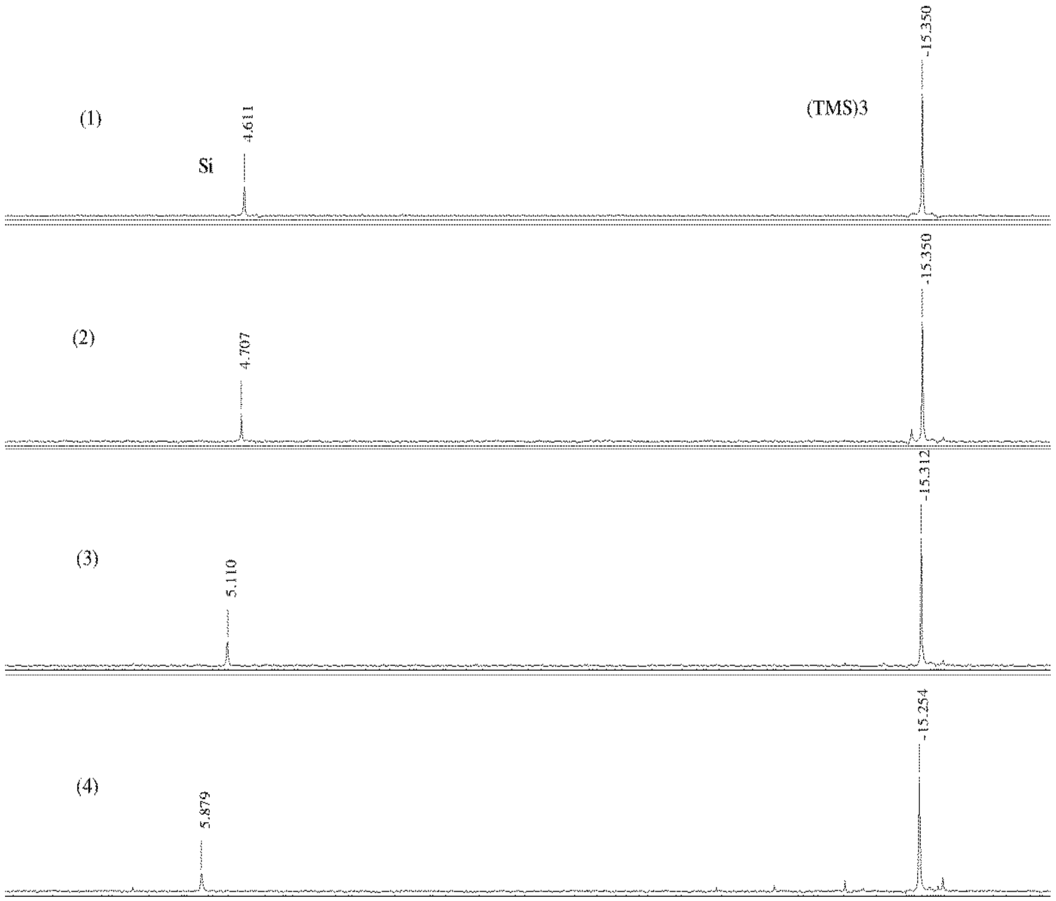

spectra (1) : reference ^{29}Si NMR spectra of substrate test in presence of triflimide

spectra (2) : 1 mmol of substrate **A**, 0.1 equiv. of iodobenzene and 1 mol% of $HNTf_2$ were successively added to NMR test tube.

spectra (3) : 1 mmol of substrate **A**, 1 equiv. of iodobenzene and 1 mol% of $HNTf_2$ were successively added to NMR test tube.

spectra (4) : 1 mmol of substrate **A**, 1.5 equiv. of iodobenzene and 1 mol% of $HNTf_2$ were successively added to NMR test tube.

All ^{29}Si NMR were performed in CD_2Cl_2 at room temperature and under nitrogen atmosphere.

Influence of reaction time

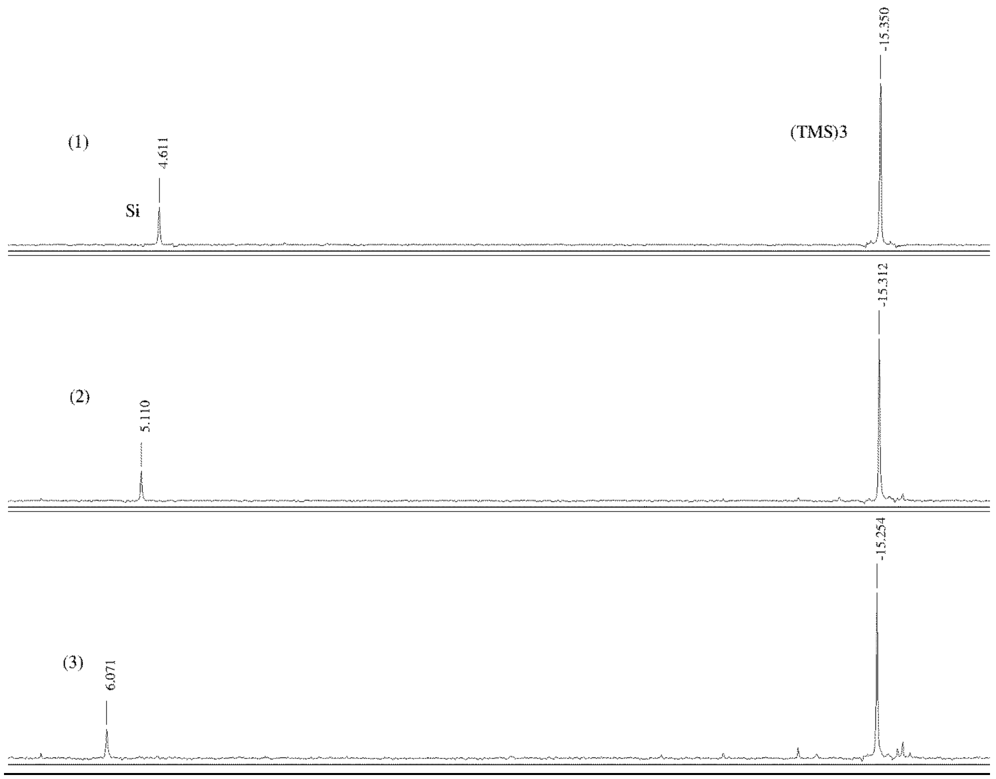

spectra (1) : reference ^{29}Si NMR spectra of substrate test in presence of triflimide

spectra (2) : 1 mmol of substrate **A**, 1 equiv. of iodobenzene and of $HNTf_2$ were successively added to NMR test tube. Spectra registered After 1 min of addition of $HNTf_2$.

spectra (3) : 1 mmol of substrate **A**, 1 equiv. of iodobenzene and of $HNTf_2$ were successively added to NMR test tube. Spectra registered After 45 min of addition of $HNTf_2$.

All ^{29}Si NMR were performed in CD_2Cl_2 at room temperature and under nitrogen atmosphere. 1 mol% of $HNTf_2$ was used.
